# Prevalence and epidemiological patterns of *Neisseria gonorrhoeae* infection in sub-Saharan Africa, 1964–2025: Systematic review, meta-analyses, and meta-regressions

**DOI:** 10.1371/journal.pmed.1004936

**Published:** 2026-06-23

**Authors:** Aisha Osman, Hina Akram, Bayan Alemrayat, Sumaya Al-Maraghi, Manale Harfouche, Laith J. Abu-Raddad

**Affiliations:** 1 Infectious Disease Epidemiology Group, Weill Cornell Medicine–Qatar, Cornell University, Qatar Foundation–Education City, Doha, Qatar; 2 Department of Population Health Sciences, Weill Cornell Medicine, Cornell University, New York, New York, United States of America; 3 Department of Public Health, College of Health Sciences, QU Health, Qatar University, Doha, Qatar; 4 College of Health and Life Sciences, Hamad bin Khalifa University, Doha, Qatar; University of Bern Faculty of Natural Sciences: Universitat Bern Philosophisch-naturwissenschaftliche Fakultat, SWITZERLAND

## Abstract

**Background:**

*Neisseria gonorrhoeae* (NG) infection is a global health concern because of its morbidity and increasing antimicrobial resistance. Sub-Saharan Africa is believed to carry a disproportionately high burden of NG infection, but the epidemiology of NG infection in this region has not been comprehensively synthesized. This study systematically reviewed and analyzed NG prevalence in sub-Saharan Africa to characterize prevalence patterns and identify populations at risk.

**Methods and findings:**

A systematic review was conducted and reported following PRISMA guidelines. Embase, PubMed, Scopus, and Web of Science were searched from inception to June 4, 2025. Eligible studies reported NG prevalence in sub-Saharan Africa. Random-effects meta-analyses generated pooled prevalence estimates, and random-effects meta-regression analyses identified associations and sources of heterogeneity.

Nine hundred fifty publications contributed 1,604 prevalence measures spanning 1964–2025. In the general population, pooled urogenital prevalence was 3.2% (95% confidence interval (CI): 2.9–3.5), with substantial between-study heterogeneity and a wide prediction interval, indicating considerable variation in prevalence across settings. Prevalence was high in key populations: among female sex workers, 11.5% (95% CI: 9.9–13.2) for urogenital and 2.0% (95% CI: 0.4–4.5) for anorectal infection; and among men who have sex with men, 2.8% (95% CI: 2.4–3.3) for urogenital, 8.3% (95% CI: 5.8–11.0) for anorectal, and 5.7% (95% CI: 3.6–8.3) for oropharyngeal infection. Symptomatic men exhibited high urogenital prevalence (51.5%; 95% CI: 47.5–55.5), and symptomatic women showed 9.0% (95% CI: 7.7–10.4). Among women with adverse pregnancy or birth outcomes, urogenital prevalence was 8.6% (95% CI: 5.3–12.6). Meta-regression analyses explained over half of the variability in prevalence, showing a long-term decline of 1% per year, a clear population type gradient, subregional differences, and decreasing prevalence with increasing age, but no variation by sex. These findings may be affected by variability in data availability across countries, anatomical sites, and population groups, as well as heterogeneity across included studies.

**Conclusions:**

NG prevalence remains markedly high in this region but has declined over time. These findings highlight the need for strengthened surveillance, expanded prevention and diagnostic strategies, and continued monitoring of gonococcal antimicrobial resistance to support effective control efforts in sub-Saharan Africa.

## Author summary

**Why was this study done?:**

- Gonorrhea is a common sexually transmitted infection that can cause serious health complications and is becoming harder to treat due to antimicrobial resistance.
- Sub-Saharan Africa is believed to carry a high burden of infection, but available evidence has not been comprehensively synthesized.
- Understanding who is most affected and how prevalence has changed over time is essential to guide prevention and control efforts.

**What did the researchers do and find?:**

- This study systematically reviewed nearly one thousand publications, contributing over 1,600 gonorrhea prevalence measures across sub-Saharan Africa over several decades.
- Average prevalence in the general population was approximately 3%, but was substantially higher in specific populations, including female sex workers, men who have sex with men, and individuals with symptoms or attending sexually transmitted infections clinics.
- Prevalence declined gradually over time but remains high, with marked variation across population groups, age categories, and subregions within sub-Saharan Africa.

**What do these findings mean?:**

- Gonorrhea remains a major public health challenge in sub-Saharan Africa, particularly among younger individuals and key populations at higher risk.
- Strengthened prevention strategies, expanded access to testing, and improved surveillance systems are needed to reduce transmission and address antimicrobial resistance.
- The findings are constrained by heterogeneity in study methods and uneven data availability across urogenital, anorectal, and oropharyngeal sites, as well as across countries and populations, which may affect the representativeness of the estimates.

## Introduction

Gonorrhea, caused by *Neisseria gonorrhoeae* (NG), is among the most common curable sexually transmitted infections (STIs) worldwide and remains a persistent public health challenge [1,2]. NG infects urogenital, anorectal, and oropharyngeal sites and is often asymptomatic—particularly among women—facilitating sustained and undetected transmission [1,2]. If untreated, NG infection can lead to serious complications, including pelvic inflammatory disease, ectopic pregnancy, and infertility in women, and epididymitis, prostatitis, and urethral stricture in men [1–4]. Globally, the World Health Organization (WHO) estimated 82.4 million new NG infections in 2020 [5,6], with increasing incidence reported in several countries in recent years [7,8].

At the time of writing, there is no licensed vaccine against NG, and control efforts rely on prevention, screening, and antimicrobial treatment. The public health threat posed by NG infection has intensified due to widespread antimicrobial resistance (AMR) and the emergence of extensively drug-resistant strains that limit effective treatment options [1,9]. In response, WHO has classified gonococcal AMR as a high-priority global concern and initiated a coordinated action plan to strengthen surveillance, guide treatment policies, and accelerate the development of new antimicrobials [10].

In parallel, WHO’s Global Health Sector Strategy on HIV, Viral Hepatitis, and STIs targets a 90% reduction in NG incidence by 2030 through expanded access to quality diagnosis, treatment, and evidence-based prevention interventions [11]. Advancing NG epidemiology is essential to achieving these objectives by informing national strategic planning, optimizing resource allocation and program performance, and supporting future pathways for potential NG vaccine introduction [11–13].

Sub-Saharan Africa faces a disproportionately high burden of STIs [14–17], including NG, in a context of constrained health resources and limited laboratory and surveillance capacity [18]. Generating robust regional estimates is therefore essential for informing national programs and guiding and optimizing targeted prevention and treatment strategies.

This study seeks to characterize NG epidemiology in sub-Saharan Africa through three core objectives: (1) systematically reviewing and synthesizing all available evidence on NG prevalence, (2) estimating pooled mean prevalence across diverse population groups, and (3) examining population-level associations with prevalence and identifying sources of between-study heterogeneity.

## Methods

### Data sources and search strategy

This systematic review was conducted in accordance with methodological standards established by the Cochrane Collaboration [19]. Findings are presented following the Preferred Reporting Items for Systematic Reviews and Meta-Analyses (PRISMA) guidelines [20], with the corresponding checklist provided in Table A in S1 Appendix.

The study protocol has been formally registered with the International Prospective Register of Systematic Reviews (PROSPERO) on 25 March 2026 (CRD420261349969). However, it was adapted from a previously published protocol [21] and further refined using methodological frameworks consistently applied in prior systematic reviews of NG and other STI prevalence, including those conducted for sub-Saharan Africa [3,17,22–29]. No substantive deviations or methodological modifications were introduced relative to previous regional systematic reviews of NG prevalence [22–25], consistent with the use of a standardized methodological approach, including all elements of the statistical analysis. Minor adaptations were made to the definition of population group categories to reflect region-specific contexts (Table 1).

**Table 1:** Population groups. Definitions of population categories used to classify NG prevalence measures.

Population	Definition
**General populations (low risk populations)**	Groups considered at relatively low risk of NG exposure, including antenatal clinic attendees, pregnant women, and individuals sampled from community-based settings not selected on the basis of high-risk behaviors or clinical presentation.
**Intermediate-risk populations**	Groups presumed to have sexual contact with individuals engaging in high-risk sexual behavior, resulting in a greater risk of NG exposure than the general population. Examples include prisoners, homeless people, people who inject drugs, migrant workers, and truck drivers.
**Female sex workers**	Women who engage in sex work, defined as the exchange of sexual services for money.
**Men who have sex with men**	Men who engage in same-sex sexual activity, particularly anal intercourse with other men.
**Symptomatic women**	Women with clinical manifestations related to NG infection, such as presenting with vaginal discharge.
**Symptomatic men**	Men with clinical manifestations related to NG infection, such as presenting with urethral discharge.
**Symptomatic women and men**	Populations of undetermined sex with clinical manifestations related to NG infection, such as vaginal or urethral discharge.
**Infertility clinic attendees**	Categorized separately due to uncertainty regarding their risk of NG exposure and the potential biological association between NG infection and infertility.
**Women with adverse pregnancy or birth outcomes**	Categorized separately because of uncertainty regarding their risk of NG exposure and the potential biological links to NG infection. Adverse pregnancy or birth outcomes were defined to include miscarriage, ectopic pregnancy, stillbirth, preterm delivery, small-for-gestational-age infants, and related complications.
**STI clinic attendees**	Individuals attending or seeking care at STI clinics.
**Individuals living with HIV and individuals in HIV-discordant couples**	Individuals living with HIV or those in a spousal relationship with an individual living with HIV.
**Sexual contacts of persons infected with NG/CT**	Individuals who have had sexual contact with persons infected with NG or CT.
**Patients with confirmed or suspected STIs and related infections**	Individuals diagnosed with an STI or suspected of having concomitant STIs or other related infections.
**Other populations**	Groups that do not fit the above definitions or have an undetermined risk of acquiring NG infection, such as cervical cancer patients, individuals evaluated following sexual assault, and mixed or undefined populations.

Abbreviations: CT, *Chlamydia trachomatis*; HIV, Human immunodeficiency virus; NG, *Neisseria gonorrhoeae*; STI, Sexually transmitted infection.

A comprehensive literature search was conducted across Embase, PubMed, Scopus, and Web of Science, with two updates, capturing all records from inception through June 4, 2025, the date of the final updated search. Broad and inclusive search criteria were used, combining indexed terms—with all associated subheadings—and free-text keywords, without imposing restrictions on any language or publication year. Where necessary, titles, abstracts, and full texts were translated using Google Translate to facilitate screening and data extraction of non-English articles. Full search strategies are provided in Table B in S1 Appendix.

The search strategy was intentionally designed to maximize sensitivity by focusing on NG-related terms (pathogen and disease) and geographic identifiers, without restricting by outcome-specific terms such as “prevalence.” This approach aimed to avoid missing relevant studies that reported prevalence estimates but were not indexed or described using explicit prevalence-related terminology. Accordingly, the operational definition of prevalence was applied during the screening and data extraction stages rather than within the search strategy. Specifically, studies were eligible if they reported sufficient data to compute prevalence.

Prevalence was defined as the proportion of individuals tested who had a laboratory-confirmed NG infection at the time of testing, corresponding to point prevalence within the sampled population. The numerator was the number of individuals with confirmed NG infection, and the denominator was the total number of individuals tested. In some included studies, particularly those based on clinic- or other facility-based samples, this measure reflects test positivity rather than population-representative prevalence.

The definition of the sub-Saharan Africa region in this study reflects conventions applied in previous STI research [17,26] and follows the regional classifications adopted by WHO Regional Office for Africa and the Joint United Nations Programme on HIV/AIDS (UNAIDS). The list of included countries, together with their subregional groupings, is presented in Box A in S1 Appendix. Mauritania is classified by WHO within the African Region rather than the Eastern Mediterranean Region and was therefore included in this study.

To complement database searches, the UNAIDS database of HIV integrated biobehavioral surveys (IBBS) was also reviewed to identify additional eligible data sources.

### Study selection and eligibility criteria

The search results were imported into EndNote (Clarivate Analytics, London, United Kingdom), where duplicates were systematically removed. The remaining records were then screened for eligibility by at least two independent reviewers (AO, HA, BA, and SA). Screening proceeded in two stages: an initial assessment of titles and abstracts, followed by full-text review of records deemed relevant or potentially relevant. Discrepancies at any stage were resolved through reviewer discussion and consensus, with final adjudication by LJA if needed.

Publications were eligible for inclusion if they reported primary data on NG prevalence from any country in sub-Saharan Africa (Box A in S1 Appendix), were based on specimens directly collected from humans, and used laboratory diagnostic methods—such as nucleic acid amplification tests (NAAT)/polymerase chain reaction (PCR), culture, or Gram stain—to detect NG infection. Eligible studies included those reporting NG detection from urogenital (e.g., urine, urethral, endocervical, or vaginal), anorectal, or oropharyngeal specimens.

Publications were excluded if they relied on self-reported infection status, included fewer than 10 participants, or collected specimens from the upper genital tract. Case reports, case series, commentaries, reviews, and qualitative studies were also excluded. Bibliographies of relevant articles and reviews were screened to identify additional publications.

In this review, a “report/publication” refers to any document that provides NG prevalence data for one or more populations, whereas a “study” denotes a specific prevalence estimate derived from a particular population. When duplicate prevalence estimates were identified across records, selection followed a predefined hierarchy: the most recent estimate was retained; if publication years were identical, the record with the largest sample size was selected; and if both were identical, the record with the most detailed extractable data was prioritized.

### Data extraction

Data from eligible publications were independently extracted and double extracted by at least two reviewers (AO, HA, BA, SA, and MH), with discrepancies resolved through consensus and input from LJA when needed. Extracted variables were pre-piloted and are summarized in Box B in S1 Appendix. Both overall prevalence measures (i.e., for the total sample) and stratified estimates were extracted when the sample size within each stratum was at least 10. Stratification of prevalence measures followed a predefined hierarchy: anatomical site, population type, sex, year of data collection, age group, and region/city.

Population groups were defined according to primary study classifications and grouped into distinct domains, including behavioral or occupational risk groups (e.g., female sex workers (FSWs) and men who have sex with men (MSM)), clinical presentation groups (e.g., symptomatic individuals), and healthcare-seeking populations (e.g., STI clinic attendees). Definitions of population type classifications are provided in Table 1. These categories are not mutually exclusive and may overlap.

When multiple anatomical sites were assessed in the same participants, site-specific prevalence estimates (urogenital, anorectal, or oropharyngeal) were extracted separately when available. When studies reported a combined measure across anatomical sites, the estimate was retained as reported and classified under the unspecified/mixed anatomical site category.

For studies using the same diagnostic assay across multiple biological specimens from the same anatomical site (e.g., urogenital), only one prevalence measure was retained. For women, priority was given to endocervical swabs, followed by vaginal swabs, and then urine specimens; for men, urethral swabs were prioritized, followed by urine and semen specimens. When different assay types were applied to the same biological specimen, each assay-specific estimate was extracted separately. This approach enabled the evaluation of diagnostic-method effects on NG prevalence through meta-regression analyses and supported the derivation of adjustment factors for STI estimation in mathematical modeling studies [14,30].

When prevalence estimates were reported for samples including both women and men, sex classification was assigned according to the predominant sex in the sample (≥60%). Prevalence data for individuals younger than 15 years were extracted but excluded from meta-analyses.

### Precision, risk of bias, and publication bias assessments

The precision and risk of bias of included studies were independently assessed by at least two reviewers (AO, HA, BA, and MH), with input from LJA. As this review did not evaluate management strategies or clinical interventions [31], the assessment approach was tailored to prevalence studies, incorporating established quality criteria [32,33], building on methods applied in prior systematic reviews of STI prevalence [22–24,27–29,34,35], and informed by the Cochrane framework [19]. The final assessment framework included one criterion for precision and two criteria for risk of bias.

Additional quality dimensions were not formally assessed because they were inherently satisfied through the review’s design and eligibility criteria or were more appropriately examined through subsequent analyses within the study (Table C in S1 Appendix). For example, the reliability of diagnostic assays used to estimate NG prevalence was evaluated through meta-regression analyses assessing the effect of assay type on prevalence.

Study precision was classified as low (<200 participants) or high (≥200 participants). Risk of bias was categorized as low or high based on sampling methodology (probability-based versus non-probability-based) and response rate (≥80% versus <80%). When information was insufficient, risk of bias was rated as unclear. These quality indicators were also incorporated into meta-regression analyses to examine their potential influence on NG prevalence estimates, consistent with methods applied in prior systematic reviews [22–24,27–29,34,35].

Publication bias was assessed using Doi plots and the Luis Furuya–Kanamori (LFK) index when at least three studies were available [36]. This approach assumes that, in the absence of bias, study estimates are symmetrically distributed around the pooled estimate, with asymmetry potentially reflecting publication bias, including small-study effects, among others [36]. Accordingly, asymmetry in the Doi plot was interpreted as indicative of potential publication bias, and an absolute LFK index value greater than 1 was considered suggestive of such bias [36].

### Meta-analyses

Stratified prevalence estimates were summarized using medians and ranges. Meta-analyses of stratified NG prevalence were conducted using DerSimonian–Laird random-effects models [37], applying the Freeman–Tukey double arcsine transformation to stabilize variance [38] after confirming its suitability for these data [39]. Pooled mean prevalence estimates and corresponding 95% confidence intervals (CIs) were generated for each population type, stratified by anatomical site and assay type, when at least three observations were available. Forest plots were used to display pooled estimates and illustrate between-study variability.

Study-specific standard errors were derived directly from the number of events (or the effective number of events for studies with complex sampling designs) and sample size under the selected transformation, rather than being back-calculated from reported CIs. These standard errors on the transformed scale were then used for inverse-variance weighting in the meta-analyses and in the meta-regression models described below within a random-effects framework.

Heterogeneity across studies was assessed using Cochran’s Q statistic, with *p* < 0.1 indicating significant heterogeneity. The I² statistic quantified the proportion of total variability attributable to true differences in prevalence rather than sampling error, while prediction intervals were calculated to characterize the expected range of true prevalence values around the pooled mean [40]. All analyses were performed in R version 4.1.3 (R Foundation for Statistical Computing, Vienna, Austria) using the *meta* package [41].

Given the substantial heterogeneity observed in prevalence estimates, pooled mean values were interpreted as summary measures reflecting averages across diverse studies rather than precise estimates of underlying prevalence [29,34]. To investigate sources of this heterogeneity, meta-regression analyses were undertaken to identify epidemiologic and methodological factors associated with variation in NG prevalence across studies.

### Meta-regression analyses

Univariable and multivariable random-effects meta-regression analyses were conducted to investigate sources of between-study heterogeneity and identify factors associated with NG prevalence. Log-transformed prevalence estimates were used [42], with the log transformation preferred over the logit transformation to enable estimation of prevalence ratios (PRs), which provide a more epidemiologically interpretable measure of association than prevalence odds ratios [27].

Selection of variables for inclusion in the meta-regression models was informed by epidemiologic relevance, prior evidence from HIV/STI research, and the need to evaluate potential sources of bias in the available prevalence estimates [22–24,27–29,34,35]. Candidate variables are listed in Box C in S1 Appendix. Variables with a *p*-value ≤0.2 in univariable analyses were considered for entry into the multivariable model, where a *p*-value ≤0.05 was taken to indicate a statistically significant association with NG prevalence. Model performance was quantified using the adjusted *R*², reflecting the proportion of between-study heterogeneity explained.

All meta-regression models were specified within a normally distributed random-effects framework, in which the random-effects term captures residual between-study heterogeneity not explained by the included covariates, thereby allowing for variation in true underlying prevalence across studies beyond sampling error.

For studies that did not report the year of data collection, this value was imputed as the publication year minus the median difference between publication year and data collection year among studies providing both. This approach preserved the temporal structure of the dataset while limiting potential bias. Meta-regression analyses were performed in Stata/SE version 16 (StataCorp, College Station, TX, USA) using the *metareg* package [42].

### Ethics

This study was conducted in accordance with established ethical guidelines and principles. As all data were obtained from publicly available sources, and no primary data collection involving human participants was conducted, formal ethics approval was not required.

### Role of the funding source

The funder of the study had no role in study design, data collection, data analysis, data interpretation, or writing of the article. HA, BA, MH, and LJA had full access to all the data in the study and had the final responsibility for the decision to submit for publication.

## Results

### Search results and scope of evidence

**Fig 1:**
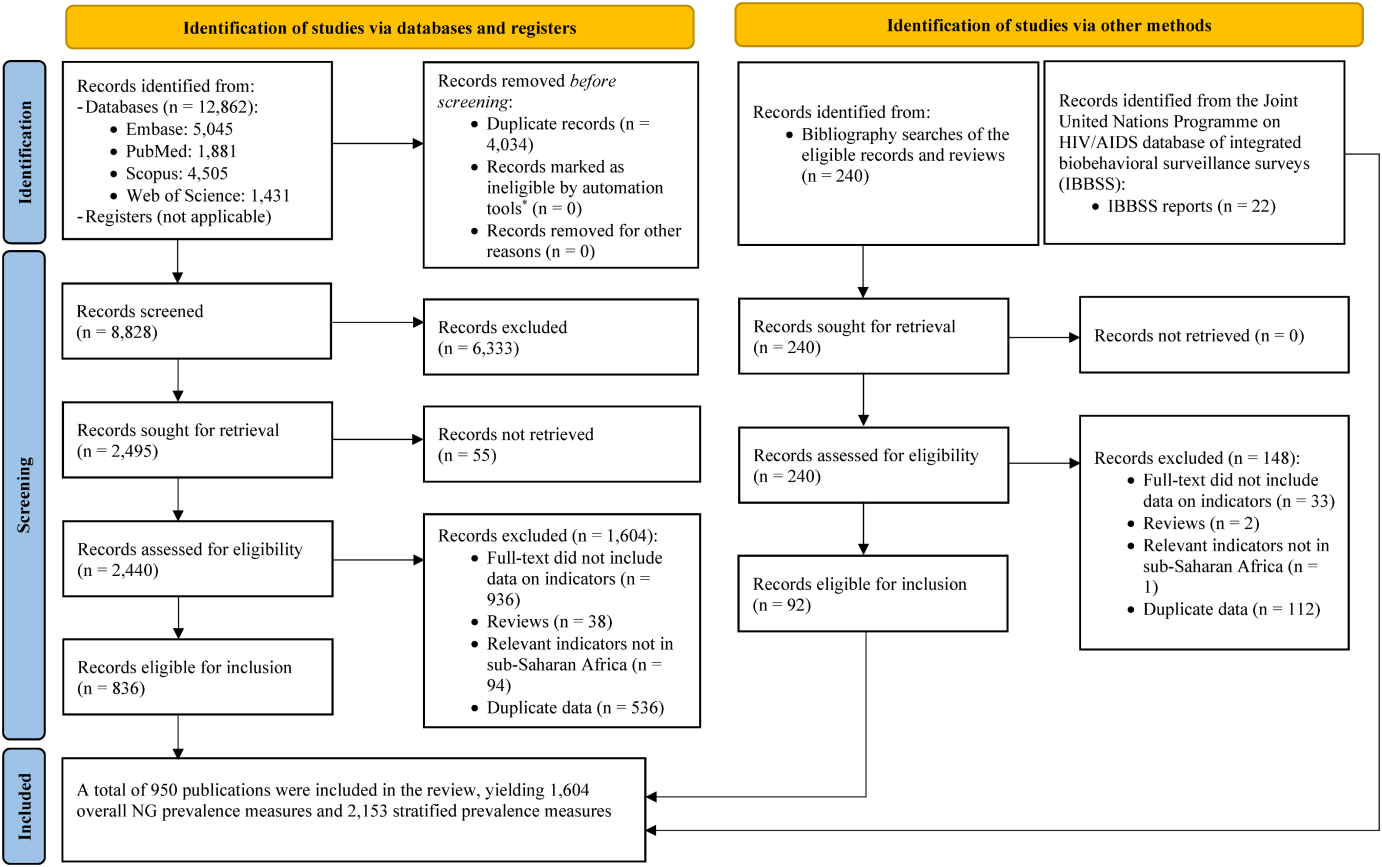
Study selection. Flowchart illustrating the study selection process for assessing NG prevalence in sub-Saharan Africa, presented according to PRISMA guidelines [20]. Abbreviations: AIDS, Acquired immunodeficiency syndrome; HIV, Human immunodeficiency virus; NG, *Neisseria gonorrhoeae*. ^*^“Marked as ineligible by automation tools” refers to records excluded during initial screening using automated or semi-automated tools (e.g., screening software) based on predefined criteria, such as non-relevant keywords or clearly ineligible study designs. In this study, title and abstract screening were conducted manually, and no automation tools were used.

Figure 1 presents the PRISMA flow diagram summarizing the study selection process. The systematic search across databases identified 12,862 records (5,045 from Embase, 1,881 from PubMed, 4,505 from Scopus, and 1,431 from Web of Science). After removal of duplicates and screening of titles and abstracts, 2,440 publications were assessed for eligibility through full-text review, of which 836 met the inclusion criteria. An additional 92 eligible publications were identified through bibliographic screening. Furthermore, 22 relevant IBBS reports were retrieved from the UNAIDS database and included in the evidence base.

In total, 950 publications met the inclusion criteria and were incorporated into this study (citations listed in Table D in S1 Appendix). Together, these publications contributed 1,604 overall NG prevalence measures and 2,153 stratified measures, generating a large dataset encompassing approximately 2.7 million individuals.

The dataset comprised 1,362 overall urogenital measures (1,869 stratified), 40 overall anorectal measures (50 stratified), 15 overall oropharyngeal measures (17 stratified), 159 overall measures derived from unspecified anatomical sites or mixed specimens (186 stratified), and 28 overall serological measures (31 stratified).

Regionally, Southern Africa contributed the largest share of prevalence measures (*n* = 536; 33.4%), followed by Eastern Africa (*n* = 519; 32.4%), Western Africa (*n* = 421; 26.2%), Central Africa (*n* = 90; 5.6%), and Northern Africa, represented solely by Mauritania (*n* = 2; 0.1%). A further 36 studies (2.2%) reported data spanning multiple subregions. At the country-level, South Africa provided the highest number of measures (*n* = 308), with Kenya (*n* = 207) and Nigeria (*n* = 168) also making substantial contributions. The geographical distribution of studies reporting NG prevalence across countries is shown in Fig A in S1 Appendix.

Prevalence estimates were drawn from a wide range of population groups. Among the most frequently represented, general populations accounted for 34.7% (*n* = 557) of all measures; FSWs, 9.1% (*n* = 146); MSM, 4.6% (*n* = 74); symptomatic men, 13.0% (*n* = 208); symptomatic women, 12.2% (*n* = 196); and STI clinic attendees, 4.6% (*n* = 74).

The included prevalence measures spanned several decades, with the earliest study conducted in 1964. Overall, 46.9% of studies (*n* = 752) were published before 2005, 22.4% (*n* = 360) between 2005 and 2014, and 30.7% (*n* = 492) in 2015 or thereafter. Fig B in S1 Appendix shows the temporal trend in NG prevalence estimates across all population groups, indicating a declining trend over time. Fig C in S1 Appendix presents subregion-specific trends within sub-Saharan Africa, similarly indicating a declining trend over time across all subregions.

## Assessment of study precision, risk of bias, and publication bias

Table E in S1 Appendix presents the assessments of precision and risk of bias for the included studies. Among the 1,604 prevalence measures, 1,022 (63.7%) were derived from studies with sample sizes of at least 200 participants, indicating high precision, while the remaining 582 (36.3%) were classified as low precision (sample size <200).

A total of 278 studies (17.3%) used probability-based sampling and were therefore classified as having a low risk of bias in the sampling method domain. In the response rate domain, 232 studies (14.5%) demonstrated a low risk of bias (response rate ≥80%), while 60 studies (3.7%) showed a high-risk of bias due to response rates <80%. The majority of studies (1,312; 81.8%) did not report response rates and were consequently assigned an unclear risk of bias for this domain.

Overall, 435 studies (27.1%) showed a low risk of bias in at least one domain; however, only 75 studies (4.7%) demonstrated low risk of bias across both domains. Conversely, 38 studies (2.4%) exhibited high-risk of bias in both the sampling method and response rate domains.

Table F in S1 Appendix summarizes the assessment of publication bias. For the most epidemiologically relevant populations, such as general populations and MSM, the Doi plots were symmetrical and the LFK index values indicated no evidence of bias or, at most, a small degree of bias. In contrast, some facility-based populations showed asymmetrical Doi plots and absolute LFK index values greater than 1, suggesting potential publication bias (Figs D, E, and F in S1 Appendix).

### Pooled mean estimates for NG prevalence

Table 2 provides an overview of stratified NG prevalence estimates, reporting medians and ranges alongside pooled mean prevalence by population type and anatomical site. Additional stratification by assay type is presented in Table G in S1 Appendix. Forest plots illustrating pooled estimates for urogenital, anorectal, and oropharyngeal infections are shown in Figs G, H, and I in S1 Appendix, respectively.

**Table 2:** NG prevalence estimates. Pooled mean prevalence of NG infection in sub-Saharan Africa, stratified by population type and anatomical site.

Population type	Stratified prevalence measures	Sample	NG prevalence (%)		Pooled mean NG prevalence (%)	Heterogeneity measures		
**Total *n***	**Total *N***	**Range**	**Median**	**Mean** **(95% CI)**	**Q** ^*^ **(*p*-value)**	**I²**^†^ **(%)** **(95% CI)**	**Prediction interval**^**‡**^ **(%)**
**General populations**								
Urogenital	706	321,056	0.0–71.8	2.8	3.2 (2.9–3.5)	11,265.3 (*p* < 0.001)	93.7 (93.4–94.0)	0.0-15.0
Anorectal	4	2,180	0.0–4.8	1.7	1.9 (0.5–4.2)	22.0 (*p* < 0.001)	86.3 (66.8–94.4)	0.0-12.3
Oropharyngeal	4	915	0.0–0.0	0.0	0.0 (0.0–0.0)	1.0 (*p* = 0.799)	0.0 (0.0–84.7)	0.0-0.0
Blood tested for antibodies^§^	9	623	0.0–28.0	18.0	15.0 (7.6–24.2)	47.6 (*p* < 0.001)	83.2 (69.5–90.7)	0.0-50.7
Unspecified/mixed	74	2,052,616	0.0–24.5	4.9	3.7 (2.7–4.9)	13,961.9 (*p* < 0.001)	99.5 (99.4–99.5)	0.0-17.7
**Intermediate-risk populations**								
Urogenital	99	38,473	0.0–30.0	1.7	2.3 (1.6–3.0)	1,126.0 (*p* < 0.001)	91.3 (90.0–92.5)	0.0-11.7
Anorectal	2	598	0.0–0.0	0.0	0.0 (0.0–0.6)	–	–	–
Blood tested for antibodies^§^	1	2,650	–	–	1.6 (1.1–2.1)	–	–	–
Unspecified/mixed	1	81	–	–	14.8 (7.9–24.4)	–	–	–
**Female sex workers**								
Urogenital	200	67,409	0.0–77.5	10.3	11.5 (9.9–13.2)	6,778.0 (*p* < 0.001)	97.1 (96.8–97.3)	0.0–42.3
Anorectal	3	1,115	0.9–3.7	2.0	2.0 (0.4–4.5)	8.1 (*p* = 0.017)	75.4 (18.6–92.5)	0.0–15.9
Blood tested for antibodies^§^	1	642	–	–	53.3 (49.3–57.2)	–	–	–
Unspecified/mixed	8	3,032	1.0–32.3	8.5	9.2 (4.0–16.0)	148.4 (*p* < 0.001)	95.3 (92.7–97.0)	0.0–38.7
**Men who have sex with men** ^**¶**^								
Urogenital	46	11,993	0.0–9.0	3.0	2.8 (2.4–3.3)	73.3 (*p* = 0.049)	38.6 (12.1–57.0)	1.1-5.1
Anorectal	36	9,893	0.0–40.0	8.0	8.3 (5.8–11.0)	610.9 (*p* < 0.001)	94.3 (92.9–95.4)	0.0–29.2
Oropharyngeal	9	4,091	0.9–13.0	6.4	5.7 (3.6–8.3)	40.2 (*p* < 0.001)	80.1 (63.0–89.3)	0.5–15.7
Unspecified/mixed	14	2,912	0.0–18.0	2.2	4.0 (1.7–7.1)	171.9 (*p* < 0.001)	92.4 (89.0–94.8)	0.0–21.0
**Symptomatic women**								
Urogenital	237	54,600	0.0–70.1	8.0	9.0 (7.7–10.4)	4,957.7 (*p* < 0.001)	95.2 (94.9–95.6)	0.0–36.9
Blood tested for antibodies^§^	3	411	12.5–54.6	32.8	31.5 (10.2–58.0)	52.2 (*p* < 0.001)	96.2 (91.9–98.2)	0.0–100.0
Unspecified/mixed	15	2,369	2.6–54.7	14.3	16.3 (9.5–24.4)	263.3 (*p* < 0.001)	94.7 (92.6–96.2)	0.0–54.7
**Symptomatic men**								
Urogenital	248	57,220	0.0–100.0	57.3	51.5 (47.5–55.5)	21,093.3 (*p* < 0.001)	98.8 (98.8–98.9)	2.5–98.5
Anorectal	3	391	0.9–32.1	28.0	16.3 (0.6–45.2)	87.4 (*p* < 0.001)	97.7 (95.6–98.8)	0.0–100.0
Oropharyngeal	3	200	3.8–10.4	6.2	5.1 (2.2–8.8)	2.0 (*p* = 0.372)	0.0 (0.0–89.6)	0.2–14.3
Blood tested for antibodies^§^	2	391	10.6–13.7	12.2	12.3 (9.2–15.9)	–	–	–
Unspecified/mixed	15	2,729	9.7–93.3	49.9	53.2 (39.8–66.4)	627.9 (*p* < 0.001)	97.8 (97.1–98.3)	5.2–97.4
**Symptomatic women and men**								
Urogenital	48	8,593	0.0–87.0	13.4	18.2 (12.4–24.9)	2,739.5 (*p* < 0.001)	98.3 (98.1–98.5)	0.0–72.1
Unspecified/mixed	4	634	12.1–81.5	40.9	42.4 (10.4–78.5)	282.8 (*p* < 0.001)	98.9 (98.4–99.3)	0.0–100.0
**Infertility clinic attendees**								
Urogenital	20	2,316	0.0–17.9	3.3	3.0 (1.2–5.4)	152.6 (*p* < 0.001)	87.6 (82.2–91.3)	0.0–17.7
Blood tested for antibodies^§^	4	324	25.0–65.0	38.9	41.1 (23.3–60.1)	29.8 (*p* < 0.001)	89.9 (77.1–95.6)	0.0–96.1
**Women with adverse pregnancy or birth outcomes** ^**||**^								
Urogenital	16	1,838	0.0–31.2	8.7	8.6 (5.3–12.6)	124.8 (*p* < 0.001)	88.0 (82.1–91.9)	0.0–28.0
Blood tested for antibodies^§^	4	150	14.3–49.0	41.2	37.0 (22.3–53.0)	10.1 (*p* = 0.018)	70.4 (15.0–89.7)	1.2–85.1
Unspecified/mixed	2	480	11.0–25.4	18.2	12.9 (10.0–16.3)	–	–	–
**STI clinic attendees**								
Urogenital	65	26,575	0.0–59.7	13.9	15.5 (12.0–19.5)	4,186.6 (*p* < 0.001)	98.5 (98.3–98.6)	0.0–53.2
Unspecified/mixed	23	9,264	2.3–69.0	16.1	20.2 (13.5–27.8)	1,485.0 (*p* < 0.001)	98.5 (98.2–98.8)	0.0–63.6
**Individuals living with HIV and individuals in HIV-discordant couples**								
Urogenital	81	28,154	0.0–35.0	3.0	3.5 (2.6–4.4)	797.3 (*p* < 0.001)	90.0 (88.2–91.5)	0.0–13.9
Anorectal	2	116	22.0–31.3	26.6	23.3 (15.9–32.0)	–	–	–
Oropharyngeal	1	100	–	–	25.0 (16.9–34.7)	–	–	–
Blood tested for antibodies^§^	1	20	–	–	30.0 (11.9–54.3)	–	–	–
Unspecified/mixed	14	9,752	0.0–13.0	2.5	2.7 (1.1–4.9)	138.9 (*p* < 0.001)	90.6 (86.1–93.7)	0.0–13.4
**Sexual contacts of persons infected with NG/CT**								
Urogenital	3	133	38.0–68.0	41.0	50.0 (31.0–69.1)	9.9 (*p* = 0.007)	79.9 (36.3–93.6)	0.0–100.0
Unspecified/mixed	3	103	22.0–32.1	30.0	25.9 (17.7–35.1)	1.3 (*p* = 0.534)	0.0 (0.0–89.6)	9.4–46.7
**Patients with confirmed or suspected STIs and related infections**								
Urogenital	25	7,619	0.7–97.6	12.2	24.3 (12.0–39.1)	4,584.7 (*p* < 0.001)	99.5 (99.4–99.5)	0.0–96.2
Blood tested for antibodies^§^	1	100	–	–	97.0 (91.5–99.4)	–	–	–
Unspecified/mixed	3	377	7.2–20.2	9.7	11.7 (5.1–20.4)	10.0 (*p* = 0.007)	80.0 (36.7–93.7)	0.0–54.0
**Other populations** ^**^								
Urogenital	75	24,441	0.0–97.8	7.0	10.4 (7.2–14.0)	2,628.0 (*p* < 0.001)	97.2 (96.8–97.5)	0.0–51.9
Blood tested for antibodies^§^	5	2,222	31.1–68.8	47.2	49.4 (34.8–64.1)	115.2 (*p* < 0.001)	96.5 (94.1–97.9)	7.2–92.2
Unspecified/mixed	10	10,243	0.6–95.2	13.0	22.2 (5.1–46.4)	592.0 (*p* < 0.001)	98.5 (98.0–98.8)	0.0–99.3

Abbreviations: CI, Confidence interval; CT, *Chlamydia trachomatis*; HIV, Human immunodeficiency virus; NG, *Neisseria gonorrhoeae*; STI, Sexually transmitted infection.

A minimum of three studies was required to perform a meta-analysis.

*Q: The Cochran’s Q statistic is a measure assessing the existence of heterogeneity in pooled outcome measures, here NG prevalence.

† I^2^: A measure that assesses the magnitude of between-study variation that is due to true differences in NG prevalence across studies rather than chance.

‡ Prediction interval: A measure that estimates the distribution (95% interval) of true NG prevalence around the estimated mean.

§ Blood tests for antibodies include haemagglutination assays, complement fixation tests, and measurements of immunoglobulins (e.g., IgG, IgA).

¶ The term “men who have sex with men” is used inclusively and encompasses men who have sex with men, transgender people, and male or transgender sex workers.

|| Adverse pregnancy or birth outcomes were defined to include miscarriage, ectopic pregnancy, stillbirth, preterm delivery, small-for-gestational-age infants, and related complications.

** Other populations include groups with an undetermined risk of acquiring NG infection, such as cervical cancer patients, individuals evaluated following sexual assault, and mixed or undefined populations.

Across general populations, pooled mean prevalence was 3.2% (95% CI: 2.9–3.5) for urogenital infection, 1.9% (95% CI: 0.5–4.2) for anorectal infection, and 0.0% (95% CI: 0.0–0.0) for oropharyngeal infection; however, the number of anorectal and oropharyngeal prevalence estimates was limited.

Among FSWs, prevalence was substantially higher, with pooled estimates of 11.5% (95% CI: 9.9–13.2) for urogenital infection and 2.0% (95% CI: 0.4–4.5) for anorectal infection. Among MSM, the pooled mean prevalence was 2.8% (95% CI: 2.4–3.3) for urogenital infection, 8.3% (95% CI: 5.8–11.0) for anorectal infection, and 5.7% (95% CI: 3.6–8.3) for oropharyngeal infection.

Symptomatic men exhibited particularly high prevalence, with pooled mean estimates of 51.5% (95% CI: 47.5–55.5) for urogenital infection, 16.3% (95% CI: 0.6–45.2) for anorectal infection, and 5.1% (95% CI: 2.2–8.8) for oropharyngeal infection. Among symptomatic women, pooled mean urogenital prevalence was 9.0% (95% CI: 7.7–10.4).

High pooled mean urogenital prevalence was also observed among STI clinic attendees (15.5%; 95% CI: 12.0–19.5), patients with confirmed or suspected STIs and related infections (24.3%; 95% CI: 12.0–39.1), and sexual contacts of persons infected with NG/*Chlamydia trachomatis* (CT) (50.0%; 95% CI: 31.0–69.1).

Pooled mean urogenital prevalence was 8.6% (95% CI: 5.3–12.6) among women with adverse pregnancy or birth outcomes and 3.0% (95% CI: 1.2–5.4) among infertility clinic attendees. Among individuals living with HIV and those in HIV-discordant couples, the pooled mean urogenital prevalence was 3.5% (95% CI: 2.6–4.4).

Serological evidence of ever having had NG infection, where available, indicated substantial lifetime exposure. The pooled mean prevalence of ever-infection was 15.0% (95% CI: 7.6–24.2) among general populations, 37.0% (95% CI: 22.3–53.0) among women with adverse pregnancy or birth outcomes, 41.1% (95% CI: 23.3–60.1) among infertility clinic attendees, 53.3% (95% CI: 49.3–57.2) among FSWs, and 97.0% (95% CI: 91.5–99.4) among patients with confirmed or suspected STIs and related infections.

Additional pooled mean prevalence estimates are presented in Table H in S1 Appendix for subgroups within the general population, including pregnant women, and for subgroups within intermediate-risk populations. Table I in S1 Appendix presents pooled estimates for populations of public health importance, stratified by studies using probability-based versus non-probability-based sampling methods.

Substantial between-study heterogeneity was observed across most meta-analyses, with strong evidence for heterogeneity (*p* < 0.1) and I² values often exceeding 50%, indicating that variability in prevalence estimates largely reflected true differences across studies rather than sampling error (Table 2 and Tables G, H, and I in S1 Appendix; and Figs G, H, and I in S1 Appendix). Most estimates were also accompanied by wide prediction intervals, reflecting this heterogeneity in prevalence measures (Table 2 and Tables G, H, and I in S1 Appendix; and Figs G, H, and I in S1 Appendix). Accordingly, pooled estimates should be interpreted as summary measures of diverse underlying populations rather than precise population-level values. To further investigate the sources of this heterogeneity, meta-regression analyses were conducted.

### Associations with NG prevalence and sources of between-study heterogeneity

Table 3 summarizes the results of the univariable and multivariable meta-regression analyses for urogenital NG infection. Two multivariable models were fitted: one treating calendar time as a categorical variable and the other as a continuous linear variable. The year of publication served as the primary temporal measure because it was available for all studies, whereas the year of data collection required imputation for a subset of observations (*n* = 243; 13.0%). Sensitivity analyses substituting the year of data collection for the year of publication produced consistent results (Table 4). All models explained most of the between-study variation, with adjusted *R*² values of ~60%, and identified coherent epidemiological patterns, including consistent gradients by population type, age, subregion, and study design effects.

**Table 3:** Associations with NG prevalence. Associations with NG prevalence and sources of between-study heterogeneity were examined through univariable and multivariable meta-regression analyses of urogenital NG prevalence in sub-Saharan Africa.

Urogenital specimens			Stratified prevalence measures	Sample size	Univariable analysis				Multivariable analyses			
**Total *n***	**Total *N***	**PR (95% CI)**	***p*-value**	**LT test *p*-value**	**Adjusted *R*** ^**2**^	**Model 1**		**Model 2**	
						**APR (95% CI)**	***p*-value**	**APR (95% CI)**	***p*-value**
**Population characteristics**	**Population type**	General populations	706	321,056	1.00	–	<0.001	51.33	1.00	–	1.00	–
		Intermediate-risk populations	99	38,473	0.80 (0.63–1.02)	0.068			1.18 (0.93–1.49)	0.166	1.05 (0.83–1.32)	0.707
		Female sex workers	200	67,409	3.05 (2.61–3.56)	<0.001			4.09 (3.50–4.78)	<0.001	3.95 (3.38–4.62)	<0.001
		Men who have sex with men^*^	46	11,993	0.90 (0.66–1.24)	0.518			1.08 (0.78–1.49)	0.635	1.07 (0.77–1.47)	0.690
		Symptomatic women	237	54,600	2.59 (2.23–3.02)	<0.001			2.36 (2.03–2.74)	<0.001	2.39 (2.05–2.78)	<0.001
		Symptomatic men	248	57,220	12.46 (10.83–14.34)	<0.001			8.91 (7.37–10.77)	<0.001	9.12 (7.54–11.04)	<0.001
		Symptomatic women and men	48	8,593	4.17 (3.13–5.54)	<0.001			2.86 (1.83–4.48)	<0.001	3.28 (2.09–5.13)	<0.001
		Infertility clinic attendees	20	2,316	1.61 (0.92–2.83)	0.094			1.49 (0.88–2.52)	0.137	1.39 (0.82–2.35)	0.224
		Women with adverse pregnancy or birth outcomes^†^	16	1,838	2.72 (1.64–4.49)	<0.001			1.78 (1.11–2.85)	0.017	1.91 (1.19–3.06)	0.007
		STI clinic attendees	65	26,575	3.79 (2.96–4.85)	<0.001			3.29 (2.60–4.16)	<0.001	3.30 (2.60–4.18)	<0.001
		Individuals living with HIV and individuals in HIV-discordant couples	81	28,154	1.08 (0.84–1.38)	0.543			1.06 (0.84–1.33)	0.645	1.02 (0.81–1.29)	0.854
		Sexual contacts of persons infected with NG/CT	3	133	13.77 (4.84–39.19)	<0.001			7.37 (2.80–19.40)	<0.001	7.15 (2.70–18.93)	<0.001
		Patients with confirmed or suspected STIs and related infections	25	7,619	4.19 (2.84–6.18)	<0.001			3.66 (2.52–5.30)	<0.001	3.52 (2.42–5.12)	<0.001
		Other populations^‡^	75	24,441	2.72 (2.13–3.48)	<0.001			2.08 (1.65–2.64)	<0.001	2.17 (1.71–2.76)	<0.001
	**Age group**	<25 years	372	172,765	1.00	–	<0.001	7.37	1.00	–	1.00	–
		25-34 years	891	315,662	1.28 (1.09–1.51)	0.003			0.84 (0.74–0.94)	0.003	0.84 (0.75–0.94)	0.003
		35-44 years	168	44,424	0.91 (0.69–1.20)	0.509			0.71 (0.59–0.86)	0.001	0.72 (0.59–0.87)	0.001
		≥45 years	39	4,711	1.10 (0.64–1.91)	0.729			0.78 (0.52–1.15)	0.205	0.77 (0.52–1.15)	0.202
		Mixed ages	399	112,858	2.59 (2.14–3.13)	<0.001			0.98 (0.85–1.13)	0.779	0.91 (0.79–1.06)	0.235
	**Sex**	Women	1,236	434,648	1.00	–	<0.001	10.90	1.00	–	1.00	–
		Men	561	176,159	2.42 (2.12–2.77)	<0.001			1.09 (0.94–1.27)	0.243	1.10 (0.95–1.27)	0.217
		Both women and men	72	39,613	1.86 (1.37–2.52)	<0.001			1.40 (0.97–2.00)	0.069	1.34 (0.93–1.92)	0.111
	**African region**	Southern Africa	653	219,247	1.00	–	<0.001	2.13	1.00	–	1.00	–
		Eastern Africa	569	202,162	0.70 (0.60–0.81)	<0.001			0.73 (0.65–0.81)	<0.001	0.74 (0.66–0.82)	<0.001
		Western Africa^§^	514	165,457	0.64 (0.54–0.75)	<0.001			0.58 (0.51–0.65)	<0.001	0.56 (0.50–0.64)	<0.001
		Central Africa	108	27,615	0.91 (0.69–1.20)	0.523			0.60 (0.49–0.73)	<0.001	0.60 (0.49–0.73)	<0.001
		Mixed regions	25	35,939	0.46 (0.27–0.77)	0.004			0.76 (0.54–1.08)	0.125	0.73 (0.51–1.03)	0.075
**Study methodology characteristics**	**Assay type**	NAAT/PCR	913	366,987	1.00	–	<0.001	9.65	1.00	–	1.00	–
		Culture^¶^	733	207,605	1.80 (1.58–2.05)	<0.001			1.03 (0.92–1.16)	0.604	0.98 (0.87–1.11)	0.779
		Gram stain/microscopy^||^	126	40,746	3.81 (2.99–4.85)	<0.001			1.26 (1.04–1.53)	0.019	1.14 (0.93–1.39)	0.208
		Rapid test	13	4,613	0.81 (0.37–1.78)	0.602			0.61 (0.34–1.08)	0.090	0.67 (0.38–1.19)	0.170
		ELISA/EIA	6	1,010	1.95 (0.71–5.36)	0.195			1.28 (0.63–2.60)	0.489	1.27 (0.63–2.59)	0.507
		Mixed	13	3,864	0.77 (0.37–1.60)	0.480			0.37 (0.22–0.63)	<0.001	0.33 (0.20–0.56)	<0.001
		Unclear	65	25,595	1.20 (0.85–1.68)	0.301			0.78 (0.60–1.01)	0.056	0.81 (0.63–1.05)	0.115
	**Sample size**	<200	504	45,326	1.00	–	<0.001	7.30	1.00	–	1.00	–
		≥200	1,365	605,094	0.46 (0.40–0.53)	<0.001			0.66 (0.59–0.73)	<0.001	0.66 (0.59–0.73)	<0.001
	**Sampling method**	Probability-based	396	164,574	1.00	–	<0.001	8.32	1.00	–	1.00	–
		Non-probability-based	1,473	485,846	2.58 (2.21–3.02)	<0.001			1.39 (1.23–1.58)	<0.001	1.40 (1.24–1.58)	<0.001
	**Response rate**	≥80%	347	164,224	1.00	–	<0.001	6.54	1.00	–	1.00	–
		<80%	81	31,812	0.76 (0.54–1.07)	0.121			0.86 (0.67–1.10)	0.233	0.85 (0.67–1.09)	0.212
		Unclear	1,441	454,384	2.05 (1.75–2.41)	<0.001			1.06 (0.94–1.20)	0.338	1.08 (0.96–1.22)	0.224
**Temporal trend**	**Year of publication**	<2005	811	240,392	1.00	–	<0.001	5.93	1.00	–	–	–
		2005-2014	444	193,319	0.44 (0.37–0.51)	<0.001			0.64 (0.56–0.73)	<0.001	–	–
		≥2,015	614	216,709	0.65 (0.56–0.74)	<0.001			0.83 (0.74–0.94)	0.003	–	–
	**Year of publication**		1,869	650,420	0.98 (0.97–0.98)	<0.001	<0.001	5.41	–	–	0.99 (0.98–0.99)	<0.001

Abbreviations: APR, Adjusted prevalence ratio; CI, Confidence interval; CT, *Chlamydia trachomatis*; EIA, Enzyme immunoassay; ELISA, Enzyme-linked immunosorbent assay; HIV, Human immunodeficiency virus; LT test, Likelihood ratio test; NAAT, Nucleic acid amplification test; NG, *Neisseria gonorrhoeae*; PR, Prevalence ratio; PCR, Polymerase chain reaction; STI, Sexually transmitted infection.

Adjusted R^2^ for the final multivariable model 1 = 60.29%.

Adjusted R^2^ for the final multivariable model 2 = 59.86%.

*The term “men who have sex with men” is used inclusively and encompasses men who have sex with men, transgender people, and male or transgender sex workers.

† Adverse pregnancy or birth outcomes were defined to include miscarriage, ectopic pregnancy, stillbirth, preterm delivery, small-for-gestational-age infants, and related complications.

‡ Other populations include groups with an undetermined risk of acquiring NG infection, such as cervical cancer patients, individuals evaluated following sexual assault, and mixed or undefined populations.

§ The Northern Africa subregion within sub-Saharan Africa comprises only one country, Mauritania, which contributed two prevalence measures. Given the limited data, this subregion was combined with Western Africa, the geographically closest region, for the purposes of the meta-regression analyses.

¶ Culture refers to microbiological culture results, with or without accompanying Gram stain analysis.

|| Gram stain/microscopy refers to diagnostic methods based on Gram stain alone, microscopy alone, Gram stain or microscopy confirmed by culture and/or PCR, or Gram stain or microscopy performed with or without culture.

**Table 4:** Sensitivity analysis. Associations with NG prevalence and sources of between-study heterogeneity were examined through univariable and multivariable meta-regression analyses of urogenital NG prevalence in sub-Saharan Africa, using year of data collection in place of year of publication as the temporal variable.

Urogenital specimens			Stratified prevalence measures	Sample size	Univariable analysis					Multivariable analyses			
**Total *n***	**Total *N***	**PR (95% CI)**		***p*-value**	**LT test** ***p*-value**	**Adjusted *R***	**Model 1**		**Model 2**	
							**APR (95% CI)**	***p*-value**	**APR (95% CI)**	***p*-value**
**Population characteristics**	**Population type**	General populations	706	321,056	1.00		–	<0.001	51.33	1.00	–	1.00	–
		Intermediate-risk populations	99	38,473	0.80 (0.63–1.02)		0.068			1.11 (0.88–1.41)	0.359	1.05 (0.83–1.32)	0.698
		Female sex workers	200	67,409	3.05 (2.61–3.56)		<0.001			4.02 (3.44–4.69)	<0.001	3.94 (3.37–4.60)	<0.001
		Men who have sex with men^*^	46	11,993	0.90 (0.66–1.24)		0.518			1.02 (0.74–1.41)	0.892	1.07 (0.78–1.48)	0.669
		Symptomatic women	237	54,600	2.59 (2.23–3.02)		<0.001			2.38 (2.05–2.77)	<0.001	2.39 (2.06–2.78)	<0.001
		Symptomatic men	248	57,220	12.46 (10.83–14.34)		<0.001			8.81 (7.29–10.65)	<0.001	9.14 (7.55–11.05)	<0.001
		Symptomatic women and men	48	8,593	4.17 (3.13–5.54)		<0.001			2.84 (1.82–4.44)	<0.001	3.28 (2.09–5.13)	<0.001
		Infertility clinic attendees	20	2,316	1.61 (0.92–2.83)		0.094			1.47 (0.87–2.48)	0.150	1.39 (0.82–2.35)	0.225
		Women with adverse pregnancy or birth outcomes^†^	16	1,838	2.72 (1.64–4.49)		<0.001			1.80 (1.13–2.89)	0.014	1.90 (1.19–3.05)	0.007
		STI clinic attendees	65	26,575	3.79 (2.96–4.85)		<0.001			3.33 (2.63–4.21)	<0.001	3.30 (2.61–4.18)	<0.001
		Individuals living with HIV and individuals in HIV-discordant couples	81	28,154	1.08 (0.84–1.38)		0.543			1.05 (0.84–1.32)	0.668	1.02 (0.81–1.29)	0.848
		Sexual contacts of persons infected with NG/CT	3	133	13.77 (4.84–39.19)		<0.001			7.32 (2.79–19.22)	<0.001	7.11 (2.69–18.80)	<0.001
		Patients with confirmed or suspected STIs and related infections	25	7,619	4.19 (2.84–6.18)		<0.001			3.55 (2.45–5.15)	<0.001	3.51 (2.42–5.11)	<0.001
		Other populations^‡^	75	24,441	2.72 (2.13–3.48)		<0.001			2.09 (1.65–2.65)	<0.001	2.18 (1.72–2.77)	<0.001
	**Age group**	<25 years	372	172,765	1.00		–	<0.001	7.37	1.00	–	1.00	–
		25-34 years	891	315,662	1.28 (1.09–1.51)		0.003			0.84 (0.75–0.95)	0.005	0.84 (0.75–0.94)	0.003
		35-44 years	168	44,424	0.91 (0.69–1.20)		0.509			0.73 (0.60–0.88)	0.001	0.72 (0.59–0.87)	0.001
		≥45 years	39	4,711	1.10 (0.64–1.91)		0.729			0.79 (0.53–1.17)	0.234	0.77 (0.52–1.15)	0.203
		Mixed ages	399	112,858	2.59 (2.14–3.13)		<0.001			0.97 (0.84–1.12)	0.675	0.91 (0.78–1.06)	0.217
	**Sex**	Women	1,236	434,648	1.00		–	<0.001	10.90	1.00	–	1.00	–
		Men	561	176,159	2.42 (2.12–2.77)		<0.001			1.10 (0.95–1.28)	0.190	1.10 (0.95–1.27)	0.220
		Both women and men	72	39,613	1.86 (1.37–2.52)		<0.001			1.41 (0.98–2.01)	0.062	1.34 (0.94–1.92)	0.109
	**African region**	Southern Africa	653	219,247	1.00		–	<0.001	2.13	1.00	–	1.00	–
		Eastern Africa	569	202,162	0.70 (0.60–0.81)		<0.001			0.73 (0.65–0.81)	<0.001	0.74 (0.66–0.82)	<0.001
		Western Africa^§^	514	165,457	0.64 (0.54–0.75)		<0.001			0.57 (0.50–0.64)	<0.001	0.56 (0.50–0.63)	<0.001
		Central Africa	108	27,615	0.91 (0.69–1.20)		0.523			0.59 (0.48–0.71)	<0.001	0.60 (0.49–0.73)	<0.001
		Mixed regions	25	35,939	0.46 (0.27–0.77)		0.004			0.76 (0.53–1.07)	0.115	0.73 (0.51–1.03)	0.074
**Study methodology characteristics**	**Assay type**	NAAT/PCR	913	366,987	1.00		–	<0.001	9.65	1.00	–	1.00	–
		Culture^¶^	733	207,605	1.80 (1.58–2.05)		<0.001			1.00 (0.89–1.13)	0.969	0.98 (0.86–1.11)	0.713
		Gram stain/microscopy^||^	126	40,746	3.81 (2.99–4.85)		<0.001			1.25 (1.03–1.52)	0.024	1.13 (0.92–1.38)	0.235
		Rapid test	13	4,613	0.81 (0.37–1.78)		0.602			0.64 (0.36–1.12)	0.119	0.67 (0.38–1.20)	0.178
		ELISA/EIA	6	1,010	1.95 (0.71–5.36)		0.195			1.23 (0.61–2.50)	0.560	1.26 (0.62–2.57)	0.517
		Mixed	13	3,864	0.77 (0.37–1.60)		0.480			0.40 (0.24–0.69)	0.001	0.33 (0.20–0.56)	<0.001
		Unclear	65	25,595	1.20 (0.85–1.68)		0.301			0.78 (0.61–1.01)	0.061	0.82 (0.63–1.05)	0.116
	**Sample size**	<200	504	45,326	1.00		–	<0.001	7.30	1.00	–	1.00	–
		≥200	1,365	605,094	0.46 (0.40–0.53)		<0.001			0.66 (0.59–0.73)	<0.001	0.66 (0.59–0.73)	<0.001
	**Sampling method**	Probability-based	396	164,574	1.00		–	<0.001	8.32	1.00	–	1.00	–
		Non-probability-based	1,473	485,846	2.58 (2.21–3.02)		<0.001			1.40 (1.24–1.58)	<0.001	1.40 (1.24–1.59)	<0.001
	**Response rate**	≥80%	347	164,224	1.00		–	<0.001	6.54	1.00	–	1.00	–
		<80%	81	31,812	0.76 (0.54–1.07)		0.121			0.86 (0.67–1.10)	0.218	0.86 (0.67–1.10)	0.219
		Unclear	1,441	454,384	2.05 (1.75–2.41)		<0.001			1.06 (0.94–1.19)	0.363	1.08 (0.96–1.22)	0.210
**Temporal trend**	**Year of data collection**	<2000	776	226,684	1.00		–	<0.001	5.93	1.00	–	–	–
		2000-2009	416	190,822	0.44 (0.37–0.51)		<0.001			0.62 (0.54–0.70)	<0.001	–	–
		≥2,010	677	232,914	0.65 (0.56–0.74)		<0.001			0.77 (0.69–0.87)	<0.001	–	–
	**Year of data collection**		1,869	650,420		0.98 (0.97–0.98)	<0.001	<0.001	5.47	–	–	0.99 (0.98–0.99)	<0.001

Abbreviations: APR, Adjusted prevalence ratio; CI, Confidence interval; CT, *Chlamydia trachomatis*; EIA, Enzyme immunoassay; ELISA, Enzyme-linked immunosorbent assay; HIV, Human immunodeficiency virus; LT test, Likelihood ratio test; NAAT, Nucleic acid amplification test; NG, *Neisseria gonorrhoeae*; PR, Prevalence ratio; PCR, Polymerase chain reaction; STI, Sexually transmitted infection.

Adjusted R^2^ for the final multivariable model 1 = 60.48%.

Adjusted R^2^ for the final multivariable model 2 = 59.94%.

*The term “men who have sex with men” is used inclusively and encompasses men who have sex with men, transgender people, and male or transgender sex workers.

† Adverse pregnancy or birth outcomes were defined to include miscarriage, ectopic pregnancy, stillbirth, preterm delivery, small-for-gestational-age infants, and related complications.

‡ Other populations include groups with an undetermined risk of acquiring NG infection, such as cervical cancer patients, individuals evaluated following sexual assault, and mixed or undefined populations.

§ The Northern Africa subregion within sub-Saharan Africa comprises only one country, Mauritania, which contributed two prevalence measures. Given the limited data, this subregion was combined with Western Africa, the geographically closest region, for the purposes of the meta-regression analyses.

¶ Culture refers to microbiological culture results, with or without accompanying Gram stain analysis.

|| Gram stain/microscopy refers to diagnostic methods based on Gram stain alone, microscopy alone, Gram stain or microscopy confirmed by culture and/or PCR, or Gram stain or microscopy performed with or without culture.

Population type was the dominant predictor of NG prevalence, independently accounting for 51.3% of the observed variation (Table 3). Relative to the general population, prevalence was highest among symptomatic men, followed by sexual contacts of persons infected with NG/CT, FSWs, patients with confirmed or suspected STIs and related infections, STI clinic attendees, symptomatic women, and women with adverse pregnancy or birth outcomes. There was no evidence of a difference in prevalence between individuals living with HIV and those in HIV-discordant couples and the general population.

No differences in prevalence were observed by sex. Prevalence was highest among individuals younger than 25 years and declined with increasing age. Subregional patterns were also observed, with the highest prevalence in Southern Africa, followed by Eastern Africa, Central Africa, and Western Africa.

All models indicated a consistent decline in NG prevalence over time, regardless of how calendar time was modeled or whether the temporal variable was based on year of publication or year of data collection. The estimated relative rate of decline was 1% per year, corresponding to a gradual but sustained reduction in prevalence over time across populations and settings.

Study design characteristics influenced prevalence estimates. A marked small-study effect was identified, with studies enrolling ≥200 participants reporting ~34% lower prevalence than those with smaller sample sizes. Studies using non-probability sampling reported higher prevalence. In contrast, there were no notable differences in prevalence by assay type or response rate.

Due to insufficient numbers of studies, meta-regression analyses were not undertaken for anorectal or oropharyngeal prevalence.

## Discussion

This study provided a comprehensive characterization of NG epidemiology in sub-Saharan Africa over several decades. Although pooled estimates suggested a urogenital prevalence of ~3% in the general population, substantial between-study heterogeneity indicated that this value should be interpreted as a summary measure rather than a precise population-level estimate.

Greater insight was provided by the distribution of estimates and consistent gradients across population types, age groups, and subregions. These patterns, supported by meta-regression analyses that explained a substantial proportion of variability, reaffirmed that sub-Saharan Africa continues to carry the highest burden of NG infection compared with other regions [5,6,14,16,22–25]. Notably, the higher prevalence in this region reflects, in part, its young population age structure [43], as the study identified higher prevalence among individuals younger than 25 years of age.

Despite the persistently high burden, NG prevalence demonstrated a gradual decline over the past several decades, with an estimated relative reduction of 1% per year across populations and settings, consistent with declines observed in other regions [22–25]. However, this pace of decline remains far below what is needed to achieve WHO target of a 90% reduction in NG incidence by 2030 [11]. Several factors may have contributed to the long-term downward trend, including safer sexual behaviors in the post-HIV era [44,45], increased STI awareness [46], expanded access to HIV/STI services [47], and broader socio-economic changes that have shaped sexual network structures [48].

However, these overall long-term declines may mask shorter-term fluctuations or recent increases in prevalence. Evidence from other regions has documented rising STI incidence in recent years, in part driven by changes in sexual risk behavior following the scale-up of HIV antiretroviral therapy and HIV pre-exposure prophylaxis [7,8,49]. Similar dynamics may be emerging in parts of sub-Saharan Africa, underscoring the need for sustained surveillance and timely data to capture evolving epidemiologic patterns.

The prevalence of urogenital NG infection was elevated among FSWs at 11.5%, while anorectal and oropharyngeal infections among MSM reached 8.3% and 5.7%, respectively. These patterns echo findings from other regions [22–25] and highlight the important contribution of commercial sex networks and other key population sexual networks to sustaining NG transmission. The results are consistent with well-established associations between NG infection and recent high-risk sexual behaviors [50–52], including frequent partner change and engagement in transactional sex [52–56]. The elevated prevalence of oropharyngeal infection is particularly concerning given its recognized role as a reservoir for the development and spread of gonococcal AMR [1,57,58]. Taken together, these findings underscore the importance of strengthening targeted prevention, screening, and treatment strategies among key populations to reduce transmission and mitigate the growing threat of AMR.

As expected, NG prevalence was extremely high among symptomatic men, reflecting the typically symptomatic presentation of infection in men [59] and underscoring its role as a major cause of urethritis in the region, consistent with findings from other settings [22–25]. Higher prevalence among women experiencing adverse pregnancy or birth outcomes suggests a possible contribution of NG infection to these outcomes, in line with evidence from other regions [3,23]. The substantial prevalence observed among sexual contacts of persons infected with NG/CT further highlights the importance of partner notification and expedited partner therapy, both core components of WHO Global STI Strategy [11].

A clear hierarchical pattern in NG prevalence was observed, with higher prevalence in higher-risk populations. This pattern explained most of the variability in prevalence across studies and reflects trends reported in other regions [22–25] and for other STIs [27–29,34,60,61]. Unlike findings from some regions [22–24], however, no differences in prevalence by sex were identified. Prevalence was highest among individuals younger than 25 years and declined with increasing age, a pattern consistent with the age distribution of STI incidence in sub-Saharan Africa [15,62,63]. Subregional differences in prevalence were observed, warranting further investigation to clarify underlying drivers.

Serologically measured ever-infection prevalence was high across all populations with available data, indicating substantial cumulative exposure to NG in the region. However, interpretation of these estimates remains constrained by persistent uncertainties regarding the accuracy and reliability of NG serological assays [64], which limit their epidemiologic value. A pronounced small-study effect [29] was evident, with studies enrolling ≥200 participants reporting lower prevalence than those with smaller sample sizes—a well-documented pattern in STI prevalence research across pathogens [22–24,27–29,34].

These findings have implications for public health policy and programmatic responses. The higher prevalence observed among key populations and individuals younger than 25 years highlights these groups as priorities for targeted prevention, testing, and early diagnosis, particularly within vulnerable populations. This is consistent with recent WHO guidance on the management of asymptomatic NG infection, which recommends targeted screening in higher prevalence settings—especially among adolescents and young people, pregnant women, and key populations—and supports the use of context-informed prevalence thresholds or prevalence-informed decision-making [65]. In such settings, expanding access to screening and strengthening early case detection may be critical to interrupt transmission [65].

Strengthening partner notification mechanisms remains essential to reduce onward transmission, particularly in high prevalence settings and key populations [66]. Furthermore, the substantial burden of NG infection, together with the growing threat of AMR, underscores the need to strengthen gonococcal AMR surveillance systems [1,9]. Integrating epidemiological surveillance with AMR monitoring, alongside timely updates to treatment guidelines, will be essential to sustain effective control efforts and maximize programmatic impact [1,9].

This study has limitations. Data availability varied across countries, anatomical sites, and population groups. Although urogenital NG infection was extensively documented, far fewer studies reported anorectal or oropharyngeal infections. Nevertheless, data were available from 40 countries in sub-Saharan Africa, representing 99% of the region’s population [43], providing broad geographic coverage despite these gaps. The analyses were based on data available up to June 4, 2025, and more recent changes in trends would not have been captured.

A high degree of between-study heterogeneity was observed in most meta-analyses, reflecting genuine epidemiological diversity across populations and settings. Accordingly, pooled estimates should be interpreted with caution, and greater emphasis should be placed on the gradients and patterns identified through the meta-regression analyses, which explained most of this heterogeneity.

Substantial variability across studies was evident in diagnostic assays, sample sizes, sampling strategies, and response rates. Diagnostic methods evolved over time, and most studies relied on convenience rather than probability-based sampling. Studies using non-probability sampling reported higher prevalence estimates, and a clear small-study effect was observed. In contrast, differences in assay type and response rate did not appear to meaningfully influence prevalence estimates. Evidence of publication bias was detected in some meta-analyses and appeared to be driven by the small-study effect. Overall, these patterns indicate that studies with weaker methodological rigor tend to overestimate NG prevalence, whereas more robustly designed studies produce lower and likely more reliable estimates.

Prevalence estimates derived from facility-based populations, such as symptomatic populations, exhibited substantial variability and often skewed distributions. This heterogeneity likely reflects spectrum effects arising from differences in demographic and behavioral risk profiles, symptom severity, and healthcare-seeking behavior, as well as variation in the catchment populations, referral patterns, and clinical scope of participating facilities. Moreover, differences in access to care, diagnostic practices, and case definitions across facilities may further contribute to between-study variability.

While an assessment of study quality was conducted and the framework distinguished between-study precision and risk of bias, it has inherent limitations. In particular, it does not fully capture issues of external validity, including the fundamental non-representativeness of clinic-based and convenience samples, and elements of reporting quality and bias are partially conflated. Moreover, statistical precision, as reflected by sample size, does not equate to representativeness, and large studies based on non-probability sampling may still yield biased estimates. Accordingly, many of the included studies cannot be interpreted as population prevalence estimates, irrespective of their statistical precision or risk of bias assessments, and should instead be considered as reflecting prevalence within specific study populations.

The precision assessment used a dichotomized sample size threshold of 200 as a pragmatic proxy for statistical precision; however, statistical precision is inherently a continuous construct, more appropriately reflected by study-specific CIs, and sample size alone does not fully capture precision, particularly in structured survey designs where clustering and weighting influence variance. This approach was adopted to facilitate comparability across studies and to assess small-study effects, a recognized source of bias in STI research [22–24,27–29,34], but it represents a simplification and does not account for complex sampling designs.

Publication bias was assessed using Doi plots and the LFK index [36], which assumes symmetry of study estimates in the absence of bias; however, in the presence of substantial between-study heterogeneity, observed asymmetry may reflect true epidemiological variation rather than publication bias.

Although the meta-regression analyses suggested a decline in NG prevalence of ~1% per year, this finding should be interpreted with caution. Temporal trends in observed prevalence may reflect not only changes in underlying epidemiology but also shifts in diagnostic technologies, healthcare access and utilization, and the composition of study populations over time, including the evolving inclusion of higher- or lower-risk populations. Accordingly, causal inference regarding temporal trends cannot be established with certainty based on these data.

This study has strengths. First, it provides a detailed characterization of NG epidemiology in sub-Saharan Africa, drawing on an extensive, systematically assembled database of 1,604 overall prevalence measures and 2,153 stratified measures from nearly 3 million individuals. The data span more than three decades, cover nearly all countries in the region, and include all subregions. This represents the largest NG prevalence database compiled for any region globally [22–24], matched only by that available for Europe [22]. Second, the breadth and scope of the dataset enabled a wide range of analyses and supported an in-depth examination of heterogeneity. The meta-regression analyses explained most of the observed variation in prevalence through epidemiological and methodological factors, offering a nuanced and robust understanding of associations with NG prevalence across the region.

Third, although the included studies varied in methodological rigor, overall quality was higher than that documented in comparable systematic reviews from other regions [22–24]. This is notable given the resource-constrained settings in which many studies were conducted. The relatively strong quality profile reflects substantial international investments in HIV/STI research in the region following recognition of the large HIV epidemic [67]. While many studies did not meet stricter criteria related to sampling method or response rate, the vast majority fulfilled the other quality domains required for prevalence studies based on standard assessment tools in the literature (Table C in S1 Appendix) [32,33,68,69]. Lastly, the study employed a comprehensive search strategy across multiple databases, without restrictions on language or publication year, and incorporated rigorous assessments of study quality, potential biases, and their influence on reported prevalence, enhancing the validity and interpretability of the findings.

To conclude, this systematic review provides a synthesis of NG epidemiology in sub-Saharan Africa, demonstrating that prevalence remains substantially higher than in other world regions and underscoring the region’s disproportionate burden. Although an apparent long-term decline in prevalence was observed, the pace of reduction remains insufficient to meet WHO 2030 target of a 90% decrease in incidence. The study findings provide a foundation to inform the design and expansion of STI and sexual health programs, strengthen gonococcal AMR surveillance, and identify priority populations for future NG vaccination strategies. Collectively, they highlight the need for sustained investment in prevention, diagnostic capacity, and treatment services to curb transmission and mitigate the growing threat of AMR.

## Supporting information

S1 Appendix Supplementary information. **Table A.** PRISMA checklist. Preferred Reporting Items for Systematic Reviews and Meta-Analyses (PRISMA) checklist. Page MJ, McKenzie JE, Bossuyt PM, Boutron I, Hoffmann TC, Mulrow CD, and colleagues. The PRISMA 2020 statement: an updated guideline for reporting systematic reviews. *BMJ*. 2021;372:n71. Epub 2021/03/31. https://doi.org/10.1136/bmj.n71. PubMed PMID: 33782057; PubMed Central PMCID: PMCPMC8005924. The PRISMA 2020 checklist is licensed under the Creative Commons Attribution 4.0 International License. **Table B.** Search strategy. Database sources and systematic search strategies were used to identify NG prevalence studies in sub-Saharan Africa. **Box A.** Countries included in sub-Saharan Africa. Countries included and their subregional classification in the study definition of sub-Saharan Africa. **Box B.** Data extraction variables. Summary of variables extracted from eligible studies. **Table C.** Quality assessment. Range of quality assessment components relevant to prevalence studies and their applicability to this systematic review and the included studies reporting NG prevalence. **Box C.** Analysis variables. Factors (variables) were predefined a priori and included in both univariable and multivariable meta-regression analyses. **Table D.** Included publications. List of publications included in this systematic review, from which NG prevalence data were extracted. **Fig A.** Geographic distribution of prevalence data. Country-level distribution of NG prevalence measures across sub-Saharan Africa, showing the spatial distribution and relative contribution of studies by country. **Fig B.** Temporal trends in NG prevalence. NG prevalence estimates across all population groups over time by A) year of data collection and B) publication year. Points represent individual prevalence estimates, and lines indicate fitted linear trends. **Fig C.** Temporal trends in NG prevalence across subregions. NG prevalence estimates across all population groups over time by year of data collection for A) Central Africa, B) Eastern Africa, C) Southern Africa, and D) Western Africa. Points represent individual prevalence estimates, and lines indicate fitted linear trends. **Table E.** Summary of study quality assessment. Summary of precision and risk of bias assessments for studies reporting NG prevalence in sub-Saharan Africa. **Table F.** Publication bias assessment. Assessment of publication bias in studies reporting NG prevalence in sub-Saharan Africa using Doi plots and the LFK index. **Fig D.** Publication bias plots for urogenital infection. Doi plots assessing publication bias among studies reporting urogenital NG prevalence in sub-Saharan Africa. **Fig E.** Publication bias plots for anorectal infection. Doi plots assessing publication bias among studies reporting anorectal NG prevalence in sub-Saharan Africa. **Fig F.** Publication bias plots for oropharyngeal infection. Doi plots assessing publication bias among studies reporting oropharyngeal NG prevalence in sub-Saharan Africa. **Table G.** NG prevalence estimates by assay type. Pooled mean prevalence of NG infection in sub-Saharan Africa, stratified by population type, anatomical site, and assay type. **Fig G.** Forest plots for urogenital infection. Forest plots presenting outcomes of the pooled mean NG prevalence in urogenital specimens among different populations in sub-Saharan Africa. **Fig H.** Forest plots for anorectal infection. Forest plots presenting outcomes of the pooled mean NG prevalence in anorectal specimens among different populations in sub-Saharan Africa. **Fig I.** Forest plots for oropharyngeal infection. Forest plots presenting outcomes of the pooled mean NG prevalence in oropharyngeal specimens among different populations in sub-Saharan Africa. **Table H.** NG prevalence estimates for select populations. Pooled mean prevalence of NG infection in sub-Saharan Africa for select populations, stratified by anatomical site. **Table I.** NG prevalence estimates by sampling method. Pooled mean prevalence of NG infection in sub-Saharan Africa for populations of public health importance, stratified by studies using A) probability-based sampling and B) non-probability-based sampling.
(DOCX)

## Abbreviations

AMR: antimicrobial resistance
APR: adjusted prevalence ratio
CI: confidence interval
CT: Chlamydia trachomatis
EIA: enzyme immunoassay
FSWs: female sex workers
HIV: human immunodeficiency virus
IBBS: integrated biobehavioral surveys
LFK: Luis Furuya-Kanamori
LT test: Likelihood ratio test
MSM: men who have sex with men
NAAT: nucleic acid amplification tests
NG: Neisseria gonorrhoeae
PCR: polymerase chain reaction
PRs: prevalence ratios
PRISMA: Preferred Reporting Items for Systematic Reviews and Meta-Analyses
PROSPERO: International Prospective Register of Systematic Reviews
STIs: sexually transmitted infections
UNAIDS: Joint United Nations Programme on HIV/AIDS
WHO: World Health Organization.
