# Supplementary material for: Prevalence and epidemiological patterns of Neisseria gonorrhoeae infection in sub-Saharan Africa, 1964–2025: Systematic review, meta-analyses, and meta-regressions
Source: PLoS Med. 2026 Jun 23;23(6):e1004936. doi: 10.1371/journal.pmed.1004936 (PMC13289870; doi:10.1371/journal.pmed.1004936)
Supplement: S1 Appendix — Table A. PRISMA checklist. Preferred Reporting Items for Systematic Reviews and Meta-Analyses (PRISMA) checklist. Page MJ, McKenzie JE, Bossuyt PM, Boutron I, Hoffmann TC, Mulrow CD, and colleagues. The PRISMA 2020 statement: an updated guideline for reporting systematic reviews. BMJ. 2021;372:n71. Epub 2021/03/31. https://doi.org/10.1136/bmj.n71. PubMed PMID: 33782057; PubMed Central PMCID: PMCPMC8005924. The PRISMA 2020 checklist is licensed under the Creative Commons Attribution 4.0 International License. Table B. Search strategy. Database sources and systematic search strategies were used to identify NG prevalence studies in sub-Saharan Africa. Box A. Countries included in sub-Saharan Africa. Countries included and their subregional classification in the study definition of sub-Saharan Africa. Box B. Data extraction variables. Summary of variables extracted from eligible studies. Table C. Quality assessment. Range of quality assessment components relevant to prevalence studies and their applicability to this systematic review and the included studies reporting NG prevalence. Box C. Analysis variables. Factors (variables) were predefined a priori and included in both univariable and multivariable meta-regression analyses. Table D. Included publications. List of publications included in this systematic review, from which NG prevalence data were extracted. Fig A. Geographic distribution of prevalence data. Country-level distribution of NG prevalence measures across sub-Saharan Africa, showing the spatial distribution and relative contribution of studies by country. Fig B. Temporal trends in NG prevalence. NG prevalence estimates across all population groups over time by A) year of data collection and B) publication year. Points represent individual prevalence estimates, and lines indicate fitted linear trends. Fig C. Temporal trends in NG prevalence across subregions. NG prevalence estimates across all population groups over time by year of data collection for A) C [file pmed.1004936.s001.docx]

**S1 Appendix. Supplementary information.**

Table of Contents

[Table A. PRISMA checklist. Preferred Reporting Items for Systematic Reviews and Meta-Analyses (PRISMA) checklist. Page MJ, McKenzie JE, Bossuyt PM, Boutron I, Hoffmann TC, Mulrow CD, et al. The PRISMA 2020 statement: an updated guideline for reporting systematic reviews. BMJ. 2021;372:n71. Epub 2021/03/31. doi: 10.1136/bmj.n71. PubMed PMID: 33782057; PubMed Central PMCID: PMCPMC8005924. The PRISMA 2020 checklist is licensed under the Creative Commons Attribution 4.0 International License. 4](#_Toc230693851)

[Table B. Search strategy. Database sources and systematic search strategies used to identify NG prevalence studies in sub-Saharan Africa. 7](#_Toc230693852)

[Box A. Countries included in sub-Saharan Africa. Countries included and their subregional classification in the study definition of sub-Saharan Africa. 9](#_Toc230693853)

[Box B. Data extraction variables. Summary of variables extracted from eligible studies. 10](#_Toc230693854)

[Table C. Quality assessment. Range of quality assessment components relevant to prevalence studies and their applicability to this systematic review and the included studies reporting NG prevalence. 11](#_Toc230693855)

[Box C. Analysis variables. Factors (variables) predefined *a priori* and included in both univariable and multivariable meta-regression analyses. 13](#_Toc230693856)

[Table D. Included publications. List of publications included in this systematic review, from which NG prevalence data were extracted. 14](#_Toc230693857)

[Fig A. Geographic distribution of prevalence data. Country-level distribution of NG prevalence measures across sub-Saharan Africa, showing the spatial distribution and relative contribution of studies by country. 47](#_Toc230693858)

[Fig B. Temporal trends in NG prevalence. NG prevalence estimates across all population groups over time by A) year of data collection and B) publication year. Points represent individual prevalence estimates, and lines indicate fitted linear trends. 48](#_Toc230693859)

[Fig C. Temporal trends in NG prevalence across subregions. NG prevalence estimates across all population groups over time by year of data collection for A) Central Africa, B) Eastern Africa, C) Southern Africa, and D) Western Africa. Points represent individual prevalence estimates, and lines indicate fitted linear trends. 49](#_Toc230693860)

[Table E. Summary of study quality assessment. Summary of precision and risk of bias assessments for studies reporting NG prevalence in sub-Saharan Africa. 50](#_Toc230693861)

[Table F. Publication bias assessment. Assessment of publication bias in studies reporting NG prevalence in sub-Saharan Africa using Doi plots and the LFK index [4]. 51](#_Toc230693862)

[Fig D. Publication bias plots for urogenital infection. Doi plots assessing publication bias among studies reporting urogenital NG prevalence in sub-Saharan Africa. 52](#_Toc230693863)

[Fig E. Publication bias plots for anorectal infection. Doi plots assessing publication bias among studies reporting anorectal NG prevalence in sub-Saharan Africa. 54](#_Toc230693864)

[Fig F. Publication bias plots for oropharyngeal infection. Doi plots assessing publication bias among studies reporting oropharyngeal NG prevalence in sub-Saharan Africa. 55](#_Toc230693865)

[Table G. NG prevalence estimates by assay type. Pooled mean prevalence of NG infection in sub-Saharan Africa, stratified by population type, anatomical site, and assay type. 56](#_Toc230693866)

[Fig G. Forest plots for urogenital infection. Forest plots presenting outcomes of the pooled mean NG prevalence in urogenital specimens among different populations in sub-Saharan Africa. 60](#_Toc230693867)

[Fig H. Forest plots for anorectal infection. Forest plots presenting outcomes of the pooled mean NG prevalence in anorectal specimens among different populations in sub-Saharan Africa. 96](#_Toc230693868)

[Fig I. Forest plots for oropharyngeal infection. Forest plots presenting outcomes of the pooled mean NG prevalence in oropharyngeal specimens among different populations in sub-Saharan Africa. 100](#_Toc230693869)

[Table H. NG prevalence estimates for select populations. Pooled mean prevalence of NG infection in sub-Saharan Africa for select populations, stratified by anatomical site. 103](#_Toc230693870)

[Table I. NG prevalence estimates by sampling method. Pooled mean prevalence of NG infection in sub-Saharan Africa for populations of public health importance, stratified by studies using A) probability-based sampling and B) non-probability-based sampling. 104](#_Toc230693871)

[References 106](#_Toc230693872)

# **Table A.** PRISMA checklist. Preferred Reporting Items for Systematic Reviews and Meta-Analyses (PRISMA) checklist. Page MJ, McKenzie JE, Bossuyt PM, Boutron I, Hoffmann TC, Mulrow CD, et al. The PRISMA 2020 statement: an updated guideline for reporting systematic reviews. BMJ. 2021;372:n71. Epub 2021/03/31. doi: 10.1136/bmj.n71. PubMed PMID: 33782057; PubMed Central PMCID: PMCPMC8005924. The PRISMA 2020 checklist is licensed under the Creative Commons Attribution 4.0 International License.

| **Section and topic** | **Item #** | **Checklist item** | **Location where item is reported** |
| --- | --- | --- | --- |
| **Title** | | |  |
| Title | 1 | Identify the report as a systematic review. | Title. |
| **Abstract** | | |  |
| Abstract | 2 | See the PRISMA 2020 for Abstracts checklist (table 2). | Abstract section. |
| **Introduction** | | |  |
| Rationale | 3 | Describe the rationale for the review in the context of existing knowledge. | Introduction section: Paragraphs 2 and 3. |
| Objectives | 4 | Provide an explicit statement of the objective(s) or question(s) the review addresses. | Introduction section: Paragraph 4. |
| **Methods** | | |  |
| Eligibility criteria | 5 | Specify the inclusion and exclusion criteria for the review and how studies were grouped for the syntheses. | Methods: Study selection and eligibility criteria section (paragraphs 2-4). |
| Information sources | 6 | Specify all databases, registers, websites, organisations, reference lists and other sources searched or consulted to identify studies. Specify the date when each source was last searched or consulted. | Methods: Data sources and search strategy section (paragraphs 3, 4, and 7); S2 Table. |
| Search strategy | 7 | Present the full search strategies for all databases, registers and websites, including any filters and limits used. | S2 Table. |
| Selection process | 8 | Specify the methods used to decide whether a study met the inclusion criteria of the review, including how many reviewers screened each record and each report retrieved, whether they worked independently, and if applicable, details of automation tools used in the process. | Methods: Study selection and eligibility criteria section (paragraph 1). |
| Data collection process | 9 | Specify the methods used to collect data from reports, including how many reviewers collected data from each report, whether they worked independently, any processes for obtaining or confirming data from study investigators, and if applicable, details of automation tools used in the process. | Methods: Data extraction section (paragraph 1). |
| Data items | 10a | List and define all outcomes for which data were sought. Specify whether all results that were compatible with each outcome domain in each study were sought (e.g., for all measures, time points, analyses), and if not, the methods used to decide which results to collect. | Methods: Data extraction section (paragraphs 2-5); S2 and S3 Boxes, and Table 1. |
|  | 10b | List and define all other variables for which data were sought (e.g., participant and intervention characteristics, funding sources). Describe any assumptions made about any missing or unclear information. | S1, S2, and S3 Boxes, and Table 1; Funding section. |
| Study risk of bias assessment | 11 | Specify the methods used to assess risk of bias in the included studies, including details of the tool(s) used, how many reviewers assessed each study and whether they worked independently, and if applicable, details of automation tools used in the process. | Methods: Precision, risk of bias, and publication bias assessments section (paragraphs 1-4); S3 Table. |
| Effect measures | 12 | Specify for each outcome the effect measure(s) (e.g. risk ratio, mean difference) used in the synthesis or presentation of results. | Methods: Meta-analyses section (paragraphs 1-4); S2 and S3 Boxes. |
| Synthesis methods | 13a | Describe the processes used to decide which studies were eligible for each synthesis (e.g. tabulating the study intervention characteristics and comparing against the planned groups for each synthesis (item #5)). | Methods: Meta-analyses (paragraphs 1-4) and Meta-regression analyses (paragraphs 1-4) sections; S2 andS3 Boxes, and Table 1. |
|  | 13b | Describe any methods required to prepare the data for presentation or synthesis, such as handling of missing summary statistics, or data conversions. | Methods: Meta-analyses (paragraphs 1-4) and Meta-regression analyses (paragraphs 1-4) sections; Boxes S2 and S3 Boxes, and Table 1. |
|  | 13c | Describe any methods used to tabulate or visually display results of individual studies and syntheses. | Methods: Meta-analyses (paragraphs 1-4) and Meta-regression analyses (paragraphs 1-4) sections; Boxes S2 and S3 Boxes, and Table 1. |
|  | 13d | Describe any methods used to synthesise results and provide a rationale for the choice(s). If meta-analysis was performed, describe the model(s), method(s) to identify the presence and extent of statistical heterogeneity, and software package(s) used. | Methods: Meta-analyses (paragraphs 1-4) and Meta-regression analyses (paragraphs 1-4) sections; Boxes S2 and S3 Boxes, and Table 1. |
|  | 13e | Describe any methods used to explore possible causes of heterogeneity among study results (e.g. subgroup analysis, metaregression). | Methods: Meta-analyses (paragraph 3) and Meta-regression analyses (paragraphs 1-4) sections; S3 Box. |
|  | 13f | Describe any sensitivity analyses conducted to assess robustness of the synthesised results. | Methods: Meta-regression analyses section (paragraphs 1-4); S3 Box, and Table 4. |
| Reporting bias assessment | 14 | Describe any methods used to assess risk of bias due to missing results in a synthesis (arising from reporting biases). | Methods: Precision, risk of bias, and publication bias assessments section (paragraph 4). |
| Certainty assessment | 15 | Describe any methods used to assess certainty (or confidence) in the body of evidence for an outcome. | Certainty (or confidence) in the body of evidence was not formally evaluated using a structured framework such as GRADE, given the focus on prevalence estimation rather than intervention effects. Instead, confidence in the evidence was assessed across complementary domains, including study precision, risk of bias, consistency of findings across populations and settings, and the magnitude and drivers of between-study heterogeneity.  This can be found in the Methods: Precision, risk of bias, and publication bias assessments section (paragraphs 1-4); S3 Table, Meta-analyses (paragraph 3) and Meta-regression analyses (paragraphs 1-4) sections. |
| **Results** | | |  |
| Study selection | 16a | Describe the results of the search and selection process, from the number of records identified in the search to the number of studies included in the review, ideally using a flow diagram (see Fig 1). | Results: Search results and scope of evidence section (paragraphs 1-2); Fig. 1. |
|  | 16b | Cite studies that might appear to meet the inclusion criteria, but which were excluded, and explain why they were excluded. | Fig. 1. |
| Study characteristics | 17 | Cite each included study and present its characteristics. | Results: Search results and scope of evidence section (paragraphs 3-6); Table S4, and S1 Fig. |
| Risk of bias in studies | 18 | Present assessments of risk of bias for each included study. | Results: Assessment of study precision, risk of bias, and publication bias section (paragraphs 1-3); S5 Table |
| Results of individual studies | 19 | For all outcomes, present, for each study: (a) summary statistics for each group (where appropriate) and (b) an effect estimate and its precision (e.g. confidence/credible interval), ideally using structured tables or plots. | Table 2 and S7-S8 Tables; S2-S3, and S7-S9 Figures. |
| Results of syntheses | 20a | For each synthesis, briefly summarise the characteristics and risk of bias among contributing studies. | Results: Assessment of study precision, risk of bias, and publication bias section (paragraphs 1-3); S5 Table. |
|  | 20b | Present results of all statistical syntheses conducted. If meta-analysis was done, present for each the summary estimate and its precision (e.g. confidence/credible interval) and measures of statistical heterogeneity. If comparing groups, describe the direction of the effect. | Results: Pooled mean estimates for NG prevalence section (paragraphs 1-9); Table 2 and S7-S9 Tables; S7-S9 Figures. |
|  | 20c | Present results of all investigations of possible causes of heterogeneity among study results. | Results: Associations with NG prevalence and sources of between-study heterogeneity section (paragraphs 1-6); Table 3. |
|  | 20d | Present results of all sensitivity analyses conducted to assess the robustness of the synthesised results. | Results: Associations with NG prevalence and sources of between-study heterogeneity section (paragraph 1); Table 4. |
| Reporting biases | 21 | Present assessments of risk of bias due to missing results (arising from reporting biases) for each synthesis assessed. | Results: Assessment of study precision, risk of bias, and publication bias section (paragraph 4); S6 Table and S4-S6 Figures. |
| Certainty of evidence | 22 | Present assessments of certainty (or confidence) in the body of evidence for each outcome assessed. | Results: Assessment of study precision, risk of bias, and publication bias section (paragraphs 1-4); S5 and S6 Tables, Pooled mean estimates for NG prevalence section (paragraphs 1-9); Table 2, S7-S9 Tables, and Associations with NG prevalence and sources of between-study heterogeneity section (paragraphs 1-6); Tables 3 and 4. |
| **Discussion** | | |  |
| Discussion | 23a | Provide a general interpretation of the results in the context of other evidence. | Discussion section (paragraphs 1-7). |
|  | 23b | Discuss any limitations of the evidence included in the review. | Discussion section (paragraphs 10-13). |
|  | 23c | Discuss any limitations of the review processes used. | Discussion section (paragraphs 14-17). |
|  | 23d | Discuss implications of the results for practice, policy, and future research. | Discussion section (paragraphs 8-9, and 21). |
| **Other information** | | |  |
| Registration and  protocol | 24a | Provide registration information for the review, including register name and registration number, or state that the review was not registered. | Methods: Data sources and search strategy section (paragraph 2). |
|  | 24b | Indicate where the review protocol can be accessed, or state that a protocol was not prepared. | Methods: Data sources and search strategy section (paragraph 2). |
|  | 24c | Describe and explain any amendments to information provided at registration or in the protocol. | Methods: Data sources and search strategy section (paragraph 2). |
| Support | 25 | Describe sources of financial or non-financial support for the review, and the role of the funders or sponsors in the review. | Methods: Role of the funding source section; Funding section. |
| Competing interests | 26 | Declare any competing interests of review authors. | Competing interests section. |
| Availability of data, code, and other materials | 27 | Report which of the following are publicly available and where they can be found: template data collection forms; data extracted from included studies; data used for all analyses; analytic code; any other materials used in the review. | Data availability statement section. |

# **Table B.** Search strategy. Database sources and systematic search strategies used to identify NG prevalence studies in sub-Saharan Africa.

| **PubMed (Last searched: June 4, 2025)** |
| --- |
| ("Neisseria gonorrhoeae"[Mesh] OR "Gonorrhea"[Mesh] OR "Pelvic Inflammatory Disease"[Mesh] OR "Epididymitis"[Mesh] OR "Orchitis"[Mesh] OR "Neisseria gonorrhoeae"[Text] OR "Gonorrhoeae"[Text] OR "Gonorrhea"[Text] OR "Gonococcus"[Text] OR "Gonococci"[Text] OR "Gonococcal"[Text] OR "Gonococcal infection"[Text] OR "Pelvic inflammatory disease"[Text] OR "Gonococcal epididymitis"[Text] OR "Orchi-epididymitis"[Text] OR "Orchiepididymitis"[Text] OR "seminal vesicle disease"[Text]) AND (("Africa South of the Sahara"[MeSH] OR "Comoros"[MeSH] OR "Ethiopia"[MeSH] OR "Madagascar"[ MeSH] OR "Mauritius"[ MeSH] OR "Sao Tome and Principe"[MeSH] OR "Seychelles"[ MeSH] OR Angola*[Text] OR Benin*[Text] OR Botswan*[Text] OR Batswana[Text] OR Burkina fas*[Text] OR Burkina*[Text] OR Burundi*[Text] OR Cameroon*[Text] OR Cabo Verde*[Text] OR cape verd*[Text] OR Central Africa Republic[Text] Central Africa*[Text] OR Chad*[Text] OR Comor*[Text] OR Congo*[Text] OR Cote d’Ivoire[Text] OR Ivorian*[Text] OR Democratic Republic of Congo[Text] OR Equatorial Guinea*[Text] OR Equatoguinean*[Text] OR Eritr*[Text] OR Ethiop*[Text] OR Gabon*[Text] OR Gambia*[Text] OR Ghana*[Text] OR Ghinea*[Text] OR Guinea-Bissau[Text] OR Kenya*[Text] OR Lesotho*[Text] OR Basotho*[Text] OR Liberia*[Text] OR Madagascar*[Text] OR Malagasy*[Text] OR Malawi*[Text] OR Mali*[Text] OR Maurit*[Text] OR Mozambi*[Text] OR Namibia*[Text] OR Niger*[Text] OR Nigeria*[Text] OR Rwanda*[Text] OR Sao Tome and Principe*[Text] OR Sao Tome*[Text] OR Senegal*[Text] OR Seychell*[Text] OR Sierra Leone*[Text] OR South Africa*[Text] OR Swazi*[Text] OR Togo*[Text] OR Uganda*[Text] OR United Republic of Tanzania[Text] OR Tanzan*[Text] OR Zambia*[Text] OR Zimbabwe*[Text] OR Mauritania*[Text])) |
| **Embase (Last searched: June 4, 2025)** |
| (exp gonorrhea / or exp neisseria gonorrhoeae / or exp epididymitis / or exp orchitis / or exp pelvic inflammatory disease/ or gonorrhea.mp. or neisseria gonorrhoeae.mp. or gonorrhoeae.mp. or gonococcus.mp. or gonococci.mp. or gonococcal.mp. or gonococcal infection.mp. or pelvic inflammatory disease.mp. or gonococcal epididymitis.mp. or orchi-epididymitis.mp. or orchiepididymitis.mp. or seminal vesicle disease.mp. or seminal disease.mp. or seminal vasculitis.mp.) and (exp "Africa south of the Sahara" or exp Southern African/ or African/ or exp Central African/ or exp West African/ or exp South African/ or exp Central African Republic/ or exp East African/ or exp Mauritius/ or exp Mauritania/ or exp "Sao Tome and Principe"/ or exp Seychelles/) or (angola* or Benin* or Botswan* or Batswana* or Burkina Fas* or Burkina* or Burundi* or Cameroon* or Cabo verde* or Cape Verd* or Central Africa* or "Central African Republic*" or Chad* or Comor* or Congo* or Cote D'ivoire* or Ivorian* or "Democratic Republic of Congo*" or Equatorial Guinea* or Equatoguinean* or Ethiop* or Eritr* or Gabon* or Gambia* or Ghana* or Ghinea* or Guinea-Bissau* or Kenya* or Lesotho* or Basotho* or Liberia* or Madagascar* or Malagasy* or Malawi* or Mali* or Maurit* or Mozambi* or Namibia* or Niger* or Nigeria* or Rwanda* or "Sao Tome and Principe*" or Sao Tome* or Senegal* or Seychell* or Sierra Leone* or South Africa* or Swazi* or Togo* or Uganda* or United republic of Tanzania* or Tanza* or Zambia* or Zimbabwe* or Mauritania*).mp. |
| **Web of Science (Last searched: June 4, 2025)** |
| ((((ALL=(gonorrhoeae)) OR ALL=(gonorrhea)) OR ALL=(*Neisseria gonorrhoeae*)) OR ALL=(gonococcus)) OR ALL=(gonococcal) and ANGOLA or BENIN or BOTSWANA or BURKINA FASO or BURUNDI or CAMEROON or CHAD or COMOROS or COTE IVOIRE or DEM REP CONGO or ESWATINI or ETHIOPIA or GABON or GAMBIA or GHANA or GUINEA or GUINEA BISSAU or KENYA or LESOTHO or LIBERIA or MADAGASCAR or MALAWI or MALI or MAURITANIA or MAURITIUS or MOZAMBIQUE or NAMIBIA or NIGER or NIGERIA or RWANDA or SENEGAL or SOUTH AFRICA or SWAZILAND or TOGO or UGANDA or TANZANIA or ZAMBIA or ZIMBABWE (Countries/Regions) |
| **SCOPUS (Last searched: June 4, 2025)** |
| ALL ( gonorrhea ) OR ALL ( neisseria AND gonorrhoeae ) OR ALL ( gonorrhoeae ) OR ALL ( gonococcus ) OR ALL ( gonococcal ) AND ( LIMIT-TO ( AFFILCOUNTRY , "Angola" ) OR LIMIT-TO ( AFFILCOUNTRY , "Benin" ) OR LIMIT-TO ( AFFILCOUNTRY , "Botswana" ) OR LIMIT-TO ( AFFILCOUNTRY , "Burkina Faso" ) OR LIMIT-TO ( AFFILCOUNTRY , "Burundi" ) OR LIMIT-TO ( AFFILCOUNTRY , "Cameroon" ) OR LIMIT-TO ( AFFILCOUNTRY , "Cabo Verde" ) OR LIMIT-TO ( AFFILCOUNTRY , "Central African Republic" ) OR LIMIT-TO ( AFFILCOUNTRY , "Chad" ) OR LIMIT-TO ( AFFILCOUNTRY , "Comoros" ) OR LIMIT-TO ( AFFILCOUNTRY , "Congo" ) OR LIMIT-TO ( AFFILCOUNTRY , "Cote d'Ivoire" ) OR LIMIT-TO ( AFFILCOUNTRY , "Democratic Republic Congo" ) OR LIMIT-TO ( AFFILCOUNTRY , "Equatorial Guinea" ) OR LIMIT-TO ( AFFILCOUNTRY , "Eritrea" ) OR LIMIT-TO ( AFFILCOUNTRY , "Ethiopia" ) OR LIMIT-TO ( AFFILCOUNTRY , "Gabon" ) OR LIMIT-TO ( AFFILCOUNTRY , "Gambia" ) OR LIMIT-TO ( AFFILCOUNTRY , "Ghana" ) OR LIMIT-TO ( AFFILCOUNTRY , "Guinea" ) OR LIMIT-TO ( AFFILCOUNTRY , "Guinea-Bissau" ) OR LIMIT-TO ( AFFILCOUNTRY , "Kenya" ) OR LIMIT-TO ( AFFILCOUNTRY , "Lesotho" ) OR LIMIT-TO ( AFFILCOUNTRY , "Liberia" ) OR LIMIT-TO ( AFFILCOUNTRY , "Madagascar" ) OR LIMIT-TO ( AFFILCOUNTRY , "Malawi" ) OR LIMIT-TO ( AFFILCOUNTRY , "Mali" ) OR LIMIT-TO ( AFFILCOUNTRY , "Mauritania" ) OR LIMIT-TO ( AFFILCOUNTRY , "Mauritius" ) OR LIMIT-TO ( AFFILCOUNTRY , "Mozambique" ) OR LIMIT-TO ( AFFILCOUNTRY , "Namibia" ) OR LIMIT-TO ( AFFILCOUNTRY , "Niger" ) OR LIMIT-TO ( AFFILCOUNTRY , "Nigeria" ) OR LIMIT-TO ( AFFILCOUNTRY , "Rwanda" ) OR LIMIT-TO ( AFFILCOUNTRY , "Sao Tome and Principe" ) OR LIMIT-TO ( AFFILCOUNTRY , "Senegal" ) OR LIMIT-TO ( AFFILCOUNTRY , "Seychelles" ) OR LIMIT-TO ( AFFILCOUNTRY , "Sierra Leone" ) OR LIMIT-TO ( AFFILCOUNTRY , "South Africa" ) OR LIMIT-TO ( AFFILCOUNTRY , "Swaziland" ) OR LIMIT-TO ( AFFILCOUNTRY , "Togo" ) OR LIMIT-TO ( AFFILCOUNTRY , "Uganda" ) OR LIMIT-TO ( AFFILCOUNTRY , "Tanzania" ) OR LIMIT-TO ( AFFILCOUNTRY , "Zambia" ) OR LIMIT-TO ( AFFILCOUNTRY , "Zimbabwe" ) ) |

Abbreviations: NG, *Neisseria gonorrhoeae*.

# Box A. Countries included in sub-Saharan Africa. Countries included and their subregional classification in the study definition of sub-Saharan Africa.

- **Central Africa:** Burundi, Cameroon, Central African Republic, Chad, Congo, Democratic Republic of Congo, Equatorial Guinea, Gabon, São Tomé and Principe.
- **Eastern Africa:** Comoros, Eritrea, Ethiopia, Kenya, Madagascar, Mauritius, Rwanda, Seychelles, Uganda, United Republic of Tanzania.
- **Northern Africa:** Mauritania.
- **Southern Africa:** Angola, Botswana, Eswatini, Lesotho, Malawi, Mozambique, Namibia, South Africa, Zambia, Zimbabwe.
- **Western Africa:** Benin, Burkina Faso, Cabo Verde, Côte d'Ivoire, Ghana, Guinea, Guinea-Bissau, Liberia, Mali, Niger, Nigeria, Senegal, Sierra Leone, The Gambia, Togo.

# Box B. Data extraction variables. Summary of variables extracted from eligible studies.

- Author(s)
- Year of publication
- Full citation
- Country
- City
- Study design
- Sampling methodology
- Year(s) of data collection
- Study site
- Study population
- Population characteristics (e.g., sex and age)
- Response rate
- Sample size of tested population
- Number of participants positive for NG infection
- Reported NG prevalence
- Anatomical site: urogenital (urethral, vaginal, endocervical, urine, and semen), anorectal, oropharyngeal, serum, mixed and unclear specimen
- Type of assay used for infection ascertainment: NAAT/PCR, culture, Gram stain/microscopy, rapid test, ELISA/EIA, serological assays (e.g., haemagglutination, complement fixation, immunoglobulin measurements such as IgG or IgA), mixed assays, and assays with unclear classification.

Abbreviations: EIA, Enzyme immunoassay; ELISA, Enzyme-linked immunosorbent assay; NAAT, Nucleic acid amplification test; NG, *Neisseria gonorrhoeae*; PCR, Polymerase chain reaction.

# Table C. Quality assessment. Range of quality assessment components relevant to prevalence studies and their applicability to this systematic review and the included studies reporting NG prevalence.

| **Risk of bias tool from Hoy, 2012 [2]** | **Risk of bias tool from Munn, 2015 [3]** | **Risk of bias assessment of included studies** |
| --- | --- | --- |
| Was the study’s target population a close representation of the national population in relation to relevant variables? | Were study participants sampled in an appropriate way? | Met in the study design of this systematic review. The systematic review investigated prevalence in all population groups. The meta-regression analyses explored the impact of population type on observed prevalence. The sampling method was one of the investigated risk of bias domains. |
| Was the sampling frame a true or close representation of the target population? | Was the sample frame appropriate to address the target population? | Met in the study design of this systematic review. Included as the probability-based versus non-probability-based sampling risk of bias domain. The meta-regression analyses also explored the impact of sampling method on observed prevalence. |
| Was some form of random selection used to select the sample, OR was a census undertaken? |  | Met in the study design of this systematic review. Included as the probability-based versus non-probability-based sampling risk of bias domain. The meta-regression analyses also explored the impact of sampling method on observed prevalence. |
| Was the likelihood of nonresponse bias minimal? | Was the response rate adequate, and if not, was the low response rate managed appropriately? | Met in the study design of this systematic review. Included as the response rate risk of bias domain. The meta-regression analyses also explored the impact of response rate on observed prevalence. |
| Were data collected directly from the subjects (as opposed to a proxy)? |  | Met in the study design of this systematic review. The inclusion criteria specified that only studies based on biomarkers collected directly from individuals are included in this systematic review. |
| Was an acceptable case definition used in the study? | Were valid methods used for the identification of the condition? | Met in the study design of this systematic review. A standardized and consistent case definition was used, that of NG infection diagnosed through laboratory methods. |
| Was the study instrument that measured the parameter of interest shown to have validity and reliability? | Was the condition measured in a standard, reliable way for all participants? | Met in the study design of this systematic review. The laboratory methods utilized in the included studies are generally well-established, with acceptable levels of specificity and sensitivity and are widely employed in both research and clinical settings. Importantly, the meta-regression analyses examined the effect of assay type on the observed prevalence. |
| Was the same mode of data collection used for all subjects? |  | Met in the study design of this systematic review. It is standard for STI studies involving biomarkers, by design, to use a consistent mode of data collection from all subjects recruited for a study, including specimen type and assay type. |
| Was the length of the shortest prevalence period for the parameter of interest appropriate? |  | Met in the study design of this systematic review. Included studies reported point prevalence measures, that is, prevalence based on a cross-sectional survey at a specific and appropriately relevant time interval. |
| Were the numerator(s) and denominator(s) for the parameter of interest appropriate? | Was there appropriate statistical analysis? | Met in the study design of this systematic review. The numerator and denominator were defined with no ambiguity: number of positive NG cases over total number of tested subjects. |
|  | Was the data analysis conducted with sufficient coverage of the identified sample? | Met in the study design of this systematic review. It is standard practice in such STI studies for data analysis to be conducted on the full reported and tested sample. |
|  | Was the sample size adequate? | Met in the study design of this systematic review. Included as the precision assessment of the sample size. Importantly, the meta-regression analyses explored the impact of sample size on observed prevalence. |
|  | Were the study subjects and the setting described in detail? | Met in the study design of this systematic review. For all included studies, the population type of study subjects and the study site were available and extracted. Populations were classified according to this systematic review definitions of study populations. |

Abbreviations: NG, *Neisseria gonorrhoeae*; STI, Sexually transmitted infection.

# Box C. Analysis variables. Factors (variables) predefined *a priori* and included in both univariable and multivariable meta-regression analyses.

| 1. Population classification as defined in Table 1 2. Sex 3. Age groups classified to best fit reported data as:  - <25 years - 25-34 years - 35-44 years - ≥45 years - Mixed age bands  1. Assay type:  - NAAT/PCR - Culture - Gram Stain/microscopy - Rapid test - ELISA/EIA - Mixed assays - Unclear  1. Sample size:  - <200 - ≥200  1. Sampling method:  - Probability-based sampling - Non-probability-based sampling  1. Response rate:  - <80% - ≥80% - Unclear  1. Year of publication category:  - <2005 - 2005-2014 - ≥2015  1. Year of publication as a linear term 2. Year of data collection category^*^  - <2000 - 2000-2009 - ≥2010  1. Year of data collection as a linear term |
| --- |

Abbreviations: EIA, Enzyme immunoassay; ELISA, Enzyme-linked immunosorbent assay; NAAT, Nucleic acid amplification test; PCR, Polymerase chain reaction.

^*^The categories were defined based on the observed median interval of 3.5 years between the year of data collection and the year of publication, which was rounded to 5 years to create a standardized 5-year bracket.

#

# Table D. Included publications. List of publications included in this systematic review, from which NG prevalence data were extracted.

| **Records identified through database searches:**   1. Abauleth R, Boni S, Kouassi-Mbengue A, Konan J, Deza S. Causation and treatment of infectious leucorrhoea at the Cocody University Hospital (Abidjan, Cote d'Ivoire). [French]. *Sante (Montrouge, France)* 2006; **16**(3): 191-5. 2. Abbai NS, Moodley P, Reddy T, et al. Clinical evaluation of the OneStep Gonorrhea RapiCard InstaTest for detection of *Neisseria gonorrhoeae* in symptomatic patients from KwaZulu-Natal, South Africa. *J Clin Microbiol* 2015; **53**(4): 1348-50. 3. Abdullahi A, Nzou SM, Kikuvi G, Mwau M. *Neisseria gonorrhoeae* infection in female sex workers in an STI clinic in Nairobi, Kenya. *PLoS ONE* 2022; **17**(2 February): e0263531. 4. Aboud S, Buhalata SN, Onduru OG, et al. High Prevalence of Sexually Transmitted and Reproductive Tract Infections (STI/RTIs) among Patients Attending STI/Outpatient Department Clinics in Tanzania. *Tropical Medicine and Infectious Disease* 2023; **8**(1): 62. 5. Aboud S, Msamanga G, Read JS, et al. Genital tract infections among HIV-infected pregnant women in Malawi, Tanzania and Zambia. *International Journal of STD and AIDS* 2008; **19**(12): 824-32. 6. Aboyeji AP, Nwabuisi C. Prevalence of sexually transmitted diseases among pregnant women in Ilorin, Nigeria. *Journal of Obstetrics and Gynaecology* 2003; **23**(6): 637-9. 7. Achamyeleh H, Toru M, Mekuriaw TD. PREVALENCE, ANTIMICROBIAL SUSCEPTIBILITY PATTERN, AND ASSOCIATED FACTORS OF *NEISSERIA GONORRHOEAE* AMONG WOMEN ATTENDING AT DEBRE MARKOS TOWN HEALTH INSTITUTIONS, NORTHWEST ETHIOPIA. *Clinical Chemistry and Laboratory Medicine* 2023; **61**(Supplement 1): S1499. 8. Achilles SL, Austin MN, Meyn LA, Mhlanga F, Chirenje ZM, Hillier SL. Impact of contraceptive initiation on vaginal microbiota. *Am J Obstet Gynecol* 2018; **218**(6): 622.e1-.e10. 9. Adachi K, Klausner JD, Bristow CC, et al. Chlamydia and Gonorrhea in HIV-Infected Pregnant Women and Infant HIV Transmission. *Sex Transm Dis* 2015; **42**(10): 554-65. 10. Affolabi D, Goma E, Sogbo F, et al. Antimicrobial susceptibility profile of *Neisseria gonorrhoeae* isolated in Cotonou, Benin (2015-2017). *Sexually Transmitted Infections* 2018; **94**(1): 20. 11. Agyarko-Poku T. Aetiological agents of infective vaginal discharge among women attending a STD clinic in Kumasi, Ghana. *Sexually Transmitted Infections* 2011; **87**: A305. 12. Agyarko-Poku T, Buabeng K, Ofori AO, Sarkodie YA. *Neisseria gonorrhoeae* infections among people living with HIV on art at STI clinic in Kumasi, Ghana. *Sexually Transmitted Infections* 2019; **95 (Supplement 1)**: A134. 13. Agyarko-Poku T, Sarkodie YA, Frempong EH. Bacterial vaginosis: Leading cause of vaginal discharge among women attending sexually transmitted infection clinic in Kumasi, Ghana. *Sexually Transmitted Infections* 2017; **93 (Supplement 2)**: A75. 14. Aho J, Koushik A, Coutlee F, Diakite SL, Rashed S. Prevalence of HIV, human papillomavirus type 16 and herpes simplex virus type 2 among female sex workers in Guinea and associated factors. *International Journal of STD and AIDS* 2014; **25**(4): 280-8. 15. Aimakhu VE. Treatment of trichomonal vaginitis with a single oral dose of tinidazole. *INTJGYNAECOBSTET* 1974; **12**(3): 84-7. 16. Ajani AA, Olanrewaju FO, Oripelaye M, Enitan AO, Olasode OA. Impact of SARS-CoV-2 pandemic on sexually transmitted infections and treatment outcomes at a healthcare facility in Ile-Ife, Nigeria. *Infect Dis Trop Med* 2024; **10**. 17. Akerele J, Abhulimen P, Okonofua F. Prevalence of asymptomatic genital infection among pregnant women in Benin City, Nigeria. *African journal of reproductive health* 2002; **6**(3): 93-7. 18. Alary M, Baganizi E, Guedeme A, et al. Evaluation of clinical algorithms for the diagnosis of gonococcal and chlamydial infections among men with urethral discharge or dysuria and women with vaginal discharge in Benin. *Sexually Transmitted Infections* 1998; **74**(SUPPL. 1): S44-S9. 19. Alary M, Behanzin L, Guedou FA, et al. Contribution of biomarkers to the validation of self-reported condom use in a treatment as prevention and a pre-exposure prophylaxis demonstration study among female sex workers. *Sexually Transmitted Infections* 2015; **91**: A242. 20. Alary M, Gbenafa-Agossa C, Aina G, et al. Evaluation of a rapid point-of-care test for the detection of gonococcal infection among female sex workers in Benin. *Sexually Transmitted Infections* 2006; **82**(SUPPL. 5): v29-v32. 21. Alary M, Mukenge-Tshibaka L, Bernier F, et al. Decline in the prevalence of HIV and sexually transmitted diseases among female sex workers in Cotonou, Benin, 1993-1999. *AIDS* 2002; **16**(3): 463-70. 22. Alausa KO, Osoba AO. Epidemiology of gonococcal vulvovaginitis among children in the tropics. *Br J Vener Dis* 1980; **56**(4): 239-42. 23. Alcaide ML, Chitalu N, Jones DL, Weiss S. Chlamydia and gonorrhea infections in HIV-positive women in urban Lusaka, Zambia. *Journal of Global Infectious Diseases* 2012; **4**(3): 141-4. 24. Alexandre I, Justel M, Martinez P, de Lejarazu RO, Pastor JC. First Attempt to Implement Ophthalmia Neonatorum Prophylaxis in Angola: Microorganisms, Efficacy, and Obstacles. *JOURNAL OF OPHTHALMOLOGY* 2015; **2015**. 25. Ali S, Sewunet T, Sahlemariam Z, Kibru G. *Neisseria gonorrhoeae* among suspects of sexually transmitted infection in Gambella hospital, Ethiopia: risk factors and drug resistance. *BMC research notes* 2016; **9**(1): 439. 26. Allen L, Schoon M. Diagnostic laparoscopy and management of patients with confirmed acute pelvic inflammatory disease. *South African Medical Journal* 1984; **65**(6): 201-2. 27. Aly N, Stach JL, Cartel JL, Bah D, Correa P. ADVANTAGE OF ELISA TEST IN EPIDEMIOLOGICAL-STUDY OF GONOCOCCAL INFECTIONS OF WOMEN. *ANNALES DE MICROBIOLOGIE* 1980; **A131**(1): 88-9. 28. Amin ET, Njumkeng C, Kika BT, Fualefac A, Njukeng P. Pattern of Antimicrobial Resistance among Bacterial Isolates from Urogenital Clinical Specimens: A Descriptive Study from the Buea Health District, Cameroon. *Drugs - Real World Outcomes* 2018; **5**(2): 101-8. 29. Amito Florence P, Otim F, Okongo F, Ogwang M, Greco D. The prevalence and antibiotics susceptibility pattern of *Neisseria gonorrhoeae* in patients attending OPD clinics at St. Mary's Hospital Lacor Uganda. *J Prev Med Hyg* 2012; **53**(4): 186-9. 30. Anahtar MN, Byrne EH, Doherty KE, et al. Cervicovaginal Bacteria Are a Major Modulator of Host Inflammatory Responses in the Female Genital Tract. *Immunity* 2015; **42**(5): 965-76. 31. Anderson BL, Firnhaber C, Liu T, et al. Effect of trichomoniasis therapy on genital HIV viral burden among African women. *Sex Transm Dis* 2012; **39**(8): 638-42. 32. Anorlu R, Imosemi D, Odunukwe N, Abudu O, Otuonye M. Prevalence of HIV among Women with Vaginal Discharge in a Gynecological Clinic. *Journal of the National Medical Association* 2004; **96**(3): 367-71. 33. Anoukoum T, Baeta S, Attipou K, Pitche P, James EY, Tchangai-Walla K. Condyloma acuminatum of the urogenital tract and its urological complications. Study in 257 cases. *J UROL* 1996; **102**(5-6): 212-5. 34. Anyanwu BN, Nwanebu FC, Uchegbu UM, Ugwuegbulam JN. Prevalence of sexually transmitted diseases (STDs) in Owerri, Nigeria. *International Journal of Environmental Health Research* 1996; **6**(2): 153-8. 35. Ao TT, Sam NE, Masenga EJ, Seage III GR, Kapiga SH. Human immunodeficiency virus type 1 among bar and hotel workers in northern Tanzania: the role of alcohol, sexual behavior, and herpes simplex virus type 2. *Sexually transmitted diseases* 2006; **33**(3): 163-9. 36. Apea-Kubi KA, Yamaguchi S, Sakyi B, Kishimoto T, Ofori-Adjei D, Hagiwara T. *Neisseria gonorrhoea*, Chlamydia trachomatis, and Treponema pallidum infection in antenatal and gynecological patients at Korle-Bu Teaching Hospital, Ghana. *Jpn J Infect Dis* 2004; **57**(6): 253-6. 37. Apers L, Zishiri C. Clinical and biomedical aspects of gonorrhoea, diagnosed in symptomatic patients in Midlands Province, Zimbabwe. *The Central African journal of medicine* 2002; **48**(7-8): 94-5. 38. Arenholt LTS, Randrianasolo BS, Rabozakandraina TOO, et al. Repeated versus single praziquantel dosing regimen in treatment of female genital schistosomiasis: a phase 2 randomised controlled trial showing no difference in efficacy. *Front Trop Dis* 2024; **5**. 39. Argent AC, Lachman PI, Hanslo D, Bass D. Sexually transmitted diseases in children and evidence of sexual abuse. *Child Abuse Negl* 1995; **19**(10): 1303-10. 40. Arowojolu AO, Bakare RA, Oni AA, Ilesanmi A. Laparoscopic and microbiological features of acute salpingitis in developing countries. *Journal of Obstetrics and Gynaecology* 1998; **18**(2): 164-8. 41. Arya OP, Nsanzumuhire H, Taber SR. Clinical, cultural, and demographic aspects of gonorrhoea in a rural community in Uganda. *Bull World Health Organ* 1973; **49**(6): 587-95. 42. Arya OP, Taber SR, Nsanze H. Gonorrhea and female infertility in rural Uganda. *Am J Obstet Gynecol* 1980; **138**(7 Pt 2): 929-32. 43. Asamoah-Adu C, Khonde N, Avorkliah M, et al. HIV infection among sex workers in Accra: Need to target new recruits entering the trade. *Journal of Acquired Immune Deficiency Syndromes* 2001; **28**(4): 358-66. 44. Asare K, Osman F, Ngcapu S, et al. Burden of sexually transmitted infections from acute HIV infection among women in South Africa: Evidence from a prospective cohort study. *Annals of Epidemiology* 2022; **74**: 132-9. 45. Asmah RH, Blankson HNA, Seanefu KA, et al. Trichomoniasis and associated co-infections of the genital tract among pregnant women presenting at two hospitals in Ghana. *BMC Women's Health* 2017; **17**(1). 46. Ayalew E, Fentaw S, Ebrahim S, Seyoum E, Woldesenbet Z, Wolde M. Comparison of syndromic versus laboratory-confirmed diagnosis of *Neisseria gonorrhoeae* and *Treponema pallidum*, infections at the selected health centers in Addis Ababa, Ethiopia (vol 19, 88, 2022). *REPRODUCTIVE HEALTH* 2022; **19**(1). 47. Babaoye FA, Ogala WN, Muhammad I. Dysuria in infancy and childhood: an analysis of 42 children presenting in the paediatrics outpatients department. *East Afr Med J* 1991; **68**(11): 860-4. 48. Bah OR, Diallo AB, Diallo A, et al. Male infertility: Frequency and aetiological aspects in the Urology-Andrology department of Conakry hospital. *Andrologie* 2007; **17**(3): 241-5. 49. Baisley K, Changalucha J, Weiss HA, et al. Bacterial vaginosis in female facility workers in north-western Tanzania: Prevalence and risk factors. *Sexually Transmitted Infections* 2009; **85**(5): 370-5. 50. Bakare RA, Ashiru JO, Adeyemi-Doro FA, et al. Non-gonococcal urethritis (NGU) due to trichomonas vaginalis in Ibadan. *West Afr J Med* 1999; **18**(1): 64-8. 51. Bakare RA, Oni AA, Arowojolu AO, et al. Efficacy of pefloxacin in acute gonococcal urethritis. *African journal of medicine and medical sciences* 1997; **26**(3-4): 185-6. 52. Bakare RA, Oni AA, Arowojolu AO, et al. Penicillinase producing *Neisseria gonnorhoeae*: the review of the present situation in Ibadan, Nigeria. *Niger Postgrad Med J* 2002c; **9**(2): 59-62. 53. Bakare RA, Oni AA, Umar US, et al. Pattern of sexually transmitted diseases among commercial sex workers (CSWs) in Ibadan, Nigeria. *African journal of medicine and medical sciences* 2002a; **31**(3): 243-7. 54. Bakare RA, Oni AA, Umar US, Kehinde AO, Fayemiwo SA, Fasina NA. Ureaplasma urealyticum as a cause of non-gonococcal urethritis: the Ibadan experience. *The Nigerian postgraduate medical journal* 2002b; **9**(3): 140-5. 55. Bakare RA, Oni AA, Umar US, et al. Non-gonococcal urethritis due to Chlamydia trachomatis: the Ibadan experience. *African journal of medicine and medical sciences* 2002d; **31**(1): 17-20. 56. Balkus JE, Srinivasan S, Anzala O, et al. Impact of periodic presumptive treatment for bacterial vaginosis on the vaginal microbiome among women participating in the preventing vaginal infections trial. *The Journal of infectious diseases* 2017; **215**(5): 723-31. 57. Ball M, Kanga JM, Meilo H, Debeugny B. TREATMENT OF ACUTE GONOCOCCAL URETHRITIS IN MEN WITH A SINGLE DOSE OF 800 MG PEFLOXACIN. *BRITISH JOURNAL OF CLINICAL PRACTICE* 1990; **44**(4): 140-1. 58. Ballard RC, Fehler HG. Chlamydial infections of the eye and genital tract in southern Africa. *S Afr Med J* 1986; **Suppl**: 76-9. 59. Ballard RC, Fehler HG, Htun Y, Radebe F, Jensen JS, Taylor-Robinson D. Coexistence of urethritis with genital ulcer disease in South Africa: Influence on provision of syndromic management. *Sexually Transmitted Infections* 2002; **78**(4): 274-7. 60. Ballard RC, Schoub BD, Schneider J, Robins-Browne RM, Koornhof HJ. Urethritis in white men--a microbiological appraisal. *S Afr Med J* 1977; **51**(20): 702-6. 61. Balle C, Gill K, Konstantinus IN, et al. Hormonal contraception and risk of STIs and bacterial vaginosis in South African adolescents: secondary analysis of a randomised trial. *Sex Transm Infect* 2021; **97**(2): 112-7. 62. Banura C, Franceschi S, Van Doorn LJ, et al. Prevalence, incidence and clearance of human papillomavirus infection among young primiparous pregnant women in Kampala, Uganda. *Int J Cancer* 2008; **123**(9): 2180-7. 63. Baribwira C, Muteganya D, Ndihokubwayo JB, Moreno JL, Nduwimana M, Rufyikiri T. An aspect of sexually transmitted diseases in infants in Burundi: Gonorrhea due to sexual abuse. [French]. *Medecine Tropicale* 1994; **54**(3): 231-3. 64. Barnabas SL, Dabee S, Passmore JS, et al. Converging epidemics of sexually transmitted infections and bacterial vaginosis in southern African female adolescents at risk of HIV. *Int J STD AIDS* 2018; **29**(6): 531-9. 65. Barry MS, Ba Diallo A, Diadhiou M, et al. Accuracy of syndromic management in targeting vaginal and cervical infections among symptomatic women of reproductive age attending primary care clinics in Dakar, Senegal. *Trop Med Int Health* 2018; **23**(5): 541-8. 66. Bayigga L, Nabatanzi R, Ssekagiri A, et al. Diverse vaginal microbiome was associated with pro-inflammatory vaginal milieu among pregnant women in Uganda. *Human Microbiome Journal* 2020; **18 (no pagination)**. 67. Beesham I, Isehunwa O, Kriel Y, et al. Sexually Transmitted Infection Prevalence, Partner Notification, and Human Immunodeficiency Virus Risk Perception in a Cohort of Women Completing Sexually Transmitted Infection Screening as Part of a Safer Conception Study. *Sexually Transmitted Diseases* 2024; **51**(6): 431-6. 68. Behanzin L, Diabate S, Minani I, et al. Decline in the prevalence of HIV and sexually transmitted infections among female sex workers in Benin over 15 years of targeted interventions. *Journal of Acquired Immune Deficiency Syndromes* 2013; **63**(1): 126-34. 69. Behanzin L, Diabate S, Minani I, et al. Decline in HIV Prevalence among Young Men in the General Population of Cotonou, Benin, 1998-2008. *PLoS ONE* 2012; **7**(8). 70. Behets FM, Andriamiadana J, Randrianasolo D, et al. Laboratory diagnosis of sexually transmitted infections in women with genital discharge in Madagascar: implications for primary care. *Int J STD AIDS* 2002; **13**(9): 606-11. 71. Behets FMTF, Rasolofomanana JR, Van Damme K, et al. Evidence-based treatment guidelines for sexually transmitted infections developed with and for female sex workers. *Tropical Medicine and International Health* 2003; **8**(3): 251-8. 72. Behling J, Chan AK, Zeh C, Nekesa C, Heinzerling L. Evaluating HIV prevention programs: Herpes simplex virus type 2 antibodies as biomarker for sexual risk behavior in young adults in resource-poor countries. *PLoS ONE* 2015; **10**(5). 73. Bekker LG, Das M, Abdool Karim Q, et al. Twice-Yearly Lenacapavir or Daily F/TAF for HIV Prevention in Cisgender Women. *New England Journal of Medicine* 2024; **391**(13): 1179 EP - 92. 74. Bello CS. Screening for gonorrhoea among college students in Zaria, Nigeria. *Med J Zambia* 1981; **15**(3): 75-7. 75. Bello CSS. Gonococcal vulvo-vaginitis in children: The Zaria experience. *NIGER J PAEDIATR* 1982a; **9**(3): 75-9. 76. Bello CSS, Elegba OY, Dada JD. SEXUALLY-TRANSMITTED DISEASES IN NORTHERN NIGERIA - 5 YEARS EXPERIENCE IN A UNIVERSITY TEACHING HOSPITAL CLINIC. *BRITISH JOURNAL OF VENEREAL DISEASES* 1983; **59**(3): 202-5. 77. Bello CSS, Idiong DU. Schistosoma urethritis: Pseudo-gonorrhoeal disease in Northern Nigeria. *Tropical Doctor* 1982b; **12**(3): 141-2. 78. Bennett FJ. GONORRHOEA: A RURAL PATTERN OF TRANSMISSION. *East Afr Med J* 1964; **41**: 163-7. 79. Bentsi C, Klufio CA, Perine PL, et al. Genital infections with Chlamydia trachomatis and *Neisseria gonorrhoeae* in Ghanaian women. *Genitourinary Medicine* 1985; **61**(1): 48-50. 80. Birhanu M, Abegaz WE, Schroder D, et al. Antimicrobial susceptibility in *Neisseria gonorrhoeae* and epidemiological data of gonorrhoea patients in five cities across Ethiopia, 2021-22. *JAC-Antimicrobial Resistance* 2024; **6**(1): dlae002. 81. Bitew A, Mengist A, Belew H, Aschale Y, Reta A. The prevalence, antibiotic resistance pattern, and associated factors of bacterial vaginosis among women of the reproductive age group from felege Hiwot referral hospital, Ethiopia. *Infection and Drug Resistance* 2021; **14**: 2685-96. 82. Bitew A, Tegene B, Yeshitela B, Howe R, Abate E, Dagnew M. Bacterial profile, antibacterial susceptibility pattern and associated factors among women attending antenatal and postnatal health services at the University of Gondar Teaching Hospital, northwest Ethiopia. *Ethiop Med J* 2019; **57**(1): 9-22. 83. Black V, Magooa P, Radebe F, Myers M, Pillay C, Lewis DA. The detection of urethritis pathogens among patients with the male urethritis syndrome, genital ulcer syndrome and HIV voluntary counselling and testing clients: Should South Africa's syndromic management approach be revised? *Sexually Transmitted Infections* 2008; **84**(4): 254-8. 84. Blankhart D, Muller O, Gresenguet G, Weis P. Sexually transmitted infections in young pregnant women in Bangui, Central African Republic. *International Journal of STD and AIDS* 1999; **10**(9): 609-14. 85. Blavo Kouame BE, Angora KE, Yeo A, et al. Contribution of PCR in the biological diagnosis of trichomonas vaginalis infection at institut pasteur of cote d'ivoire. *Sexually Transmitted Infections* 2017; **93 (Supplement 2)**: A46. 86. Bogaerts J, Lepage P, De Clercq A, et al. Shigella and gonococcal vulvovaginitis in prepubertal Central African girls. *Pediatric Infectious Disease Journal* 1992; **11**(10): 890-2. 87. Bogaerts J, Martinez Tello W, Verbist L, Piot P, Vandepitte J. Norfloxacin versus thiamphenicol for treatment of uncomplicated gonorrhea in Rwanda. *Antimicrob Agents Chemother* 1987; **31**(3): 434-7. 88. Bogaerts J, Ricart CA, Van Dyck E, Piot P. The etiology of genital ulceration in Rwanda. *Sex Transm Dis* 1989; **16**(3): 123-6. 89. Bogaerts J, Tello WM, Akingeneye J, Mukantabana V, Van Dyck E, Piot P. Effectiveness of norfloxacin and ofloxacin for treatment of gonorrhoea and decrease of in vitro susceptibility to quinolones over time in Rwanda. *Genitourin Med* 1993; **69**(3): 196-200. 90. Bogaerts J, Verhaegen J, Martinez Tello W, et al. Characterization, in vitro susceptibility, and clinical significance of CDC group HB-5 from Rwanda. *J Clin Microbiol* 1990; **28**(10): 2196-9. 91. Boger MF, Hasselrot T, Kaldhusdal V, et al. Sustained immune activation and impaired epithelial barrier integrity in the ectocervix of women with chronic HIV infection. *PLoS Pathogens* 2024; **20**(11): e1012709. 92. Bossard C, Chihana M, Nicholas S, et al. HIV, sexual violence, and termination of pregnancy among adolescent and adult female sex workers in Malawi: A respondent-driven sampling study. *PLoS ONE* 2022; **17**(12 December): e0279692. 93. Bourgeois A, Henzel D, Dibanga G, et al. [Development and evaluation of screening algorithms for sexually transmitted diseases in pregnant women at Libreville, Gabon]. *Sante* 1996; **6**(2): 115-21. 94. Bourgeois A, Henzel D, Malonga-Mouelet G, et al. Clinical algorithms for the screening of pregnant women for STDs in Libreville, Gabon: Which alternatives? *Sexually Transmitted Infections* 1998; **74**(1): 35-9. 95. Bouwhuis SA, Davis MDP. Contribution of sexually transmitted diseases and socioeconomic factors to perinatal mortality in rural Ghana. *International Journal of Dermatology* 2004; **43**(1): 27-30. 96. Brabin L, Kemp J, Dollimore N, et al. Reproductive tract infections and abortion among adolescent girls in rural Nigeria. *The Lancet* 1995; **345**(8945): 300-4. 97. Braddick MR, Ndinya-Achola JO, Mirza NB, et al. Towards developing a diagnostic algorithm for Chlamydia trachomatis and *Neisseria gonorrhoeae* cervicitis in pregnancy. *Genitourin Med* 1990a; **66**(2): 62-5. 98. Braunstein SL, Ingabire CM, Kestelyn E, et al. High human immunodeficiency virus incidence in a cohort of Rwandan female sex workers. *Sex Transm Dis* 2011; **38**(5): 385-94. 99. Brown IM, Cruickshank JG. Aetiological factors in pelvic inflammatory disease in urban Blacks in Rhodesia. *S Afr Med J* 1976; **50**(34): 1342-4. 100. Brown LB, Krysiak R, Kamanga G, et al. *Neisseria gonorrhoeae* antimicrobial susceptibility in Lilongwe, Malawi, 2007. *Sexually Transmitted Diseases* 2010; **37**(3): 169-72. 101. Buhalata SN, Kwesigabo G, Sembuche S, Aboud S, Temu MM, Changalucha JM. Genital tract infections in women attending sexually transmitted infection clinics in Mwanza, north-west Tanzania. *Southern African Journal of Epidemiology and Infection* 2013; **28**(1): 48-54. 102. Bukusi EA, Cohen CR, Meier AS, et al. Bacterial vaginosis: Risk factors among Kenyan women and their male partners. *Sexually Transmitted Diseases* 2006; **33**(6): 361-7. 103. Bukusi EA, Cohen CR, Stevens CE, et al. Effects of human immunodeficiency virus 1 infection on microbial origins of pelvic inflammatory disease and on efficacy of ambulatory oral therapy. *Am J Obstet Gynecol* 1999; **181**(6): 1374-81. 104. Burchard GD, Wolff T. [Study of bacterial sensitivity in a Lambaréné hospital (Gabon)]. *Med Trop (Mars)* 1985; **45**(3): 265-9. 105. Burchell HJ, Welgemoed NC. MICROBIOLOGICAL ETIOLOGY OF ACUTE PELVIC INFLAMMATORY DISEASE AT PELONOMI-HOSPITAL, BLOEMFONTEIN. *SOUTH AFRICAN MEDICAL JOURNAL* 1988; **73**(2): 81-2. 106. Burney P. Some aspects of sexually transmitted disease in Swaziland. *Br J Vener Dis* 1976; **52**(6): 412-4. 107. Butcher R, Jarju S, Obayemi D, et al. Prevalence of five treatable sexually transmitted infections among women in Lower River region of The Gambia. *BMC Infectious Diseases* 2023; **23**(1): 471. 108. Buve A, Weiss HA, Laga M, et al. The epidemiology of gonorrhoea, chlamydial infection and syphilis in four African cities. *AIDS* 2001; **15**(SUPPL. 4): S79-S88. 109. Bwayo JJ, Omari AM, Mutere AN, et al. Long distance truck-drivers: 1. Prevalence of sexually transmitted diseases (STDs). *East Afr Med J* 1991; **68**(6): 425-9. 110. Caceres CF, Celentano DD, Coates TJ, et al. Sexually transmitted disease and HIV prevalence and risk factors in concentrated and generalized HIV epidemic settings. *AIDS* 2007; **21**(SUPPL. 2): S81-S90. 111. Carty MJ, Nzioki JM, Verhagen AR. The role of gonococcus in acute pelvic inflammatory disease in Nairobi. *East Afr Med J* 1972; **49**(5): 376-9. 112. Carveth-Johnson T, Dunin De Skrzynno S, Wynn A, et al. Integrating STI testing and treatment with routine HIV care in Gaborone, Botswana. *Sexually transmitted diseases* 2021; **26**. 113. Celum CL, Bukusi EA, Bekker LG, et al. PrEP use and HIV seroconversion rates in adolescent girls and young women from Kenya and South Africa: the POWER demonstration project. *Journal of the International AIDS Society* 2022; **25**(7): e25962. 114. Celum CL, Gill K, Morton JF, et al. Incentives conditioned on tenofovir levels to support PrEP adherence among young South African women: a randomized trial. *Journal of the International AIDS Society* 2020; **23**(11): e25636. 115. Chabata ST, Fearon E, Musemburi S, et al. High Prevalence of Sexually Transmitted Infections and Poor Sensitivity and Specificity of Screening Algorithms for Chlamydia and Gonorrhea Among Female Sex Workers in Zimbabwe: Analysis of Respondent-Driven Sampling Surveys in 3 Communities. *Sex Transm Dis* 2025; **52**(2): 117-24. 116. Chalamilla G, Mbwana J, Mhalu F, et al. Patterns of sexually transmitted infections in adolescents and youth in Dar es Salaam, Tanzania. *BMC Infectious Diseases* 2006; **6 (no pagination)**. 117. Chanzu NM, Mwanda W, Oyugi J, Anzala O. Mucosal blood group antigen expression profiles and HIV infections: A study among female sex workers in Kenya. *PLoS ONE* 2015; **10**(7). 118. Chaponda EB, Bruce J, Michelo C, Chandramohan D, Chico RM. Assessment of syndromic management of curable sexually transmitted and reproductive tract infections among pregnant women: an observational cross-sectional study. *BMC Pregnancy and Childbirth* 2021; **21**(1). 119. Charanchi SM, Kudi AA, Tahir F. Antimicrobial sensitivity patterns of urogenital bacterial isolates among Hiv positive patients in the federal medical centre in gombe. *Internet Journal of Infectious Diseases* 2011; **10**(1). 120. Chaudhry S, Sangani B, Ojwang SB, Khan KS. Retrospective study of alleged sexual assault at the Aga Khan Hospital, Nairobi. *East Afr Med J* 1995; **72**(3): 200-2. 121. Chawafambira TA, Kufa T, Pascoe M, Lowe S. High prevalence of sexually transmitted infections in pregnant women living with HIV in Harare, Zimbabwe: A cross-sectional study. *Sexual Health* 2024; **24**(4): 40. 122. Chiduo M, Theilgaard ZP, Bakari V, et al. Prevalence of sexually transmitted infections among women attending antenatal clinics in Tanga, north eastern Tanzania. *Int J STD AIDS* 2012; **23**(5): 325-9. 123. Chigbu LN, Aluka C, Eke RA. Trichomoniasis as an indicator for existing sexually transmitted infections in women in Aba, Nigeria. *Annals of African Medicine* 2006; **5**(1): 1-5. 124. Chikwari CD, Bandason T, Beale M, et al. Co-prevalent sexually transmitted infections among individuals presenting with genital ulcer disease in Zimbabwe. *Sexually Transmitted Diseases* 2024; **51**(1 Supplement 1): S343-S4. 125. Chikwari CD, Simms V, Kranzer K, et al. Evaluation of a community-based aetiological approach for sexually transmitted infections management for youth in Zimbabwe: intervention findings from the STICH cluster randomised trial. *eClinicalMedicine* 2023; **62**: 102125. 126. Chikwem JO, Mohammed I, Bwala HG, Ola TO. Human immunodeficiency virus (HIV) infection in patients attending a sexually transmitted diseases clinic in Borno State of Nigeria. *Trop Geogr Med* 1990; **42**(1): 17-21. 127. Chirenje ZM, Dhibi N, Handsfield HH, et al. The Etiology of Vaginal Discharge Syndrome in Zimbabwe: Results from the Zimbabwe STI Etiology Study. *Sexually Transmitted Diseases* 2018; **45**(6): 422-8. 128. Chirenje ZM, Gundacker HM, Richardson B, et al. Risk Factors for Incidence of Sexually Transmitted Infections among Women in a Human Immunodeficiency Virus Chemoprevention Trial: VOICE (MTN-003). *Sexually Transmitted Diseases* 2017; **44**(3): 135-40. 129. Chitneni P, Bwana MB, Muyindike W, et al. STI prevalence among men living with HIV engaged in safer conception care in rural, southwestern Uganda. *PLoS ONE* 2021; **16**(3 March). 130. Chitneni P, Bwana MB, Owembabazi M, et al. Sexually Transmitted Infection Prevalence among Women at Risk for HIV Exposure Initiating Safer Conception Care in Rural, Southwestern Uganda. *Sexually Transmitted Diseases* 2020; **47**(8): E24-E8. 131. Cinman AC, Matos RASD, Blerk PJPV. Urethral Strictures at Baragwanath Hospital. *Br J Urol* 1980; **52**(5): 386-9. 132. Clemetson DB, Moss GB, Willerford DM, et al. Detection of HIV DNA in cervical and vaginal secretions: prevalence and correlates among women in Nairobi, Kenya. *Jama* 1993; **269**(22): 2860-4. 133. Clift S, Anemona A, Watson-Jones D, et al. Variations of HIV and STI prevalences within communities neighbouring new goldmines in Tanzania: Importance for intervention design. *Sexually Transmitted Infections* 2003; **79**(4): 307-12. 134. Climentine MR, Kuonza L, Kufa-Chakezha T. Predictors of persistent and recurrent genital sti symptoms at sentinel surveillance sites in South Africa, January 2015-june 2016. *Sexually Transmitted Infections* 2017; **93 (Supplement 2)**: A1. 135. Cohen CR, Koochesfahani KM, Meier AS, et al. Immunoepidemiologic profile of Chlamydia trachomatis infection: Importance of heat-shock protein 60 and interferon-γ. *Journal of Infectious Diseases* 2005b; **192**(4): 591-9. 136. Cohen CR, Mugo NR, Astete SG, et al. Detection of Mycoplasma genitalium in women with laparoscopically diagnosed acute salpingitis. *Sexually Transmitted Infections* 2005a; **81**(6): 463-6. 137. Cohen CR, Plummer FA, Mugo N, et al. Increased interleukin-10 in the endocervical secretions of women with non-ulcerative sexually transmitted diseases: A mechanism for enhanced HIV-1 transmission? *AIDS* 1999; **13**(3): 327 EP - 32. 138. Cohen CR, Sinei S, Reilly M, et al. Effect of human immunodeficiency virus type 1 infection upon acute salpingitis: a laparoscopic study. *J Infect Dis* 1998; **178**(5): 1352-8. 139. Cohen CR, Sinei SS, Bukusi EA, Bwayo JJ, Holmes KK, Brunham RC. Human leukocyte antigen class II DQ alleles associated with Chlamydia trachomatis tubal infertility. *Obstet Gynecol* 2000; **95**(1): 72-7. 140. Cohen MS, Hoffman IF, Royce RA, et al. Reduction of concentration of HIV-1 in semen after treatment of urethritis: implications for prevention of sexual transmission of HIV-1. AIDSCAP Malawi Research Group. *Lancet* 1997; **349**(9069): 1868-73. 141. Collet M, Reniers J, Frost E, et al. Infertility in Central Africa: Infection is the cause. *International Journal of Gynecology and Obstetrics* 1988; **26**(3): 423-8. 142. Colvin M, Abdool Karim SS, Connolly C, Hoosen AA, Ntuli N. HIV infection and asymptomatic sexually transmitted infections in a rural South African community. *Int J STD AIDS* 1998; **9**(9): 548-50. 143. Colvin M, Sharp B. Sexually transmitted infections and HIV in a rural community in the Lesotho highlands. *Sexually Transmitted Infections* 2000; **76**(1): 39-42. 144. Combe P, La Ruche G, Bonard D, et al. Hepatitis B and C infections, human immunodeficiency virus and other sexually transmitted infections among women of childbearing age in Cote d'Ivoire, West Africa. *Transactions of the Royal Society of Tropical Medicine and Hygiene* 2001; **95**(5): 493-6. 145. Compain F, Nodjikouambaye ZA, Sadjoli D, et al. Low prevalence of common sexually transmitted infections contrasting with high prevalence of mycoplasma asymptomatic genital carriage: A community-based cross-sectional survey in adult women living in N'Djamena, Chad. *Open Microbiology Journal* 2019; **13**(1): 222-9. 146. Congo-Ouedraogo M, Poncin T, Sangaré L, et al. Genomic and antimicrobial resistance analyses of *Neisseria gonorrhoeae* isolates, Burkina Faso, 2018–2019. *J Eur Acad Dermatol Venereol* 2022; **36**(7): e565-e8. 147. Connolly S, Wall KM, Parker R, et al. Sociodemographic factors and STIs associated with Chlamydia trachomatis and *Neisseria gonorrhoeae* infections in Zambian female sex workers and single mothers. *International Journal of STD and AIDS* 2020a; **31**(4): 364-74. 148. Coovadia Y, Dada, MA, Kharsany, A., Ramsaroop, U., Bhamjee A. The emergence of penicillinase-producing strains of *Neisseria gonorrhoeae* in Durban. *South African Medical Journal* 1984; **65**(21): 835-7. 149. Coovadia YM, Kharsany A, Hoosen A. The microbial aetiology of genital ulcers in black men in Durban, South Africa. *Genitourinary Medicine* 1985; **61**(4): 266-9. 150. Cowan FM, Hargrove JW, Langhaug LF, et al. The appropriateness of core group interventions using presumptive periodic treatment among rural Zimbabwean women who exchange sex for gifts or money. *Journal of Acquired Immune Deficiency Syndromes* 2005a; **38**(2): 202-7. 151. Cowan FM, Langhaug LF, Hargrove JW, et al. Is sexual contact with sex workers important in driving the HIV epidemic among men in rural Zimbabwe? *J Acquir Immune Defic Syndr* 2005b; **40**(3): 371-6. 152. Cowan FM, Langhaug LF, Mashungupa GP, et al. School based HIV prevention in Zimbabwe: Feasibility and acceptability of evaluation trials using biological outcomes. *AIDS* 2002; **16**(12): 1673-8. 153. Cowley G, Milne G, Teixeira da Silva E, et al. Prevalence of and risk factors for curable sexually transmitted infections on Bubaque Island, Guinea Bissau. *Sex Transm Infect* 2021; **97**(1): 51-5. 154. Crewe-Brown HH, Mahomed MF, Pochee E, Shewan KA, Adams A, Ebrahim O. Penicillinase producing strains of *Neisseria gonorrhoeae* in Pretoria. *South African medical journal = Suid-Afrikaanse tydskrif vir geneeskunde* 1985; **67**(5): 159. 155. Crocchiolo P, Lencioni R, Esposito R. The problem of the sexually transmitted diseases in African developing countries: A study in Zimbabwe. [Italian]. *Giornale di Malattie Infettive e Parassitarie* 1983; **35**(5): 483-8. 156. Cruickshank JG. The gonococcus and penicillin in Rhodesia. *Cent Afr J Med* 1976; **22**(11): 215-6. 157. Cuylaerts V, De Baetselier I, Muvunyi CM, et al. Implementation and evaluation of the Presto combined qualitative real-time assay for Chlamydia trachomatis and *Neisseria gonorrhoeae* in Rwanda. *African Journal of Laboratory Medicine* 2019; **8**(1). 158. D'Amico M, Mbah JCE, Gupta K, et al. Chlamydia trachomatis -Specific Antibody Responses in Women in Cameroon With Secondary Infertility. *Sexually Transmitted Diseases* 2023; **50**(11): E30-E3. 159. D'Costa LJ, Plummer FA, Bowmer I, et al. Prostitutes are a major reservoir of sexually transmitted diseases in Nairobi, Kenya. *Sex Transm Dis* 1985; **12**(2): 64-7. 160. Dada-Adegbola HO, Oni AA. Review of cases of children with gonorrhoea--source of infection. *Afr J Med Med Sci* 2001; **30**(4): 347-51. 161. Dagnra AY, David M. [Frequency and susceptibility to antibiotics of *Neisseria gonorrhoeae* at Lomé]. *Sante* 1999; **9**(5): 332-4. 162. Daly CC, Maggwa N, Mati JK, et al. Risk factors for gonorrhoea, syphilis, and trichomonas infections among women attending family planning clinics in Nairobi, Kenya. *Genitourin Med* 1994; **70**(3): 155-61. 163. Daly CC, Wangel AM, Hoffman IF, et al. Validation of the WHO diagnostic algorithm and development of an alternative scoring system for the management of women presenting with vaginal discharge in Malawi. *Sex Transm Infect* 1998; **74 Suppl 1**: S50-8. 164. Damiba AE, Vermund SH, Kelley KF. Prevalence of gonorrhoea, syphilis and trichomoniasis in prostitutes in Burkina Faso. *East Afr Med J* 1990; **67**(7): 473-7. 165. Daramola T, Oyediran MA. Venereal diseases in Lagos. *Isr J Med Sci* 1971; **7**(2): 288-94. 166. Davi SD, Okwu DG, Luetgehetmann M, et al. Epidemiology of co-infections in pregnant women living with human immunodeficiency virus 1 in rural Gabon: a cross-sectional study. *Infectious Diseases of Poverty* 2023; **12**(1): 64. 167. Davis MJ. Urethritis and prostatitis in an industry. *S Afr Med J* 1965; **39**(43): 1101-5. 168. De Baetselier I, Vuylsteke B, Yaya I, et al. To pool or not to pool samples for sexually transmitted infections detection in men who have sex with men? An evaluation of a new pooling method using the genexpert instrument in West Africa. *Sexually Transmitted Diseases* 2020; **47**(8): 556-61. 169. De Jongh M, Dangor Y, Adam A, Hoosen AA. Gonococcal resistance: Evolving from penicillin, tetracycline to the quinolones in South Africa - Implications for treatment guidelines. *International Journal of STD and AIDS* 2007; **18**(10): 697-9. 170. De Jongh M, Lekalakala MR, Le Roux M, Hoosen AA. Risk of having a sexually transmitted infection in women presenting at a termination of pregnancy clinic in Pretoria, South Africa. *J Obstet Gynaecol* 2010; **30**(5): 480-3. 171. De Schampheleire I, Van de Velden L, Meheus A. [Strategies for treatment of gonorrhea at the primary gynecology office in Pikine, Senegal]. *Ann Soc Belg Med Trop* 1984; **64**(2): 191-7. 172. De Villiers FPR, Prentice MA, Bergh AM, Miller SD. Sexually transmitted disease surveillance in a child abuse clinic. *South African Medical Journal* 1992; **81**(2): 84-6. 173. de Vos L, Mdingi MMM, Gigi RMS, Gebengu A, Peters RPH. Spectrum of sexual partner types among adults screened for sexually transmitted infections in the Eastern Cape, South Africa. *PLoS ONE* 2025; **20**(5): e0323414. 174. De Voux A, Mvududu R, Happel A, et al. Point-of-Care Sexually Transmitted Infection Testing Improves HIV Preexposure Prophylaxis Initiation in Pregnant Women in Antenatal Care in Cape Town, South Africa, 2019 to 2021. *Sexually Transmitted Diseases* 2023a; **50**(2): 92-7. 175. de Voux A, Nyemba DC, Silliman M, et al. Point-of-care testing for sexually transmitted infections and HIV pre-exposure prophylaxis among pregnant women in South Africa, 2021-2022: randomised controlled trial. *Sexually Transmitted Infections* 2023b; **100**(2): 77-83. 176. de Waaij DJ, Dubbink JH, Peters RP, Ouburg S, Morré SA. Comparison of GMT presto assay and Roche cobas® 4800 CT/NG assay for detection of Chlamydia trachomatis and Neisseria gonorrhoeae in dry swabs. *Journal of microbiological methods* 2015; **118**: 70-4. 177. De Walque D, Dow WH, Nathan R, et al. Incentivising safe sex: A randomised trial of conditional cash transfers for HIV and sexually transmitted infection prevention in rural Tanzania. *BMJ Open* 2012; **2**(1). 178. Deceuninck G, Asamoah-Adu C, Khonde N, et al. Improvement of clinical algorithms for the diagnosis of Neisseria gonorrhoeae and Chlamydia trachomatis by the use of Gram-stained smears among female sex workers in Accra, Ghana. *Sex Transm Dis* 2000; **27**(7): 401-10. 179. Deese J, Philip N, Lind M, et al. Sexually transmitted infections among women randomised to depot medroxyprogesterone acetate, a copper intrauterine device or a levonorgestrel implant. *Sexually Transmitted Infections* 2021; **97**(4): 249-55. 180. Deklerk E, Anderson R, Geffen C. THE ENZYME-LINKED IMMUNOSORBENT-ASSAY (ELISA) IN THE LABORATORY DIAGNOSIS OF GONORRHEA - A COMPARATIVE-EVALUATION. *SOUTH AFRICAN MEDICAL JOURNAL* 1983; **64**(12): 451-4. 181. Dela H, Attram N, Behene E, et al. Risk factors associated with gonorrhea and chlamydia transmission in selected health facilities in Ghana. *BMC Infect Dis* 2019; **19**(1): 425. 182. Delacollette C, Kihemu K, Delacollettelebrun C, Habyambere R. PREVALENCE AND ANTIBIOTIC-SENSITIVITY OF NEISSERIA-GONORRHOEAE INFECTION IN A RURAL AREA IN KIVU, ZAIRE. *ANNALES DE LA SOCIETE BELGE DE MEDECINE TROPICALE* 1986; **66**(1): 87-90. 183. Delany-Moretlwe S, Hughes J, Bock P, et al. Long acting injectable cabotegravir is safe and effective in preventing HIV infection in cisgender women: interim results from HPTN 084. *J Int AIDS Soc* 2021; **24 Suppl 1**(Suppl 1): e25659. 184. Delany-Moretlwe S, Mgodi N, Bekker LG, et al. High prevalence and incidence of gonorrhoea and chlamydia in young women eligible for HIV pre-exposure prophylaxis in South Africa and Zimbabwe: Results from the HPTN 082 trial. *Sexually Transmitted Infections* 2023; **99**(7): 433-9. 185. Demba E, Morison L, van der Loeff MS, et al. Bacterial vaginosis, vaginal flora patterns and vaginal hygiene practices in patients presenting with vaginal discharge syndrome in The Gambia, West Africa. *BMC Infect Dis* 2005; **5**: 12. 186. Demissie E, Amare A, Birhanu M, Gizachew M. Neisseria gonorrhoeae antimicrobial resistance patterns and associated risk factors in women of childbearing potential in northwestern Ethiopia. *BMC Women's Health* 2024; **24**(1): 82. 187. Demuylder X, Laga M, Tennstedt C, Vandyck E, Aelbers GNM, Piot P. THE ROLE OF NEISSERIA-GONORRHOEAE AND CHLAMYDIA-TRACHOMATIS IN PELVIC INFLAMMATORY DISEASE AND ITS SEQUELAE IN ZIMBABWE. *JOURNAL OF INFECTIOUS DISEASES* 1990; **162**(2): 501-5. 188. Denny L, Kuhn L, Pollack A, Wright TC. Direct visual inspection for cervical cancer screening - An analysis of factors influencing test performance. *CANCER* 2002; **94**(6): 1699-707. 189. Dery S, Guure C, Afagbedzi S, et al. Biobehavioral survey using time location sampling among female sex workers living in Ghana in 2020. *Frontiers in public health* 2024; **12**: 1137799. 190. Desai PJ, Morrison JA, Fleming AF. Penicillinase-producing Neisseria gonorrhoeae in Ndola, Zambia. *Trans R Soc Trop Med Hyg* 1990; **84**(1): 131. 191. Detels R, Green AM, Klausner JD, et al. The incidence and correlates of symptomatic and asymptomatic chlamydia trachomatis and neisseria gonorrhoeae infections in selected populations in five countries. *Sexually Transmitted Diseases* 2011; **38**(6): 503-9. 192. Deutschmann S, Bohne W, Mujuni F, et al. Low prevalence of Neisseria gonorrhoeae infections and no evidence of resistance against third generation cephalosporins in a cohort of HIV positive patients from a tertiary hospital in Tanzania. *Tropical Medicine and International Health* 2015; **20**: 287. 193. Dhont N, Muvunyi C, Luchters S, et al. HIV infection and sexual behaviour in primary and secondary infertile relationships: A case - Control study in Kigali, Rwanda. *Sexually Transmitted Infections* 2011; **87**(1): 28-34. 194. Diabate S, Behanzin L, Guedou FA, et al. Pre-exposure prophylaxis in real life: experience from a prospective, observational and demonstration project among men who have sex with men in Benin, West Africa. *Journal of the International AIDS Society* 2023; **26**(6): e26130. 195. Diallo AB, Mbengue AS, Camara M, et al. Bacterial vaginosis in Dakar (Senegal) in 2015. *American Journal of Tropical Medicine and Hygiene* 2016; **95 (5 Supplement 1)**: 331. 196. Diallo AS, Ngom M, Mbacké Daffe SM, et al. [Contribution of qPCR to the diagnosis of cervico-vaginal infections at the Hôpital Principal de Dakar, Senegal]. *Med Trop Sante Int* 2024; **4**(1). 197. Diallo MO, Ettiègne-Traoré V, Maran M, et al. Sexually transmitted diseases and human immunodeficiency virus infections in women attending an antenatal clinic in Abidjan, Côte d'Ivoire. *Int J STD AIDS* 1997; **8**(10): 636-8. 198. Dias BDC, Sekgele W, Nhlapo D, et al. Extragenital Sexually Transmitted Infections among High-Risk Men Who Have Sex with Men in Johannesburg, South Africa. *Sexually Transmitted Diseases* 2024; **51**(4): 245-50. 199. Dietrich M, Hoosen AA, Moodley J, Moodley S. Urogenital tract infections in pregnancy at King Edward VIII Hospital, Durban, South Africa. *Genitourin Med* 1992; **68**(1): 39-41. 200. Dieye AM, Samb NG, Ba A, et al. Effectiveness of syndromic approach for management of urethral discharge in Senegal. [French]. *Medecine tropicale : revue du Corps de sante colonial* 2003; **63**(1): 45-8. 201. Dionne JA, Anchang-Kimbi J, Hao J, et al. Trimethoprim-Sulfamethoxazole Plus Azithromycin to Prevent Malaria and Sexually Transmitted Infections in Pregnant Women With HIV (PREMISE): A Randomized, Double-Masked, Placebo-Controlled, Phase IIB Clinical Trial. *Open Forum Infectious Diseases* 2024; **11**(5): ofae274. 202. Ditsele RMM, Le Roux M, Matebane K, Nchabeleng M, Monokoane S. Bacterial vaginosis and vaginal microorganisms in pregnant women with a history of adverse pregnancy outcomes at Dr George Mukhari Academic Hospital, Pretoria, South Africa. *International Journal of Infectious Diseases* 2014; **21**: 426. 203. Dize L, West SK, Mkocha H, Quinn TC, Gaydos CA. Evaluation of pooled ocular and vaginal swabs by the Cepheid GeneXpert CT/NG assay for the detection of Chlamydia trachomatis and Neisseria gonorrhoeae compared to the GenProbe Aptima Combo 2 Assay. *Diagn Microbiol Infect Dis* 2015; **81**(2): 102-4. 204. Djomand G, Schlefer M, Gutreuter S, et al. Prevalence and Correlates of Genital Infections Among Newly Diagnosed Human Immunodeficiency Virus-Infected Adults Entering Human Immunodeficiency Virus Care in Windhoek, Namibia. *Sex Transm Dis* 2016; **43**(11): 698-705. 205. Donders GG, Donders F, Bellen G, et al. Screening for abnormal vaginal microflora by self-assessed vaginal pH does not enable detection of sexually transmitted infections in Ugandan women. *Diagn Microbiol Infect Dis* 2016; **85**(2): 227-30. 206. Donders GG, van Gerven V, de Wet HG, van Straten AM, de Boer F. Rapid antigen tests for Neisseria gonorrhoeae and Chlamydia trachomatis are not accurate for screening women with disturbed vaginal lactobacillary flora. *Scand J Infect Dis* 1996; **28**(6): 559-62. 207. Dosso M, Faye H, Diakite-Harding Y. Epidemiological aspects and prevalence of Neisseria gonorrhoea in genital infections in Abidjan: Analysis of 1,742 swabs. *BULL SOC PATHOL EXOT FIL* 1986; **79**(1): 130-9. 208. Downs JA, Van Dam GJ, Changalucha JM, et al. Association of schistosomiasis and HIV infection in Tanzania. *American Journal of Tropical Medicine and Hygiene* 2012; **87**(5): 868-73. 209. Doyle AM, Ross DA, Maganja K, et al. Long-term biological and behavioural impact of an adolescent sexual health intervention in tanzania: Follow-up survey of the community-based mema kwa vijana trial. *PLoS Medicine* 2010; **7**(6). 210. Drake AL, Kinuthia J, Matemo D, McClelland RS, Unger J, John-Stewart G. Prevalence and cofactors for STIs among pregnant adolescents in Western Kenya. *Sexually Transmitted Infections Conference: STI and AIDS World Congress* 2013; **89**(SUPPL. 1). 211. Duba TC, Peters RPH, Ehlers MM, Pruis N, Majola SM, Kock M. Detection of genital mycoplasmas in women visiting the infertility clinic of an academic hospital, pretoria, South Africa. *Sexually Transmitted Infections* 2017; **93 (Supplement 2)**: A64. 212. Dunaiski CM, Kock MM, Jung H, Peters RPH. Importance of Candida infection and fluconazole resistance in women with vaginal discharge syndrome in Namibia. *Antimicrobial Resistance and Infection Control* 2022; **11**(1): 104. 213. Dunaiski CM, Kock MM, Jung H, Peters RPH. Prospective Cohort Study of Treatment Outcomes of Vaginal Discharge Syndrome in Women in Windhoek, Namibia. *Sexually Transmitted Diseases* 2024; **51**(7): 460-5. 214. Duncan ME, Tibaux G, Pelzer A, et al. First coitus before menarche and risk of sexually transmitted disease. *Lancet* 1990; **335**(8685): 338-40. 215. Duncan S, Thiong'o AN, Macharia M, et al. High prevalence of quinolone resistance in Neisseria gonorrhoeae in coastal Kenya. *Sex Transm Infect* 2011; **87**(3): 231. 216. Dunkle KL, Beksinska ME, Rees VH, Ballard RC, Htun Y, Wilson ML. Risk factors for HIV infection among sex workers in Johannesburg, South Africa. *Int J STD AIDS* 2005; **16**(3): 256-61. 217. Duplessis C, Puplampu N, Nyarko E, et al. Gonorrhea surveillance in Ghana, Africa. *Mil Med* 2015; **180**(1): 17-22. 218. Dylewski J, D’Costa LJ, Nsanze H, Ronald AR. Single-dose therapy with trimethoprim-sulfametrole for chancroid in females. *Sexually Transmitted Diseases* 1986; **13**(3): 166-8. 219. Dziva Chikwari C, Dauya E, Simms V, et al. Effect of a community-based intervention for sexually transmitted infections on population-level prevalence among youth in Zimbabwe (STICH): a cluster-randomised trial. *Lancet Glob Health* 2025; **13**(1): e134-e45. 220. Dziva Chikwari C, Nzvere FP, Simms V, et al. Sexually transmitted infections screening and management for adolescents as part of a community-based comprehensive health check-up in Zimbabwe (Y-Check). *Sexual Health* 2024; **24**(4): 33 EP - 4. 221. Ebhodaghe BI, Ako-Nai KA, Aderoba AK. Evaluation of risk factors in MTCT among HIV-seropositive pregnant women in selected centers in Akure, South Western Nigeria. *Annals of Tropical Medicine and Public Health* 2017; **10**(1): 165 EP - 81. 222. Efosa OB, Uwadiegwu AP. Cytopathological Examination and Epidemiological Study of Cervicitis in Commercial Sex Workers (CSWs) in Coal City (Enugu), Nigeria. *Ethiop J Health Sci* 2015; **25**(3): 225-30. 223. Egere JU, Mbonu OO, Okaru J. Cefotaxime (Claforan) in the treatment of gonococcal urethritis in Nigeria. *Clinical Trials Journal* 1982; **19**(3): 162-9. 224. Ehinmidu JO, Bolaji RO, Adegboye EEA. Isolation and antibiotic susceptibility profile of Neisseria gonorrhoeae isolated from urine samples in Zaria, northern Nigeria. *Journal of Phytomedicine and Therapeutics* 2006; **8-11**: 20-4. 225. Ekabua JE, Ekabua KJ, Ekanem EI, Iklaki CU. Is the process of diagnosing and treating incidental medical findings a barrier to contraceptive acceptance and use. *Journal of Obstetrics and Gynaecology* 2009; **29**(3): 237 EP - 9. 226. Ekwempu CC, Lawande RV, Egler LJ. Microbial flora of the lower genital tract of women in labour in Zaria, Nigeria. *J Clin Pathol* 1981; **34**(1): 82-3. 227. Ekweozor CC, Olaleye OD, Tomori O, Saliu I, Essien EM. Sexually transmitted diseases in Ibadan in the 1990's: HIV infection--an additional dimension. *Afr J Med Med Sci* 1994; **23**(4): 363-7. 228. Ekwere PD, Etuk EH. Semen quality among subfertile males following treatment with ofloxacin. *CURR THER RES CLIN EXP* 1991; **50**(3): 425-32. 229. Elegbe IA, Elegbe I, Fabiyi OC, Oshoba M. Causes of vaginal infections in Nigeria. *J R Soc Health* 1986; **106**(6): 222-3. 230. Elias Dah TT. The management of syphilis and gonococcal infections among men who have sex with men (MSM) in a community medical center named "centre oasis, ouagadougou, burkina faso". *Sexually Transmitted Infections* 2011; **87**: A329. 231. Elliott B, Brunham RC, Laga M, et al. Maternal gonococcal infection as a preventable risk factor for low birth weight. *Journal of infectious diseases* 1990; **161**(3): 531-6. 232. Emele FE, Anyiwo CE. Prevalence and horizontal propagation of gonococcal infections among Nigerian children. *Acta Paediatr* 1998; **87**(12): 1295-6. 233. Fakunle YM, Watkins B. Influence of self-medication on prevalence and antibiotic sensitivity of N. gonorrhoeae in Zaria (Nigeria). *East Afr Med J* 1976; **53**(12): 693-6. 234. Fatiregun AA, Afolabi Bamgboye E. Sexually Transmitted Diseases seen in a Nigerian tertiary institution. *West African Journal of Medicine* 2004; **23**(3): 236-9. 235. Favot I, Ngalula J, Mgalla Z, Klokke AH, Gumodoka B, Boerma JT. HIV infection and sexual behaviour among women with infertility in Tanzania: a hospital-based study. *International journal of epidemiology* 1997; **26**(2): 414-9. 236. Fawole OI, Asuzu MC. Where have all the STDs gone? *Afr J Med Med Sci* 1998; **27**(3-4): 193-5. 237. Faye-Kette Achi YH, Sylla-Koko DF, N'Douba-Kacou A, et al. Vulvo vaginitis on girls in a tropical city: Bacteriological aspects. [French]. *Medecine et Maladies Infectieuses* 1993a; **23**(12): 934-6. 238. Faye-Kette YH, Kouassi AA, Sylla-Koko DF, et al. [Prevalence of 4 agents of sexually transmitted diseases in leukorrhea in Abidjan (Ivory Coast)]. *Bull Soc Pathol Exot* 1993b; **86**(4): 245-7; discussion 7. 239. Fayemiwo SA, Fatiregun AA, Bakare RA. Pattern of sexually transmitted infections (STIS) in hormonal contraceptives and intrauterine devices (IUD) users attending family planning clinics in Ibadan, Nigeria. *Sexually Transmitted Infections* 2011; **87**: A175-A6. 240. Fayemiwo SA, Novak-Frazer L, Adewole IF, Rautemaa-Richardson R. Epidemiology of bacterial genitourinary infections among women of reproductive age in Nigeria. *Sexual Health* 2024; **24**(4): 82. 241. Feldblum PJ, Kuyoh M, Omari M, Ryan KA, Bwayo JJ, Welsh M. Baseline STD prevalence in a community intervention trial of the female condom in Kenya. *Sex Transm Infect* 2000; **76**(6): 454-6. 242. Feldblum PJ, Lie CC, Weaver MA, et al. Baseline factors associated with incident HIV and STI in four microbicide trials. *Sex Transm Dis* 2010; **37**(10): 594-601. 243. Fentaw S, Abubeker R, Asamene N, Assefa M, Bekele Y, Tigabu E. Antimicrobial susceptibility profile of Gonococcal isolates obtained from men presenting with urethral discharge in Addis Ababa, Ethiopia: Implications for national syndromic treatment guideline. *PLoS ONE* 2020; **15**(6). 244. Ferré VM, Ekouevi DK, Gbeasor-Komlanvi FA, et al. Prevalence of human papillomavirus, human immunodeficiency virus and other sexually transmitted infections among female sex workers in Togo: a national cross-sectional survey. *Clin Microbiol Infect* 2019b; **25**(12): 1560.e1-.e7. 245. Ferré VM, Gbeasor-Komlanvi FA, Collin G, et al. Prevalence of Human Papillomavirus, Human Immunodeficiency Virus, and Other Sexually Transmitted Infections Among Men Who Have Sex With Men in Togo: A National Cross-sectional Survey. *Clin Infect Dis* 2019a; **69**(6): 1019-26. 246. Ferre VM, Sadio A, Gbeasor-Komlanvi DF, et al. HIGH PREVALENCE OF HPV, OTHER STI, AND ANAL LESIONS AMONG MSM IN TOGO. *Topics in Antiviral Medicine* 2023; **31**(2): 412. 247. Ferreira-Marques J. [CONTRIBUTION TO THE STUDY OF VENEREAL AND CUTANEOUS DISEASES AT ADDIS-ABABA]. *Dermatol Trop Ecol Geogr* 1964; **30**: 139-51. 248. Finlayson MH, Gibbs B, Brede HD. Diagnosis and incidence of Neisseria gonorrhoeae in Cape coloured females in the Western Cape. Laboratory aspects. *S Afr Med J* 1974a; **48**(7): 259-60. 249. Finlayson MH, Willey KFD, Brede HD, Wilson AJ. A note on the use of reduced transport fluid (RTF) for isolation of Neisseria gonorrhoeae. *South African Medical Journal* 1974b; **47**(28): 1195-6. 250. Folgosa E, Gonzalez C, Osman NB, Hägerstrand I, Bergström S, Ljungh Å. A case control study of chorioamniotic infection and histological chorioamnionitis in stillbirth. *APMIS* 1997; **105**(4): 329-36. 251. Fonck K, Kidula N, Jaoko W, et al. Validity of the vaginal discharge algorithm among pregnant and non-pregnant women in Nairobi, Kenya. *Sex Transm Infect* 2000a; **76**(1): 33-8. 252. Fonck K, Kidula N, Kirui P, et al. Pattern of sexually transmitted diseases and risk factors among women attending an STD referral clinic in Nairobi, Kenya. *Sex Transm Dis* 2000b; **27**(7): 417-23. 253. Fonck K, Mwai C, Ndinya-Achola J, Bwayo J, Temmerman M. Health-seeking and sexual behaviors among primary healthcare patients in Nairobi, Kenya. *Sex Transm Dis* 2002; **29**(2): 106-11. 254. Fortas C, Harimanana AN, Rasoanandrianina SB, et al. Sexually transmitted infections and bacterial vaginosis in women of child-bearing age in Antananarivo, Madagascar: prevalence and risk factors from a cross-sectional study. *BMC Infect Dis* 2025; **25**(1): 262. 255. Franceschi S, Smith JS, Van Den Brule A, et al. Cervical infection with Chlamydia trachomatis and Neisseria gonorrhoeae in women from ten areas in four continents. *SEXUALLY TRANSMITTED DISEASES* 2007; **34**(8): 563-9. 256. Francis SC, Holm Hansen C, Irani J, et al. Results from a cross-sectional sexual and reproductive health study among school girls in Tanzania: High prevalence of bacterial vaginosis. *Sexually Transmitted Infections* 2019; **95**(3): 219-27. 257. Francis SC, Looker C, Vandepitte J, et al. Bacterial vaginosis among women at high risk for HIV in Uganda: High rate of recurrent diagnosis despite treatment. *Sexually Transmitted Infections* 2016; **92**(2): 142-8. 258. Francis SC, Mthiyane TN, Baisley K, et al. Prevalence of sexually transmitted infections among young people in South Africa: A nested survey in a health and demographic surveillance site. *PLoS Medicine* 2018; **15**(2). 259. FRANSEN L, NSANZE H, D'COSTA LJ, BRUNHAM RC, PIOT P. Parents of infants with ophthalmia neonatorum: a high-risk group for sexually transmitted diseases. *Sexually transmitted diseases* 1985; **12**(3): 150-4. 260. Frohlich JA, Abdool Karim Q, Mashego MM, Sturm AW, Abdool Karim SS. Opportunities for treating sexually transmitted infections and reducing HIV risk in rural South Africa. *J Adv Nurs* 2007; **60**(4): 377-83. 261. Frost E, Leclerc A, Gioanni G, Goeman J, Peeters M, Collet M. CHLAMYDIAE INFECT THE PLACENTA LESS OFTEN THAN GONOCOCCI. *GENITOURINARY MEDICINE* 1988; **64**(5): 349-50. 262. Fwambah L, Andisi C, Streatfield C, et al. Exposure to common infections may shape basal immunity and potentially HIV-1 acquisition amongst a high-risk population in Coastal Kenya. *Front Immunol* 2023; **14**. 263. Gadoth A, Mvumbi G, Hoff NA, et al. Urogenital Schistosomiasis and Sexually Transmitted Coinfections among Pregnant Women in a Schistosome-Endemic Region of the Democratic Republic of Congo. *Am J Trop Med Hyg* 2019; **101**(4): 828-36. 264. Galega FP, Heymann DL, Nasah BT. GONOCOCCAL OPHTHALMIA NEONATORUM - THE CASE FOR PROPHYLAXIS IN TROPICAL AFRICA. *BULLETIN OF THE WORLD HEALTH ORGANIZATION* 1984; **62**(1): 95-8. 265. Garrett NJ, Osman F, Maharaj B, et al. Beyond syndromic management: Opportunities for diagnosis-based treatment of sexually transmitted infections in low- and middle-income countries. *PLoS ONE* 2018; **13**(4). 266. Gateau T, Zeller HG. Epidemiological approach for sexually transmitted diseases in Antsiranana (north Madagascar). Between prevention and treatment, the choice of a strategy against sexually transmitted diseases. *Arch Inst Pasteur Madagascar* 1996; **63**(1-2): 8-11. 267. Geremew RA, Agizie BM, Bashaw AA, Seid ME, Yeshanew AG. Prevalence of Selected Sexually Transmitted Infection (STI) and Associated Factors among Symptomatic Patients Attending Gondar Town Hospitals and Health Centers. *Ethiop J Health Sci* 2017; **27**(6): 589-600. 268. Germain M, Alary M, Guedeme A, et al. Evaluation of a screening algorithm for the diagnosis of genital infections with Neisseria gonorrhoeae and Chlamydia trachomatis among female sex workers in Benin. *Sexually Transmitted Diseases* 1997; **24**(2): 109-15. 269. Ghys PD, Diallo MO, Ettiegne-Traore V, et al. Increase in condom use and decline in HIV and sexually transmitted diseases among female sex workers in Abidjan, Cote d'Ivoire, 1991-1998. *AIDS* 2002; **16**(2): 251-8. 270. Gibbs A, Healy K, Kaldhusdal V, et al. Preserved Mucosal-Associated Invariant T Cells in the Cervical Mucosa of HIV-Infected Women with Dominant Expression of the TRAV1-2-TRAJ20 T Cell Receptor α-Chain. Journal of Infectious Diseases; 2022: Oxford University Press; 2022. p. 1428-40. 271. Gichangi PB, Ndinya-Achola JO, Ombete J, Nagelkerke NJ, Temmerman M. Antimicrobial prophylaxis in pregnancy: a randomized, placebo-controlled trial with cefetamet-pivoxil in pregnant women with a poor obstetric history. *Am J Obstet Gynecol* 1997; **177**(3): 680-4. 272. Gichuhi S, Bosire R, Mbori-Ngacha D, et al. Risk factors for neonatal conjunctivitis in babies of HIV-1 infected mothers. *Ophthalmic Epidemiol* 2009; **16**(6): 337-45. 273. Gigi RMS, Babalola CM, Klausner JD, et al. Prevalence and incidence of Chlamydia trachomatis and Neisseria gonorrhoeae among pregnant women in a high HIV prevalence setting in South Africa. *Sexually Transmitted Diseases* 2024; **51**(1 Supplement 1): S280-S1. 274. Ginindza TG, Stefan CD, Tsoka-Gwegweni JM, et al. Prevalence and risk factors associated with sexually transmitted infections (STIs) among women of reproductive age in Swaziland. *Infectious Agents and Cancer* 2017; **12**(1). 275. Giuliano AR, Botha MH, Zeier M, et al. High HIV, HPV, and STI prevalence among young Western Cape, South African women: EVRI HIV prevention preparedness trial. *J Acquir Immune Defic Syndr* 2015; **68**(2): 227-35. 276. Gnatou GYS, Gbeasor-Komlanvi FA, Afanvi KA, et al. Prevalence of Chlamydia trachomatis, Neisseria gonorrhoeae, Treponema pallidum, and HIV among women in Kara, 2022. *Sante Publique* 2024; **36**(3): 137-46. 277. Gomes JP, Tavira L, Exposto F, Prieto E, Catry MA. Neisseria gonorrhoeae and Chlamydia trachomatis infections in patients attending STD and family planning clinics in Bissau, Guinea-Bissau. *Acta Trop* 2001; **80**(3): 261-4. 278. Gomih-Alakija A, Ting J, Mugo N, et al. Clinical characteristics associated with Mycoplasma genitalium among female sex workers in Nairobi, Kenya. *J Clin Microbiol* 2014; **52**(10): 3660-6. 279. Gomo E, Ndamba J, Nhandara C, Murahwa SZ, Nyazema NZ. Prevalence of gonorrhoea and knowledge of sexually transmitted infections in a farming community in Zimbabwe. *Cent Afr J Med* 1997; **43**(7): 192-5. 280. Gore-Langton GR, Ashorn U, Gutman JR, et al. PREVALENCE OF CURABLE SEXUALLY TRANSMITTED AND REPRODUCTIVE TRACT INFECTIONS AMONG PREGNANT WOMEN IN KENYA, MALAWI, AND TANZANIA, 2017-20. *American Journal of Tropical Medicine and Hygiene* 2023; **108**(4 Supplement): 566. 281. Govender L, Hoosen AA, Moodley J, Moodley P, Sturm AW. Bacterial vaginosis and associated infections in pregnancy. *Int J Gynaecol Obstet* 1996; **55**(1): 23-8. 282. Govender S, Lebani T, Nell R. Antibiotic susceptibility patterns of Neisseria gonorrhoeae isolates in Port Elizabeth. *S Afr Med J* 2006; **96**(3): 225-6. 283. Govender V, Moodley D, Naidoo M, Connoly C, Ngcapu S, Abdool Karim Q. High incidence of asymptomatic genital tract infections in pregnancy in adolescent girls and young women: Need for repeat aetiological screening. *Sexually Transmitted Infections* 2023; **99**(7): 482-8. 284. Govender V, Naidoo M, Moodley D. Sexually transmitted infections and bacterial vaginosis among adolescent girls and young women in the early postpartum period: a cross-sectional study. *BMC Infect Dis* 2024; **24**(1): 898. 285. Grabert BK, Islam JY, Kabare E, et al. Testing for Sexually Transmitted Infection Using Wet and Dry Self-Collected Brush Samples Among Women in Mombasa, Kenya. *Sexually Transmitted Diseases* 2022; **49**(9): E100-E3. 286. Grabowski MK, Mpagazi J, Kiboneka S, et al. The HIV and sexually transmitted infection syndemic following mass scale-up of combination HIV interventions in two communities in southern Uganda: a population-based cross-sectional study. *The Lancet Global Health* 2022; **10**(12): e1825-e34. 287. Gray CM, O’Hagan KL, Lorenzo-Redondo R, et al. Impact of chemokine C–C ligand 27, foreskin anatomy and sexually transmitted infections on HIV-1 target cell availability in adolescent South African males. *Mucosal Immunol* 2020; **13**(1): 118-27. 288. Gray R, Azire J, Serwadda D, et al. Male circumcision and the risk of sexually transmitted infections and HIV in Rakai, Uganda. *AIDS* 2004; **18**(18): 2428-30. 289. Grech ES, Everett JV, Mukasa F. Epidemiological aspects of acute pelvic inflammatory disease in Uganda. *Trop Doct* 1973; **3**(3): 123-7. 290. Green H, Taleghani S, Nyemba D, Myer L, Davey DJ. Partner notification and treatment for sexually transmitted infections among pregnant women in Cape Town, South Africa. *Int J STD AIDS* 2020; **31**(13): 1282-90. 291. Gregson S, Mason PR, Garnett GP, et al. A rural HIV epidemic in Zimbabwe? Findings from a population-based survey. *Int J STD AIDS* 2001; **12**(3): 189-96. 292. Grijsen ML, Graham SM, Mwangome M, et al. Screening for genital and anorectal sexually transmitted infections in HIV prevention trials in Africa. *Sex Transm Infect* 2008; **84**(5): 364-70. 293. Gueguen A, Pecarrere JL, Ribot JJ. [Neisseria gonorrhoeae in Madagascar. Antibiotic sensitivity--research of strains producing beta-lactamase]. *Arch Inst Pasteur Madagascar* 1980; **47**(1): 65-85. 294. Gueye Ndiaye A, Faye CM, Ndiaye I, et al. [Screening for HIV, syphilis, Chlamydia trachomatis and Neisseria gonorrhoreae during a combined survey conducted in Malicouna, a Senegalese rural area]. *Bull Soc Pathol Exot* 2009; **102**(3): 150-4. 295. Gueye SB, Diop-Ndiaye H, Gningue A, et al. Performance of the Abbott Real Time CT/NG assay in urines and cervico-vaginal samples from Senegal. *J Infect Dev Ctries* 2014; **8**(7): 898-903. 296. Guffey MB, Richardson B, Husnik M, et al. HPTN 035 phase II/IIb randomised safety and effectiveness study of the vaginal microbicides BufferGel and 0.5% PRO 2000 for the prevention of sexually transmitted infections in women. *Sex Transm Infect* 2014; **90**(5): 363-9. 297. Guimaraes H, Castro R, Tavira LT, Exposto FL. Assessing therapeutic management of vaginal and urethral symptoms in an anonymous HIV testing centre in Luanda, Angola. *Journal of Infection in Developing Countries* 2013; **7**(10): 720-5. 298. Gumede L, Kufa-Chakezha T, Maseko V, Kularatne R. Predictors of sexually transmitted coinfections in women presenting with bacterial vaginosis to primary healthcare facilities in South Africa. *Sexually Transmitted Infections* 2017a; **93 (Supplement 2)**: A143. 299. Gumede L, Radebe F, Nhlapo D, Maseko V, Kufa-Chakezha T, Kularatne R. Evaluation of the Copan eSwab, a liquid-based microbiology transport system, for the preservation of Neisseria gonorrhoeae at different temperatures. *Southern African Journal of Infectious Diseases* 2017b; **32**(3): 96-9. 300. Guyot A, Jarrett B, Sanvee L, Dore D. Antimicrobial resistance of Neisseria gonorrhoeae in Liberia. *Trans R Soc Trop Med Hyg* 1998; **92**(6): 670-4. 301. Habte-Gabr E, Geyid A, Serdo D, Biddle J, Perine PL. Single-dose treatment of uncomplicated acute gonococcal urethritis in Ethiopian men: comparison of rosoxacin, spectinomycin, penicillin, and ampicillin. *Sex Transm Dis* 1987; **14**(3): 153-5. 302. Hailemariam M, Abebe T, Mihret A, Lambiyo T. Prevalence of Neisseria gonorrhea and their antimicrobial susceptibility patterns among symptomatic women attending gynecology outpatient department in Hawassa referral hospital, Hawassa, Ethiopia. *Ethiop J Health Sci* 2013; **23**(1): 10-8. 303. Hailu K, Gebretsadik A. Determinants of gonorrhea and syphilis infections among pregnant women attending antenatal clinic at Dilla University Referral Hospital, Ethiopia: Unmatched case-control study. *Womens Health (Lond)* 2020; **16**: 1745506520940095. 304. Hall SM, Whitcomb MA. Screening for gonorrhoea in family planning acceptors in a developing community. *Public Health* 1978; **92**(3): 121-4. 305. Halpern V, Obunge O, Ogunsola F, et al. Interim data monitoring to enroll higher-risk participants in HIV prevention trials. *BMC Medical Research Methodology* 2009; **9**(1). 306. Hamill MM, Onzia A, Wang TH, et al. High burden of untreated syphilis, drug resistant Neisseria gonorrhoeae, and other sexually transmitted infections in men with urethral discharge syndrome in Kampala, Uganda. *BMC Infectious Diseases* 2022; **22**(1): 440. 307. Harijaona V, Ramambason JD, Morisset R, Rasamindrakotroka A, Ravaoarinoro M. Prevalence of and risk factors for sexually-transmitted infections in hidden female sex workers. *Med Mal Infect* 2009; **39**(12): 909-13. 308. Harms G, Iyambo SN, Corea A, Radebe F, Fehler HG, Ballard RC. Perceptions and patterns of reproductive tract infections in a young rural population in North-West Namibia. *Int J STD AIDS* 1998; **9**(12): 744-50. 309. Harms G, Matull R, Randrianasolo D, et al. Pattern of sexually transmitted diseases in a Malagasy population. *Sex Transm Dis* 1994; **21**(6): 315-20. 310. Harryparsad R, Meyer B, Taku O, et al. Prevalence and incidence of sexually transmitted infections among South African women initiating injectable and long-acting contraceptives. *PLoS ONE* 2023; **18**(11 November): e0294285. 311. Hawken MP, Melis RD, Ngombo DT, et al. Part time female sex workers in a suburban community in Kenya: a vulnerable hidden population. *Sex Transm Infect* 2002a; **78**(4): 271-3. 312. Hawken MP, Melis RD, Ngombo DT, et al. Opportunity for prevention of HIV and sexually transmitted infections in Kenyan youth: results of a population-based survey. *J Acquir Immune Defic Syndr* 2002b; **31**(5): 529-35. 313. Hazel A, Ponnaluri-Wears S, Davis GS, Low BS, Foxman B. High prevalence of Neisseria gonorrhoeae in a remote, undertreated population of Namibian pastoralists. *Epidemiol Infect* 2014; **142**(11): 2422-32. 314. Heffron R, Casmir E, Aswani L, et al. HIV risk and pre-exposure prophylaxis interest among women seeking post-abortion care in Kenya: a cross-sectional study. *J Int AIDS Soc* 2021; **24**(5): e25703. 315. Hellmann NS, Nsubuga PS, Bainganabaingi DJ, et al. SINGLE-DOSE AMPICILLIN-SULBACTAM VERSUS CEFTRIAXONE AS TREATMENT FOR UNCOMPLICATED GONORRHEA IN A UGANDAN STD CLINIC POPULATION WITH A HIGH PREVALENCE OF PPNG INFECTION. *JOURNAL OF TROPICAL MEDICINE AND HYGIENE* 1995; **98**(2): 95-100. 316. Hira SK, Feldblum PJ, Kamanga J, Mukelabai G, Weir SS, Weir JC. Condom and Nonoxynol-9 use and the incidence of HIV infection in serodiscordant couples in Zambia. *International Journal of STD & AIDS* 1997; **8**(4): 243-50. 317. Hira SK, Sheth J, Bhat S. Ophthalmia neonatorum in Zambia. *European Journal of Sexually Transmitted Diseases* 1986; **3**(2): 103-6. 318. Hira SK, Spruyt AB, Feldblum PJ, Sunkutu MR, Glover LH, Steiner MJ. Spermicide acceptability among patients at a sexually transmitted disease clinic in Zambia. *American Journal of Public Health* 1995; **85**(8): 1098-103. 319. Hladik W, Baughman AL, Serwadda D, et al. Burden and characteristics of HIV infection among female sex workers in Kampala, Uganda - a respondent-driven sampling survey. *BMC public health* 2017; **17**(1): 565. 320. Hoffman CM, Mbambazela N, Sithole P, et al. Provision of Sexually Transmitted Infection Services in a Mobile Clinic Reveals High Unmet Need in Remote Areas of South Africa: A Cross-sectional Study. *Sex Transm Dis* 2019; **46**(3): 206-12. 321. Hokororo A, Kihunrwa A, Hoekstra P, et al. High prevalence of sexually transmitted infections in pregnant adolescent girls in Tanzania: A multi-community cross-sectional study. *Sexually Transmitted Infections* 2015; **91**(7): 473-8. 322. Holali Ameyapoh A, Katawa G, Ritter M, et al. Hookworm Infections and Sociodemographic Factors Associated With Female Reproductive Tract Infections in Rural Areas of the Central Region of Togo. *Front Microbiol* 2021; **12**. 323. Homsy J, King R, Bannink F, et al. Primary HIV prevention in pregnant and lactating Ugandan women: A randomized trial. *PLoS ONE* 2019; **14**(2). 324. Hoosen AA, Abdul H, Moodley J, Sturm AW. Sexually transmitted infections in ambulatory patients with pelvic inflammatory disease. *Journal of Obstetrics and Gynaecology* 1996a; **16**(6): 544-7. 325. Hoosen AA, Kharsany ABM, Ison CA. Single low-dose ceftriaxone for the treatment of gonococcal ophthalmia - Implications for the national programme for the syndromic management of sexually transmitted diseases. *South African Medical Journal* 2002; **92**(3): 238-40. 326. Hoosen AA, Moodley J, Pudifin DJ, Duursma J, Coetzee KD, Kharsany ABM. Sexually transmitted pathogens and colposcopic findings in asymptomatic HIV-1 antibody-positive blood donors. *South African Medical Journal* 1990; **77**(12): 626-8. 327. Hoosen AA, Nteta C, Moodley J, Sturm AW. Sexually transmitted diseases including HIV infection in women with Bartholin's gland abscesses. *Genitourinary Medicine* 1995; **71**(3): 155-7. 328. Hoosen AA, Ntsaluba A, Moodley J, Moodley C, Sturm AW. Asymptomatic lower genital tract infections in women undergoing hysterosalpingography. *Journal of Obstetrics and Gynaecology* 1996b; **16**(5): 381-3. 329. Hoosen AA, O'Farrell N, van den Ende J. Microbiology of acute epididymitis in a developing community. *Genitourin Med* 1993; **69**(5): 361-3. 330. Hoosen AA, Quinlan DJ, Moodley J, Kharsany ABM, Vandenende J. SEXUALLY-TRANSMITTED PATHOGENS IN ACUTE PELVIC INFLAMMATORY DISEASE. *SOUTH AFRICAN MEDICAL JOURNAL* 1989; **76**(6): 251-4. 331. Hoosen AA, Ross SM, Mulla MJ, Patel M. The incidence of selected vaginal infections among pregnant urban blacks. *S Afr Med J* 1981; **59**(23): 827-9. 332. Hopcraft M, Verhagen AR, Ngigi S, Haga AC. Genital infections in developing countries: experience in a family planning clinic. *Bull World Health Organ* 1973; **48**(5): 581-6. 333. Hubacher D, Raymond ER, Beksinska M, et al. Hormonal contraception and the risks of STI acquisition: results of a feasibility study to plan a future randomized trial. *Contraception* 2008; **77**(5): 366-70. 334. Huber A. [Inflammatory diseases of the female genital organs in childhood]. *Wien Med Wochenschr* 1969; **119**(21): 385-91 concl. 335. Hurkchand H, Makuluma H, Molefe N, Molapo M. Measuring the impact of HIV and STIs in a community in a coal mining town, Mpumalanga, South Africa. *J S Afr Inst Min Metall* 2005; **105**(6): 365-8. 336. Hutton-Nyameaye AA, Asiamah M, Asafo-Adjei K, et al. Knowledge and prevalence of common sexually transmitted infections among patients seeking care at selected health facilities in Southern Ghana. *PLOS Glob Public Health* 2024; **4**(7). 337. Huyveneers LEP, Maphanga M, Umunnakwe CN, et al. Prevalence, incidence and recurrence of sexually transmitted infections in HIV-negative adult women in a rural South African setting. *Trop Med Int Health* 2023; **28**(4): 335-42. 338. Ibekwe VI, Ubochi KC, Anyanwu BN. The prevalence of penicillin resistance in organisms that cause sexually transmitted diseases in Port Harcourt, Nigeria. *International Journal of Environmental Health Research* 2000; **10**(3): 251-5. 339. Ibrahim K, Izebe KS, Yusufu AY, Abdulrahim ME, Oladosu P, Inyang US. Isolation and characterization of common pathogens isolated in vaginitis in selected hospitals in Abuja. *Journal of Phytomedicine and Therapeutics* 2003; **8-11**: 49-53. 340. Ihekwaba FN. Hazards of Urethral Instrumentation. *Tropical Doctor* 1979; **9**(3): 121-3. 341. Ihunwo OA, Ogbonna IC, Chukwuekezie JO. Isolation of Neisseria gonorrhoeae using urine as a holding medium. *East Afr Med J* 1998; **75**(10): 572-5. 342. Iquatt B, Sawhney AN. Symptomless gonorrhoea in women in Maiduguri (north eastern Nigeria). *Genitourin Med* 1988; **64**(5): 349. 343. Isara A, Baldeh AK. Prevalence of sexually transmitted infections among pregnant women attending antenatal clinics in west coast region of the gambia. *African Health Sciences* 2021; **21**(2): 585-92. 344. Jackson DJ, Rakwar JP, Chohan B, et al. Urethral infection in a workplace population of East African men: evaluation of strategies for screening and management. *J Infect Dis* 1997; **175**(4): 833-8. 345. Jacobs B, Mayaud P, Changalucha J, et al. Sexual transmission of hepatitis B in Mwanza, Tanzania. *Sex Transm Dis* 1997; **24**(3): 121-6. 346. Jansen HAFM, Morison L, Mosha F, et al. Geographical variations in the prevalence of HIV and other sexually transmitted infections in rural Tanzania. *International Journal of STD and AIDS* 2003; **14**(4): 274-80. 347. Jarolimova J, Chidumwa G, Chimbindi N, et al. Prevalence of Curable Sexually Transmitted Infections in a Population-Representative Sample of Young Adults in a High HIV Incidence Area in South Africa. *Sexually Transmitted Diseases* 2023; **50**(12): 796-803. 348. Jarolimova J, Yan J, Govere S, et al. Sexually transmitted infection testing integrated with HIV prevention and contraceptive services in hair salons in urban South Africa. *Journal of Acquired Immune Deficiency Syndromes (1999)* 2025. 349. Jarrett OD, Srinivasan S, Richardson BA, et al. Specific vaginal bacteria are associated with an increased risk of Trichomonas vaginalis acquisition in women. *Journal of Infectious Diseases* 2019; **220**(9): 1503-10. 350. Jary A, Teguete I, Sidibe Y, et al. Prevalence of cervical HPV infection, sexually transmitted infections and associated antimicrobial resistance in women attending cervical cancer screening in Mali. *International Journal of Infectious Diseases* 2021; **108**: 610-6. 351. Jespers V, Crucitti T, Menten J, et al. Prevalence and correlates of bacterial vaginosis in different sub-populations of women in sub-Saharan Africa: a cross-sectional study. *PLoS ONE* 2014; **9**(10): e109670. 352. Jewanraj J, Ngcapu S, Osman F, et al. The Impact of Semen Exposure on the Immune and Microbial Environments of the Female Genital Tract. *Front Reprod Health* 2020; **2**. 353. Jones HE, Altini L, de Kock A, Young T, van de Wijgert JH. Home-based versus clinic-based self-sampling and testing for sexually transmitted infections in Gugulethu, South Africa: randomised controlled trial. *Sex Transm Infect* 2007; **83**(7): 552-7. 354. Joseph Davey D, Fynn L, Rousseau E, et al. Evaluation of point-of-care diagnostics for sexually transmitted infection on oral PrEP initiation and persistence among young people in South Africa: a randomized controlled study. *J Int AIDS Soc* 2025; **28**(5): e26488. 355. Joshi RM, Lawande RV, Mathur DR. Sexually transmitted diseases in Zaria, Northern Nigeria. *European Journal of Sexually Transmitted Diseases* 1986; **3**(2): 99-102. 356. Juliana NCA, Deb S, Ouburg S, et al. The prevalence of chlamydia trachomatis and three other non-viral sexually transmitted infections among pregnant women in pemba island tanzania. *Pathogens* 2020; **9**(8): 1-12. 357. Kahn MJ, Dionne JA, Manga SM, Nulah KL, Van Der Pol B, Ye Y. Preliminary performance characteristics of the less expensive Atila iAMP-4STI NAAT compared to the Cepheid Xpert CT/NG for diagnosing genital CT & NG in women engaged in transactional sex in Cameroon. *Sexually Transmitted Diseases* 2024; **51**(1 Supplement 1): S210-S1. 358. Kahsay AG, Mezgebo TA, Gebrekidan GB, Desta BL, Mihretu HG, Dejene TA. Prevalence, Antibiotic Resistance and Associated Factors of Neisseria gonorrhoeae Among Patients Attending Non-Profitable Private Clinics in Mekelle, Tigrai, Ethiopia. *Infection and Drug Resistance* 2023; **16**: 4065-72. 359. Kaida A, Dietrich JJ, Laher F, et al. A high burden of asymptomatic genital tract infections undermines the syndromic management approach among adolescents and young adults in South Africa: implications for HIV prevention efforts. *BMC Infect Dis* 2018; **18**(1): 499. 360. Kakaire O, Byamugisha JK, Tumwesigye NM, Gamzell-Danielsson K. Prevalence and factors associated with sexually transmitted infections among HIV positive women opting for intrauterine contraception. *PLoS ONE* 2015; **10**(4): e0122400. 361. Kakooza F, Musinguzi P, Workneh M, et al. Implementation of a standardised and quality-assured enhanced gonococcal antimicrobial surveillance programme in accordance with WHO protocols in Kampala, Uganda. *Sexually Transmitted Infections* 2021; **97**(4): 312-6. 362. Kakou-Ngazoa S. Evaluation of real time pcr for detection of five sexually transmitted diseases in Abidjan, Ivory Coast. *Sexually Transmitted Infections* 2011; **87**: A275. 363. Kamali A, Byomire H, Muwonge C, et al. A randomised placebo-controlled safety and acceptability trial of PRO 2000 vaginal microbicide gel in sexually active women in Uganda. *Sexually Transmitted Infections* 2010; **86**(3): 222-6. 364. Kamali A, Quigley M, Nakiyingi J, et al. Syndromic management of sexually-transmitted infections and behaviour change interventions on transmission of HIV-1 in rural Uganda: A community randomised trial. *Lancet* 2003; **361**(9358): 645-52. 365. Kamanga JWK, Kalenga MK. Study of antibiotic resistance of Neisseria gonorrhoeae in man at the Labotest of Lubumbashi. [French]. *Revue Francaise de Gynecologie et d'Obstetrique* 1996; **91**(10): 471-4. 366. Kamenga MC, De Cock KM, St Louis ME, et al. The impact of human immunodeficiency virus infection on pelvic inflammatory disease: a case-control study in Abidjan, Ivory Coast. *Am J Obstet Gynecol* 1995; **172**(3): 919-25. 367. Kampikaho A, Irwig LM. A randomized trial of penicillin and streptomycin in the prevention of post-partum infection in Uganda. *International Journal of Gynecology and Obstetrics* 1993; **41**(1): 43-52. 368. Kanu EM, Rottmann H, Olaru ID, et al. Sexually transmitted infections in women in a rural hospital in Sierra Leone: a retrospective database study. *IJID Regions* 2025; **15**. 369. Kanyina EW, Kamau L, Muturi M. Cervical precancerous changes and selected cervical microbial infections, Kiambu County, Kenya, 2014: a cross sectional study. *BMC Infect Dis* 2017; **17**(1): 647. 370. Kapiga SH, Ewings FM, Ao T, et al. The epidemiology of HIV and HSV-2 infections among women participating in microbicide and vaccine feasibility studies in Northern Tanzania. *PLoS ONE* 2013; **8**(7): e68825. 371. Kapiga SH, Shao JF, Lwihula GK, Hunter DJ. Risk factors for HIV infection among women in Dar-es-Salaam, Tanzania. *J Acquir Immune Defic Syndr (1988)* 1994; **7**(3): 301-9. 372. Kapiga SH, Vuylsteke B, Lyamuya EF, Dallabetta G, Laga M. Evaluation of sexually transmitted diseases diagnostic algorithms among family planning clients in Dar es Salaam, Tanzania. *Sex Transm Infect* 1998; **74 Suppl 1**: S132-8. 373. Kapina M, Reid C, Roman K, et al. HIV incidence rates and risk factors for urban women in Zambia: Preparing for a microbicide clinical trial. *Sexually Transmitted Diseases* 2009; **36**(3): 129-33. 374. Kasprowicz D, Wilczynska W, Korzeniewski K. Health Status of Tsimihety Women: Sexually Transmitted Infections and Schistosomiasis, Northern Madagascar. *JOURNAL OF CLINICAL MEDICINE* 2025; **14**(10). 375. Katusiime C. Profiles of multidrug resistant gonorrhea in HIV-infected patients attending an urban hospital in Uganda. *HIV Medicine* 2019; **20 (Supplement 9)**: 215. 376. Katusiime C, Schlech WF, Parkes-Ratanshi R, Sempa J, Kambugu A. Characteristics of Sexually Transmitted Infections among High-Risk HIV-Positive Patients Attending an Urban Clinic in Uganda. *Journal of the International Association of Providers of AIDS Care* 2016; **15**(1): 36-41. 377. Kaul R, Kimani J, Nagelkerke NJ, et al. Monthly antibiotic chemoprophylaxis and incidence of sexually transmitted infections and HIV-1 infection in Kenyan sex workers: a randomized controlled trial. *Jama* 2004; **291**(21): 2555-62. 378. Kehinde AO, Lawoyin TO. Prevalence of STI/HIV co-infections among special treatment clinic attendees in Ibadan, Nigeria. *J R Soc Promot Health* 2005; **125**(4): 186-90. 379. Kerschberger B, Lekelem S, Daka M, et al. Mycoplasma genitalium infection in Eswatini amid syndromic case management: prevalence, coinfections, diagnostic challenges and treatment gaps. *BMC Infectious Diseases* 2025; **25**(1): 547. 380. Kerubo E, Laserson KF, Otecko N, et al. Prevalence of reproductive tract infections and the predictive value of girls' symptom-based reporting: findings from a cross-sectional survey in rural western Kenya. *Sex Transm Infect* 2016; **92**(4): 251-6. 381. Kesah FN, Payne V, Asakizi A. Prevalence and etiology of sexually transmitted infections in a gynecologic unit of a developing country. *Annals of Tropical Medicine and Public Health* 2013; **6**(5): 526-31. 382. Kestelyn P, Bogaerts J, Stevens AM, Piot P, Meheus A. Treatment of adult gonococcal keratoconjunctivitis with oral norfloxacin. *Am J Ophthalmol* 1989; **108**(5): 516-23. 383. Kharsany A, Hoosen A, Moodley J, Bagaratee J, Gouws E. The association between sexually transmitted pathogens and cervical intra-epithelial neoplasia in a developing community. *Sexually transmitted infections* 1993; **69**(5): 357-60. 384. Kharsany ABM, Hoosen AA, Moodley J. Bacterial vaginosis and lower genital tract infections in women attending out-patient clinics at a tertiary institution serving a developing community. *Journal of Obstetrics and Gynaecology* 1997; **17**(2): 171-5. 385. Kharsany ABM, McKinnon LR, Lewis L, et al. Population prevalence of sexually transmitted infections in a high HIV burden district in KwaZulu-Natal, South Africa: Implications for HIV epidemic control. *Int J Infect Dis* 2020; **98**: 130-7. 386. Kiene SM, Lule H, Sileo KM, Silmi KP, Wanyenze RK. Depression, alcohol use, and intimate partner violence among outpatients in rural Uganda: vulnerabilities for HIV, STIs and high risk sexual behavior. *BMC Infect Dis* 2017; **17**(1): 88. 387. Kim EJ, Hladik W, Barker J, et al. Sexually transmitted infections associated with alcohol use and HIV infection among men who have sex with men in Kampala, Uganda. *Sex Transm Infect* 2016; **92**(3): 240-5. 388. Kinoshita-Moleka R, Smith JS, Atibu J, et al. Low prevalence of HIV and other selected sexually transmitted infections in 2004 in pregnant women from Kinshasa, the Democratic Republic of the Congo. *Epidemiol Infect* 2008; **136**(9): 1290-6. 389. Kinuthia J, Drake AL, Matemo D, et al. HIV acquisition during pregnancy and postpartum is associated with genital infections and partnership characteristics. *AIDS* 2015; **29**(15): 2025-33. 390. Kiragga AN, Onzia A, Nakate V, et al. Community pharmacies: Key players in point-of-care diagnostics for STI screening in Africa. *PLoS ONE* 2024; **19**(12): e0315191. 391. Kivata MW, Mbuchi M, Eyase FL, et al. gyrA and parC mutations in fluoroquinolone-resistant Neisseria gonorrhoeae isolates from Kenya. *BMC Microbiol* 2019; **19**(1): 76. 392. Kiyingi J, Nabunya P, Bahar OS, et al. Prevalence and predictors of HIV and sexually transmitted infections among vulnerable women engaged in sex work: Findings from the Kyaterekera Project in Southern Uganda. *PLoS ONE* 2022; **17**(9 September): e0273238. 393. Klein JMA, Runge I, Pannen AK, et al. Prevalence of bacterial vaginosis, sexually transmitted infections and their association with HPV infections in asymptomatic women attending antenatal care in Ethiopia. *ecancermedicalscience* 2024; **18**: 1783. 394. Kleppa E, Holmen SD, Lillebo K, et al. Cervical ectopy: Associations with sexually transmitted infections and HIV. A cross-sectional study of high school students in rural South Africa. *Sexually Transmitted Infections* 2015; **91**(2): 124-9. 395. Klouman E, Masenga EJ, Klepp KI, Sam NE, Nkya W, Nkya C. HIV and reproductive tract infections in a total village population in rural Kilimanjaro, Tanzania: women at increased risk. *J Acquir Immune Defic Syndr Hum Retrovirol* 1997; **14**(2): 163-8. 396. Klouman E, Masenga EJ, Sam NE, Klepp KI. Asymptomatic gonorrhoea and chlamydial infection in a population-based and work-site based sample of men in Kilimanjaro, Tanzania. *Int J STD AIDS* 2000; **11**(10): 666-74. 397. Koffi SK, Faye-Kette H, Kacou-N'douba A, Kouassi-M'bengue A, Dosso M. [Evaluation of first void urine in the detection of Neisseria gonorrhoeae in patients less symptomatic in Abidjan (Côte d'Ivoire)]. *Med Trop (Mars)* 2009; **69**(3): 275-7. 398. Kosseim M, Ronald A, Plummer FA, D'Costa L, Brunham RC. Treatment of acute pelvic inflammatory disease in the ambulatory setting: trial of cefoxitin and doxycycline versus ampicillin-sulbactam. *Antimicrob Agents Chemother* 1991; **35**(8): 1651-6. 399. Kotikot T, Ndalamia J, Ogutu H, et al. Reproductive tract infections among low risk women attending KAVI-VZV 001 Study in Nairobi, Kenya. *AIDS Research and Human Retroviruses* 2016; **32 (Supplement 1)**: 281. 400. Koumans EH, Barker K, Massanga M, et al. Patient-led partner referral enhances sexually transmitted disease service delivery in two towns in the Central African Republic. *International Journal of STD and AIDS* 1999; **10**(6): 376-82. 401. Koyalta D, Mboumba Bouassa RS, Maiga A, et al. High Prevalence of Anal Oncogenic Human Papillomavirus Infection in Young Men Who Have Sex with Men Living in Bamako, Mali. *Infectious Agents and Cancer* 2021; **16**(1): 51. 402. Kreiss J, Ngugi E, Holmes K, et al. Efficacy of nonoxynol 9 contraceptive sponge use in preventing heterosexual acquisition of HIV in Nairobi prostitutes. *Jama* 1992; **268**(4): 477-82. 403. Kristensen JK. The prevalence of symptomatic sexually transmitted diseases and human immunodeficiency virus infection in outpatients in Lilongwe, Malawi. *Genitourin Med* 1990; **66**(4): 244-6. 404. Kufa T, Gumede L, Maseko DV, Radebe F, Kularatne R. The demographic and clinical profiles of women presenting with vaginal discharge syndrome at primary care facilities in south africa: Associations with age and implications for management. *South African Medical Journal* 2018; **108**(10). 405. Kularatne R, Maseko V, Mahlangu P, Muller E, Kufa T. Etiological Surveillance of Male Urethritis Syndrome in South Africa: 2019 to 2020. *Sexually Transmitted Diseases* 2022a; **49**(8): 560-4. 406. Kularatne R, Muller E, Maseko V, Dias BDC, Kufa T. Etiological Surveillance of Vaginal Discharge Syndrome in South Africa: 2019 to 2020. *Sexually Transmitted Diseases* 2022b; **49**(8): 565-70. 407. Kularatne RS, Kufa T, Gumede L, Maseko DV, Lewis DA. Demographic and behavioral risk factors associated with reduced susceptibility of neisseria gonorrhoeae to first-line antimicrobials in South African men with gonococcal urethral discharge. *Antimicrobial Agents and Chemotherapy* 2021; **65**(10): e00389-21. 408. Kurth AE, Sidle JE, Chhun N, et al. Computer-based counseling program (Care+ Kenya) to promote prevention and HIV health for people living with HIV/AIDS: A randomized controlled trial. *AIDS Education and Prevention* 2019; **31**(5): 395-406. 409. Kusemererwa S, Ruzagira E, Onyango M, Kabarambi A, Abaasa A. Associations between intravaginal practices and incidence of sexually transmitted infections and bacterial vaginosis among women enrolled in the dapivirine vaginal ring trial (The Ring Study) in southwestern Uganda: a retrospective secondary analysis. *BMJ Open* 2024; **14**(4): e079497. 410. Kwena ZA, Bukusi EA, Ng'ayo MO, et al. Prevalence and risk factors for sexually transmitted infections in a high-risk occupational group: the case of fishermen along Lake Victoria in Kisumu, Kenya. *Int J STD AIDS* 2010; **21**(10): 708-13. 411. Kyebambe PS, Namala C. Sexually transmissible infections (STI'S) among HIV clients attending an urban ugandan HIV clinic. *American Journal of Tropical Medicine and Hygiene* 2016; **95 (5 Supplement 1)**: 546. 412. Labbe A-C, Mendonca AP, Alves AC, et al. The impact of syphilis, HIV-1, and HIV-2 on pregnancy outcome in Bissau, Guinea-Bissau. *Sexually transmitted diseases* 2002: 157-67. 413. Labbé AC, Pépin J, Khonde N, et al. Periodical antibiotic treatment for the control of gonococcal and chlamydial infections among sex workers in Benin and Ghana: a cluster-randomized placebo-controlled trial. *Sex Transm Dis* 2012; **39**(4): 253-9. 414. Ladipo OA, Farr G, Otolorin E, et al. Prevention of IUD-related pelvic infection: the efficacy of prophylactic doxycycline at IUD insertion. *Adv Contracept* 1991; **7**(1): 43-54. 415. Lafort Y, Sawadogo Y, Delvaux T, Vuylsteke B, Laga M. Should family planning clinics provide clinical services for sexually transmitted infections? A case study from Côte d'Ivoire. *Trop Med Int Health* 2003; **8**(6): 552-60. 416. Laga M, Plummer FA, Nzanze H, et al. Epidemiology of ophthalmia neonatorum in Kenya. *Lancet* 1986; **2**(8516): 1145-9. 417. Laga M, Plummer FA, Piot P, et al. Prophylaxis of gonococcal and chlamydial ophthalmia neonatorum. A comparison of silver nitrate and tetracycline. *N Engl J Med* 1988; **318**(11): 653-7. 418. Langwenya N, Todd CS, Jones HE, et al. Risk-based screening to identify reproductive tract infection among HIV-infected women desiring use of intrauterine contraceptives. *BMJ Sexual and Reproductive Health* 2021; **47**(2): 137-43. 419. Lankoande S, Meda N, Sangare L, et al. Prevalence and risk of HIV infection among female sex workers in Burkina Faso. *International Journal of STD & AIDS* 1998; **9**(3): 146-50. 420. Lassey AT, Newman MJ, Opintan JA. Vaginal flora of first time urban family planning attendants in Accra, Ghana. *West African Journal of Medicine* 2005; **24**(3): 219-22. 421. Latif AS. Sexually, transmitted disease in clinic patients in Salisbury, Zimbabwe. *Br J Vener Dis* 1981; **57**(3): 181-3. 422. Latif AS, Gwanzura L, Machiha A, et al. Antimicrobial susceptibility in Neisseria gonorrhoeae isolates from five sentinel surveillance sites in Zimbabwe, 2015-2016. *Sex Transm Infect* 2018; **94**(1): 62-6. 423. Latif AS, Mason PR, Marowa E, Gwanzura L, Chingono A, Mbengeranwa OL. Risk factors for gonococcal and chlamydial cervical infection in pregnant and non-pregnant women in Zimbabwe. *Cent Afr J Med* 1999; **45**(10): 252-8. 424. Laurent C, Seck K, Coumba N, et al. Prevalence of HIV and other sexually transmitted infections, and risk behaviours in unregistered sex workers in Dakar, Senegal. *AIDS* 2003; **17**(12): 1811-6. 425. Lazenby GB, Taylor PT, Badman BS, et al. An association between Trichomonas vaginalis and high-risk human papillomavirus in rural Tanzanian women undergoing cervical cancer screening. *Clin Ther* 2014; **36**(1): 38-45. 426. Le Bacq F, Mason PR, Gwanzura L, Robertson VJ, Latif AS. HIV and other sexually transmitted diseases at a rural hospital in Zimbabwe. *Genitourin Med* 1993; **69**(5): 352-6. 427. Le Noc P, Le Noc D. The female genital tract infections observed in Yaounde: the importance of gonococcal etiology. *Medecine Tropicale* 1974; **34**(4): 573-81. 428. Le Roux M, Ngwenya IK, Nemarude AL, De Villiers BE, Mathebula M, Nchabeleng M. Sexually transmitted infections and sexual behaviour among men having sex with men from Tshwane, South Africa. *International Journal of STD and AIDS* 2023; **34**(3): 183-90. 429. le Roux MC, Hoosen AA. Quantitative Real-Time Polymerase Chain Reaction for the Diagnosis of Mycoplasma genitalium Infection in South African Men With and Without Symptoms of Urethritis. *Sex Transm Dis* 2017; **44**(1): 17-20. 430. LeeVan E, Hu F, Mitchell AB, et al. Associations of gender identity with sexual behaviours, social stigma and sexually transmitted infections among adults who have sex with men in Abuja and Lagos, Nigeria. *Journal of the International AIDS Society* 2022; **25**(7): e25956. 431. Lemos MP, Lazarus E, Isaacs A, et al. Daily Vaginal Swabs and Mobile Phone Sex Report for Assessing HIV Virion Exposure Prospectively among a Cohort of Young Sexually Active Women in South Africa (HVTN 915). *Journal of Acquired Immune Deficiency Syndromes* 2019; **81**(2): e39-e48. 432. Lepine A, Szawlowski S, Nitcheu E, et al. The effect of protecting women against economic shocks to fight HIV in Cameroon, Africa: The POWER randomised controlled trial. *PLoS Medicine* 2024; **21**(10): e1004355. 433. Leroy V, De Clercq A, Ladner J, Bogaerts J, Van De Perre P, Dabis F. Should screening of genital infections be part of antenatal care in areas of high HIV prevalence? A prospective cohort study from Kigali, Rwanda, 1992-1993. *Genitourinary Medicine* 1995; **71**(4): 207-11. 434. Leutscher P, Jensen JS, Hoffmann S, et al. Sexually transmitted infections in rural Madagascar at an early stage of the HIV epidemic: a 6-month community-based follow-up study. *Sex Transm Dis* 2005; **32**(3): 150-5. 435. Lewis DA, Chirwa TF, Msimang VM, Radebe FM, Kamb ML, Firnhaber CS. Urethritis/cervicitis pathogen prevalence and associated risk factors among asymptomatic HIV-infected patients in South Africa. *Sex Transm Dis* 2012b; **39**(7): 531-6. 436. Lewis DA, Muller E, Steele L, et al. Prevalence and associations of genital ulcer and urethral pathogens in men presenting with genital ulcer syndrome to primary health care clinics in South Africa. *Sex Transm Dis* 2012a; **39**(11): 880-5. 437. Lewis DA, Pillay C, Mohlamonyane O, et al. The burden of asymptomatic sexually transmitted infections among men in Carletonville, South Africa: Implications for syndromic management. *Sexually Transmitted Infections* 2008b; **84**(5): 371-6. 438. Lewis DA, Ricketts C, Vezi A, Maseko V. Do you have an STI? Findings from a dedicated men's sexual health clinic in alexandra township, South Africa. *Sexually Transmitted Infections Conference: STI and AIDS World Congress* 2013; **89**(SUPPL. 1). 439. Lewis DA, Scott L, Slabbert M, et al. Escalation in the relative prevalence of ciprofloxacin-resistant gonorrhoea among men with urethral discharge in two South African cities: association with HIV seropositivity. *Sex Transm Infect* 2008a; **84**(5): 352-5. 440. Libombo A, Folgosa E, Bergstrom S. Risk factors in puerperal endometritis-myometritis: An incident case-referent study. *Gynecologic and Obstetric Investigation* 1994; **38**(3): 198-205. 441. Lindan CP, Anglemyer A, Hladik W, et al. High-risk motorcycle taxi drivers in the HIV/AIDS era: a respondent-driven sampling survey in Kampala, Uganda. *International Journal of STD and AIDS* 2015; **26**(5): 336-45. 442. Lindman J, Djalo MA, Biai A, et al. Prevalence of sexually transmitted infections and associated risk factors among female sex workers in Guinea-Bissau. *Sex Transm Infect* 2024; **100**(7): 411-7. 443. Lô BB, Philippon M, Cunin P, Meynard D, Tandia-Diagana M. [The microbial etiology of genital discharges in Nouakchott, Mauritania]. *Bull Soc Pathol Exot* 1997; **90**(2): 81-2. 444. Lokken EM, Jisuvei C, Oyaro B, et al. Nugent Score, Amsel's Criteria, and a Point-of-Care Rapid Test for Diagnosis of Bacterial Vaginosis: Performance in a Cohort of Kenyan Women. *Sexually Transmitted Diseases* 2022; **49**(1): E22-E5. 445. Louis JP, Migliani R, Trebucq A, et al. Management of sexually transmitted diseases in urban environments in Cameroon in 1992. [French]. *Annales de la Societe Belge de Medecine Tropicale* 1993a; **73**(4): 267-78. 446. Low AJ, Konate I, Nagot N, et al. Neisseria gonorrhoeae and Chlamydia trachomatis infection in HIV-1-infected women taking antiretroviral therapy: A prospective cohort study from Burkina Faso. *Sexually Transmitted Infections* 2014; **90**(2): 100 EP - 3. 447. Lowe S, Mudzviti T, Mandiriri A, et al. Sexually transmitted infections, the silent partner in HIV-infected women in Zimbabwe. *Southern African Journal of HIV Medicine* 2019; **20**(1). 448. Lowndes CM, Alary M, Gnintoungbé CAB, et al. Management of sexually transmitted diseases and HIV prevention in men at high risk: targeting clients and non-paying sexual partners of female sex workers in Benin. *AIDS* 2000; **14**(16). 449. Lowndes CM, Alary M, Labbe AC, et al. Interventions among male clients of female sex workers in Benin, West Africa: An essential component of targeted HIV preventive interventions. *Sexually Transmitted Infections* 2007; **83**(7): 577-81. 450. Luján J, de Oñate WA, Delva W, et al. Prevalence of sexually transmitted infections in women attending antenatal care in Tete province, Mozambique. *S Afr Med J* 2008; **98**(1): 49-51. 451. Lule G, Behets FM, Hoffman IF, et al. STD/HIV control in Malawi and the search for affordable and effective urethritis therapy: a first field evaluation. *Genitourin Med* 1994; **70**(6): 384-8. 452. Luntamo M, Kulmala T, Mbewe B, Cheung YB, Maleta K, Ashorn P. Effect of repeated treatment of pregnant women with sulfadoxine- pyrimethamine and azithromycin on preterm delivery in Malawi: A randomized controlled trial. *American Journal of Tropical Medicine and Hygiene* 2010; **83**(6): 1212-20. 453. Ly F, Guéye N, Samb ND, Sow PS, Ndiaye B, Mahé A. [Prospective study of sexually transmitted infections in Dakar, Senegal]. *Med Trop (Mars)* 2006; **66**(1): 64-8. 454. Mabey D, Hanlon P, Hanlon L, Marsh V, Forsey T. Chlamydial and gonococcal ophthalmia neonatorum in The Gambia. *Ann Trop Paediatr* 1987; **7**(3): 177-80. 455. Mabey DC, Ogbaselassie G, Robertson JN, Heckels JE, Ward ME. Tubal infertility in the Gambia: chlamydial and gonococcal serology in women with tubal occlusion compared with pregnant controls. *Bull World Health Organ* 1985; **63**(6): 1107-13. 456. Mabey DC, Whittle HC. Genital and neonatal chlamydial infection in a trachoma endemic area. *Lancet* 1982; **2**(8293): 300-1. 457. Mabey DCW, Lloydevans NE, Conteh S, Forsey T. SEXUALLY-TRANSMITTED DISEASES AMONG RANDOMLY SELECTED ATTENDERS AT AN ANTENATAL CLINIC IN THE GAMBIA. *BRITISH JOURNAL OF VENEREAL DISEASES* 1984; **60**(5): 331-6. 458. Mabonga E, Parkes-Ratanshi R, Riedel S, et al. Complete ciprofloxacin resistance in gonococcal isolates in an urban Ugandan clinic: findings from a cross-sectional study. *Int J STD AIDS* 2019; **30**(3): 256-63. 459. Machungo F, Zanconato G, Persson K, et al. Syphilis, gonorrhoea and chlamydial infection among women undergoing legal or illegal abortion in Maputo. *Int J STD AIDS* 2002; **13**(5): 326-30. 460. Maduna LD, Kock MM, van der Veer B, et al. Antimicrobial Resistance of Neisseria gonorrhoeae Isolates from High-Risk Men in Johannesburg, South Africa. *Antimicrob Agents Chemother* 2020; **64**(11). 461. Maduna LD, Taole M, Motanteli K, Mothobi I, Mosehle T, Peters RPH. High rates of ciprofloxacin and doxycycline resistance in Neisseria gonorrhoeae infection in Lesotho. *Sexual Health* 2024; **24**(4): 9. 462. Maffre E, Ba H, Mattern, Baylet R, Gueye C, Wone I. [Venereal diseases at the Institut d'Hygiène Sociale de Dakar. Epidemiologic aspects. II. Gonorrhea]. *Bull Soc Med Afr Noire Lang Fr* 1965; **10**(4): 603-8. 463. Mahenge A, Mwalongo W, Mnkai J, et al. The prevalence of sexually transmitted infections and risk factors among young adult female Mbeya-Tanzania. *BMC Proceedings* 2022; **16**(Supplement 2). 464. Mahlangu MP, Müller EE, Venter JME, Maseko DV, Kularatne RS. The Prevalence of Mycoplasma genitalium and Association With Human Immunodeficiency Virus Infection in Symptomatic Patients, Johannesburg, South Africa, 2007-2014. *Sex Transm Dis* 2019; **46**(6): 395-9. 465. Maina AN, Kimani J, Anzala O. Prevalence and risk factors of three curable sexually transmitted infections among women in Nairobi, Kenya. *BMC Res Notes* 2016; **9**: 193. 466. Maina AN, Mureithi MW, Ndemi JK, Revathi G. Diagnostic accuracy of the syndromic management of four stis among individuals seeking treatment at a health centre in nairobi, kenya: A cross-sectional study. *Pan African Medical Journal* 2021; **40**: 138. 467. Malefo MA, Ayo-Yusuf O, Mokgatle MM. Risk factors for sexually transmitted infections among men who have sex with men. *African journal of primary health care & family medicine* 2023; **15**(1): e1-e7. 468. Malope BI, MacPhail P, Mbisa G, et al. No evidence of sexual transmission of Kaposi's sarcoma herpes virus in a heterosexual South African population. *AIDS* 2008; **22**(4): 519-26. 469. Mamadou S, Rabiou S, Aboubacar A, et al. Prevalence of the HIV infection and five other sexually-transmitted infections among sex workers in Niamey, Niger. *Bulletin de la Societe de pathologie exotique (1990)* 2006; **99**(1): 19-22. 470. Mandara NA, Takulia S, Kanyawana J, Mhalu F. Asymptomatic gonorrhoea in women attending family planning clinics in Dar es Salaam, Tanzania. Results of a pilot study. *Trop Geogr Med* 1980; **32**(4): 329-32. 471. Manga M, Hassan U, Halima F, et al. Sexually transmitted infections in gombe, North-Eastern Nigeria. *Sexually Transmitted Infections* 2021; **97**(SUPPL 1): A162. 472. Manhart LE, Mostad SB, Baeten JM, Astete SG, Mandaliya K, Totten PA. High Mycoplasma genitalium organism burden is associated with shedding of HIV-1 DNA from the cervix. *Journal of Infectious Diseases* 2008; **197**(5): 733-6. 473. Manjate A, Sergon G, Kenga D, et al. Prevalence of sexually transmitted infections (STIs), associations with sociodemographic and behavioural factors, and assessment of the syndromic management of vaginal discharge in women with urogenital complaints in Mozambique. *Front Reprod Health* 2024; **6**. 474. Manning-Geist B, Murphy B, Comeau D, et al. Predictors of medical outcome in 1,712 Ethiopian survivors of rape. *Annals of Global Health* 2016; **82 (3)**: 324. 475. Månsson F, Camara C, Biai A, et al. High prevalence of HIV-1, HIV-2 and other sexually transmitted infections among women attending two sexual health clinics in Bissau, Guinea-Bissau, West Africa. *Int J STD AIDS* 2010; **21**(9): 631-5. 476. Marcus R, C P, Gill K, et al. Acceptability, feasibility and cost of point of care testing for sexually transmitted infections among South African adolescents where syndromic management is standard of care. *BMC health services research* 2023; **23**(1): 1078. 477. Martin K, Dauya E, Dziva Chikwari C, et al. Evaluation of a novel point-of-care lateral flow assay screening for Neisseria gonorrhoeae infection among pregnant women in Zimbabwe. *PLOS Glob Public Health* 2025; **5**(2). 478. Masawe AE, Nsibambi J, Lomholt G. Treatment of gonorrhoea with a combination of probenecid and sodium penicillin G among African Ugandans. *Afr J Med Sci* 1972; **3**(2): 163-7. 479. Masese L, Baeten JM, Richardson BA, et al. Changes in the contribution of genital tract infections to HIV acquisition among Kenyan high-risk women from 1993 to 2012. *AIDS* 2015; **29**(9): 1077-85. 480. Masese LN, Wanje G, Kabare E, et al. Screening for Sexually Transmitted Infections in Adolescent Girls and Young Women in Mombasa, Kenya: Feasibility, Prevalence, and Correlates. *Sex Transm Dis* 2017; **44**(12): 725-31. 481. Masha SC, Wahome E, Vaneechoutte M, Cools P, Crucitti T, Sanders EJ. High prevalence of curable sexually transmitted infections among pregnant women in a rural county hospital in Kilifi, Kenya. *PLoS ONE* 2017; **12**(3). 482. Mashingaidze R, Moodie Z, Allen M, et al. Sexually transmitted infections amongst men who have sex with men (MSM) in South Africa. *PLOS Glob Public Health* 2023; **3**(4). 483. Mason PR, Gwanzura L, Latif AS, Marowa E. Genital infections in women attending a genito-urinary clinic in Harare, Zimbabwe. *Genitourin Med* 1990; **66**(3): 178-81. 484. Mason PR, Katzenstein DA, Chimbira TH, Mtimavalye L. Vaginal flora of women admitted to hospital with signs of sepsis following normal delivery, cesarean section or abortion. The Puerperal Sepsis Study Group. *Cent Afr J Med* 1989a; **35**(3): 344-51. 485. Mason PR, Katzenstein DA, Chimbira TH, Mtimavalye L. Microbial flora of the lower genital tract of women in labour at Harare Maternity Hospital. The Puerperal Sepsis Study Group. *Cent Afr J Med* 1989b; **35**(3): 337-44. 486. Mathebula RC, Kuonza LR, Musekiwa A, Kularatne R, Maseko V, Kufa T. Factors associated with repeat genital symptoms among sexually transmitted infection service attendees in South Africa, 2015 - 2016. *S Afr Med J* 2020; **110**(7): 661-6. 487. Mathews C, van Rensburg A, Schierhout G, et al. An assessment of care provided by a public sector STD clinic in Cape Town. *Int J STD AIDS* 1998; **9**(11): 689-94. 488. Matoga M, Chen J, Massa C, et al. Assessment of urethritis etiology among HIV-infected men attending an STI clinic in Lilongwe, Malawi. *Sexually Transmitted Infections* 2019; **95 (Supplement 1)**: A337-A8. 489. Matteelli A, Kassa A, Gerbase A, et al. Passive sentinel surveillance system for sexually transmitted diseases in primary healthcare sites in Ethiopia, 1991-3. *Sexually Transmitted Infections* 2000; **76**(2): 131-3. 490. Maueia C, Murahwa A, Manjate A, et al. The relationship between selected sexually transmitted pathogens, HPV and HIV infection status in women presenting with gynaecological symptoms in Maputo City, Mozambique. *PLoS ONE* 2024; **19**(9): e0307781. 491. Maureen EC, Chinyere U, Charity ON, Chidera O. CONCOMITANT MALARIAL INFECTION AND PATHOGENIC MICROBIOTA IN THE REPRODUCTIVE TRACT OF PREGNANT WOMEN IN ORLU, IMO STATE, NIGERIA. *Asian Journal of Microbiology, Biotechnology and Environmental Sciences* 2022; **24**(2): 409-13. 492. Mavedzenge SN, Weiss HA, Montgomery ET, et al. Determinants of differential HIV incidence among women in three southern African locations. *J Acquir Immune Defic Syndr* 2011; **58**(1): 89-99. 493. Mayanja Y, Kamacooko O, Senyonga W, et al. Willingness of adolescent girls and young women in Kampala, Uganda to participate in future efficacy trials of novel biomedical HIV prevention interventions: the antiretroviral implant. *Journal of the International AIDS Society* 2021; **24 (SUPPL 1)**: 213. 494. Mayanja Y, Lunkuse J, Kalungi H, Senyonga W, Kamacooko O, Ruzagira E. High prevalence of curable sexually transmitted infections among 14-19 year old adolescents at risk of HIV infection in Kampala, Uganda. *Sexually Transmitted Infections* 2021; **97**(SUPPL 1): A94. 495. Mayaud P, Changalucha J, Grosskurth H, et al. The value of urine specimens in screening for male urethritis and its microbial aetiologies in Tanzania. *Genitourin Med* 1992; **68**(6): 361-5. 496. Mayaud P, Grosskurth H, Changalucha J, et al. Risk assessment and other screening options for gonorrhoea and chlamydial infections in women attending rural Tanzanian antenatal clinics. *Bull World Health Organ* 1995; **73**(5): 621-30. 497. Mayaud P, ka-Gina G, Cornelissen J, et al. Validation of a WHO algorithm with risk assessment for the clinical management of vaginal discharge in Mwanza, Tanzania. *Sex Transm Infect* 1998a; **74 Suppl 1**: S77-84. 498. Mayaud P, Msuya W, Todd J, et al. STD rapid assessment in Rwandan refugee camps in Tanzania. *Genitourin Med* 1997; **73**(1): 33-8. 499. Mayaud P, Uledi E, Cornelissen J, et al. Risk scores to detect cervical infections in urban antenatal clinic attenders in Mwanza, Tanzania. *Sex Transm Infect* 1998b; **74 Suppl 1**: S139-46. 500. Mbizvo EM, Msuya SE, Stray-Pedersen B, Sundby J, Chirenje ZM, Hussain A. Determinants of reproductive tract infections among asymptomatic women in Harare, Zimbabwe. *Cent Afr J Med* 2001; **47**(3): 57-64. 501. Mboup A, Béhanzin L, Guédou FA, et al. Early antiretroviral therapy and daily pre-exposure prophylaxis for HIV prevention among female sex workers in Cotonou, Benin: a prospective observational demonstration study. *Journal of the International AIDS Society* 2018; **21**(11): e25208. 502. Mbu ER, Kongnyuy EJ, Mbopi-Keou FX, Tonye RN, Nana PN, Leke RJI. Gynaecological morbidity among HIV positive pregnant women in Cameroon. *Reproductive Health* 2008; **5**(1): 3. 503. Mbu RE, Mbopi-Keou FX, Alemdji G, et al. Unexpectedly high prevalence of sexually transmitted diseases in married women attending family planning clinics in Yaounde, Cameroon [1]. *International Journal of STD and AIDS* 2005; **16**(3): 270-1. 504. McCallum M, Tozer RA. A survey of selected vaginal flora in Malawian women. *Cent Afr J Med* 1973; **19**(8): 176-8. 505. McClelland RS, Baeten JM, Richardson BA, et al. A comparison of genital HIV-1 shedding and sexual risk behavior among Kenyan women based on eligibility for initiation of HAART according to WHO guidelines. *Journal of Acquired Immune Deficiency Syndromes* 2006; **41**(5): 611-5. 506. McClelland RS, Wang CC, Mandaliya K, et al. Treatment of cervicitis is associated with decreased cervical shedding of HIV-1. *AIDS* 2001; **15**(1): 105-10. 507. McCormack S, Ramjee G, Kamali A, et al. PRO2000 vaginal gel for prevention of HIV-1 infection (Microbicides Development Programme 301): a phase 3, randomised, double-blind, parallel-group trial. *The Lancet* 2010; **376**(9749): 1329-37. 508. McHaro RD, Kisinda A, Njovu L, et al. Prevalence of and risk factors associated with HIV, Herpes Simplex Virus-type 2, Chlamydia trachomatis and Neisseria gonorrhoeae infections among 18–24 year old students attending Higher Learning Institutions in Mbeya-Tanzania. *PLoS ONE* 2022; **17**(5 May). 509. McKinnon LR, Izulla P, Nagelkerke N, et al. Risk Factors for HIV Acquisition in a Prospective Nairobi-Based Female Sex Worker Cohort. *AIDS and behavior* 2015; **19**(12): 2204-13. 510. Meda N, Ledru S, Fofana M, et al. Sexually transmitted diseases and human immunodeficiency virus infection among women with genital infections in Burkina Faso. *International Journal of STD and AIDS* 1995; **6**(4): 273-7. 511. Meda N, Sangare L, Lankoande S, et al. Pattern of sexually transmitted diseases among pregnant women in Burkina Faso, west Africa: Potential for a clinical management based on simple approaches. *Genitourinary Medicine* 1997; **73**(3): 188-93. 512. Meheus A. [Incidence and prevalence of venereal diseases in selected populations from urban areas in Rwanda]. *Ann Soc Belg Med Trop* 1973b; **53**(3): 179-85. 513. Meheus A, Ballard R, Dlamini M, Ursi JP, Van Dyck E, Piot P. Epidemiology and aetiology of urethritis in Swaziland. *Int J Epidemiol* 1980a; **9**(3): 239-45. 514. Meheus A, De Clercq A, Prat R. Prevalence of gonorrhoea in prostitutes in a Central African town. *The British journal of venereal diseases* 1974; **50**(1): 50-2. 515. Meheus A, Friedman F, Van Dyck E, Guyver T. Genital infections in prenatal and family planning attendants in Swaziland. *East Afr Med J* 1980b; **57**(3): 212-7. 516. Meheus A, Van Dyck E, Friedman F. Genital infections in Swaziland. *Ann Soc Belg Med Trop* 1982; **62**(4): 361-7. 517. Meheus AZ. Epidemiological treatment of gonorrhoea. *Lancet* 1973a; **2**(7830): 678-9. 518. Mehta SD, Agingu W, Garges E, et al. Extended-spectrum s-lactamase and macrolide resistance in Neisseria gonorrhoeae in western Kenya. *Sexually Transmitted Diseases* 2024; **51**(1 Supplement 1): S122. 519. Mehta SD, Okall D, Graham SM, N'Gety G, Bailey RC, Otieno F. Behavior Change and Sexually Transmitted Incidence in Relation to PREP Use Among Men Who Have Sex with Men in Kenya. *AIDS and behavior* 2021; **25**(7): 2219-29. 520. Mehta SD, Zulaika G, Agingu W, et al. Analysis of bacterial vaginosis, the vaginal microbiome, and sexually transmitted infections following the provision of menstrual cups in Kenyan schools: Results of a nested study within a cluster randomized controlled trial. *PLoS Medicine* 2023; **20**(7 July): e1004258. 521. Meiring JA, Kemp E, Jennings DL, Mdhlovu M, Koornhof HJ. High-level penicillin-resistant gonococcal infections in Port Elizabeth. *S Afr Med J* 1989; **75**(3): 118-9. 522. Melendez J, Dawa B, Hamill MM, et al. Oropharyngeal gonorrhea among men with symptomatic urethritis seeking treatment at government health centers in Kampala, Uganda. *Sexually Transmitted Diseases* 2024; **51**(1 Supplement 1): S127-S8. 523. Meless H, Abegaze B. Drug susceptibility of Neisseria isolates from patients attending clinics for sexually transmitted diseases in Addis Ababa. *East Afr Med J* 1997; **74**(7): 447-9. 524. Melo J, Folgosa E, Manjate D, et al. Low prevalence of HIV and other sexually transmitted infections in young women attending a youth counselling service in Maputo, Mozambique. *Tropical Medicine and International Health* 2008; **13**(1): 17-20. 525. Menendez C, Castellsague X, Renom M, et al. Prevalence and risk factors of sexually transmitted infections and cervical neoplasia in women from a rural area of southern Mozambique. *Infectious Diseases in Obstetrics and Gynecology* 2010; **2010 (no pagination)**. 526. Mertelsmann AM, Mukerebe C, Miyaye D, et al. Clinical and Demographic Factors Associated With Kaposi Sarcoma–Associated Herpesvirus Shedding in Saliva or Cervical Secretions in a Cohort of Tanzanian Women. *Open Forum Infectious Diseases* 2024; **11**(4). 527. Mgodi NM, Takuva S, Edupuganti S, et al. A Phase 2b Study to Evaluate the Safety and Efficacy of VRC01 Broadly Neutralizing Monoclonal Antibody in Reducing Acquisition of HIV-1 Infection in Women in Sub-Saharan Africa: Baseline Findings. *J Acquir Immune Defic Syndr* 2021; **87**(1): 680-7. 528. Mhalu FS. Inter-relationships between HIV infection and other sexually transmitted diseases. *East Afr Med J* 1990; **67**(7): 512-7. 529. Mhlongo S, Magooa P, Muller EE, et al. Etiology and STI/HIV coinfections among patients with urethral and vaginal discharge syndromes in South Africa. *Sex Transm Dis* 2010; **37**(9): 566-70. 530. Migliori GB, Borghesi A, Adriko C, et al. Tuberculosis and HIV infection association in a rural district of northern Uganda: epidemiological and clinical considerations. *Tuber Lung Dis* 1992; **73**(5): 285-90. 531. Mirza NB, Nsanze H, D'Costa LJ, Piot P. Microbiology of vaginal discharge in Nairobi, Kenya. *Br J Vener Dis* 1983; **59**(3): 186-8. 532. Mithi B, Luhanga M, Kaminyoghe F, Chiumia F, Banda DL, Nyama L. Antibiotic use and resistance patterns at Rumphi District Hospital in Malawi: a cross-sectional study. *BMC Infectious Diseases* 2024; **24**(1): 445. 533. Mkhize P, Mehou-Loko C, Maphumulo N, et al. Differences in HIV risk factors between South African adolescents and adult women and their association with sexually transmitted infections. *Sex Transm Infect* 2025; **101**(3): 174-82. 534. Mlisana K, Naicker N, Werner L, et al. Symptomatic vaginal discharge is a poor predictor of sexually transmitted infections and genital tract inflammation in high-risk women in South Africa. *Journal of Infectious Diseases* 2012; **206**(1): 6-14. 535. Mmbaga EJ, Hussain A, Leyna GH, et al. Incidence of HIV-1 infection and changes in prevalence of reproductive tract infections and sexual risk behaviours: a population-based longitudinal study in rural Tanzania. *Afr J AIDS Res* 2006; **5**(3): 281-8. 536. Mocumbi S, Gafos M, Munguambe K, Goodall R, McCormack S. High HIV prevalence and incidence among women in Southern Mozambique: Evidence from the MDP microbicide feasibility study. *PLoS ONE* 2017; **12**(3). 537. Mofolorunsho K, Mabaso N, Nundlall N, Nightingale A, Nyirenda M, Abbai N. Prevalence and associated risk factors of chlamydia and gonorrhoea infections among men who have sex with men in Durban, South Africa. *African journal of reproductive health* 2024; **28**(4): 90-110. 538. Mogaka JN, Abuna F, Dettinger JC, et al. HIGH ACCEPTABILITY OF STI TESTING AND EPT AMONG PREGNANT KENYAN WOMEN INITIATING PrEP. *Topics in Antiviral Medicine* 2023; **31**(2): 306. 539. Momanyi V, Rono B, Musinguzi N, et al. Prevalence and incidence of sexually transmitted infections in young women receiving peer-supported, community-delivered PrEP and other sexual health services in Kisumu, Kenya. *Sexual Health* 2024; **24**(4): 100. 540. Moodley C, Tootla H, Amien I, Engel ME. Evaluating the utility of the allplex STI essential assay to determine the occurrence of urogenital sexually transmitted infections among symptomatic and asymptomatic patients in cape town, south africa. *PLoS ONE* 2023; **18**(11 November): e0292534. 541. Moodley D, Moodley P, Sebitloane M, et al. High prevalence and incidence of asymptomatic sexually transmitted infections during pregnancy and postdelivery in KwaZulu Natal, South Africa. *Sex Transm Dis* 2015; **42**(1): 43-7. 542. Moodley P, Pillay C, Nzimande G, Coovadia YM, Sturm AW. Lower dose of ciprofloxacin is adequate for the treatment of Neisseria gonorrhoeae in KwaZulu Natal, South Africa. *Int J Antimicrob Agents* 2002a; **20**(4): 248-52. 543. Moodley P, Sturm PD, Connolly C, Sturm AW. Identification of women at high STD risk among STD clinic attendees: implications for STD programmes. *International journal of STD & AIDS* 2003; **14**(8): 526-31. 544. Moodley P, Wilkinson D, Connolly C, Moodley J, Sturm AW. Trichomonas vaginalis is associated with pelvic inflammatory disease in women infected with human immunodeficiency virus. *Clin Infect Dis* 2002; **34**(4): 519-22. 545. Morar NS, Ramjee G, Gouws E, Wilkinson D. Vaginal douching and vaginal substance use among sex workers in KwaZulu-Natal, South Africa. *S Afr J Sci* 2003; **99**(7-8): 371-4. 546. Morency P, Dubois MJ, Gresenguet G, et al. Aetiology of urethral discharge in Bangui, Central African Republic. *Sexually Transmitted Infections* 2001; **77**(2): 125-9. 547. Morikawa E, Mudau M, Olivier D, et al. Acceptability and Feasibility of Integrating Point-of-Care Diagnostic Testing of Sexually Transmitted Infections into a South African Antenatal Care Program for HIV-Infected Pregnant Women. *Infect Dis Obstet Gynecol* 2018; **2018**: 3946862. 548. Morrison CS, Sekadde-Kigondu C, Miller WC, Weiner DH, Sinei SK. Use of sexually transmitted disease risk assessment algorithms for selection of intrauterine device candidates. *Contraception* 1999; **59**(2): 97-106. 549. Morrison CS, Sunkutu MR, Musaba E, Glover LH. Sexually transmitted disease among married Zambian women: the role of male and female sexual behaviour in prevention and management. *Genitourin Med* 1997; **73**(6): 555-7. 550. Moses E, Pedersen HN, Mitchell SM, et al. Uptake of community-based, self-collected HPV testing vs. visual inspection with acetic acid for cervical cancer screening in Kampala, Uganda: Preliminary results of a randomised controlled trial. *Tropical Medicine and International Health* 2015; **20**(10): 1355-67. 551. Moses S, Ngugi EN, Costigan A, et al. Response of a sexually transmitted infection epidemic to a treatment and prevention programme in Nairobi, Kenya. *Sexually Transmitted Infections* 2002; **78**(SUPPL. 1): i114-i20. 552. Moss GB, Overbaugh J, Welch M, et al. Human immunodeficiency virus DNA in urethral secretions in men: association with gonococcal urethritis and CD4 cell depletion. *Journal of infectious diseases* 1995; **172**(6): 1469-74. 553. Mostad SB, Overbaugh J, DeVange DM, et al. Hormonal contraception, vitamin A deficiency, and other risk factors for shedding of HIV-1 infected cells from the cervix and vagina. *Lancet* 1997; **350**(9082): 922-7. 554. Msemwaa B, Mushi MF, Kidenyac B, et al. Urogenital pathogens in urine samples of clinically diagnosed urinary tract infected patients in Tanzania: A laboratory based cross-sectional study. *IJID Regions* 2023; **7**: 170-5. 555. Msuya SE, Uriyo J, Hussain A, et al. Prevalence of sexually transmitted infections among pregnant women with known HIV status in northern Tanzania. *Reproductive Health* 2009; **6**(1). 556. Mtove G, Chico RM, Madanitsa M, et al. Fetal growth and birth weight are independently reduced by malaria infection and curable sexually transmitted and reproductive tract infections in Kenya, Tanzania, and Malawi: A pregnancy cohort study. *International Journal of Infectious Diseases* 2023; **135**: 28-40. 557. Muitta E, Were T, Nyamache AK, Muhoho N. Atypical cervical cytomorphologic predictors: A descriptive study of pre-cervical cancer patients of low education in kenya. *Pan African Medical Journal* 2019; **33**. 558. Mukenge-Tshibaka L, Alary M, Lowndes CM, et al. Syndromic versus laboratory-based diagnosis of cervical infections among female sex workers in Benin: Implications of nonattendance for return visits. *Sexually Transmitted Diseases* 2002; **29**(6): 324-30. 559. Mulanga-Kabeya C, Morel E, Patrei D, et al. Prevalence and risk assessment for sexually transmitted infections in pregnant women and female sex workers in Mali: Is syndromic approach suitable for screening? *Sexually Transmitted Infections* 1999; **75**(5): 358-60. 560. Müller EE, Gumede LYE, Maseko DV, et al. Emergence of high-level azithromycin-resistant <i>Neisseria gonorrhoeae</i> causing male urethritis in Johannesburg, South Africa, 2021. *SEXUAL HEALTH* 2024; **21**(1). 561. Mullick S, Cox LA, Martin CE, Fipaza Z, Ncube S. Comparing the Integration of Syndromic versus Etiological Management of Sexually Transmitted Infections Into HIV Pre-Exposure Prophylaxis Services for Adolescent Girls and Young Women, in South Africa. *Journal of Adolescent Health* 2023; **73**(6 Supplement): S67-S72. 562. Mulu W, Abera B, Yimer M, Hailu T, Ayele H, Abate D. Bacterial agents and antibiotic resistance profiles of infections from different sites that occurred among patients at Debre Markos Referral Hospital, Ethiopia: a cross-sectional study. *BMC Res Notes* 2017; **10**(1): 254. 563. Mulu W, Yimer M, Zenebe Y, Abera B. Common causes of vaginal infections and antibiotic susceptibility of aerobic bacterial isolates in women of reproductive age attending at Felegehiwot referral Hospital, Ethiopia: A cross sectional study. *BMC Women's Health* 2015; **13**. 564. Mungati M, Machiha A, Mugurungi O, et al. The Etiology of Genital Ulcer Disease and Coinfections With Chlamydia trachomatis and Neisseria gonorrhoeae in Zimbabwe: Results From the Zimbabwe STI Etiology Study. *Sex Transm Dis* 2018; **45**(1): 61-8. 565. Muraguri N, Tun W, Okal J, et al. HIV and STI prevalence and risk factors among male sex workers and other men who have sex with men in nairobi, kenya. *Journal of Acquired Immune Deficiency Syndromes* 2015; **68**(1): 91-6. 566. Mussa A, Bame B, Chakona S, et al. Prevalence of chlamydia and gonorrhoea among patients presenting with genital ulcer disease in Botswana. *Sexual Health* 2024; **24**(4): 61. 567. Mussa A, Wynn A, Ryan R, et al. Prevalence of Chlamydia trachomatis and Neisseria gonorrhoeae infection and associated factors among asymptomatic pregnant women in Botswana. *International Journal of STD and AIDS* 2023; **34**(7): 448-56. 568. Mwakagile D, Swai AB, Sandström E, Urassa E, Biberfeld G, Mhalu FS. High frequency of sexually transmitted diseases among pregnant women in Dar es Salaam, Tanzania: need for intervention. *East Afr Med J* 1996; **73**(10): 675-8. 569. Mwaniki SW, Kaberia PM, Mugo PM, Palanee-Phillips T. Prevalence of five curable sexually transmitted infections and associated risk factors among tertiary student men who have sex with men in Nairobi, Kenya: a respondent-driven sampling survey. *Sexual Health* 2023; **20**(2): 105-17. 570. Mwatelah R, Peterson S, Bonner C, et al. Prevalence and mucosal impact of STIS in young women from Mombasa, Kenya with varying exposure to sex work. *Sexually Transmitted Infections* 2019; **95 (Supplement 1)**: A176-A7. 571. Nacht C, Agingu W, Otieno F, Odhiambo F, Mehta SD. Antimicrobial resistance patterns in Neisseria gonorrhoeae among male clients of a sexually transmitted infections clinic in Kisumu, Kenya. *Int J STD AIDS* 2020; **31**(1): 46-52. 572. Naidoo J, Samsunder N, Govender K, et al. Point-of-care testing for sexually transmitted infections in HIV prevention trials. *Sexually Transmitted Infections* 2017; **93 (Supplement 2)**: A133-A4. 573. Nair G, Celum C, Szydlo D, et al. Adherence, safety, and choice of the monthly dapivirine vaginal ring or oral emtricitabine plus tenofovir disoproxil fumarate for HIV pre-exposure prophylaxis among African adolescent girls and young women: a randomised, open-label, crossover trial. *The Lancet HIV* 2023; **10**(12): e779-e89. 574. Nakku-Joloba E, Mboowa G, Ssengooba W, et al. Prevalence and antimicrobial resistance profiles of Neisseria gonorrhea and Chlamydia trachomatis isolated from individuals attending STD clinics in Kampala, Uganda. *African Health Sciences* 2022; **22**: 62-71. 575. Nakubulwa S, Kaye DK, Bwanga F, Tumwesigye NM, Mirembe FM. Genital infections and risk of premature rupture of membranes in Mulago Hospital, Uganda: A case control study Womens Health. *BMC Research Notes* 2015; **8**(1). 576. Namkinga LA, Matee MI, Kivaisi AK, Moshiro C. Prevalence and risk factors for vaginal candidiasis among women seeking primary care for genital infections in Dar es Salaam, Tanzania. *East Afr Med J* 2005; **82**(3): 138-43. 577. Napierala S, Bair EF, Omollo OD, et al. High prevalence of STIs among men engaged in transactional sex and alcohol use in western Kenya: important implications for STI prevention interventions. *Sex Transm Infect* 2025; **101**(3): 183-6. 578. Nasah BT, Nguematcha R, Eyong M, Godwin S. Gonorrhea, Trichomonas and Candida Among Gravid and Nongravid Women in Cameroon. *International Journal of Gynecology & Obstetrics* 1980; **18**(1): 48-52. 579. Nasio JM, Nagelkerke NJ, Mwatha A, Moses S, Ndinya-Achola JO, Plummer FA. Genital ulcer disease among STD clinic attenders in Nairobi: association with HIV-1 and circumcision status. *Int J STD AIDS* 1996; **7**(6): 410-4. 580. Nathoo KJ, Latif AS, Trijssenaar JE. Aetiology of neonatal conjunctivitis in Harare. *Cent Afr J Med* 1984; **30**(7): 123-6. 581. Ndinya-Achola J, Ghee A, Kihara A, et al. High HIV prevalence, low condom use and gender differences in sexual behaviour among patients with STD-related complaints at a Nairobi primary health care clinic. *International journal of STD & AIDS* 1997; **8**(8): 506-14. 582. Ndoye I, Mboup S, De Schryver A, et al. Diagnosis of sexually transmitted infections in female prostitutes in Dakar, Senegal. *Sex Transm Infect* 1998; **74 Suppl 1**: S112-7. 583. Ndumbe PM, Watonsi E, Nyambi P, Mbaya P, Yanga D. Sexually transmitted infections in selected high risk populations in Cameroon [3]. *Genitourinary Medicine* 1992; **68**(3): 193-4. 584. Ngaroua, Eloundou NJ, Djibrilla Y, Asmaou O, Mbo AJ. Epidemiological, clinical aspects and management of urethral stenosis in adult patients in a district hospital in ngaoundere, Cameroon. [French]. *Pan African Medical Journal* 2017; **26 (no pagination)**. 585. Ngetsa CJ, Heymann MW, Thiong'o A, et al. Rectal gonorrhoea and chlamydia among men who have sex with men in coastal Kenya. *Wellcome Open Res* 2020; **4**. 586. Ngobese B, Swe Swe-Han K, Tinarwo P, Abbai NS. Low prevalence of macrolide resistance in Mycoplasma genitalium infections in a cohort of pregnant women living with human immunodeficiency virus. *International Journal of STD and AIDS* 2022; **33**(14): 1174-82. 587. Ngom NS, Gassama O, Dieng A, et al. Vaginal Carriage of Group B Streptococcus (GBS) in Pregnant Women, Antibiotic Sensitivity and Associated Risk Factors in Dakar, Senegal. *Microbiology Insights* 2023; **16**. 588. Nkuo-Akenji T, Nkwesheu A, Nyasa R, Tallah E, Ndip R, Angwafo IF. Knowledge of HIV/AIDS, sexual behaviour and pevalence of sexually transmitted infections among female students of the University of Buea, Cameroon. *African Journal of AIDS Research* 2007; **6**(2): 157-63. 589. Nkwabong E. What are the most common sexually transmitted bacteria in women with cervico-vaginitis nowadays? *Indian J Sex Transm Dis AIDS* 2020; **41**(1): 39-42. 590. Nkwabong E, Dingom MA. Acute Pelvic Inflammatory Disease in Cameroon: A Cross Sectional Descriptive Study. *African journal of reproductive health* 2015; **19**(4): 87-91. 591. Nkya WM, Gillespie SH, Howlett W, et al. Sexually transmitted diseases in prostitutes in Moshi and Arusha, Northern Tanzania. *Int J STD AIDS* 1991; **2**(6): 432-5. 592. Nnochiri E. DIAGNOSIS AND TREATMENT OF TRICHOMONAL URETHRITIS IN NIGERIAN MALES. *Br J Vener Dis* 1964; **40**(3): 191-3. 593. Notelovitz M. The antenatal detection of asymptomatic disease. *S Afr Med J* 1974; **48**(5): 178-84. 594. Nouaman MN, Coffie PA, Agoua AA, et al. Syndromic and biological screening for sexually transmitted infections in female sex workers in Côte d'Ivoire: the ANRS 12381 PRINCESSE cohort study. *Front Public Health* 2025; **13**: 1535122. 595. Nqou-Milama E, Cotand G. Sensitivity of N. gonorrhoeae to certain antibiotics. Penicillin resistant strains. *MED AFR NOIRE* 1980; **27**(10): 783-4. 596. Nsanze H, Fast MV, D'Costa LJ, Tukei P, Curran J, Ronald A. Genital ulcers in Kenya. Clinical and laboratory study. *Br J Vener Dis* 1981; **57**(6): 378-81. 597. Nsofor BI, Bello CS, Ekwempu CC. Sexually transmitted disease among women attending a family planning clinic in Zaria, Nigeria. *Int J Gynaecol Obstet* 1989; **28**(4): 365-7. 598. Nuwagaba-Biribonwoha H, Simelane S, Sithole T, et al. Feasibility and acceptability of point of care testing for sexually transmitted infections in outpatient clinics offering integrated services in Eswatini. *Sexually transmitted diseases* 2024. 599. Nwabueze RN, Onyia GOC, Adaelu IL. Incidence of acquired immune deficiency syndrome (AIDS) and other sexually transmitted diseases (STD) in Abia State, Nigeria. *International Journal of Environmental Health Research* 2000; **10**(3): 263-70. 600. Nwadike VU, Olusanya O, Anaedobe GC, Kalu I, Ojide KC. Patterns of sexually transmitted infections in patients presenting in special treatment clinic in Ibadan south western Nigeria. *Pan Afr Med J* 2015; **21**: 222. 601. Nwadioha S, Egah D, Nwokedi E, Onwuezobe I. A study of female genital swabs in primary health care centres in Jos, Nigeria. *Asian Pacific Journal of Tropical Disease* 2011; **1**(1): 52-4. 602. Nwadioha S, Egesie JO, Emejuo H, Iheanacho E. Prevalence of pathogens of abnormal vaginal discharges in a Nigerian tertiary hospital. *Asian Pacific Journal of Tropical Medicine* 2010; **3**(6): 483-5. 603. Nwokolo U. Two gram single dose amoxycillin in treatment of gonococcal and other urethritides. *Med J Zambia* 1975; **9**(4): 102-4. 604. Nyakambi M, Waruru A, Oladokun A. Prevalence of genital Chlamydia trachomatis among women of reproductive age attending outpatient clinic at Kisumu County Referral Hospital, Kenya, 2021. *J Public Health Afr* 2022; **13**(3). 605. Nzila N, Laga M, Thiam MA, et al. HIV and other sexually transmitted diseases among female prostitutes in Kinshasa. *AIDS* 1991; **5**(6): 715-21. 606. O'Farrell N, Hoosen AA, Kharsany ABM, Van den Ende J. Sexually transmitted pathogens in pregnant women in a rural South Africa community. *Genitourinary Medicine* 1989; **65**(4): 276-80. 607. O'Farrell N, Windsor I, Becker P. HIV-1 infection among heterosexual attenders at a sexually transmitted disease clinic in Durban. *South African Medical Journal* 1991; **80**(1): 17-20. 608. Obaseiki-Ebor EE, Oyaide SM, Okpere EE. Incidence of penicillinase producing Neisseria gonorrhoeae (PPNG) strains and susceptibility of gonococcal isolates to antibiotics in Benin City, Nigeria. *Genitourin Med* 1985; **61**(6): 367-70. 609. Oboho KO. Problems of venereal disease in Nigeria. 1. Gonococcal resistance to antibiotics and treatment of gonorrhoea. *Fam Pract* 1984; **1**(4): 219-21. 610. Odendaal HJ, Schoeman J, Grové D, et al. The association between Chlamydia trachomatis genital infection and spontaneous preterm labour. *South African Journal of Obstetrics and Gynaecology* 2006; **12**(3): 146-9. 611. Odongo EAI. The role of the venereal disease laboratory, Mulago Hospital, Kampala in the diagnosis of sexually transmitted diseases in Uganda. *East African Medical Journal* 1977; **54**(7): 385-92. 612. Odugbemi T, Oyewole F, Isichei CS, Onwukeme KE, Adeyemi-Doro FA. Single oral dose of azithromycin for therapy of susceptible sexually transmitted diseases: a multicenter open evaluation. *West African journal of medicine* 1993; **12**(3): 136-40. 613. Ogbonna CI, Ogbonna IB, Ogbonna AA, Anosike JC. Studies on the incidence of Trichomonas vaginalis amongst pregnant women in Jos area of Plateau State, Nigeria. *Angew Parasitol* 1991; **32**(4): 198-204. 614. Ogilvie GS, Mitchell S, Sekikubo M, et al. Results of a community-based cervical cancer screening pilot project using human papillomavirus self-sampling in Kampala, Uganda. *Int J Gynaecol Obstet* 2013; **122**(2): 118-23. 615. Ogunbanjo BO, Osoba AO. Trichomonal vaginitis in Nigerian women. *Trop Geogr Med* 1984; **36**(1): 67-70. 616. Ogunbanjo BO, Osoba AO, Ochei J. Infective factors of male infertility among Nigerians. *Afr J Med Med Sci* 1989; **18**(1): 35-8. 617. Ojezele MO, Oborephiada ON, Adedapo EA, Ojezele SO. A retrospective review of pid amongst women seen in gopc and their immediate outcomes at a health facility in nigeria between 2007-2017. *East African Medical Journal* 2018; **95**(9): 1927-34. 618. Okiring J, Getahun M, Gutin SA, et al. Sexual partnership concurrency and age disparities associated with sexually transmitted infection and risk behavior in rural communities in Kenya and Uganda. *Int J Infect Dis* 2022; **120**: 158-67. 619. Okonko I, Akinpelu A, Okerentugba P. Prevalence of sexually transmitted infections (STIs) among attendees of AFRH centre in Ibadan, Southwestern Nigeria. *Middle East J Sci Res* 2012; **11**: 24-31. 620. Okonofua FE, Ako-Nai KA, Dighitoghi MD. Lower genital tract infections in infertile Nigerian women compared with controls. *Genitourin Med* 1995; **71**(3): 163-8. 621. Okonofua FE, Snow RC, Alemnji GA, Okoruwa A, Ijaware CO. Serological and clinical correlates of gonorrhoea and syphilis in fertile and infertile Nigerian women. *Genitourin Med* 1997; **73**(3): 194-7. 622. Okunlola MA, Stella-Maris ON, Tokzaka AA, Ojengbede OA. Study on Vaginitis Among Intrauterine Contraceptive Device Users in Ibadan, South-western Nigeria. *J Reprod Contracept* 2009; **20**(4): 247-55. 623. Okwen PM, Mbangsi MZ, Okwen KTT. To conquer cervical and breast cancer in little charted waters: Synergistic and integrated approaches to identify early breast and cervical cancer in Cameroon. *Journal of clinical oncology : official journal of the American Society of Clinical Oncology* 2022; **40**(16_suppl): e17510-e. 624. Oladele AC, Mopelola DAA, Temitope OS. Prevalence of multi-drug resistant pathogen isolated from high vaginal swab in Nigeria. *AFRICAN JOURNAL OF MICROBIOLOGY RESEARCH* 2011; **5**(26): 4577-82. 625. Olakolu SS, Abioye-Kuteyi EA, Oyegbade OO. Sexually transmitted infections among patients attending the General Practice Clinic, Wesley Guild Hospital, Ilesa, Nigeria. *S Afr Fam Pract* 2011; **53**(1): 63-70. 626. Olaru ID, Chisenga M, Yeung S, et al. Sexually transmitted infections and prior antibiotic use as important causes for negative urine cultures among adults presenting with urinary tract infection symptoms to primary care clinics in Zimbabwe: a cross-sectional study. *BMJ Open* 2021; **11**(8): e050407. 627. Olayinka AT, Olayinka BO. Sexually transmitted diseases in children, a cause for concern [5]. *Journal of Tropical Pediatrics* 2002; **48**(5): 316. 628. Oliver VO, Otieno G, Gvetadze R, et al. High prevalence of sexually transmitted infections among women screened for a contraceptive intravaginal ring study, Kisumu, Kenya, 2014. *Int J STD AIDS* 2018; **29**(14): 1390-9. 629. Omolo M. 2018/2019 surveillance update on Neisseria gonorrhoeae isolates. *Sexually Transmitted Infections* 2019; **95 (Supplement 1)**: A359. 630. Omosa-Manyonyi GS, de Kam M, Tostmann A, et al. Evaluation and optimization of the syndromic management of female genital tract infections in Nairobi, Kenya. *BMC Infectious Diseases* 2023; **23**(1): 547. 631. Ondoa P, Gautam R, Rusine J, et al. Twelve-Month Antiretroviral Therapy Suppresses Plasma and Genital Viral Loads but Fails to Alter Genital Levels of Cytokines, in a Cohort of HIV-Infected Rwandan Women. *PLoS ONE* 2015; **10**(5): e0127201. 632. Ongom VL, Wamboka JW, Nakagwa E, Yiga A, Kamulegeya JK, Munafu C. The prevalence of venereal diseases among food and liquor-handlers in public places in Kampala, Uganda. *East Afr Med J* 1976; **53**(7): 389-97. 633. Ongom VL, Wamboka JW, Odong EAI, Mafigiri J. A single oral dose treatment of Trichomonas vaginalis infection with tinidazole (Fasigyn) in Uganda. *East African Medical Journal* 1974; **51**(12): 878-82. 634. Oni AA, Adu FD, Ekweozor CC. Isolation of herpes simplex virus from sexually transmitted disease patients in Ibadan, Nigeria. *Sex Transm Dis* 1994; **21**(4): 187-90. 635. Oni AA, Adu FD, Ekweozor CC, Bakare RA. Herpetic urethritis in male patients in Ibadan. *West Afr J Med* 1997; **16**(1): 27-9. 636. Onifade A, Osoba AO. Venereal disease among Nigerian women attending intra-uterine contraceptive device clinics. *J Trop Med Hyg* 1972; **75**(11): 213-6. 637. Onifade A, Osoba AO. Venereal diseases among women complaining of infertility. *WEST AFR MED J* 1975; **23**(2): 84-6. 638. Onigbogi O, Ojo O. Prevalence of sexually transmitted diseases among female patients presenting with hepatitis B virus and human immunodeficiency virus infections in a Nigerian teaching hospital. *Clinical Microbiology and Infection* 2009; **15**(S4): S585-S6. 639. Onoya D, Reddy P, Sifunda S, et al. Transactional sexual relationships, sexually transmitted infection risk, and condom use among young Black Women in peri-urban areas of the Western Cape Province of South Africa. *Womens Health Issues* 2012; **22**(3): e277-82. 640. Onyebueke EA, Udoh IP, Ezeli OF. Prevalence of urinary tract infection and antibiotic resistance pattern of isolates in benign prostatic hyperplasia patients with urinary catheter seen at a tertiary hospital in Enugu state, Nigeria. *Pharmacologyonline* 2020; **1**: 92-104. 641. Oree G, Naicker M, Maise HC, Tinarwo P, Ramsuran V, Abbai NS. Comparison of methods for the detection of Neisseria gonorrhoeae from South African women attending antenatal care. *International Journal of STD and AIDS* 2021; **32**(5): 396-402. 642. Oronsaye F. Novel laboratory diagnosis for neiserria gonococcus in male patients suspected of suffering from gonorrhea. *Clinical Chemistry and Laboratory Medicine (CCLM)* 2014; **52**(Supplement): S608. 643. Osegbe DN, Amaku EO. The causes of male infertility in 504 consecutive Nigerian patients. *Int Urol Nephrol* 1985; **17**(4): 349-58. 644. Osisanya JO, Sehgal SC, Iyanda A. Pattern of genito-urinary parasitic infections at the Teaching Hospital, Sokoto, Nigeria. *East Afr Med J* 1990; **67**(1): 51-7. 645. Osman NB, Folgosa E, Bergstrom S. An incident case-referent study of threatening preterm birth and genital infection. *Journal of Tropical Pediatrics* 1995; **41**(5): 267-72. 646. Osoba AO. Epidemiology of urethritis in Ibadan. *Br J Vener Dis* 1972; **48**(2): 116-20. 647. Osoba AO, Alausa KO. Vulvovaginitis in Nigerian children. *NIGERJPEDIAT* 1974; **1**(1): 26-32. 648. Osoba AO, Onifade A. Venereal diseases among pregnant women in Nigeria. *West Afr Med J Niger Med Dent Pract* 1973; **22**(1): 23-5. 649. Otieno F, Ng'ety G, Okall D, et al. Incident gonorrhoea and chlamydia among a prospective cohort of men who have sex with men in Kisumu, Kenya. *Sex Transm Infect* 2020; **96**(7): 521-7. 650. Otieno FO, Ndivo R, Oswago S, et al. Correlates of prevalent sexually transmitted infections among participants screened for an HIV incidence cohort study in Kisumu, Kenya. *Int J STD AIDS* 2015; **26**(4): 225-37. 651. Oware K, Adiema L, Rono B, et al. Characteristics of Kenyan women using HIV PrEP enrolled in a randomized trial on doxycycline postexposure prophylaxis for sexually transmitted infection prevention. *BMC Women's Health* 2023; **23**(1): 296. 652. Ozoh PTE, Atenaga PU. Sexually transmitted diseases in Bauchi, Nigeria. *International Journal of Environmental Health Research* 1993; **3**(4): 185-9. 653. Ozumba BC, Megafu U. Pattern of vulval warts at the University of Nigeria Teaching Hospital, Enugu, Nigeria. *Int J Gynaecol Obstet* 1991; **34**(4): 347-52. 654. Palanee-Phillips T, Schwartz K, Brown ER, et al. Characteristics of women enrolled into a randomized clinical trial of dapivirine vaginal ring for HIV-1 prevention. *PLoS ONE* 2015; **10**(6). 655. Palanee-Phillips T, Tenza S, Moodley K, et al. Baseline socio-demographic characteristics associated with HIV prevalence rates among MSM in the TRANSFORM study, Johannesburg, South Africa. *AIDS Research and Human Retroviruses* 2018; **34 (Supplement 1)**: 232. 656. Paz-Bailey G, Rahman M, Chen C, et al. Changes in the etiology of sexually transmitted diseases in botswana between 1993 and 2002: Implications for the clinical management of genital ulcer disease. *Clinical Infectious Diseases* 2005; **41**(9): 1304-12. 657. Pedersen Sheller J, Johannessen G, Olsen T, Maruping S. Neisseria gonorrhoeae, Trichomonas vaginalis, and yeast reported from attenders at an antenatal clinic in a rural area in Botswana. *Genitourinary Medicine* 1990; **66**(6): 460. 658. Peeling RW, Kimani J, Plummer F, et al. Antibody to chlamydial hsp60 predicts an increased risk for chlamydial pelvic inflammatory disease. *J Infect Dis* 1997; **175**(5): 1153-8. 659. Peer AD, Kagaayi J, Ssekubugu R, et al. HIV, STIs and pregnancy among women of reproductive age in a Lake Victoria fishing community: A populationbased study. *Journal of the International AIDS Society Conference: 23rd International AIDS Conference Virtual* 2020; **23**(SUPPL 4). 660. Pépin J, Deslandes S, Khonde N, et al. Low prevalence of cervical infections in women with vaginal discharge in west Africa: implications for syndromic management. *Sex Transm Infect* 2004; **80**(3): 230-5. 661. Pépin J, Labbé AC, Khonde N, et al. Mycoplasma genitalium: an organism commonly associated with cervicitis among west African sex workers. *Sex Transm Infect* 2005; **81**(1): 67-72. 662. Pepin J, Sobéla F, Deslandes S, et al. Etiology of urethral discharge in West Africa: the role of Mycoplasma genitalium and Trichomonas vaginalis. *Bulletin of the World Health Organization* 2001; **79**(2): 118-26. 663. Pépin J, Sobela F, Khonde N, et al. The syndromic management of vaginal discharge using single-dose treatments: a randomized controlled trial in West Africa. *Bull World Health Organ* 2006; **84**(9): 729-38. 664. Perine PL, Biddle JW, Nsanze H, D'Costa LJ, Osaba AO, Widy-Wirski R. Gonococcal drug resistance and treatment of gonorrhoea in Nairobi. *East Afr Med J* 1980; **57**(4): 238-46. 665. Perine PL, Duncan ME, Krause DW, Awoke S. Pelvic inflammatory disease and puerperal sepsis in Ethiopia. I. Etiology. *Am J Obstet Gynecol* 1980; **138**(7 Pt 2): 969-73. 666. Perine PL, Totten PA, Knapp JS, Holmes KK, Bentsi C, Klufio CA. Diversity of gonococcal plasmids, auxotypes, and serogroups in Ghana. *Lancet* 1983; **1**(8332): 1051-2. 667. Peters R, Radebe O, Hamiwe T, et al. High rate of repeat sexually transmitted infections among men who have sex with men in South Africa. *Sexually Transmitted Infections* 2017; **93 (Supplement 2)**: A164. 668. Peters RP, Dubbink JH, van der Eem L, et al. Cross-sectional study of genital, rectal, and pharyngeal Chlamydia and gonorrhea in women in rural South Africa. *Sex Transm Dis* 2014; **41**(9): 564-9. 669. Peters RPH, Adamson PC, Daniels J, et al. Evaluation of a lateral flow assay for point-of-care detection of Neisseria gonorrhoeae infection in asymptomatic individuals in South Africa. *Sexually Transmitted Diseases* 2024b; **51**(1 Supplement 1): S142. 670. Peters RPH, Klausner JD, Mazzola L, et al. Novel lateral flow assay for point-of-care detection of Neisseria gonorrhoeae infection in syndromic management settings: a cross-sectional performance evaluation. *The Lancet* 2024a; **403**: 657-64. 671. Pettifor A, Delany S, Kleinschmidt I, Miller WC, Atashili J, Rees H. Use of injectable progestin contraception and risk of STI among South African women. *Contraception* 2009; **80**(6): 555-60. 672. Pettifor AE, Kleinschmidt I, Levin J, et al. A community-based study to examine the effect of a youth HIV prevention intervention on young people aged 15-24 in South Africa: results of the baseline survey. *Trop Med Int Health* 2005; **10**(10): 971-80. 673. Pettifor AE, Turner AN, Van Damme K, et al. Increased risk of chlamydial and gonococcal infection in adolescent sex workers in Madagascar. *Sex Transm Dis* 2007; **34**(7): 475-8. 674. Phillips I, Fernandes R, Pirani AA, Wagaine D. Antibiotic sensitivity of gonococci in Kampala. *East Afr Med J* 1969; **46**(1): 38-45. 675. Pichard E, Toure A, Soula G, Traore HA, Samake S. Fitz-Hugh-Curtis syndrome in Mali. About 11 cases. [French]. *Medecine et Maladies Infectieuses* 1990; **20**(11): 555-8. 676. Pickering JM, Whitworth JA, Hughes P, et al. Aetiology of sexually transmitted infections and response to syndromic treatment in southwest Uganda. *Sex Transm Infect* 2005; **81**(6): 488-93. 677. Pillay DG, Hoosen AA, Vezi B, Moodley C. Diagnosis of Trichomonas vaginalis in male urethritis. *Trop Geogr Med* 1994; **46**(1): 44-5. 678. Piot P, Plummer FA, Rey MA, et al. Retrospective seroepidemiology of AIDS virus infection in Nairobi populations. *J Infect Dis* 1987; **155**(6): 1108-12. 679. Price MA, Kuteesa M, Oladimeji M, et al. High STI burden among a cohort of adolescents aged 12-19 years in a youthfriendly clinic in South Africa. *PLoS ONE* 2024; **19**(7 July): e0306771. 680. Price MA, Zimba D, Hoffman IF, et al. Addition of treatment for trichomoniasis to syndromic management of urethritis in Malawi: A randomized clinical trial. Sexually Transmitted Diseases; 2003; 2003. p. 516-22. 681. Priddy FH, Wakasiaka S, Hoang TD, et al. Anal sex, vaginal practices, and HIV incidence in female sex workers in Urban Kenya: Implications for the development of intravaginal HIV prevention methods. *AIDS Research and Human Retroviruses* 2011; **27**(10): 1067-72. 682. Quincke G. [Sensitivity to penicillin of gonococcal strains isolated in Lomé, Togo]. *Z Tropenmed Parasitol* 1970; **21**(4): 433-7. 683. Rambaran S, Naidoo K, Dookie N, Moodley P, Sturm AW. Resistance Profile of Neisseria gonorrhoeae in KwaZulu-Natal, South Africa Questioning the Effect of the Currently Advocated Dual Therapy. *Sex Transm Dis* 2019; **46**(4): 266-70. 684. Ramuthaga TN, Mahomed FM, Greeff AS, CreweBrown HH, Vermeulen R. Comparison of urine with urethral swabs for the detection of Chlamydia trachomatis in men attending an STD clinic. *SOUTH AFRICAN MEDICAL JOURNAL* 1995; **85**(12): 1287-9. 685. Rassjo EB, Kambugu F, Tumwesigye MN, Tenywa T, Darj E. Prevalence of sexually transmitted infections among adolescents in Kampala, Uganda, and theoretical models for improving syndromic management. *Journal of Adolescent Health* 2006; **38**(3): 213-21. 686. Råssjö EB, Mirembe F, Darj E. Self-reported sexual behaviour among adolescent girls in Uganda: reliability of data debated. *Afr Health Sci* 2011; **11**(3): 383-9. 687. Ratnam AV, Chatterjee TK, Mulenga RC. Sexually transmitted diseases in pregnant women in Lusaka. *Med J Zambia* 1980a; **14**(5): 75-8. 688. Ratnam AV, Din SN, Chatterjee TK. Gonococcal infection in women with pelvic inflammatory disease in Lusaka, Zambia. *Am J Obstet Gynecol* 1980b; **138**(7 Pt 2): 965-8. 689. Rebe K, Lewis D, Myer L, et al. A Cross Sectional Analysis of Gonococcal and Chlamydial Infections among Men-Who-Have-Sex-with-Men in Cape Town, South Africa. *PLoS ONE* 2015; **10**(9): e0138315. 690. Richardson BA, Lavreys L, Martin HL, et al. Evaluation of a low-dose nonoxynol-9 gel for the prevention of sexually transmitted diseases - A randomized clinical trial. *SEXUALLY TRANSMITTED DISEASES* 2001; **28**(7): 394-400. 691. Riedner G, Rusizoka M, Hoffmann O, et al. Baseline survey of sexually transmitted infections in a cohort of female bar workers in Mbeya Region, Tanzania. *Sexually Transmitted Infections* 2003; **79**(5): 382-7. 692. Rietmeijer CA, Mungati M, Machiha A, et al. The Etiology of Male Urethral Discharge in Zimbabwe: Results from the Zimbabwe STI Etiology Study. *Sex Transm Dis* 2018; **45**(1): 56-60. 693. Roddy RE, Zekeng L, Ryan KA, Tamoufé U, Tweedy KG. Effect of nonoxynol-9 gel on urogenital gonorrhea and chlamydial infection -: A randomized controlled trial. *JAMA-JOURNAL OF THE AMERICAN MEDICAL ASSOCIATION* 2002; **287**(9): 1117-22. 694. Romoren M, Rahman M, Sundby J, Hjortdahl P. Chlamydia and gonorhea in pregnancy: Effectiveness of diagnosis and treatment in Botswana. *Sexually Transmitted Infections* 2004; **80**(5): 395-400. 695. Ross MW, Nyoni J, Ahaneku HO, Mbwambo J, McClelland RS, McCurdy SA. High HIV seroprevalence, rectal STIs and risky sexual behaviour in men who have sex with men in Dar es Salaam and Tanga, Tanzania. *BMJ Open* 2014; **4**(8): e006175. 696. Ross S, * Windsor, IM,* Robins-Browne, RM,** Ballard, RC,*** Adhikari, M.*, Fenn D. Microbiological studies during the perinatal period-an attempt to correlate selected bacterial and viral infections with intra-uterine deaths and preterm labour. *South african medical journal* 1984; **66**(16): 598-603. 697. Rotimi VO, Somorin AO. Sexually transmitted diseases in clinic patients in Lagos. *Br J Vener Dis* 1980; **56**(1): 54-6. 698. Roudiere JL. Decreased fertility in Gabon. Role for female genital tract infections. [French]. *Revue Francaise de Gynecologie et d'Obstetrique* 1998; **93**(6): 452-6. 699. Rubin A. Vaginal discharge in the black pregnanyt patient-an evaluation of the relationship between symptomatology and diagnosis. *South African Medical Journal* 1980; **57**(11): 404-6. 700. Rustomjee R, Kharsany AB, Connolly CA, Karim SS. A randomized controlled trial of azithromycin versus doxycycline/ciprofloxacin for the syndromic management of sexually transmitted infections in a resource-poor setting. *J Antimicrob Chemother* 2002; **49**(5): 875-8. 701. Rutherford GW, Anglemyer A, Bagenda D, et al. University students and the risk of HIV and other sexually transmitted infections in Uganda: the Crane survey. *Int J Adolesc Med Health* 2014; **26**(2): 209-15. 702. Rutstein SE, Chen JS, Jere E, et al. High incidence of curable sexually transmitted infections among persons accessing oral PrEP at an integrated sexually transmitted infection/PrEP clinic in Lilongwe, Malawi. *Sexually Transmitted Diseases* 2025: 10.1097/OLQ.0000000000002170. 703. Ryan KA, Zekeng L, Roddy RE, Weir SS. Prevalence and prediction of sexually transmitted diseases among sex workers in Cameroon. *International Journal of STD and AIDS* 1998; **9**(7): 403-7. 704. Sabeta CT, Gopo JM. EPIDEMIOLOGIC SURVEY OF PENICILLINASE-PRODUCING NEISSERIA-GONORRHEA (PPNG) IN ZIMBABWE. *DISCOVERY AND INNOVATION* 1994; **6**(4): 346-53. 705. Sagar M, Lavreys L, Baeten JM, et al. Identification of modifiable factors that affect the genetic diversity of the transmitted HIV-1 population. *AIDS* 2004; **18**(4): 615-9. 706. Sahile A, Teshager L, Fekadie M, Gashaw M. Prevalence and Antimicrobial Susceptibility Patterns of Neisseria gonorrhoeae among Suspected Patients Attending Private Clinics in Jimma, Ethiopia. *Int J Microbiol* 2020; **2020**. 707. Saleh-Onoya D, Reddy PS, Ruiter RA, Sifunda S, Wingood G, van den Borne B. Condom use promotion among isiXhosa speaking women living with HIV in the Western Cape Province, South Africa: a pilot study. *AIDS Care* 2009; **21**(7): 817-25. 708. Sanders CJG, Mulder MMM. Periurethral gland abscess: Aetiology and treatment. *Sexually Transmitted Infections* 1998; **74**(4): 276-8. 709. Sanders EJ, Okuku HS, Smith AD, et al. High HIV-1 incidence, correlates of HIV-1 acquisition, and high viral loads following seroconversion among MSM. *AIDS* 2013; **27**(3): 437-46. 710. Sanon PS, Gershy-Damet GM, M'Boup S, et al. [Prevalence of Chlamydia trachomatis in genital samples from Abidjan]. *Bull Soc Pathol Exot* 1992; **85**(3): 209-11. 711. Sarenje KL, Ngalamika O, Maimbolwa MC, Siame A, Munsaka SM, Kwenda G. Antimicrobial resistance of Neisseria gonorrhoeae isolated from patients attending sexually transmitted infection clinics in Urban Hospitals, Lusaka, Zambia. *BMC Infectious Diseases* 2022; **22**(1): 688. 712. Sarr M, Gueye D, Mboup A, et al. Uptake, retention, and outcomes in a demonstration project of pre-exposure prophylaxis among female sex workers in public health centers in Senegal. *International Journal of STD and AIDS* 2020; **31**(11): 1063-72. 713. Sarrat H. [Male urethritis at Dakar (epidemiological and biological aspects)]. *Bull Soc Med Afr Noire Lang Fr* 1973; **18**(4): 581-6. 714. Sarrat H. [Female genital infections at Dakar (preliminary note)]. *Bull Soc Med Afr Noire Lang Fr* 1974a; **19**(1): 29-32. 715. Sarrat H, Ridet J, Deubel V. [Results of an etiological survey of female genital infections in rural areas of Senegal]. *Bull Soc Med Afr Noire Lang Fr* 1974b; **19**(1): 76-9. 716. Saywon D, Beddoe A, Dottino P, Murphy J, Jallah W, King S. CERVICAL CANCER AND STI SCREENING IN LIBERIA: COMPARISON OF CURRENTLY USED CERVICAL CANCER SCREENING METHODS. CERVICAL CANCER AND SEXUALLY TRANSMITTED INFECTIONS SCREENING IN LIBERIA: COMPARISON OF CURRENTLY USED CERVICAL CANCER SCREENING METHODS. *International Journal of Gynecological Cancer* 2024; **34**(Supplement 3): A276 EP - A7. 717. Schneider H, Coetzee DJ, Fehler HG, et al. Screening for sexually transmitted diseases in rural South African women. *Sex Transm Infect* 1998; **74 Suppl 1**: S147-52. 718. Schonfeld A, Feldt T, Tufa TB, et al. Prevalence and impact of sexually transmitted infections in pregnant women in central Ethiopia. *International Journal of STD and AIDS* 2018; **29**(3): 251-8. 719. Schröder D, Sorano S, Shipitsyna E, et al. Prevalence and epidemiology of Mycoplasma genitalium and the absence of macrolide resistance in M. genitalium among pregnant women attending antenatal care in Zambia. *Front Public Health* 2025; **13**: 1576376. 720. Sehgal SC. Epidemiology of male urethritis in Nigeria. *J Trop Med Hyg* 1990a; **93**(2): 151-2. 721. Sehgal SC, Nalini V. The role and prevalence of Gardnerella vaginalis in anaerobic vaginosis. *Infection* 1990b; **18**(2): 83-5. 722. Senkoro RH, Juma H, Namkinga L. Prevalence and Risk factors associated with Sexually Transmitted Infections among Women of reproductive age attending reproductive and child health clinics in Dodoma and Dar es Salaam Tanzania. *Tanzania Journal of Health Research* 2024; **25**(1): 642-55. 723. Serafino X, Cave L, Tossou H. [Urethral stricture in African men in Dakar (clinical and radiologic study)]. *J Urol Nephrol (Paris)* 1969; **75**(3): 141-65. 724. Sewpershad NB, Venter I, Gumede L, et al. Relative prevalence of STI pathogens, vaginal conditions and HIV co-infection among STI patients attending Alexandra Health Centre, Gauteng Province, South Africa (2011-2013). *International Journal of Infectious Diseases* 2014; **21**: 423. 725. Shephard M, Matthews S, Kularatne R, et al. Independent clinic-based evaluation of point-of-care testing for the screening of Chlamydia trachomatis, Neisseria gonorrhoea and Trichomonas vaginalis in women-at-risk in Australia, Guatemala, Morocco, and South Africa. *BMC Infectious Diseases* 2024; **24**(Supplement 1): 277. 726. Simms V, Bandason T, Dauya E, Chikwari CD, Ferrand RA, Kranzer K. STI prevalence among young adults diagnosed with HIV in childhood and adulthood: A cross-sectional survey in Zimbabwe. *Sexually Transmitted Diseases* 2024; **51**(1 Supplement 1): S177. 727. Simpson JA, Oliver SP. Beta-lactamase-producing isolates of Neisseria gonorrhoeae in Cape Town. *S Afr Med J* 1986; **69**(5): 307-8. 728. Sineque A, Ceffa S, Parruque F, et al. Impact of STIs on cervical cancer screening: Prevalence of Chlamydia trachomatis and Neisseria gonorrhoeae in visual inspection with acetic acid (VIA) positive women in Mozambique. *Int J STD AIDS* 2024; **35**(13): 1019-24. 729. Singa B, Glick SN, Bock N, et al. Sexually transmitted infections among HIV-infected adults in HIV care programs in Kenya: a national sample of HIV clinics. *Sex Transm Dis* 2013; **40**(2): 148-53. 730. Sivapalasingam S, McClelland RS, Ravel J, et al. An effective intervention to reduce intravaginal practices among HIV-1 uninfected Kenyan women. *AIDS Research and Human Retroviruses* 2014; **30**(11): 1046-54. 731. Skoler-Karpoff S, Ramjee G, Ahmed K, et al. Efficacy of Carraguard for prevention of HIV infection in women in South Africa: a randomised, double-blind, placebo-controlled trial. *Lancet* 2008; **372**(9654): 1977-87. 732. Smith AD, Fearon E, Kabuti R, et al. Disparities in HIV/STI burden and care coverage among men and transgender persons who have sex with men in Nairobi, Kenya: A cross-sectional study. *BMJ Open* 2021; **11**(12). 733. Sobéla F, Pépin J, Gbéléou S, et al. A tale of two countries: HIV among core groups in Togo. *JAIDS Journal of Acquired Immune Deficiency Syndromes* 2009; **51**(2): 216-23. 734. Søfteland S, Sebitloane M, Galappaththi-Arachchige HN, et al. Diagnosis of female genital schistosomiasis and other genital infections in young South African women: challenges in the syndromic approach. *Front Trop Dis* 2024; **5**. 735. Sogbetun AO, Alausa KO, Osoba AO. Sexually transmitted diseases in Ibadan, Nigeria. *Br J Vener Dis* 1977; **53**(3): 155-60. 736. Somorin AO, Orebiyi HI. Microbiological study of sexually transmitted diseases among out-patients in a Nigerian hospital. *Cent Afr J Med* 1981; **27**(3): 42-4. 737. Sow AI, Cisse MF, Samb A. [Bacteriology of vulvo-vaginitis in pediatrics]. *Dakar Med* 1996; **41**(2): 125-7. 738. Steen R, Vuylsteke B, DeCoito T, et al. Evidence of declining STD prevalence in a South African mining community following a core-group intervention. *Sex Transm Dis* 2000; **27**(1): 1-8. 739. Stranix-Chibanda L, Hamilton EL, Ngo J, et al. Safety, tolerability, and acceptability of long-acting injectable cabotegravir for HIV prevention in cisgender female adolescents (HPTN 084-01): a single-arm, open-label, phase 2b trial. *Lancet HIV* 2025; **12**(4): e252-e60. 740. Sturm AW, Wilkinson D, Ndovela N, Bowen S, Connolly C. Pregnant women as a reservoir of undetected sexually transmitted diseases in rural South Africa: implications for disease control. *American Journal of Public Health* 1998; **88**(8): 1243-5. 741. Sturm PD, Connolly C, Khan N, Ebrahim S, Sturm AW. Vaginal tampons as specimen collection device for the molecular diagnosis of non-ulcerative sexually transmitted infections in antenatal clinic attendees. *Int J STD AIDS* 2004a; **15**(2): 94-8. 742. Sturm PDJ, Moodley P, Khan N, et al. Aetiology of male urethritis in patients recruited from a population with a high HIV prevalence. *International Journal of Antimicrobial Agents* 2004b; **24**: 8-14. 743. Sturm PDJ, Moodley P, Nzimande G, Balkistan R, Connolly C, Sturm AW. Diagnosis of bacterial vaginosis on self-collected vaginal tampon specimens. *International Journal of STD and AIDS* 2002; **13**(8): 559-63. 744. Sturm-Ramirez K, Brumblay H, Diop K, et al. Molecular epidemiology of genital Chlamydia trachomatis infection in high-risk women in Senegal, West Africa. *Journal of Clinical Microbiology* 2000; **38**(1): 138-45. 745. Sturt AS, Webb EL, Himschoot L, et al. Association of Female Genital Schistosomiasis with the Cervicovaginal Microbiota and Sexually Transmitted Infections in Zambian Women. *Open Forum Infectious Diseases* 2021; **8**(9): ofab438. 746. Tadele A, Hussen S, Shimelis T. Prevalence and associated factors of Chlamydia trachomatis and Neisseria gonorrhoeae among female commercial sex workers in Hawassa City, Southern Ethiopia. *BMC Infect Dis* 2019; **19**(1): 61. 747. Tadesse A, Mekonnen A, Kassu A, Asmelash T. Antimicrobial sensitivity of Neisseria gonorrhoea in Gondar, Ethiopia. *East Afr Med J* 2001; **78**(5): 259-61. 748. Tadesse E, Teshome M, Amsalu A, Shimelis T. Genital Chlamydia trachomatis Infection among Women of Reproductive Age Attending the Gynecology Clinic of Hawassa University Referral Hospital, Southern Ethiopia. *PLoS ONE* 2016; **11**(12): e0168580. 749. Taffa N, Bjune G, Sundby J, Gaustad P, Alestrøm A. Prevalence of gonococcal and chlamydial infections and sexual risk behavior among youth in Addis Ababa, Ethiopia. *Sex Transm Dis* 2002; **29**(12): 828-33. 750. Tafuma TA, Merrigan MB, Okui LA, et al. HIV/sexually transmitted infection prevalence and sexual behavior of men who have sex with men in 3 districts of botswana: Results from the 2012 biobehavioral survey. *Sexually Transmitted Diseases* 2014; **41**(8): 480-5. 751. Taha TE, Dallabetta GA, Hoover DR, et al. Trends of HIV-1 and sexually transmitted diseases among pregnant and postpartum women in urban Malawi. *AIDS* 1998; **12**(2): 197-203. 752. Taku O, Brink A, Meiring TL, et al. Detection of sexually transmitted pathogens and co-infection with human papillomavirus in women residing in rural Eastern Cape, South Africa. *PeerJ* 2021; **9 (no pagination)**. 753. Takuva S, Mugurungi O, Mutsvangwa J, et al. Etiology and antimicrobial susceptibility of pathogens responsible for urethral discharge among men in Harare, Zimbabwe. *Sex Transm Dis* 2014; **41**(12): 713-7. 754. Tamrat R, Kasa T, Sahilemariam Z, Gashaw M. Prevalence and Factors Associated with Sexually Transmitted Infections among Jimma University Students, Southwest Ethiopia. *Int J Microbiol* 2020; **2020**. 755. Tann CJ, Mpairwe H, Morison L, et al. Lack of effectiveness of syndromic management in targeting vaginal infections in pregnancy in Entebbe, Uganda. *Sex Transm Infect* 2006; **82**(4): 285-9. 756. Taylor-Robinson D, Ballard RC, Thomas BJ, Renton A. STD/HIV control in Malawi. *Genitourin Med* 1995; **71**(3): 202. 757. Taylor-Robinson D, Jensen JS, Fehler G, Radebe F, Ballard RC. Observations on the microbiology of urethritis in black South African men. *Int J STD AIDS* 2002; **13**(5): 323-5. 758. Temmerman M, Chomba EN, Ndinya-Achola J, Plummer FA, Coppens M, Piot P. Maternal human immunodeficiency virus-1 infection and pregnancy outcome. *Obstet Gynecol* 1994; **83**(4): 495-501. 759. Temmerman M, Gilks CF, Sanghvi HCG. Spontaneous and induced abortions at Kenyatta national hospital, Nairobi, Kenya [2]. *International Journal of Gynecology and Obstetrics* 1993; **41**(2): 182-3. 760. Temmerman M, Laga M, Ndinya-Achola JO, et al. Microbial aetiology and diagnostic criteria of postpartum endometritis in Nairobi, Kenya. *Genitourin Med* 1988; **64**(3): 172-5. 761. Temmerman M, Lopita MI, Sanghvi HC, Sinei SK, Plummer FA, Piot P. The role of maternal syphilis, gonorrhoea and HIV-1 infections in spontaneous abortion. *Int J STD AIDS* 1992a; **3**(6): 418-22. 762. Temmerman M, Plummer FA, Farah A, Wamola IA, Brunham RC, Piot P. Gonorrhoea in pregnancy. *Journal of Obstetrics and Gynaecology* 1992b; **12**(3): 162-6. 763. Thigpen MC, Kebaabetswe PM, Paxton LA, et al. Antiretroviral preexposure prophylaxis for heterosexual HIV transmission in Botswana. *New England Journal of Medicine* 2012; **367**(5): 423-34. 764. Thivalapill N, Jasumback CL, Perry SH, et al. Predicting Sexually Transmitted Infections Among HIV+ Adolescents and Young Adults: A Novel Risk Score to Augment Syndromic Management in Eswatini. *J Acquir Immune Defic Syndr* 2020; **85**(5): 543-52. 765. Thomas T, Choudhri S, Kariuki C, Moses S. Identifying cervical infection among pregnant women in Nairobi, Kenya: limitations of risk assessment and symptom-based approaches. *Genitourin Med* 1996; **72**(5): 334-8. 766. Tibebu M, Shibabaw A, Medhin G, Kassu A. Neisseria gonorrhoeae non-susceptible to cephalosporins and quinolones in Northwest Ethiopia. *BMC Infect Dis* 2013; **13**: 415. 767. Tobias S, Shonhiwa S, Norbert F, et al. Microbiological surveillance for sexually transmitted infections in Windhoek and Oshakati, Namibia (2007). *Sexually Transmitted Infections* 2011; **87**: A103. 768. Tobin L, Guerra L, Ahouanvoeke L, et al. Is it time to use nucleic acid amplification tests for identification of persons with sexually transmitted infections?: evidence from seroprevalence and behavioral epidemiology risk surveys in men with chlamydia and gonorrhea. *Pan Afr Med J* 2020; **36**: 299. 769. Todd J, Changalucha J, Ross DA, et al. The sexual health of pupils in years 4 to 6 of primary schools in rural Tanzania. *Sexually Transmitted Infections* 2004; **80**(1): 35-42. 770. Tounkara FK, Teguete I, Guedou FA, et al. Human papillomavirus genotype distribution and factors associated among female sex workers in West Africa. *PLoS ONE* 2020; **15**(11 November). 771. Tovo SF, Zohoncon TM, Dabire AM, et al. Molecular epidemiology of human papillomaviruses, neisseria gonorrhoeae, chlamydia trachomatis and mycoplasma genitalium among female sex workers in burkina faso: Prevalence, coinfections and drug resistance genes. *Tropical Medicine and Infectious Disease* 2021; **6**(2). 772. Trout CH, Dembele O, Diakite D, et al. West African female sex workers in Mali: Reduction in HIV prevalence and differences in risk profiles of sex workers of differing nationalities of origin. *Journal of Acquired Immune Deficiency Syndromes* 2015; **68**(Supplement 2): S221-S31. 773. Truong HHM, Otieno B, Kadede K, et al. Prevalence of chlamydia and gonorrhoea among Kenyan adolescents. *Sexually Transmitted Infections* 2025: sextrans-2024-056333. 774. Tsai AY, Dueger E, Macalino GE, et al. The U.S. military's Neisseria gonorrhoeae resistance surveillance initiatives in selected populations of five countries. *Msmr* 2013; **20**(2): 25-7. 775. Tswana SA, Nyström L, Moyo SR, et al. Hospital-based study of sexually transmitted diseases at Murewa rural district hospital, Zimbabwe 1991-1992. *Sex Transm Dis* 1995; **22**(1): 1-6. 776. Tun W, Vu L, Adebajo SB, et al. Population-based prevalence of hepatitis B and C virus, HIV, syphilis, gonorrhoea and chlamydia in male injection drug users in Lagos, Nigeria. *Int J STD AIDS* 2013; **24**(8): 619-25. 777. Twahirwa Rwema JO, Herbst S, Hamill MM, et al. Cross-sectional assessment of determinants of STIs among men who have sex with men and transgender women in Kigali, Rwanda. *Sexually Transmitted Infections* 2021. 778. Tyndall MW, Kidula N, Sande J, Ombette J, Temmerman M. Predicting Neisseria gonorrhoeae and Chlamydia trachomatis infection using risk scores, physical examination, microscopy, and leukocyte esterase urine dipsticks among asymptomatic women attending a family planning clinic in Kenya. *Sex Transm Dis* 1999; **26**(8): 476-82. 779. Tyndall MW, Nasio J, Maitha G, et al. Leukocyte esterase urine strips for the screening of men with urethritis--use in developing countries. *Genitourin Med* 1994; **70**(1): 3-6. 780. Usanga VU, Abia-Bassey L, Inyang-Etoh PC, Udoh SM, Ani F, Archibong E. Prevalence of sexually transmitted diseases in pregnant and non-pregnant women in Calabar, cross river state, Nigeria. *Internet Journal of Gynecology and Obstetrics* 2011; **14**(2). 781. Vallely A, Kasindi S, Hambleton IR, et al. Microbicides development program, Tanzania-baseline characteristics of an occupational cohort and reattendance at 3 months. *Sex Transm Dis* 2007; **34**(9): 638-43. 782. Van de Perre P, Lepage P, Kestelyn P, et al. Acquired immunodeficiency syndrome in Rwanda. *The Lancet* 1984; **324**(8394): 62-5. 783. Van De Velden L, Jancloes M, Mboup S. Prevalence of female gonococcosis in urban areas of Pikine (Senegal) and sensitivity to antibiotics. *MED AFR NOIRE* 1982; **29**(1): 47-53. 784. van de Wijgert J, Altini L, Jones H, et al. Two methods of self-sampling compared to clinician sampling to detect reproductive tract infections in Gugulethu, South Africa. *Sex Transm Dis* 2006; **33**(8): 516-23. 785. van de Wijgert JH, Morrison CS, Brown J, et al. Disentangling contributions of reproductive tract infections to HIV acquisition in African Women. *Sex Transm Dis* 2009; **36**(6): 357-64. 786. van der Veer C, Kondoni C, Kuyere A, et al. Prevalence of sexually transmitted infection in pregnancy and their association with adverse birth outcomes: a case–control study at Queen Elizabeth Central Hospital, Blantyre, Malawi. *Sexually Transmitted Infections* 2024. 787. van Hall MA, Petit PL, van Hall HN, Mouton RP, Ndinya-Achola JO. Prevalence of resistance of N. gonorrhoeae to penicillin and three other antibiotics in a rural area in Kenya. *East Afr Med J* 1991; **68**(11): 853-9. 788. van Liere G, Kock MM, Radebe O, et al. High Rate of Repeat Sexually Transmitted Diseases Among Men Who Have Sex With Men in South Africa: A Prospective Cohort Study. *Sex Transm Dis* 2019; **46**(11): e105-e7. 789. van Rensburg HJ, Odendaal HJ. The prevalence of potential pathogenic micro-organisms in the endocervix of pregnant women at Tygerberg Hospital. *S Afr Med J* 1992; **81**(3): 156-7. 790. Vandepitte J, Bukenya J, Weiss HA, et al. HIV and other sexually transmitted infections in a cohort of women involved in high-risk sexual behavior in Kampala, Uganda. *Sex Transm Dis* 2011; **38**(4): 316-23. 791. Vandepitte JM, Malele F, Kivuvu DM, et al. HIV and other sexually transmitted infections among female sex workers in Kinshasa, Democratic Republic of Congo, in 2002. *Sex Transm Dis* 2007; **34**(4): 203-8. 792. Verhagen AR, Van der Ham M, Heimans AL, Kranendonk O, Maina AN. Diminished antibiotic sensitivity of Neisseria gonorrhoeae in urban and rural areas in Kenya. *Bull World Health Organ* 1971; **45**(6): 707-17. 793. Verwijs MC, Agaba SK, Sumanyi JC, et al. Targeted point-of-care testing compared with syndromic management of urogenital infections in women (WISH): a cross-sectional screening and diagnostic accuracy study. *Lancet Infect Dis* 2019; **19**(6): 658-69. 794. Vickerman P, Terris-Prestholt F, Delany S, Kumaranayake L, Rees H, Watts C. Are targeted HIV prevention activities cost-effective in high prevalence settings? Results from a sexually transmitted infection treatment project for sex workers in Johannesburg, South Africa. *Sex Transm Dis* 2006; **33**(10 Suppl): S122-32. 795. Vieira-Baptista P, Grinceviciene S, Bellen G, et al. Genital Tract Infections in an Isolated Community: 100 Women of the Príncipe Island. *Infect Dis Obstet Gynecol* 2017; **2017**: 3058569. 796. Ville Y, Leruez M, Glowaczower E, Robertson JN, Ward ME. The role of Chlamydia trachomatis and Neisseria gonorrhoeae in the aetiology of ectopic pregnancy in Gabon. *Br J Obstet Gynaecol* 1991; **98**(12): 1260-6. 797. Vink G, Moodley J. Gonorrhoea in black women attending a gynaecological outpatient department. *S Afr Med J* 1980; **58**(22): 901-2. 798. Völker F, Cooper P, Bader O, et al. Prevalence of pregnancy-relevant infections in a rural setting of Ghana. *BMC Pregnancy Childbirth* 2017; **17**(1): 172. 799. Vuylsteke B, Bastos R, Barreto J, et al. High prevalence of sexually transmitted diseases in a rural area in Mozambique. *Genitourin Med* 1993b; **69**(6): 427-30. 800. Vuylsteke B, Laga M, Alary M, et al. Clinical algorithms for the screening of women for gonococcal and chlamydial infection: evaluation of pregnant women and prostitutes in Zaire. *Clin Infect Dis* 1993a; **17**(1): 82-8. 801. Vuylsteke B, Semde G, Sika L, et al. High prevalence of HIV and sexually transmitted infections among male sex workers in Abidjan, Cote d'Ivoire: need for services tailored to their needs. *Sex Transm Infect* 2012a; **88**(4): 288-93. 802. Vuylsteke B, Semdé G, Sika L, et al. HIV and STI prevalence among female sex workers in Côte d'Ivoire: why targeted prevention programs should be continued and strengthened. *PLoS ONE* 2012b; **7**(3): e32627. 803. Vuylsteke BL, Ettiègne-Traore V, Anoma CK, et al. Assessment of the validity of and adherence to sexually transmitted infection algorithms at a female sex worker clinic in Abidjan, Côte d'Ivoire. *Sex Transm Dis* 2003; **30**(4): 284-91. 804. Wade AS, Kane CT, Diallo PA, et al. HIV infection and sexually transmitted infections among men who have sex with men in Senegal. *AIDS* 2005; **19**(18): 2133-40. 805. Wahome E, Thiong’o AN, Mwashigadi G, et al. An Empiric Risk Score to Guide PrEP Targeting Among MSM in Coastal Kenya. *AIDS and Behavior* 2018; **22**: 35-44. 806. Wall KM, Nyombayire J, Parker R, et al. Etiologies of genital inflammation and ulceration in symptomatic Rwandan men and women responding to radio promotions of free screening and treatment services. *PLoS ONE* 2021; **16**(4 April). 807. Walraven G, Scherf C, West B, et al. The burden of reproductive-organ disease in rural women in The Gambia, West Africa. *Lancet* 2001; **357**(9263): 1161-7. 808. Watson PA. The use of screening tests for sexually transmitted diseases in a third world community - A feasibility study in Malawi. *European Journal of Sexually Transmitted Diseases* 1985; **2**(2): 63-5. 809. Watson-Jones D, Changalucha J, Gumodoka B, et al. Syphilis in pregnancy in Tanzania. I. Impact of maternal syphilis on outcome of pregnancy. *The Journal of infectious diseases* 2002; **186**(7): 940-7. 810. Watson-Jones D, Mugeye K, Mayaud P, et al. High prevalence of trichomoniasis in rural men in Mwanza, Tanzania: results from a population based study. *Sex Transm Infect* 2000; **76**(5): 355-62. 811. Watson-Jones D, Weiss HA, Changalucha JM, et al. Adverse birth outcomes in United Republic of Tanzania - Impact and prevention of maternal risk factors. *Bulletin of the World Health Organization* 2007; **85**(1): 9-18. 812. Wawer MJ, Gray RH, Sewankambo NK, et al. A randomized, community trial of intensive sexually transmitted disease control for AIDS prevention, Rakai, Uganda. *AIDS* 1998; **12**(10): 1211-25. 813. Weir SS, Feldblum PJ, Zekeng L, Roddy RE. The use of nonoxynol-9 for protection against cervical gonorrhea. *American Journal of Public Health* 1994; **84**(6): 910-4. 814. Weissenberger R, Robertson A, Holland S, Hall W. The incidence of gonorrhoea in urban Rhodesian Black women. *S Afr Med J* 1977; **52**(28): 1119-20. 815. Welgemoed NC, Mahaffey A, Van den Ende J. Prevalence of Neisseria gonorrhoeae infection in patients attending an antenatal clinic. *S Afr Med J* 1986; **69**(1): 32-4. 816. Wessel HF, Herrmann B, Dupret A, Moniz F, Brito C, Bergström S. Genital infections among antenatal care attendees in Cape Verde. *African journal of reproductive health* 1998; **2**(1): 32-40. 817. Wilkinson D, Ndovela N, Harrison A, Lurie M, Connolly C, Sturm AW. Family planning services in developing countries: an opportunity to treat asymptomatic and unrecognised genital tract infections? *Genitourin Med* 1997; **73**(6): 558-60. 818. Williams BG, Taljaard D, Campbell CM, et al. Changing patterns of knowledge, reported behaviour and sexually transmitted infections in a South African gold mining community. *AIDS* 2003; **17**(14): 2099-107. 819. Winston SE, Chirchir AK, Muthoni LN, et al. Prevalence of sexually transmitted infections including HIV in street-connected adolescents in western Kenya. *Sex Transm Infect* 2015; **91**(5): 353-9. 820. Wolday D, Z GM, Mohammed Z, et al. Risk factors associated with failure of syndromic treatment of sexually transmitted diseases among women seeking primary care in Addis Ababa. *Sexually Transmitted Infections* 2004; **80**(5): 392-4. 821. Woo VG, Cohen CR, Bukusi EA, Huchko MJ. Direct questioning is more effective than patient-initiated report for the detection of sexually transmitted infections in a primary care HIV clinic in Western Kenya. *Sex Transm Dis* 2013; **40**(2): 158-61. 822. Wools KK, Menya D, Muli F, Heilman D, Jones R. Perception of risk, sexual behaviour and STD/HIV prevalence in Western Kenya. *East African Medical Journal* 1998; **75**(12): 679-83. 823. Workneh M, Hamill MM, Kakooza F, et al. Antimicrobial Resistance of Neisseria Gonorrhoeae in a Newly Implemented Surveillance Program in Uganda: Surveillance Report. *JMIR Public Health Surveill* 2020; **6**(2): e17009. 824. Wright EA, Aisien AO. Pelvic inflammatory disease and the intrauterine contraceptive device. *Int J Gynaecol Obstet* 1989; **28**(2): 133-6. 825. Wu L, Ssebuliba T, Muwonge TR, et al. Alignment of PrEP Use With Potential HIV Exposure in Young Women and Men in Uganda. *Journal of Acquired Immune Deficiency Syndromes (1999)* 2025; **98**(4): 326 EP - 33. 826. Wyffels A, Haegeman F, Meheus A. [Prevalence of penicillinase-producing Neisseria gonorrhoeae and treatment of gonorrhea in Niger]. *Ann Soc Belg Med Trop* 1985; **65**(4): 363-7. 827. Wynn A, Ramogola-Masire D, Gaolebale P, et al. Prevalence and treatment outcomes of routine Chlamydia trachomatis, Neisseria gonorrhoeae and Trichomonas vaginalis testing during antenatal care, Gaborone, Botswana. *Sexually transmitted infections* 2018; **94**(3): 230-5. 828. Yala F. [Preliminary surveys on Neisseria gonorrhoeae infections in Brazzaville. Incidence and drug sensitivity]. *Bull Soc Pathol Exot Filiales* 1980; **73**(1): 38-48. 829. Yalew GT, Muthupandian S, Hagos K, et al. Prevalence of bacterial vaginosis and aerobic vaginitis and their associated risk factors among pregnant women from northern Ethiopia: A cross-sectional study. *PLoS ONE* 2022; **17**(2 February). 830. Yéo A, Kouamé-Blavo B, Kouamé CE, et al. Establishment of a Gonococcal Antimicrobial Surveillance Programme, in Accordance With World Health Organization Standards, in Côte d'Ivoire, Western Africa, 2014-2017. *Sex Transm Dis* 2019; **46**(3): 179-84. 831. Yirenya-Tawiah D, Annang TN, Apea-Kubi KA, et al. Chlamydia Trachomatis and Neisseria Gonorrhoeae prevalence among women of reproductive age living in urogenital schistosomiasis endemic area in Ghana. *BMC Res Notes* 2014; **7**: 349. 832. Yuh T, Micheni M, Selke S, et al. Sexually Transmitted Infections Among Kenyan Adolescent Girls and Young Women With Limited Sexual Experience. *Front Public Health* 2020; **8**: 303. 833. Yvert F, Riou JY, Frost E, Ivanoff B, Ossari S, Bouatsia P. [Gonococcal infections in Gabon (Haut-Ogooué)]. *Pathol Biol (Paris)* 1984; **32**(2): 80-4. 834. Zachariah R, Harries AD, Nkhoma W, et al. Behavioural characteristics, prevalence of Chlamydia trachomatis and antibiotic susceptibility of Neisseria gonorrhoeae in men with urethral discharge in Thyolo, Malawi. *Trans R Soc Trop Med Hyg* 2002; **96**(3): 232-5. 835. Zenebe MH, Mekonnen Z, Loha E, Padalko E. Prevalence, risk factors and association with delivery outcome of curable sexually transmitted infections among pregnant women in Southern Ethiopia. *PLoS ONE* 2021; **16**(3 March). 836. Zimba TF, Apalata T, Sturm WA, Moodley P. Aetiology of sexually transmitted infections in Maputo, Mozambique. *J Infect Dev Ctries* 2011; **5**(1): 41-7.   **Records identified through bibliographies of eligible studies, reviews, and country-level reports:**   1. AIDS Control Programme-Ministry of Health SoPH-MU. The crane survey report: high risk group surveys conducted in 2008/9, Kampala, Uganda. 2010. 2. Alli J, Okonko I, Odu N, Kolade A. Detection and prevalence of genital pathogens among attendees of STI clinic of a tertiary care hospital in Ibadan, Southwestern Nigeria. World Journal of Medical Sciences 2011; 6(3): 152-61. 3. Apalata T, Longo-Mbenza B, Sturm A, Carr W, Moodley P. Factors associated with symptomatic vulvovaginal candidiasis: a study among women attending a primary healthcare clinic in Kwazulu-Natal, South Africa. Annals of medical and health sciences research 2014; 4(3): 410-6. 4. Auvert B, Ballard R, Campbell C, et al. HIV infection among youth in a South African mining town is associated with herpes simplex virus-2 seropositivity and sexual behaviour. AIDS 2001; 15(7): 885-98. 5. Bailey RC, Moses S, Parker CB, et al. Male circumcision for HIV prevention in young men in Kisumu, Kenya: a randomised controlled trial. The lancet 2007; 369(9562): 643-56. 6. Bosu W, Ampofo W, Armar-Klemesu M, et al. Final report: socio-cultural and bio-behavioural survey in high and low HIV prevalent areas in Ghana. 2011. 7. Braddick MR, Kreiss JK, Embree JE, et al. Impact of maternal HIV infection on obstetrical and early neonatal outcome. AIDS 1990b; 4(10): 1001-6. 8. Cameron DW, D'Costa L, Maitha G, et al. Female to male transmission of human immunodeficiency virus type 1: risk factors for seroconversion in men. The Lancet 1989; 334(8660): 403-7. 9. Celum C, Wald A, Hughes J, et al. Effect of aciclovir on HIV-1 acquisition in herpes simplex virus 2 seropositive women and men who have sex with men: a randomised, double-blind, placebo-controlled trial. The Lancet 2008; 371(9630): 2109-19. 10. Center for Social Research, Save the Children Federation USA, Malawi Ministry of Health and Population, MEASURE Evaluation. Avoiding Unwanted Pregnancy and Sexually Transmitted Infections: A Rural Malawi District Study. Chapel Hill, NC; 2004. 11. Changalucha J, West B, Rwakatare M, Marealle J. Strain diversity and antimicrobial susceptibility of Neisseria gonorrhoeae in Mwanza and Ngara Districts, Tanzania. Tanzania Journal of Health Research 2002; 4(2): 33-7. 12. Cisse D. Second generation monitoring of HIV among sex workers and their male sexual partners in Senegal: Laval University; 2006. 13. Crewe-Brown H, * Krige, FK,* Davel, GH,* Barron, C.,* Jansen van Vuuren, JAM,* Shipham, SO*, Roux J. Genital ulceration in males at Ga-Rankuwa Hospital, Pretoria. South African Medical Journal 1982; 62(23): 861-3. 14. Crewe-Brown HH AA, Ebrahim O, et al. . The aetiology of acute urethritis in a southern African general practice. S Afr J Epidemiol Infect 1991; 6(1): 31–3. 15. De Jongh M, Le Roux M, Adam A, Caliendo A, Hoosen AA. Co-infection with neisseria gonorrhoeae, Chlamydia trachomatis and Trichomonas vaginalis in symptomatic south african men with urethritis: implications for syndromic management. 2009. 16. De Schampheleire I, Van de Velden L, Van Dyck E, Guindo S, Quint W, Fransen L. [Sexually transmitted diseases in the female population of Pikine, Senegal]. Ann Soc Belg Med Trop 1990; 70(3): 227-35. 17. Delany S, Mlaba N, Clayton T, et al. Impact of aciclovir on genital and plasma HIV-1 RNA in HSV-2/HIV-1 co-infected women: a randomized placebo-controlled trial in South Africa. AIDS 2009; 23(4): 461-9. 18. Delany-Moretlwe S, Bello B, Kinross P, et al. HIV prevalence and risk in long-distance truck drivers in South Africa: a national cross-sectional survey. International journal of STD & AIDS 2014; 25(6): 428-38. 19. Ekanem EI, Ekott M, Udo AE, Efiok EE, Inyang-Out A. Prevalence of sexually transmitted diseases in pregnant women in Ikot Ekpene, a rural community in Akwa Ibom State, Nigeria. 2012. 20. Enabulele IO, Kemajo TS. Prevalence of Sexually Transmitted Infections in Patients Attending Sexually Transmitted Disease (STD) Clinics in Benin City, Nigeria. Journal of Medical Laboratory Science 2006; 15. 21. Enhancing Nigeria’s Response to HIV/AIDS Programme. Prevalence of sexually transmitted infections among men who have sex with men and injecting drug users and validation of audio computer-assisted self interview (ACASI) technique in Abuja, Lagos, and Ibadan, Nigeria: Report fact sheet. Abuja, 2011. 22. Erwin JOT. Reproductive tract infections among women in Ado-Ekiti, Nigeria: Symptoms recognition, perceived causes and treatment choices. Health Transition Review 1993: 135-49. 23. FAST MV, D'COSTA LJ, NSANZE H, et al. The clinical diagnosis of genital ulcer disease in men in the tropics. Sexually transmitted diseases 1984: 72-6. 24. Geyid A, Tesfaye HS, Abraha A, Lemeneh Y, Desta S, Feleke W. Isolates of STDs causative agents from sex workers Addis Ababa (a preliminary report). Ethiopian Journal of Health Development 1990; 4(2). 25. Ghana AIDS Commission. Integrated Biological and Behavioral Surveillance Survey among Female Sex Workers in Ghana: 2010-2011. 2012. 26. Goldenberg RL, Mwatha A, Read JS, et al. The HPTN 024 Study: the efficacy of antibiotics to prevent chorioamnionitis and preterm birth. Am J Obstet Gynecol 2006; 194(3): 650-61. 27. Gosmann C, Handley SA, Farcasanu M, et al. Lactobacillus-deficient cervicovaginal bacterial communities are associated with increased HIV acquisition in young South African women. Immunity 2017; 46(1): 29-37. 28. Greenblatt RM, Lukehart SA, Plummer FA, et al. Genital ulceration as a risk factor for human immunodeficiency virus infection. AIDS 1988; 2(1): 47-50. 29. Gresenguet G, Kreiss JK, Chapko MK, Hillier SL, Weiss NS. HIV infection and vaginal douching in Central Africa. AIDS 1997; 11(1). 30. Hanson S, Sunkutu RM, Kamanga J, Hojer B, Sandstrom E. STD care in Zambia: an evaluation of the guidelines for case management through a syndromic approach. International Journal of STD & AIDS 1996; 7(5): 324-32. 31. Heffron R, Donnell D, Rees H, et al. Use of hormonal contraceptives and risk of HIV-1 transmission: a prospective cohort study. The Lancet Infectious Diseases 2012; 12(1): 19-26. 32. Heffron R, Ngure K, Velloza J, et al. Implementation of a comprehensive safer conception intervention for HIV‐serodiscordant couples in Kenya: uptake, use and effectiveness. Journal of the International AIDS Society 2019; 22(4): e25261. 33. Hema M N TI, Meda N, Drabo D, Somé J, Konaté I, Sanou A, Diallo R, Karambiri D, Ouédraogo M, Somé F, Traoré S, Diabaté H, Somé R, Mayaud P, Van De Perre P, Nagot N,. Incidence of pregnancies as an indicator of high-risk HIV infection behaviors in a context of low HIV seroconversion rate: case of the HIV pre-vaccination cohort in Ouagadougou. Burkina Faso; 2011. 34. Hoosen AA MJ, Maitin P. . Bacterial vaginosis in symptomatic women attending a gynaecology outpatient clinic. S Afr J Epidemiol Infect 1997; 12: 119–21. 35. Hoosen AA vdEJ, Kharsany AB. The aetiology of acute urethritis in black males in Durban, South Africa and penicillin susceptibility of Neisseria gonorrhoeae isolates. Southern African Journal of Epidemiology and Infection 1987; 2: 4-6. 36. Ibrahim S, Bukar M, Mohammad Y, Audu B, Ibrahim H. Prevalence of vaginal candidiasis among pregnant women with abnormal vaginal discharge in Maiduguri. Nigerian Journal of Medicine 2013; 22(2): 138-42. 37. International Organization for Migration. Integrated biological and behavioural surveillance survey among migrant female sex workers in Nairobi, Kenya. 2010. 38. Jatau E, Galadima M, Odama L, Kwaga J. Prevalence and antimicrobial susceptibility of Neisseria gonorrhoeae isolated from patients in various locations of Kaduna state, Nigeria. Nigerian journal of surgical research 2003; 5(1): 50-6. 39. Kapiga S, Sam N, Mlay J, et al. The epidemiology of HIV-1 infection in northern Tanzania: results from a community-based study. AIDS care 2006; 18(4): 379-87. 40. Kapiga SH, Sam NE, Shao JF, et al. Herpes simplex virus type 2 infection among bar and hotel workers in northern Tanzania: prevalence and risk factors. Sexually transmitted diseases 2003; 30(3): 187-92. 41. Kapiga SH, Sam NE, Shao JF, et al. HIV-1 epidemic among female bar and hotel workers in northern Tanzania: risk factors and opportunities for prevention. JAIDS Journal of Acquired Immune Deficiency Syndromes 2002; 29(4): 409-17. 42. Kaydos-Daniels SC, Miller WC, Hoffman I, et al. The Use of Specimens from Various Genitourinary Sites in Men, to Detect Trichomonas vaginalis Infection. The Journal of Infectious Diseases 2004; 189(10): 1926-31. 43. Kelly HA, Sawadogo B, Chikandiwa A, et al. Epidemiology of high-risk human papillomavirus and cervical lesions in African women living with HIV/AIDS: effect of anti-retroviral therapy. AIDS 2017; 31(2): 273-85. 44. Kharsany A, Mashego M, Mdlotshwa M, Frohlich J, Karim QA. Direct Questioning of Genital Symptoms Increasing Opportunities for Identifying and Treating Sexually Transmitted Infections in Primary Health-care Settings. African journal of reproductive health 2006; 10(2): 105-14. 45. Kibukamusoke J. Venereal disease in East Africa. Transactions of the Royal Society of Tropical Medicine and Hygiene 1965; 59(6): 642-8. 46. Kjetland EF, Gwanzura L, Ndhlovu PD, et al. Herpes simplex virus type 2 prevalence of epidemic proportions in rural Zimbabwean women: association with other sexually transmitted infections. Archives of gynecology and obstetrics 2005; 272: 67-73. 47. Kjetland EF, Poggensee G, Helling-Giese G, et al. Female genital schistosomiasis due to Schistosoma haematobium Clinical and parasitological findings in women in rural Malawi. Acta Tropica 1996; 62(4): 239-55. 48. Kularatne R, Radebe F, Kufa-Chakezha T, Mbulawa Z, Lewis D. Sentinel surveillance of sexually transmitted infection syndrome aetiologies and HPV genotypes among patients attending primary health care facilities in South Africa, April 2014–September 2015: National Institute of Communicable Diseases, 2017. 49. Kumwenda N, Hoffman I, Chirenje M, et al. HIV incidence among women of reproductive age in Malawi and Zimbabwe. Sexually transmitted diseases 2006; 33(11): 646-51. 50. La Ruche G, Messou N, Ali-Napo L, et al. Vaginal Douching: Association With Lower Genital Tract Infections in African Pregnant Women. Sexually Transmitted Diseases 1999; 26(4). 51. Lassey A, Adanu R, Newman M, Opintah J. Potential pathogens in the lower genital tract at manual vacuum aspiration for incomplete abortion in Korle Bu Teaching Hospital, Ghana. East African medical journal 2004; 81(8): 398-401. 52. Lindan CP, Allen S, Serufilira A, et al. Predictors of mortality among HIV-infected women in Kigali, Rwanda. Annals of internal medicine 1992; 116(4): 320-8. 53. Louis MESK, Munkolenkole, Brown C, Nelson AM, et al. Risk for Perinatal HIV-1 Transmission According to Maternal Immunologic, Virologic, and Placental Factors. JAMA 1993b; 269(22): 2853-9. 54. Low AJ, Clayton T, Konate I, et al. Genital warts and infection with human immunodeficiency virus in high-risk women in Burkina Faso: a longitudinal study. BMC infectious diseases 2011; 11: 1-9. 55. Mabey D, Tedder R, Hughes A, et al. Human retroviral infections in The Gambia: prevalence and clinical features. Br Med J (Clin Res Ed) 1988; 296(6615): 83-6. 56. Martin HL, Jr., Jackson DJ, Mandaliya K, et al. Preparation for AIDS vaccine evaluation in Mombasa, Kenya: establishment of seronegative cohorts of commercial sex workers and trucking company employees. AIDS Res Hum Retroviruses 1994; 10 Suppl 2: S235-7. 57. Martin PMV, Gresenguet G, Massanga M, Georges A, Testa J. Association between HIV1 infection and sexually transmitted disease among men in central Africa. Research in Virology 1992; 143: 205-9. 58. Mason PR, Gregson S, Gwanzura L, Cappuccinelli P, Rapelli P, Fiori PL. Enzyme immunoassay for urogenital trichomoniasis as a marker of unsafe sexual behaviour. Epidemiology & Infection 2001; 126(1): 103-9. 59. Mbofana FS, Brito FJ, Saifodine A, Cliff JL. Syndromic management of sexually transmitted diseases at primary care level, Mozambique. Sexually Transmitted Infections 2002; 78(1): e2. 60. Mbopi-Kéou F-X, Grésenguet G, Mayaud P, et al. Interactions between Herpes Simplex Virus Type 2 and Human Immunodeficiency Virus Type 1 Infection in African Women: Opportunities for Intervention. The Journal of Infectious Diseases 2000; 182(4): 1090-6. 61. McClelland RS, Richardson BA, Hassan WM, et al. Improvement of Vaginal Health for Kenyan Women at Risk for Acquisition of Human Immunodeficiency Virus Type 1: Results of a Randomized Trial. The Journal of Infectious Diseases 2008; 197(10): 1361-8. 62. MEASURE Evaluation. Evaluation of the USAID support for HIV prevention in Mali from 2000 to 2010. 2014. 63. Meda N, Ndoye I, M‚Boup S, et al. Low and stable HIV infection rates in Senegal: natural course of the epidemic or evidence for success of prevention? AIDS 1999; 13(11). 64. MEHEUS A, VAN DYCK E, URSI JP, BALLARD RC, PIOT P. Etiology of genital ulcerations in Swaziland. Sexually Transmitted Diseases 1983: 33-5. 65. Miller W. Final report: programmatic mapping and prevalence of HIV and other STIs among key populations in Angola: PLACE study 2017. University of North Carolina; 2018. 66. Ministry of Health and Public Hygiene. Survey of HIV and associated risk factors among men who have sex with men in San-Pedro, Côte d'Ivoire. 2018. 67. Ministry of Health and Wellness-Republic of Botswana. Biological and behavioral surveillance survey of HIV/STIs among select key populations. 2017. 68. Ministry of Health of Mali. Integrated survey on STI prevalence and behaviors conducted in Mali from April to June 2009: final report. 2010. 69. Ministry of Health-Republic of Benin. Second generation surveillance survey of STI/HIV/AIDS in Benin. 2012. 70. Ministry of Health-Republic of Benin. Second generation surveillance survey on STIs, HIV and AIDS among adolescents-young people, truck drivers, persons deprived of liberty, sex workers and clients, bar and restaurant waitresses in 2021 in Benin. 2022. 71. Ministry of Health-Republic of Botswana. 2012 Mapping, size estimation & Behavioral and Biological Surveillance Survey (BBSS) of HIV/STI among selectd high-risk sub-populations in Botswana. 2013. 72. Moges B, Yismaw G, Kassu A, et al. Sexually transmitted infections based on the syndromic approach in Gondar town, northwest Ethiopia: a retrospective study. BMC Public Health 2013; 13: 1-5. 73. Moodley S. Mixed sexually transmitted infections in adult male patients with urethral discharge and female patients with pelvic inflammatory disease. Afr J Microbiol Res 2013; 7(19): 1946-50. 74. Morison L, Weiss HA, Buve A, et al. Commercial sex and the spread of HIV in four cities in sub-Saharan Africa. AIDS 2001; 15: S61-S9. 75. Moss GB, Clemetson D, D'Costa L, et al. Association of cervical ectopy with heterosexual transmission of human immunodeficiency virus: results of a study of couples in Nairobi, Kenya. Journal of Infectious Diseases 1991; 164(3): 588-91. 76. Msuya SE, Mbizuo E, Stray-Pedersen B, Sundby J, Sam NE, Hassain A. Reproductive tract infections among women attending primary health care facilities in Moshi, Tanzania. East African medical journal 2002; 79(1): 16-21. 77. Mujugira A, Baeten JM, Donnell D, et al. Characteristics of HIV-1 serodiscordant couples enrolled in a clinical trial of antiretroviral pre-exposure prophylaxis for HIV-1 prevention. PLoS ONE 2011; 6(10): e25828. 78. Mukamuyango J, Ingabire R, Karita E, et al. Prevalence of HIV, sexual transmitted infections and high risk sexual behaviors among female sex workers in Kigali, Rwanda. AIDS Research and Human Retroviruses 2014; 30(S1): A279-A. 79. N'Guematcha R. Antibiotic sensitivity of Neisseria gonorrhoeae isolates from Yaounde (Cameroon). Annales de Microbiologie 1980; 131 A(1): 90. 80. National AIDS Control Programme. HIV behavioral and biological surveillance survey among female sex workers in Dar es Salaam, 2010. 2011. 81. National Council for the Fight Against AIDS. National integrated surveillance survey of STIs and HIV/AIDS. 2015. 82. National Council for the Fight Against AIDS, Division AIDS/STI. Report of the integrarted survey among Men who have Sex with Men (MSM) in Senegal (2014). 2014. 83. National MARPs Programme N-MoH. 2010-2011 Integrated biological and behavioural surveillance survey among key populations in Nairobi and Kisumu, Kenya. Kenya; 2014. 84. Nduati R, John G, Mbori-Ngacha D, et al. Effect of Breastfeeding and Formula Feeding on Transmission of HIV-1A Randomized Clinical Trial. JAMA 2000; 283(9): 1167-74. 85. Nwokedi EE, Azeez-Akande O, Dikko AU. Pattern Of Sexually Transmitted Infections In A Reference Clinic Of Aminu Kano Teaching Hospital, Kano. Highland Medical Research Journal 2004; 2(2): 55-60. 86. O'Farrell N, Morison L, Moodley P, et al. Association Between HIV and Subpreputial Penile Wetness in Uncircumcised Men in South Africa. JAIDS Journal of Acquired Immune Deficiency Syndromes 2006; 43(1). 87. Okonko I, Okerentugba P, Adejuwon A, Onoh C. Prevalence of sexually transmitted infections (STIs) among attendees of lead city university medical centre in Ibadan, Southwestern Nigeria. Nigeria Arch Appl Sci Res 2012b; 4(2): 980-7. 88. Olatunbosun DA. Infertility and Subfertility in Rural Western Nigeria. 1978. 89. Ongom VL. Prevalence and incidence of venereal diseases in military communities in Uganda. East African Medical Journal 1970; 47(9): 479–83. 90. Opoku BK, Sarkodie Y. Prevalence of genital chlamydia and gonococcal infections in at risk women in the Kumasi metropolis, Ghana. Ghana Medical Journal 2010; 44(1). 91. Oyelese AO, Onipede AO, Aboderin AO, Adedosu AN, Onayemi O. Sexually transmitted infections in Obafemi Awolowo University Teaching Hospital, Ile-Ife, Nigeria: A decade of clinic experience. African journal of clinical and experimental microbiology 2004; 6(1). 92. Plourde PJ, Plummer FA, Pepin J, et al. Human immunodeficiency virus type 1 infection in women attending a sexually transmitted diseases clinic in Kenya. Journal of Infectious Diseases 1992; 166(1): 86-92. 93. PLUMMER FA, D'COSTA LJ, NSANZE H, et al. Clinical and microbiologic studies of genital ulcers in Kenyan women. Sexually transmitted diseases 1985; 12(4): 193-7. 94. Poggensee G, Kiwelu I, Weger V, et al. Female genital schistosomiasis of the lower genital tract: prevalence and disease-associated morbidity in northern Tanzania. The Journal of infectious diseases 2000; 181(3): 1210-3. 95. Ramjee G, Kapiga S, Weiss S, et al. The value of site preparedness studies for future implementation of phase 2/IIb/III HIV prevention trials: experience from the HPTN 055 study. JAIDS Journal of Acquired Immune Deficiency Syndromes 2008; 47(1): 93-100. 96. Ramjee G, Williams B, Gouws E, Van Dyck E, De Deken B, Karim SA. The impact of incident and prevalent herpes simplex virus-2 infection on the incidence of HIV-1 infection among commercial sex workers in South Africa. JAIDS Journal of Acquired Immune Deficiency Syndromes 2005; 39(3): 333-9. 97. Rebbapragada A, Wachihi C, Pettengell C, et al. Negative mucosal synergy between Herpes simplex type 2 and HIV in the female genital tract. AIDS 2007; 21(5): 589-98. 98. Roche SD, Omollo V, Mogere P, et al. A modified pharmacy provider-led delivery model of oral HIV pre- and post-exposure prophylaxis in Kenya: a pilot study extension. J Int AIDS Soc 2025; 28 Suppl 1(Suppl 1): e26467. 99. Ross DA, Changalucha J, Obasi AI, et al. Biological and behavioural impact of an adolescent sexual health intervention in Tanzania: a community-randomized trial. AIDS 2007; 21(14): 1943-55. 100. Rours G, Verkooyen R, Hop W, et al. Sexually transmitted infections in pregnant urban South African women: socio-economic characteristics and risk factors. Southern African Journal of Epidemiology and Infection 2006; 21(1): 14-9. 101. Seck K, Samb N, Tempesta S, et al. Prevalence and risk factors of cervicovaginal HIV shedding among HIV-1 and HIV-2 infected women in Dakar, Senegal. Sexually transmitted infections 2001; 77(3): 190-3. 102. Sectoral Unit for the Fight against HIV/AIDS Tuberculosis and Viral Hepatitis- Ministry of Health and Social Affairs. Bio-behavioural study of STIs, HIV and tuberculosis in female sex workers and truck drivers in Mali 2017-2019. 2019. 103. Shaetonhodi NG, de Voux A, Babalola CM, et al. Prevalence, symptomology, and correlates of curable sexually transmitted infections among pregnant women in Eastern Cape, South Africa. Int J STD AIDS 2025: 9564624251347484. 104. Simonse JN, Plummer FA, Ngugi EN, et al. HIV infection among lower socioeconomic strata prostitutes in Nairobi. AIDS 1990; 4(2): 139-44. 105. Sinei S, M'riara G, Schulz K, et al. The prevalence of Neisseria gonorrhoea and Chlamydia trachomatis in intra-uterine contraceptive acceptors in Kenya. Journal of obstetrics & gynaecology of Eastern and Central Africa 1988; 7(2): 71-3. 106. Sobngwi-Tambekou J, Taljaard D, Nieuwoudt M, Lissouba P, Puren A, Auvert B. Male circumcision and Neisseria gonorrhoeae, Chlamydia trachomatis and Trichomonas vaginalis: observations after a randomised controlled trial for HIV prevention. Sex Transm Infect 2009; 85(2): 116-20. 107. Sylverken AA, Owusu-Dabo E, Yar DD, et al. Bacterial etiology of sexually transmitted infections at a STI clinic in Ghana; use of multiplex real time PCR. Ghana medical journal 2016; 50(3): 142-8. 108. TDRC NAC, FHI/IMPACT,. Round 1 Behavioral and Biologic Surveillance Survey Zambia. 2000. 109. Thornton RL. The demand for, and impact of, learning HIV status. American Economic Review 2008; 98(5): 1829-63. 110. Urassa WK, Kapiga SH, Msamanga GI, Antelman G, Coley J, Fawzi WW. Risk factors for syphilis among HIV-1 infected pregnant women in Dar es Salaam, Tanzania. African journal of reproductive health 2001: 54-62. 111. Van Damme L, Corneli A, Ahmed K, et al. Preexposure Prophylaxis for HIV Infection among African Women. New England Journal of Medicine 2012; 367(5): 411-22. 112. Van de Perre P, De Clercq A, Cogniaux-Leclerc J, Nzaramba D, Butzler J, Sprecher-Goldberger S. Detection of HIV p17 antigen in lymphocytes but not epithelial cells from cervicovaginal secretions of women seropositive for HIV: implications for heterosexual transmission of the virus. Sexually Transmitted Infections 1988; 64(1): 30-3. 113. West Africa Program to Combat AIDS and STI. Report: population size estimates and Integrated Biological and Behavioral Survey among Female Sex Workers and their intimate partners in Ghana. 2020. 114. Zahn R, Domiguez K, Sanchez T, et al. High prevalence of sexually transmitted infections in a cohort of men who have sex with men and transgender women from Port Elizabeth and Cape Town, South Africa. 2016. |
| --- |

Abbreviations: NG, *Neisseria gonorrhoeae*.

# Fig A. Geographic distribution of prevalence data. Country-level distribution of NG prevalence measures across sub-Saharan Africa, showing the spatial distribution and relative contribution of studies by country.


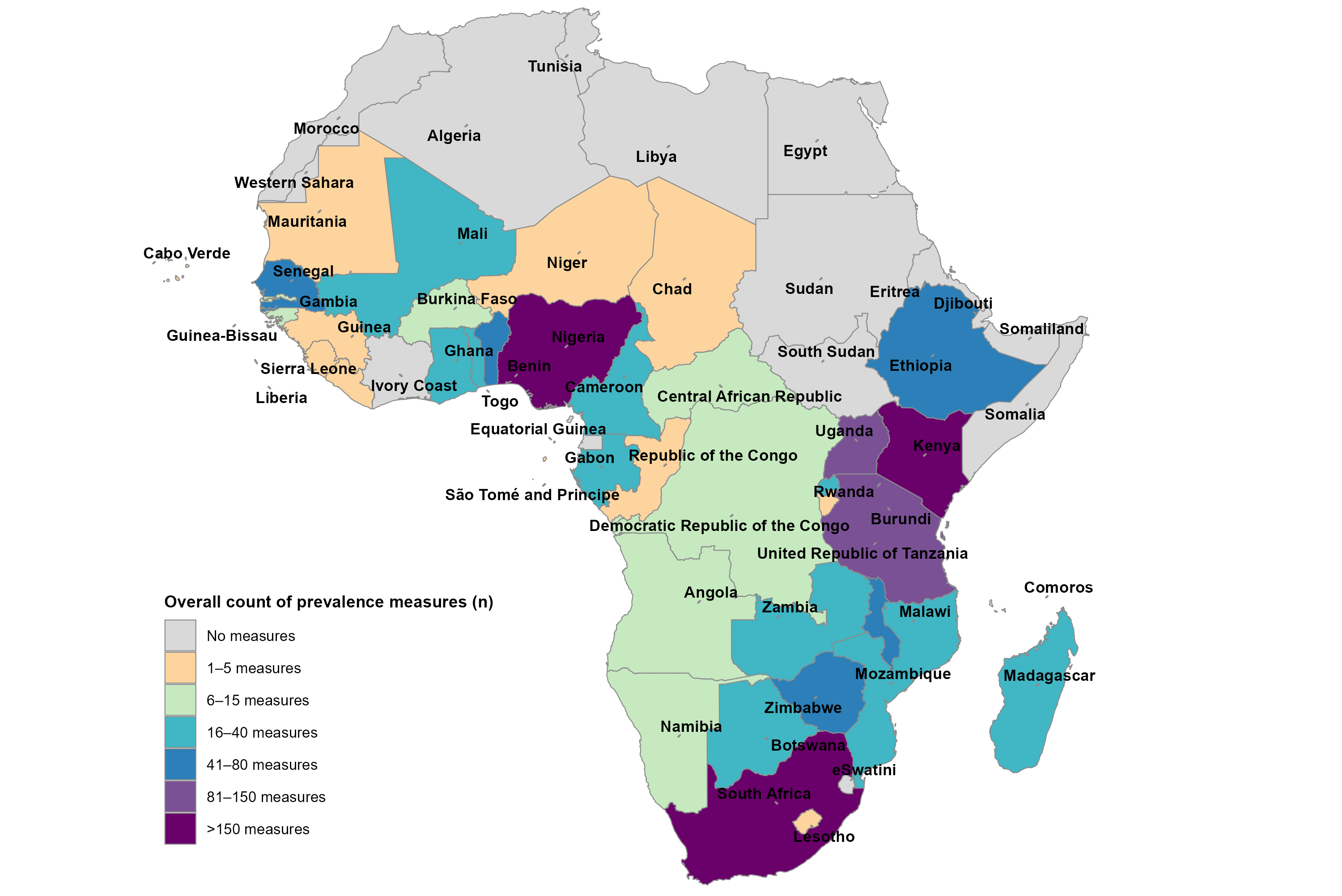


Abbreviations: NG, *Neisseria gonorrhoeae*.

The map was generated in R using Natural Earth public-domain country boundary shapefiles (<https://www.naturalearthdata.com/>).

# **Fig B. Temporal trends in NG prevalence.** NG prevalence estimates across all population groups over time by A) year of data collection and B) publication year. Points represent individual prevalence estimates, and lines indicate fitted linear trends.

| A By year of data collection  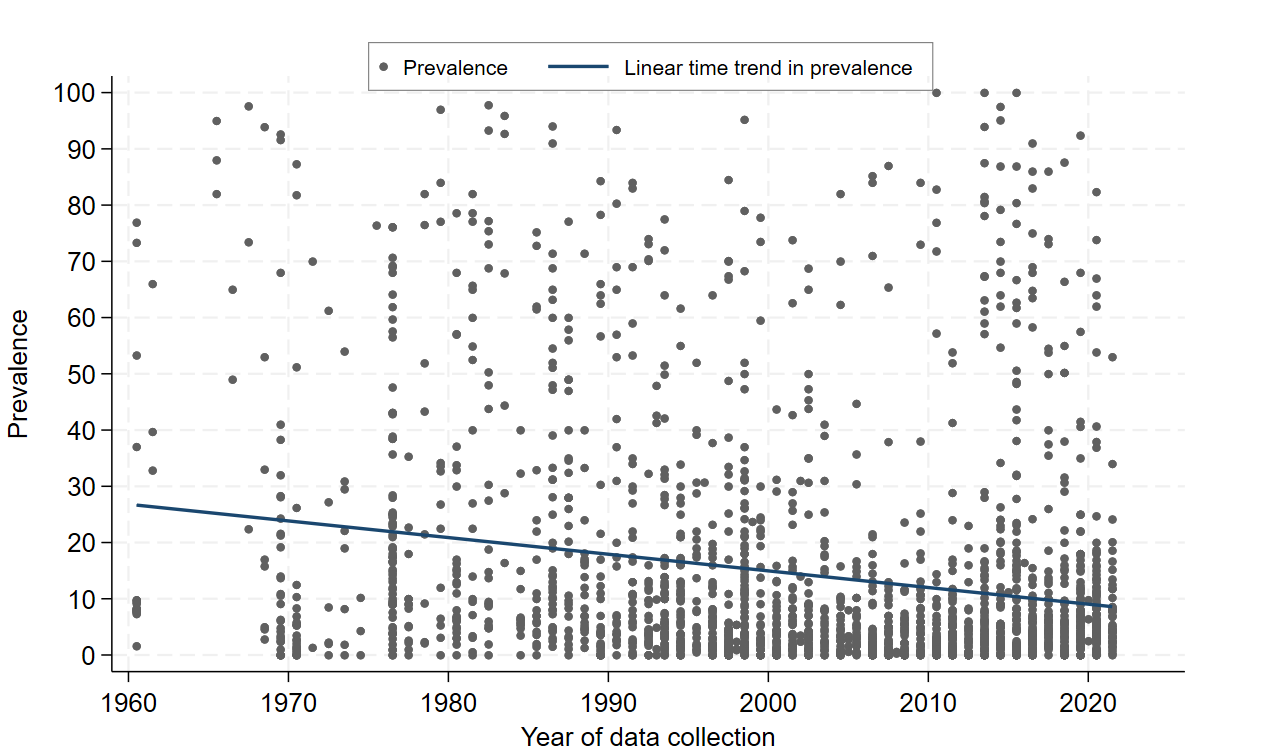 | B By publication year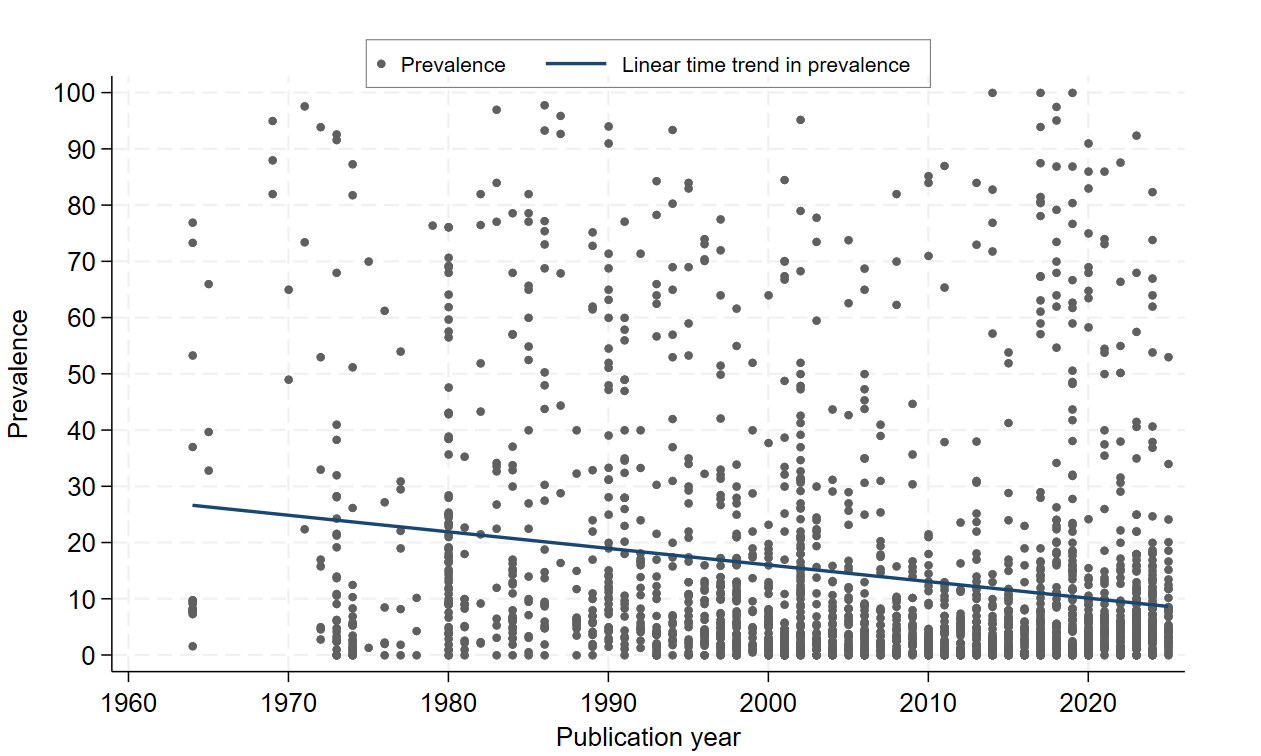 |
| --- | --- |

Abbreviations: NG, *Neisseria gonorrhoeae*.

# Fig C. Temporal trends in NG prevalence across subregions. NG prevalence estimates across all population groups over time by year of data collection for A) Central Africa, B) Eastern Africa, C) Southern Africa, and D) Western Africa. Points represent individual prevalence estimates, and lines indicate fitted linear trends.

| A Central Africa  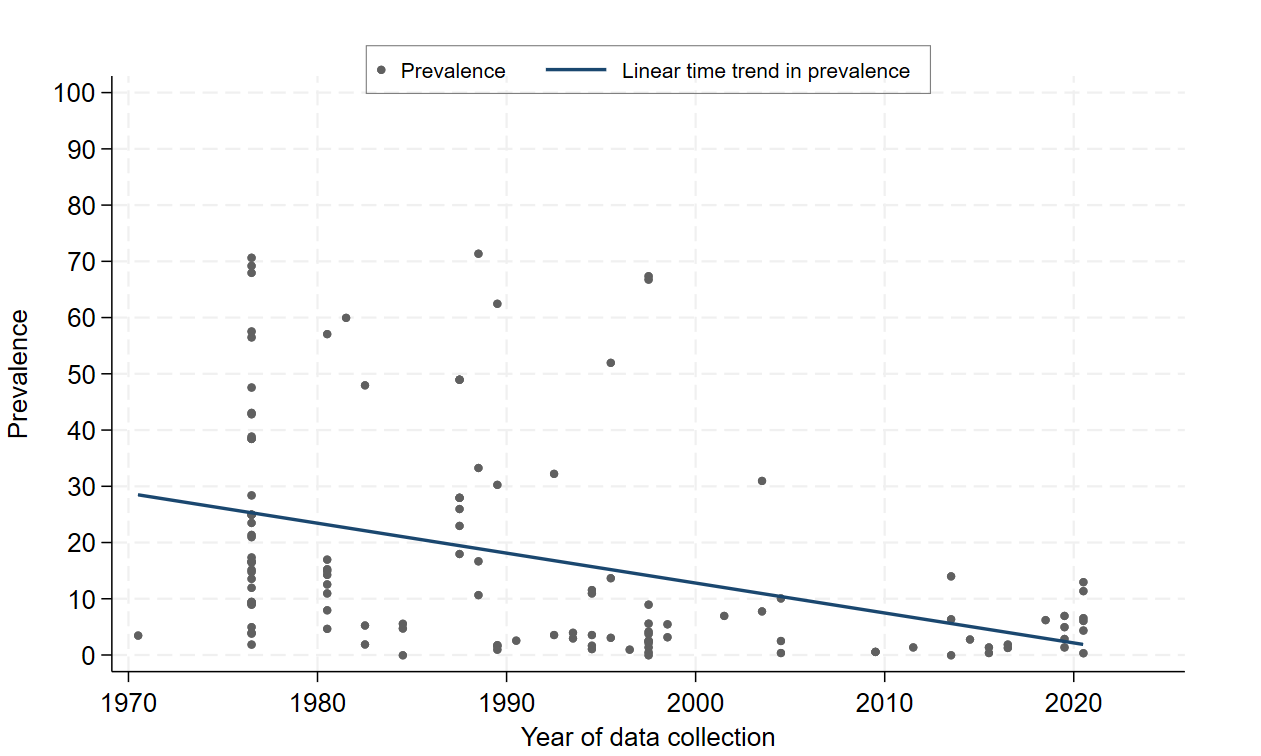 | B Eastern Africa  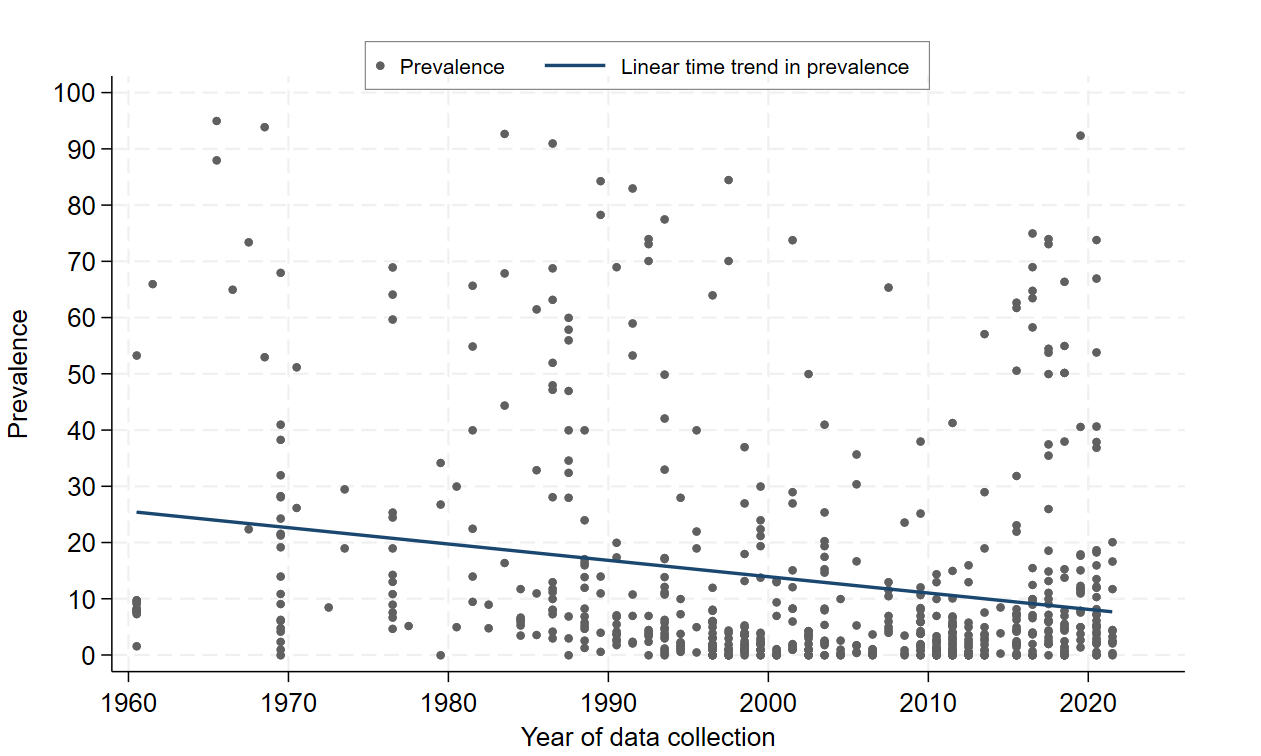 |
| --- | --- |
| C Southern Africa  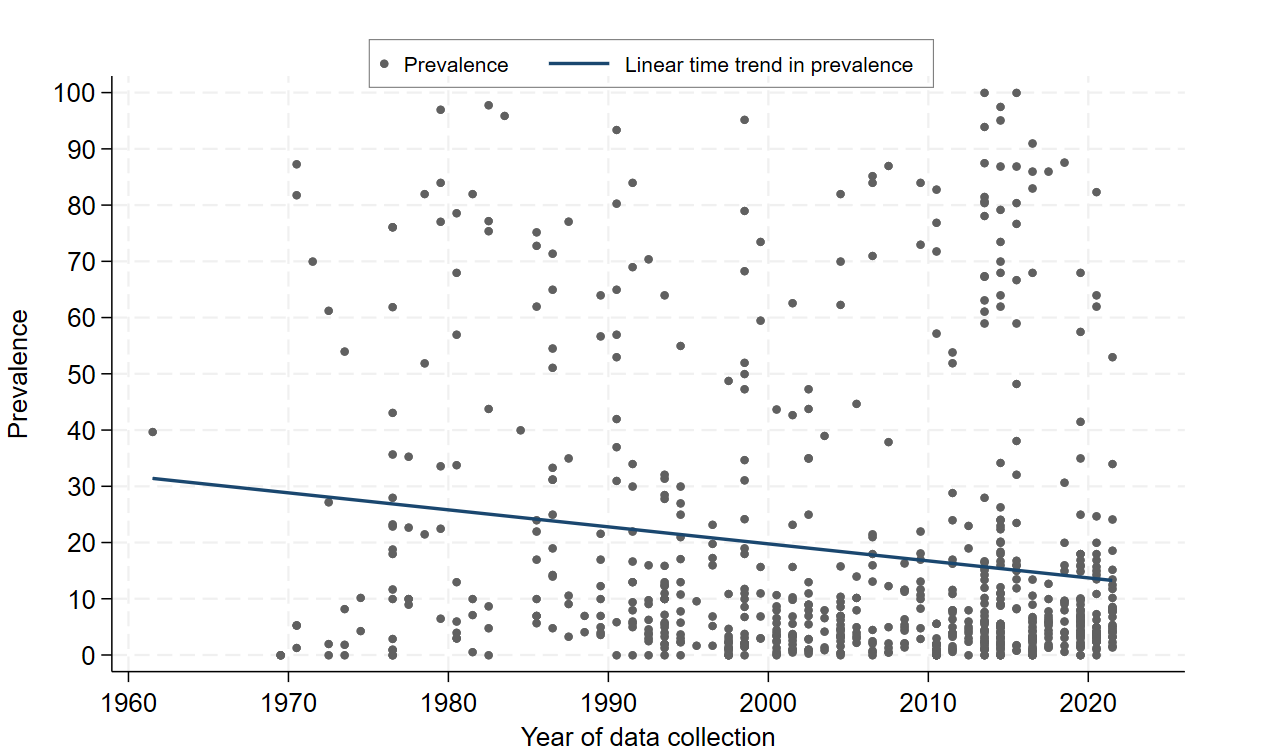 | D Western Africa  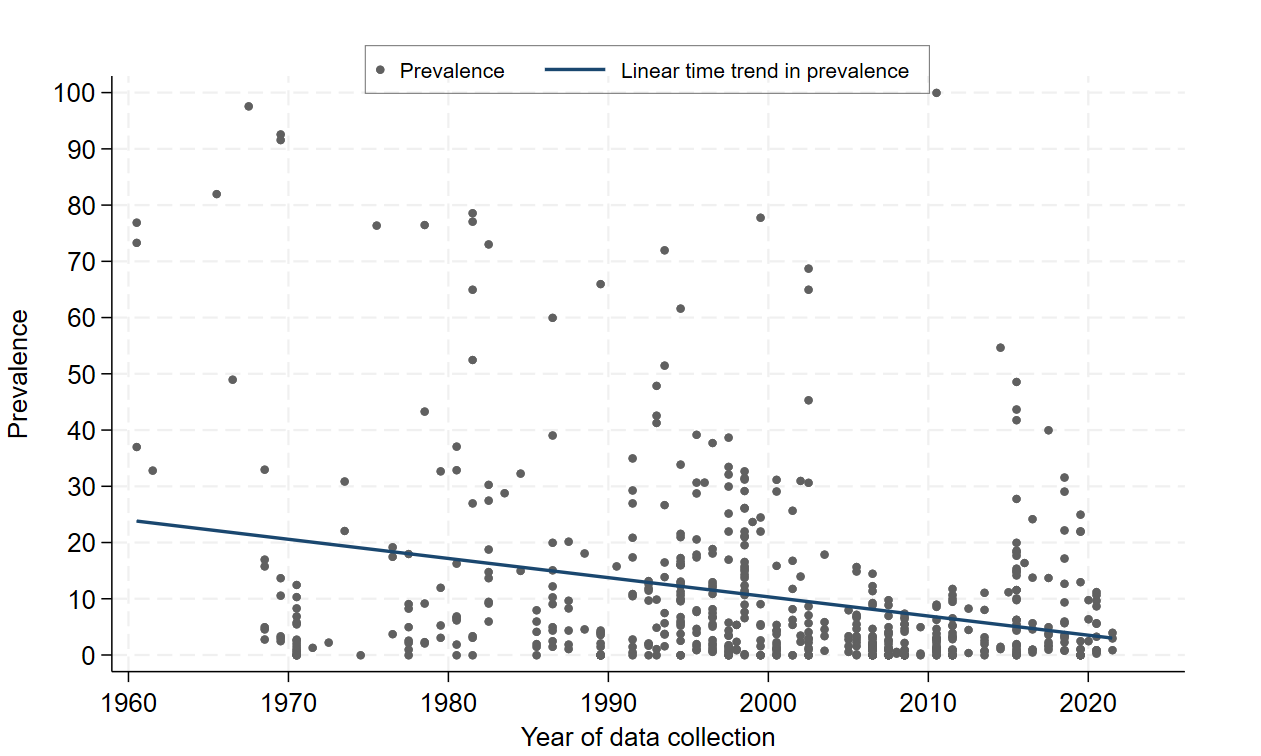 |

Abbreviations: NG, *Neisseria gonorrhoeae*.

# Table E. Summary of study quality assessment. Summary of precision and risk of bias assessments for studies reporting NG prevalence in sub-Saharan Africa.

| **Quality assessment** | **NG prevalence measures** | |
| --- | --- | --- |
|  | **Number of studies** | **Percentage** |
| **Precision of prevalence measures^*^** | | |
| Low precision | 582 | 36.3% |
| High precision | 1,022 | 63.7% |
| **Risk of bias quality domain^†^** | | |
| **Sampling method** | | |
| Low risk of bias | 278 | 17.3% |
| High risk of bias | 1,326 | 82.7% |
| Unclear risk of bias | 0 | 0.0% |
| **Response rate** | | |
| Low risk of bias | 232 | 14.5% |
| High risk of bias | 60 | 3.7% |
| Unclear risk of bias | 1,312 | 81.8% |
| **Summary of the risk of bias assessment** | | |
| **Low risk of bias** |  |  |
| In at least one quality domain | 435 | 27.1% |
| In both quality domains | 75 | 4.7% |
| **High risk of bias** |  |  |
| In at least one quality domain | 1,348 | 84.0% |
| In both quality domains | 38 | 2.4% |
| **Unclear risk of bias** |  |  |
| In at least one quality domain | 1,312 | 81.8% |
| In both quality domains | 0 | 0.0% |
| **Prevalence studies where risk of bias assessment was possible** | 1,604 | 100.0% |

Abbreviations: NG, *Neisseria gonorrhoeae*.

^*^Precision was assessed based on the study’s overall sample size, rather than the subsample size of individual strata, as reported in the publication/record.

^†^Risk of bias was assessed based on the study’s overall sample size, rather than the subsample size of individual strata, as reported in the publication/record.

# **Table F.** Publication bias assessment. Assessment of publication bias in studies reporting NG prevalence in sub-Saharan Africa using Doi plots and the LFK index [4].

| **Population type** | **Number of measures** | **LFK index** | **Doi plot inspection** | **Interpretation** |
| --- | --- | --- | --- | --- |
| **Current urogenital infection** | | | | |
| General populations | 706 | 0.97 | Symmetrical Doi plot | No publication bias |
| Intermediate-risk populations | 99 | 0.46 | Symmetrical Doi plot | No publication bias |
| Female sex workers | 200 | 1.30 | Asymmetrical Doi plot | Indicative of potential publication bias |
| Men who have sex with men^*^ | 46 | -0.38 | Symmetrical Doi plot | No publication bias |
| Symptomatic women | 237 | 1.45 | Asymmetrical Doi plot | Indicative of potential publication bias |
| Symptomatic men | 248 | -2.06 | Asymmetrical Doi plot | Indicative of potential publication bias |
| Symptomatic women and men | 48 | 0.38 | Symmetrical Doi plot | No publication bias |
| Infertility clinic attendees | 20 | 1.78 | Asymmetrical Doi plot | Indicative of potential publication bias |
| Women with adverse pregnancy or birth outcomes^†^ | 16 | 0.60 | Symmetrical Doi plot | No publication bias |
| STI clinic attendees | 65 | 0.85 | Symmetrical Doi plot | No publication bias |
| Individuals living with HIV and individuals in HIV-discordant couples | 81 | 3.05 | Asymmetrical Doi plot | Indicative of potential publication bias |
| Sexual contacts of persons infected with NG/CT | 3 | 0.73 | Symmetrical Doi plot | No publication bias |
| Patients with confirmed or suspected STIs and related infections | 25 | 1.62 | Asymmetrical Doi plot | Indicative of potential publication bias |
| Other populations^‡^ | 75 | 2.85 | Asymmetrical Doi plot | Indicative of potential publication bias |
| **Current anorectal infection** | | | | |
| General populations | 4 | -1.88 | Asymmetrical Doi plot | Indicative of potential publication bias |
| Female sex workers | 3 | -2.44 | Asymmetrical Doi plot | Indicative of potential publication bias |
| Men who have sex with men^*^ | 36 | -0.26 | Symmetrical Doi plot | No publication bias |
| Symptomatic men | 3 | -0.20 | Symmetrical Doi plot | No publication bias |
| **Current oropharyngeal infection** | | | | |
| Men who have sex with men^*^ | 12 | 1.95 | Asymmetrical Doi plot | Indicative of potential publication bias |
| STI clinic attendees | 3 | 5.72 | Asymmetrical Doi plot | Indicative of potential publication bias |
| **Serology (ever infection)** |  |  |  |  |
| General populations | 4 | 5.14 | Asymmetrical Doi plot | Indicative of potential publication bias |
| Men who have sex with men^*^ | 9 | 1.06 | Asymmetrical Doi plot | Indicative of potential publication bias |
| Symptomatic men | 3 | 1.42 | Asymmetrical Doi plot | Indicative of potential publication bias |

Abbreviations: CT, *Chlamydia trachomatis*; HIV, Human immunodeficiency virus; LFK, Luis Furuya-Kanamori; NG, *Neisseria gonorrhoeae*; STI, Sexually transmitted infection.

A minimum of three studies was required to perform this assessment.

^*^The term “men who have sex with men” is used inclusively and encompasses men who have sex with men, transgender people, and male or transgender sex workers.

^†|^Adverse pregnancy or birth outcomes were defined to include miscarriage, ectopic pregnancy, stillbirth, preterm delivery, small-for-gestational-age infants, and related complications.
^‡^Other populations include groups with an undetermined risk of acquiring NG infection, such as cervical cancer patients, individuals evaluated following sexual assault, and mixed or undefined populations.

# Fig D. Publication bias plots for urogenital infection. Doi plots assessing publication bias among studies reporting urogenital NG prevalence in sub-Saharan Africa.

| A. General populations  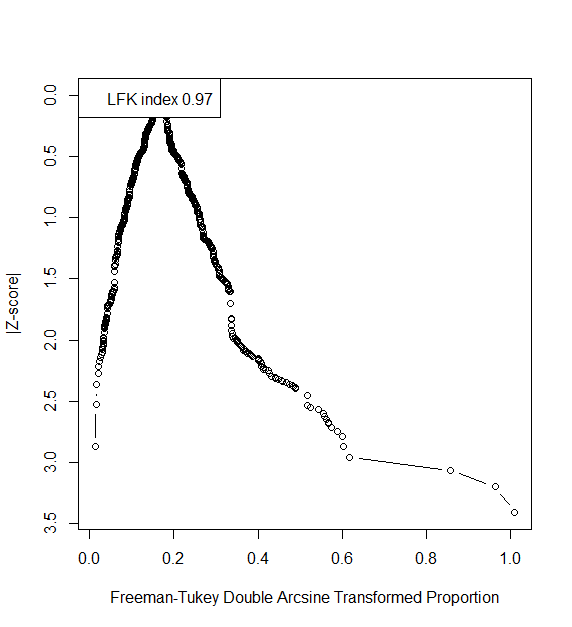 | B. Intermediate-risk populations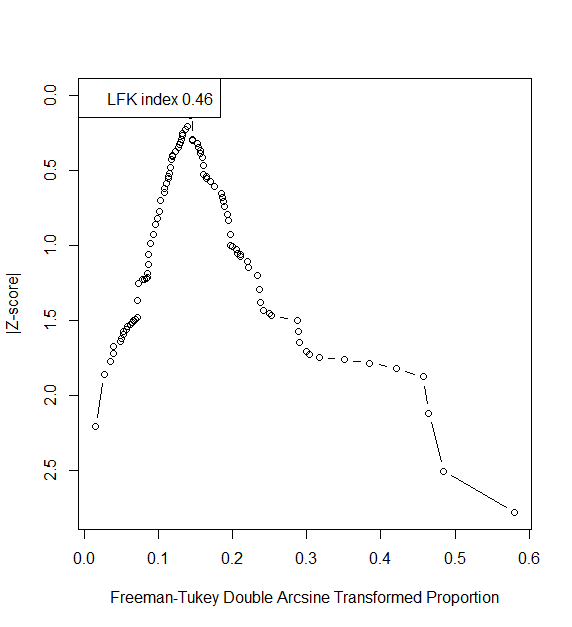 | C. Female sex workers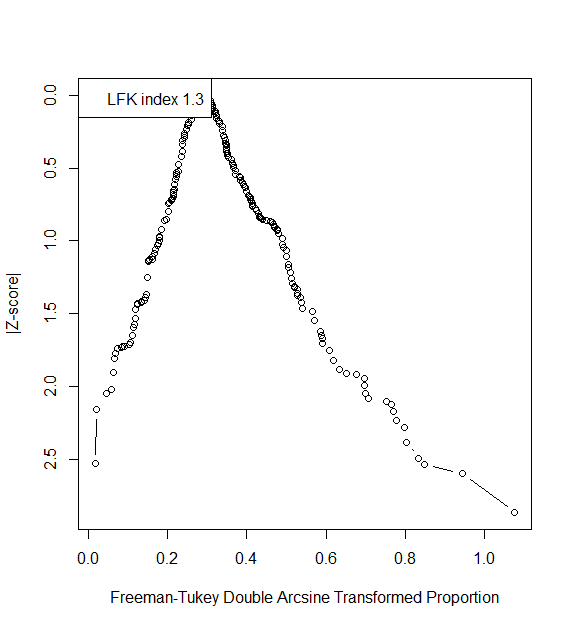 |
| --- | --- | --- |
| D. Men who have sex with men^*^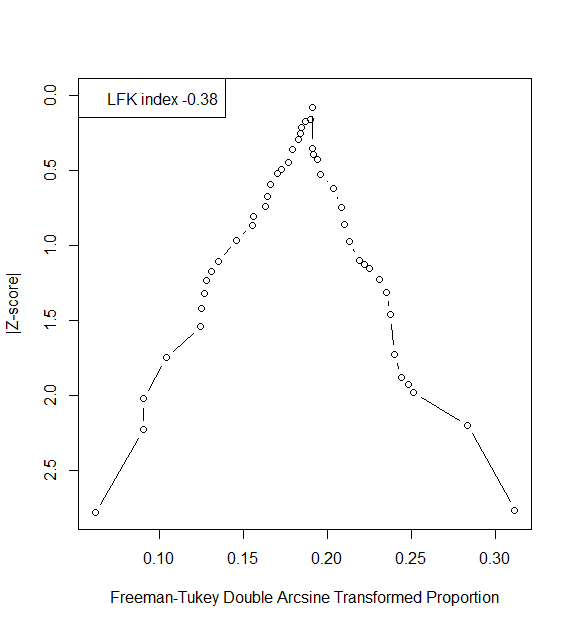 | E. Symptomatic women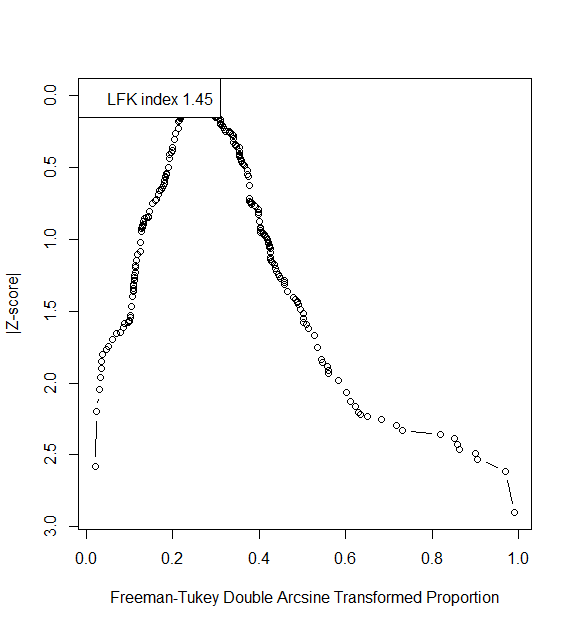 | F. Symptomatic men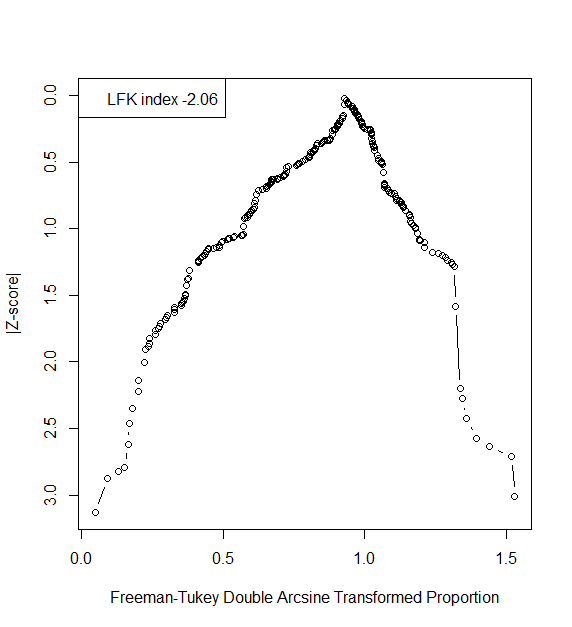 |
| G. Symptomatic women and men  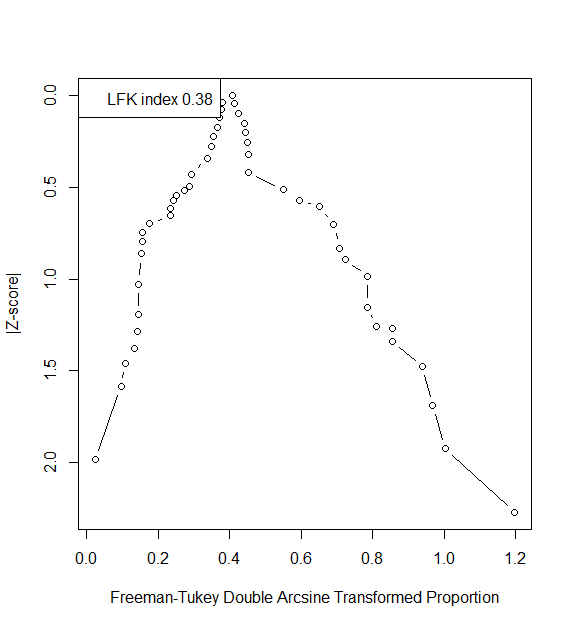 | H. Infertility clinic attendees  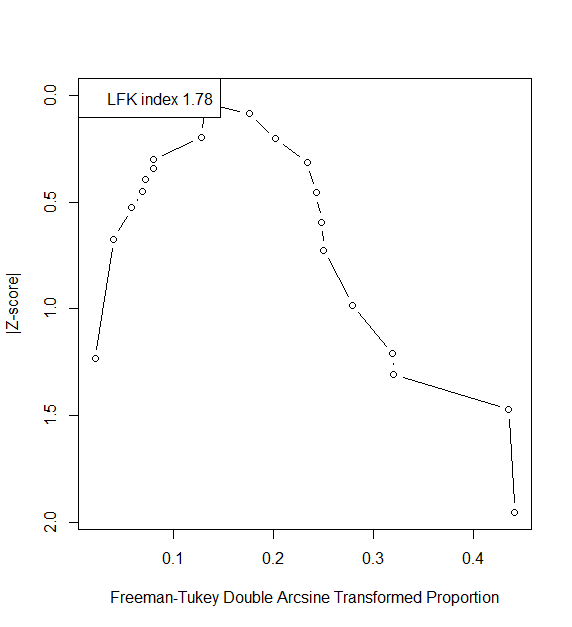 | I. Women with adverse pregnancy or birth outcomes^†^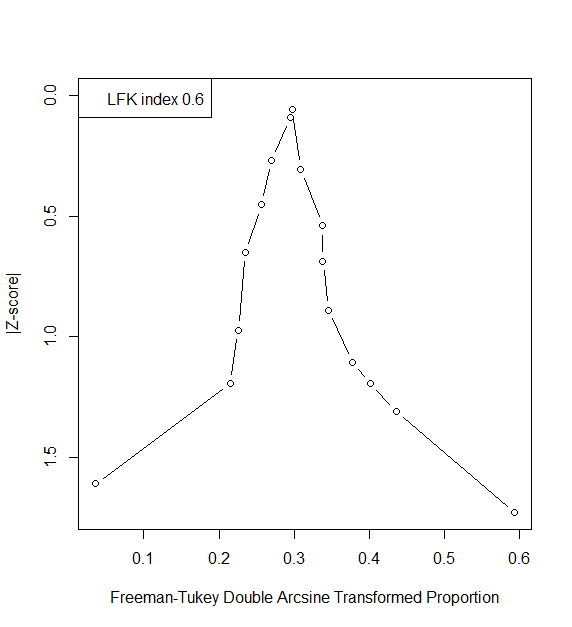 |
| J. STI clinic attendees  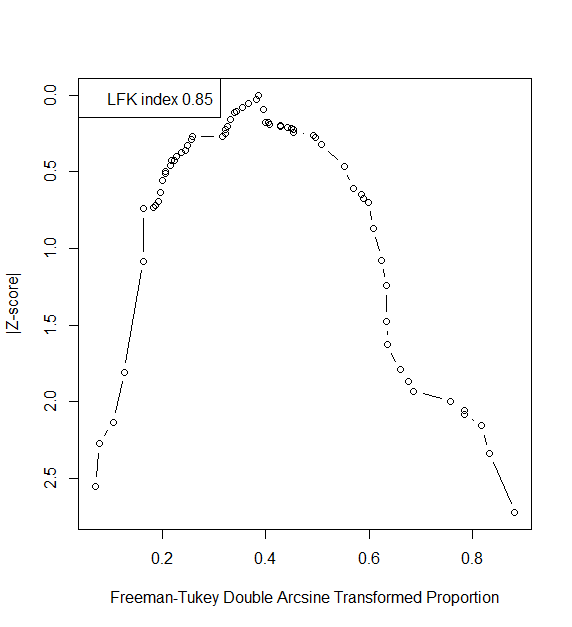 | K. Individuals living with HIV and individuals in HIV-discordant couples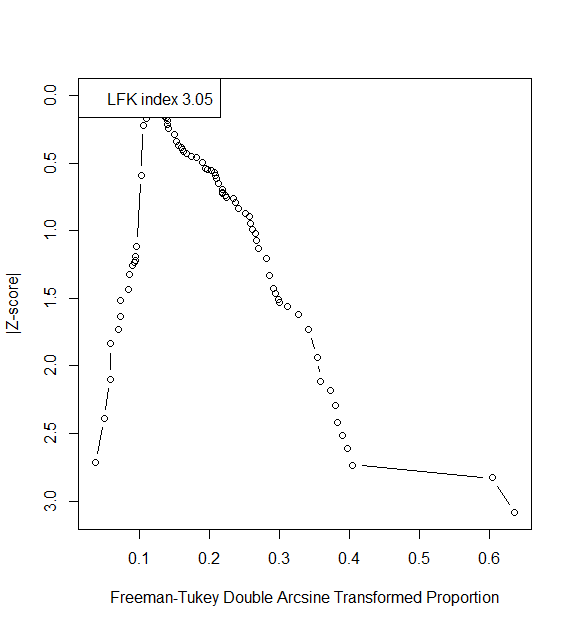 | L. Sexual contacts of persons infected with NG/CT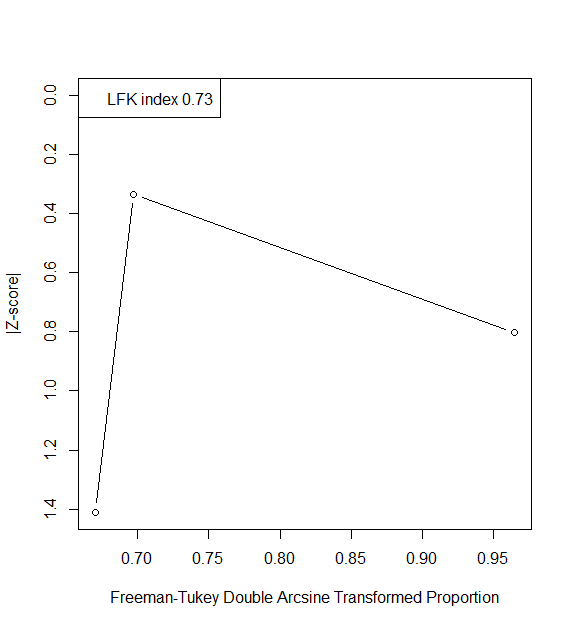 |
| M. Patients with confirmed or suspected STIs and related infections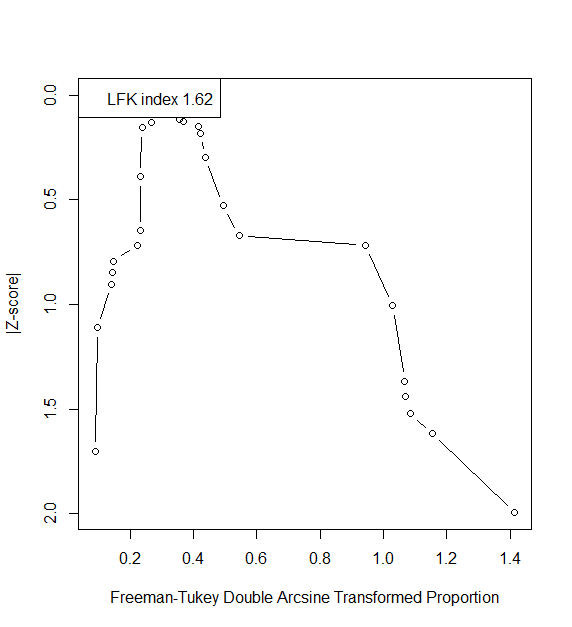 | N. Other populations^‡^  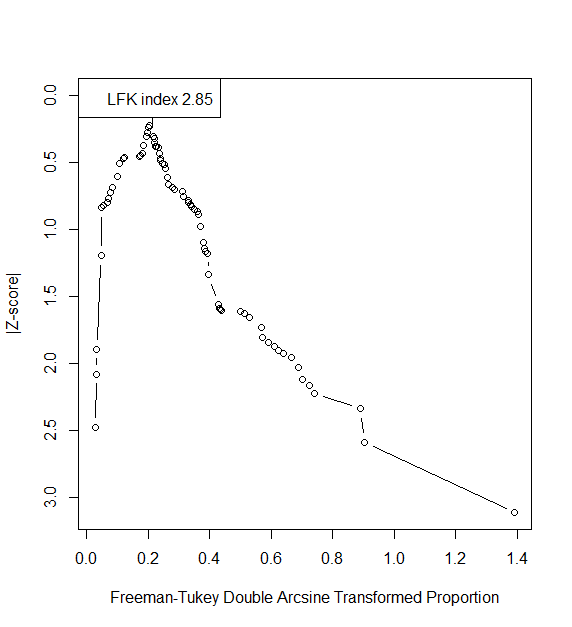 |  |

Abbreviations: CT, *Chlamydia trachomatis*; HIV, Human immunodeficiency virus; LFK, Luis Furuya-Kanamori; NG, *Neisseria gonorrhoeae*; STI, Sexually transmitted infection.

^*^The term “men who have sex with men” is used inclusively and encompasses men who have sex with men, transgender people, and male or transgender sex workers.

^†^Adverse pregnancy or birth outcomes were defined to include miscarriage, ectopic pregnancy, stillbirth, preterm delivery, small-for-gestational-age infants, and related complications.
^‡^Other populations include groups with an undetermined risk of acquiring NG infection, such as cervical cancer patients, individuals evaluated following sexual assault, and mixed or undefined populations.

# Fig E. Publication bias plots for anorectal infection. Doi plots assessing publication bias among studies reporting anorectal NG prevalence in sub-Saharan Africa.

| A. General populations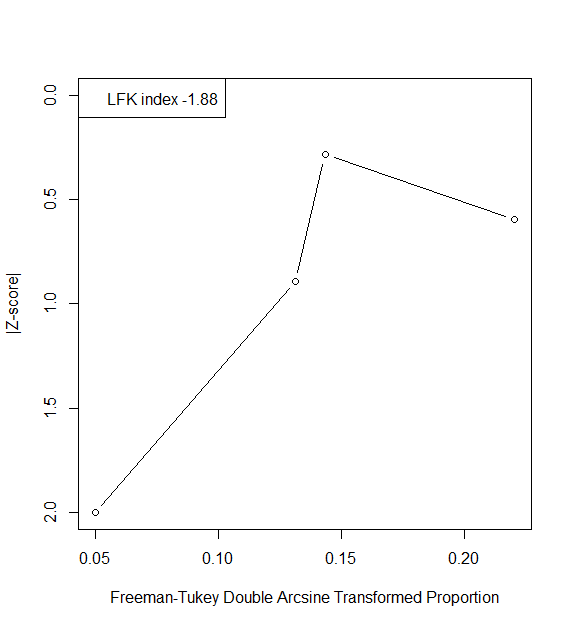 | B. Female sex workers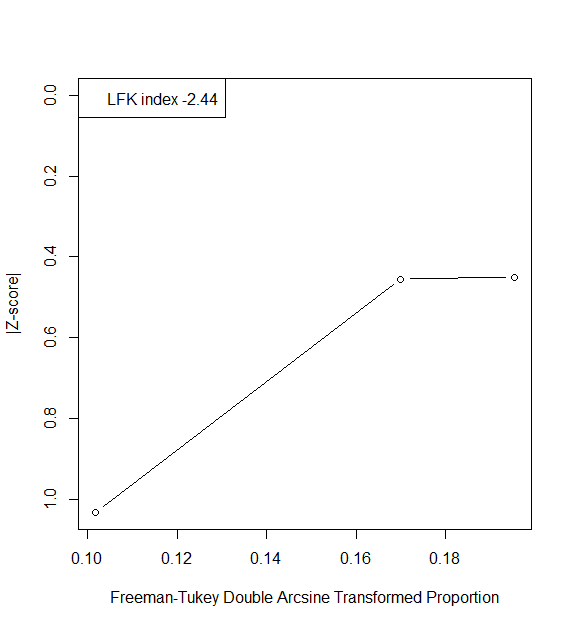 | C. Men who have sex with men^*^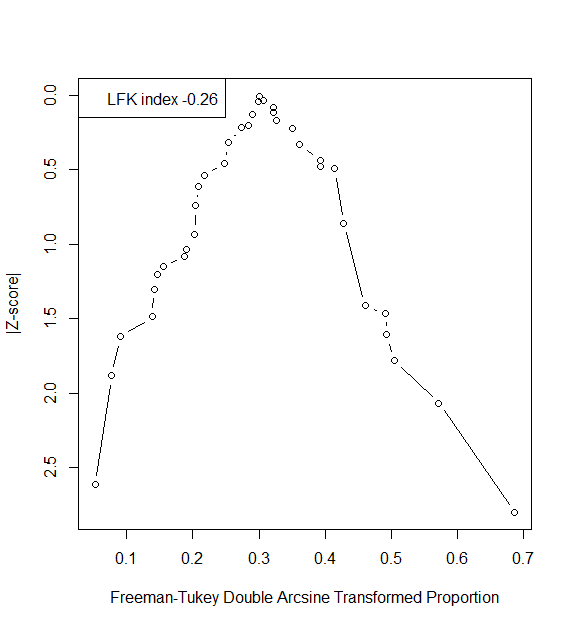 |
| --- | --- | --- |
| D. Symptomatic men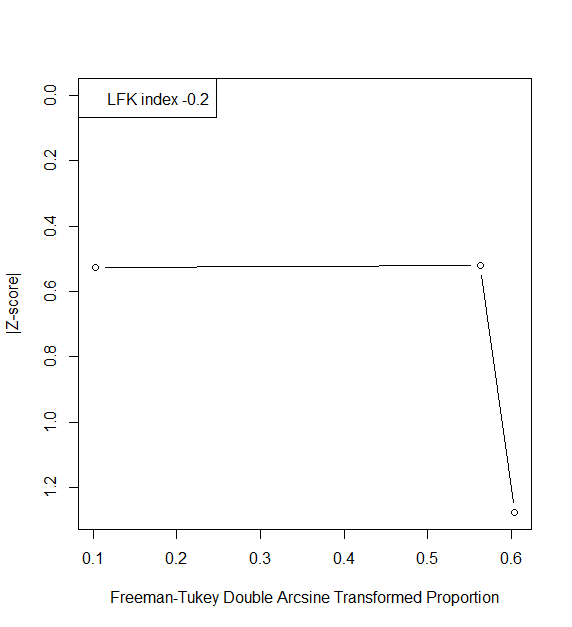 |  |  |

Abbreviations: LFK, Luis Furuya-Kanamori; NG, *Neisseria gonorrhoeae*.

^*^The term “men who have sex with men” is used inclusively and encompasses men who have sex with men, transgender people, and male or transgender sex workers.

# Fig F. Publication bias plots for oropharyngeal infection. Doi plots assessing publication bias among studies reporting oropharyngeal NG prevalence in sub-Saharan Africa.

| A. General populations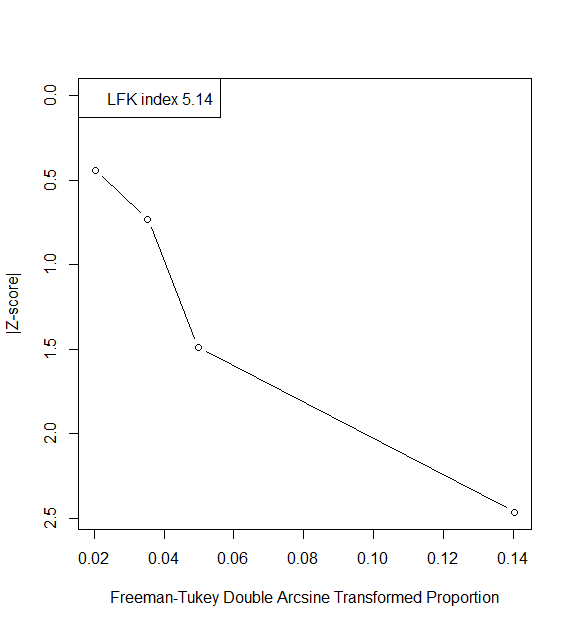 | B. Men who have sex with men^*^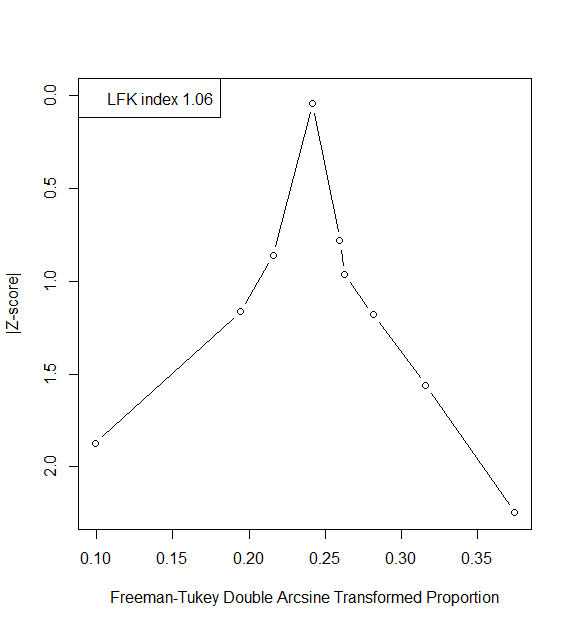 | C. Symptomatic men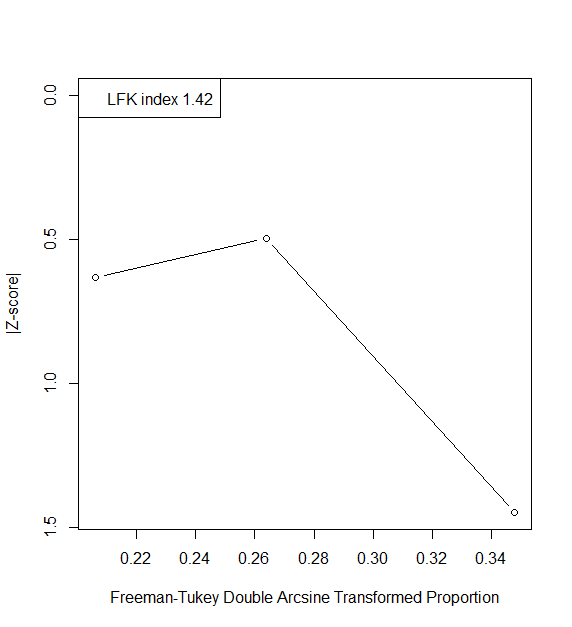 |
| --- | --- | --- |
| Abbreviations: LFK, Luis Furuya-Kanamori; NG, *Neisseria gonorrhoeae*.  ^*^The term “men who have sex with men” is used inclusively and encompasses men who have sex with men, transgender people, and male or transgender sex workers. | | |

# Table G. NG prevalence estimates by assay type. Pooled mean prevalence of NG infection in sub-Saharan Africa, stratified by population type, anatomical site, and assay type.

| **Population type** | **Stratified prevalence measures** | **Sample** | **NG prevalence (%)** | | **Pooled mean NG prevalence (%)** | **Heterogeneity measures** | | |
| --- | --- | --- | --- | --- | --- | --- | --- | --- |
|  | **Total n** | **Total N** | **Range** | **Median** | **Mean**  **(95% CI)** | **Q^*^**  **(p-value)** | **I²**^†^ **(%)**  **(95% CI)** | **Prediction interval^‡^ (%)** |
| **Urogenital specimens** | | | | | | | | |
| **General populations** | | | | | | | | |
| NAAT/PCR | 412 | 209,842 | 0.0-71.8 | 2.5 | 2.9 (2.5-3.2) | 6,780.0 (p <0.001) | 93.9 (93.5-94.3) | 0.0-13.3 |
| Culture^§^ | 245 | 86,619 | 0.0-32.1 | 3.0 | 3.4 (2.8-3.9) | 3,218.9 (p <0.001) | 92.4 (91.7-93.1) | 0.0-15.3 |
| Gram stain/microscopy^¶^ | 27 | 7,474 | 0.0-32.0 | 3.6 | 6.1 (3.0-10.1) | 790.7 (p <0.001) | 96.7 (96.0-97.3) | 0.0-36.0 |
| Rapid test | 4 | 1,541 | 0.1-8.5 | 1.2 | 1.8 (0.0-6.2) | 58.1 (p <0.001) | 94.8 (89.8-97.4) | 0.0-26.6 |
| ELISA/EIA | 3 | 457 | 7.0-33.3 | 7.1 | 13.4 (2.0-32.2) | 29.3 (p <0.001) | 93.2 (83.4-97.2) | 0.0-95.5 |
| Mixed assays | 1 | 185 | - | - | 7.6 (4.2-12.4) | - | - | - |
| Unclear assays | 14 | 14,938 | 0.8-10.0 | 5.0 | 3.7 (2.3-5.4) | 151.6 (p <0.001) | 91.4 (87.4-94.2) | 0.0-11.2 |
| **Overall** | **706** | **321,056** | **0.0-71.8** | **2**.**8** | **3**.**2 (2**.**9-3**.**5)** | **11,265**.**3 (p <0**.**001)** | **93.7 (93.4-94.0)** | **0**.**0-15**.**0** |
| **Intermediate-risk populations** | |  | | | |  |  |  |
| NAAT/PCR | 78 | 32,114 | 0.0-30.0 | 2.0 | 2.6 (1.8-3.5) | 925.0 (p <0.001) | 91.7 (90.2-92.9) | 0.0-13.1 |
| Culture^§^ | 11 | 4,317 | 0.0-8.1 | 1.1 | 1.3 (0.4-2.7) | 101.5 (p <0.001) | 90.2 (84.4-93.8) | 0.0-8.1 |
| Gram stain/microscopy^¶^ | 2 | 610 | 1.0-7.9 | 4.5 | 4.6 (3.1-6.6) | - | - | - |
| Rapid test | 1 | 328 | - | - | 0.0 (0.0-1.1) | - | - | - |
| Unclear assays | 7 | 1,104 | 0.0-5.5 | 1.0 | 0.8 (0.3-1.5) | 1,126.0 (p <0.001) | 18.8 (0.0-62.6) | 0.2-1.8 |
| **Overall** | **99** | **38,473** | **0**.**0-30**.**0** | **1**.**7** | **2**.**3 (1**.**6-3**.**0)** | **925**.**0 (p <0**.**001)** | **91**.**3 (90**.**0-92**.**5)** | **0**.**0-11**.**7** |
| **Female sex workers** | | | | | | | | |
| NAAT/PCR | 132 | 43,905 | 0.0-47.9 | 9.2 | 10.1 (8.5-11.7) | 3,214.0 (p <0.001) | 95.9 (95.5-96.3) | 0.0-34.0 |
| Culture^§^ | 49 | 15,691 | 0.0-77.5 | 16.0 | 17.8 (12.5-23.6) | 2,818.3 (p <0.001) | 98.3 (98.1-98.5) | 0.0-66.2 |
| Gram stain/microscopy^¶^ | 4 | 1,602 | 5.7-20.0 | 6.9 | 8.9 (4.1-15.1) | 19.9 (p <0.001) | 84.9 (62.5-93.9) | 0.0-35.5 |
| Rapid test | 6 | 2,606 | 0.0-13.0 | 6.3 | 5.7 (1.9-11.2) | 127.2 (p <0.001) | 96.1 (93.6-97.6) | 0.0-30.3 |
| Mixed assays | 1 | 639 | - | - | 9.4 (7.2-11.9) | - | - | - |
| Unclear assays | 8 | 2,966 | 1.8-18.6 | 12.9 | 9.9 (5.9-14.9) | 114.3 (p <0.001) | 93.9 (90.1-96.2) | 0.3-29.4 |
| **Overall** | **200** | **67,409** | **0**.**0-77**.**5** | **10**.**3** | **11**.**5 (9**.**9-13**.**2)** | **6,778**.**0 (p <0**.**001)** | **97**.**1 (96**.**8-97**.**3)** | **0**.**0-42**.**3** |
| **Men who have sex with men^\|\|^** | | | | | | | | |
| NAAT/PCR | 46 | 11,993 | 0.0-9.0 | 3.0 | 2.8 (2.4-3.3) | 73.3 (p = 0.005) | 38.6 (12.1-57.0) | 1.10-5.1 |
| **Overall** | **46** | **11,993** | **0**.**0-9**.**0** | **3**.**0** | **2**.**8 (2**.**4-3**.**3)** | **73**.**3 (p = 0**.**005)** | **38**.**6 (12**.**1-57**.**0)** | **1**.**10-5**.**1** |
| **Symptomatic women** | | | | | | | | |
| NAAT/PCR | 83 | 21,573 | 0.0-68.3 | 10.2 | 10.1 (8.3-12.1) | 1,334.4 (p <0.001) | 93.9 (92.9-94.7) | 0.0-32.3 |
| Culture^§^ | 137 | 28,479 | 0.0-61.9 | 6.8 | 8.1 (6.4-10.0) | 2,423.4 (p <0.001) | 94.4 (93.8-95.0) | 0.0-36.6 |
| Gram stain/microscopy^¶^ | 9 | 3,153 | 1.4-70.1 | 13.2 | 16.5 (5.6-31.4) | 627.1 (p <0.001) | 98.7 (98.3-99.0) | 0.0-77.0 |
| Rapid test | 1 | 86 | - | - | 1.2 (0.0-6.3) | - | - | - |
| Mixed assays | 4 | 800 | 0.8-3.2 | 2.0 | 2.0 (1.1-3.2) | 2.9 (p = 0.415) | 0.0 (0.0-84.7) | 0.5-4.4 |
| Unclear assays | 3 | 509 | 0.3-62.0 | 7.0 | 15.5 (0.0-62.1) | 130.9 (p <0.001) | 98.5 (97.3-99.1) | 0.0-100.0 |
| **Overall** | **237** | **54,600** | **0**.**0-70**.**1** | **8**.**0** | **9**.**0 (7**.**7-10**.**4)** | **4,957**.**7 (p <0**.**001)** | **95**.**2 (94**.**9-95**.**6)** | **0**.**0-36**.**9** |
| **Symptomatic men** | | | | | | | | |
| NAAT/PCR | 58 | 14,489 | 4.9-100.0 | 66.6 | 61.1 (54.2-67.8) | 4,595.8 (p <0.001) | 98.8 (98.6-98.9) | 12.6-98.4 |
| Culture^§^ | 121 | 22,384 | 0.0-100.0 | 51.9 | 45.8 (39.8-51.8) | 7,864.8 (p <0.001) | 98.5 (98.4-98.6) | 0.2-97.7 |
| Gram stain/microscopy^¶^ | 59 | 18,902 | 2.5-97.5 | 64.0 | 55.2 (47.3-62.9) | 6,215.5 (p <0.001) | 99.1 (99.0- 99.1) | 5.0-98.9 |
| Rapid test | 1 | 52 | - | - | 28.8 (17.1-43.1) | - | - | - |
| Mixed assays | 3 | 606 | 0.0-62.3 | 15.8 | 19.4 (0.0-66.3) | 270.5 (p <0.001) | 99.3 (98.8-99.5) | 0.0-100.0 |
| Unclear assays | 6 | 787 | 5.0-94.0 | 67.8 | 58.4 (26.1-87.2) | 351.4 (p <0.001) | 98.6 (98.0-99.0) | 0.0-100.0 |
| **Overall** | **248** | **57,220** | **0**.**0-100**.**0** | **57**.**3** | **51**.**5 (47**.**5-55**.**5)** | **21,093**.**3 (p <0**.**001)** | **98**.**8 (98**.**8-98**.**9)** | **2**.**5-98**.**5** |
| **Symptomatic women and men** | | | | | | | | |
| NAAT/PCR | 2 | 359 | 16.9-87.0 | 52.0 | 55.7 (50.4-60.9) | - | - | - |
| Culture^§^ | 27 | 5,017 | 0.0-71.4 | 18.7 | 24.6 (15.8-34.5) | 1,712.6 (p <0.001) | 98.5 (98.2-98.7) | 0.0-80.7 |
| Gram stain/microscopy^¶^ | 2 | 725 | 19.0-50.2 | 34.6 | 33.0 (29.5-36.5) | - | - | - |
| Mixed assays | 1 | 118 | - | - | 5.1 (1.9-10.7) | - | - | - |
| Unclear assays | 16 | 2,374 | 0.6-18.9 | 6.2 | 6.4 (3.7-9.7) | 2,739.5 (p <0.001) | 88.6 (83.1-92.3) | 0.0-23.6 |
| **Overall** | **48** | **8,593** | **0**.**0-87**.**0** | **13**.**4** | **18**.**2 (12**.**4-24**.**9)** | **1,712**.**6 (p <0**.**001)** | **98**.**3 (98**.**1-98**.**5)** | **0**.**0-72**.**1** |
| **Infertility clinic attendees** | | | | | | | | |
| NAAT/PCR | 4 | 505 | 0.0-7.0 | 2.7 | 2.8 (0.5-6.5) | 10.5 (p = 0.015) | 71.5 (19.0-90.0) | 0.0-18.5 |
| Culture^§^ | 13 | 1,502 | 0.0-17.4 | 2.5 | 2.4 (0.5-5.4) | 91.6 (p <0.001) | 86.9 (79.3-91.7) | 0.0-18.0 |
| Gram stain/microscopy^¶^ | 2 | 192 | 0.0-5.8 | 2.9 | 2.5 (0.0-10.7) | - | - | - |
| Unclear assays | 1 | 117 | - | - | 17.9 (11.5-26.1) | - | - | - |
| **Overall** | **20** | **2,316** | **0**.**0-17**.**9** | **3**.**3** | **3**.**0 (1**.**2-5**.**4)** | **152**.**6 (p <0**.**001)** | **87**.**6 (82**.**2-91**.**3)** | **0**.**0-17**.**7** |
| **Women with adverse pregnancy or birth outcomes^**^** | | | | | | | | |
| NAAT/PCR | 4 | 398 | 7.7-14.3 | 9.9 | 9.5 (6.7-12.7) | 1.3 (p = 0.730) | 0.0 (0.0- 84.7) | 5.2-14.9 |
| Culture^§^ | 11 | 1,286 | 0.0-17.1 | 6.9 | 6.4 (3.4-10.1) | 54.6 (p <0.001) | 81.7 (68.3- 89.4) | 0.0-21.6 |
| Gram stain/microscopy^¶^ | 1 | 154 | - | - | 31.2 (24.0-39.1) | - | - | - |
| **Overall** | **16** | **1,838** | **0**.**0-31**.**2** | **8**.**7** | **8**.**6 (5**.**3-12**.**6)** | **124**.**8 (p <0**.**001)** | **88**.**0 (82**.**1-91**.**9)** | **0**.**0-28**.**0** |
| **STI clinic attendees** | | | | | | | | |
| NAAT/PCR | 18 | 2,827 | 4.4-55.0 | 17.6 | 22.0 (13.9-31.3) | 573.9 (p <0.001) | 97.0 (96.2-97.7) | 0.0-67.8 |
| Culture^§^ | 37 | 19,031 | 0.0-59.7 | 13.9 | 13.2 (9.0-18.1) | 2,803.0 (p <0.001) | 98.7 (98.5-98.9) | 0.0-49.9 |
| Gram stain/microscopy^¶^ | 6 | 4,017 | 3.3-35.3 | 17.1 | 19.0 (9.3-31.0) | 263.2 (p <0.001) | 98.1 (97.2-98.7) | 0.0-63.0 |
| ELISA/EIA | 2 | 120 | 10.7-16.7 | 13.7 | 13.3 (7.8-20.7) | - | - |  |
| Mixed assays | 1 | 158 | - | - | 3.2 (1.0-7.2) | - | - | - |
| Unclear assays | 1 | 422 | - | - | 5.0 (3.1-7.5) | - | - | - |
| **Overall** | **65** | **26,575** | **0**.**0-59**.**7** | **13**.**9** | **15**.**5 (12**.**0-19**.**5)** | **4,186**.**6 (p <0**.**001)** | **98**.**5 (98**.**3-98**.**6)** | **0**.**0-53**.**2** |
| **Individuals living with HIV and individuals in HIV-discordant couples** | | | | | | | | |
| NAAT/PCR | 45 | 19,046 | 0.0-14.0 | 2.6 | 3.1 (2.2-4.1) | 345.8 (p <0.001) | 87.3 (83.9-90.0) | 0.0-10.9 |
| Culture^§^ | 32 | 8,287 | 0.0-35.0 | 3.5 | 4.2 (2.5-6.3) | 365.8 (p <0.001) | 1.5 (89.1- 93.4) | 0.0-19.6 |
| Gram stain/microscopy^¶^ | 1 | 432 | - | - | 0.2 (0.0-1.3) | - | - | - |
| Unclear assays | 3 | 389 | 1.3-13.5 | 2.5 | 4.5 (0.2-12.9) | 12.4 (p <0.001) | 83.9 (51.6-94.6) | 0.0-55.4 |
| **Overall** | **81** | **28,154** | **0**.**0-35**.**0** | **3**.**0** | **3**.**5 (2**.**6-4**.**4)** | **797**.**3 (p <0**.**001)** | **90**.**0 (88**.**2-91**.**5)** | **0**.**0-13**.**9** |
| **Sexual contacts of persons infected with NG/CT** | | | | | | | | |
| NAAT/PCR | 1 | 21 | - | - | 38.1 (18.1-61.6) | - | - | - |
| Culture^§^ | 1 | 56 | - | - | 67.9 (54.0-79.7) | - | - | - |
| Gram stain/microscopy^¶^ | 1 | 56 | - | - | 41.1 (28.1-55.0) | - | - | - |
| **Overall** | **3** | **133** | **38**.**0-68**.**0** | **41**.**0** | **50**.**0 (31**.**0-69**.**1)** | **9**.**9 (p = 0**.**007)** | **79**.**9 (36**.**3-93**.**6)** | **0**.**0-100**.**0** |
| **Patients with confirmed or suspected STIs and related infections** | | | | | | | | |
| NAAT/PCR | 3 | 1,545 | 1.0-5.3 | 3.0 | 3.1 (1.1-6.0) | 6.5 (p = 0.039) | 69.3 (0.0-91.0) | 0.0-18.1 |
| Culture^§^ | 10 | 1,056 | 1.5-97.6 | 21.1 | 37.6 (13.9-64.9) | 880.2 (p <0.001) | 99.0 (98.7-99.2) | 0.0-100.0 |
| Gram stain/microscopy^¶^ | 7 | 2,093 | 4.6-84.0 | 22.4 | 38.2 (12.8-67.7) | 750.1 (p <0.001) | 99.2 (99.0-99.4) | 0.0-100.0 |
| Mixed assays | 2 | 1,358 | 0.7-0.9 | - | 0.8 (0.4-1.4) | - | - | - |
| Unclear assays | 3 | 1,567 | 5.0-18.0 | 11.6 | 10.9 (4.6-19.4) | 34.3 (p <0.001) | 94.2 (86.3-97.5) | 0.0-56.5 |
| **Overall** | **25** | **7,619** | **0**.**7-97**.**6** | **12**.**2** | **24**.**3 (12**.**0-39**.**1)** | **4,584**.**7 (p <0**.**001)** | **99**.**5 (99**.**4-99**.**5)** | **0**.**0-96**.**2** |
| **Other populations^††^** | | | | | | | | |
| NAAT/PCR | 27 | 8,370 | 0.9-41.5 | 6.0 | 7.8 (5.3-10.9) | 421.4 (p <0.001) | 93.8 (92.1- 95.2) | 0.0-27.1 |
| Culture^§^ | 39 | 13,880 | 0.0-97.8 | 12.1 | 14.9 (9.1-21.9) | 1,914.8 (p <0.001) | 98.0 (97.7- 98.3) | 0.0-67.9 |
| Gram stain/microscopy^¶^ | 5 | 1,336 | 0.0-33.8 | 1.0 | 4.5 (0.0-17.6) | 119.8 (p <0.001) | 96.7 (94.4-98.0) | 0.0-66.8 |
| ELISA/EIA | 1 | 433 | - | - | 4.6 (2.8-7.0) | - | - | - |
| Unclear assays | 3 | 422 | 0.0-0.0 | 0.0 | 0.0 (0.0-0.3) | 0.4 (p = 0.828) | 0.0 (0.0- 89.6) | 0.0-1.5 |
| **Overall** | **75** | **24,441** | **0**.**0-97**.**8** | **7**.**0** | **10**.**4 (7**.**2-14**.**0)** | **2,628**.**0 (p <0**.**001)** | **97**.**2 (96**.**8-97**.**5)** | **0**.**0-51**.**9** |
| **Anorectal specimens** | | | | | | | | |
| **General populations** |  |  |  |  |  |  |  |  |
| NAAT/PCR | 1 | 612 | - | - | 1.6 (0.8-3.0) | - | - | - |
| Culture^§^ | 3 | 1,568 | 0.0-4.8 | 1.9 | 1.9 (0.1-5.5) | 14.5 (p <0.001) | 86.2 (60.0-95.2) | 0.0-25.8 |
| **Overall** | **4** | **2,180** | **0**.**0-4**.**8** | **1**.**7** | **1**.**9 (0**.**5-4**.**2)** | **22**.**0 (p <0**.**001)** | **86**.**3 (66**.**8-94**.**4)** | **0**.**0-12**.**3** |
| **Intermediate-risk populations** | | | | | | | | |
| NAAT/PCR | 2 | 598 | 0.0-0.0 | 0.0 | 0.0 (0.0-0.6) | - | - | - |
| **Overall** | **2** | **598** | **0**.**0-0**.**0** | **0**.**0** | **0**.**0 (0**.**0-0**.**6)** | **-** | **-** | **-** |
| **Female sex workers** | | | | | | | | |
| NAAT/PCR | 3 | 1,115 | 0.9-3.7 | 2.0 | 2.0 (0.4-4.5) | 8.1 (p = 0.017) | 75.4 (18.6-92.5) | 0.0-15.9 |
| **Overall** | **3** | **1,115** | **0**.**9-3**.**7** | **2**.**0** | **2**.**0 (0**.**4-4**.**5)** | **8**.**1 (p = 0**.**017)** | **75**.**4 (18**.**6-92**.**5)** | **0**.**0-15**.**9** |
| **Men who have sex with men^\|\|^** | | | | | | | | |
| NAAT/PCR | 35 | 9,843 | 0.0-29.1 | 8.0 | 7.7 (5.5-10.2) | 582.2 (p <0.001) | 94.2 (92.8-95.3) | 0.0-26.2 |
| Unclear assays | 1 | 50 | - | - | 40.0 (26.4-54.8) | - | - | - |
| **Overall** | **36** | **9,893** | **0**.**0-40**.**0** | **8**.**0** | **8**.**3 (5**.**8-11**.**0)** | **610**.**9 (p <0**.**001)** | **94**.**3 (92**.**9-95**.**4)** | **0**.**0-29**.**2** |
| **Symptomatic men** | | | | | | | | |
| NAAT/PCR | 1 | 78 | - | - | 32.1 (21.9-43.6) | - | - | - |
| Gram stain/microscopy^¶^ | 1 | 235 | - | - | 0.9 (0.1-3.0) | - | - | - |
| Unclear assays | 1 | 78 | - | - | 28.2 (18.6-39.5) | - | - | - |
| **Overall** | **3** | **391** | **0**.**9-32**.**1** | **28**.**0** | **16**.**3 (0**.**6-45**.**2)** | **87**.**4 (p <0**.**001)** | **97**.**7 (95**.**6-98**.**8)** | **0**.**0-100**.**0** |
| **Individuals living with HIV and individuals in HIV-discordant couples** | | | | | | | | |
| NAAT/PCR | 1 | 100 | - | - | 22.0 (14.3-31.4) | - | - | - |
| Culture^§^ | 1 | 16 | - | - | 31.3 (11.0-58.7) | - | - | - |
| **Overall** | **2** | **116** | **22**.**0-31**.**3** | **26**.**6** | **23**.**3 (15**.**9-32**.**0)** | **-** | **-** | **-** |
| **Oropharyngeal specimens** | | | | | | | | |
| **General populations** | | | | | | | | |
| NAAT/PCR | 1 | 603 | - | - | 0.0 (0.0-0.6) | - | - | - |
| Culture^§^ | 3 | 312 | 0.0-0.0 | 0.0 | 0.0 (0.0-0.0) | 0.5 (p = 0.763) | 0.0 (0.0- 89.6) | 0.0-1.4 |
| **Overall** | **4** | **915** | **0**.**0-0**.**0** | **0**.**0** | **0**.**0 (0**.**0-0**.**0)** | **1**.**0 (p = 0**.**799)** | **0**.**0 (0**.**0-84**.**7)** | **0**.**0-0**.**0** |
| **Men who have sex with men^\|\|^** | | | | | | | | |
| NAAT/PCR | 9 | 4,091 | 0.9-13.0 | 6.4 | 5.7 (3.6-8.3) | 40.2 (p <0.001) | 80.1 (63.0-89.3) | 0.5-15.7 |
| **Overall** | **9** | **4,091** | **0**.**9-13**.**0** | **6**.**4** | **5**.**7 (3**.**6-8**.**3)** | **40**.**2 (p <0**.**001)** | **80**.**1 (63**.**0-89**.**3)** | **0**.**5-15**.**7** |
| **Symptomatic men** | | | | | | | | |
| Culture^§^ | 3 | 200 | 3.8-10.4 | 6.2 | 5.1 (2.2-8.8) | 2.0 (p = 0.372) | 0.0 (0.0-89.6) | 0.2-14.3 |
| **Overall** | **3** | **200** | **3**.**8-10**.**4** | **6**.**2** | **5**.**1 (2**.**2-8**.**8)** | **2**.**0 (p = 0**.**372)** | **0**.**0 (0**.**0-89**.**6)** | **0**.**2-14**.**3** |
| **Individuals living with HIV and individuals in HIV-discordant couples** | | | | | | | | |
| NAAT/PCR | 1 | 100 | - | - | 25.0 (16.9-34.7) | - | - | - |
| **Overall** | **1** | **100** | **-** | **-** | **25**.**0 (16**.**9-34**.**7)** | **-** | **-** | **-** |
| **Serological specimens** | | | | | | | | |
| **General populations** | | | | | | | | |
| Culture^§^ | 1 | 41 | - | - | 0.0 (0.0-8.6) | - | - | - |
| ELISA/EIA | 2 | 344 | 4.8-17.5 | 11.2 | 13.7 (10.2-17.8) | - | - | - |
| Blood tests for antibodies^‡‡^ | 6 | 238 | 7.5-28.0 | 26.5 | 21.4 (14.0-29.7) | 11.1 (p = 0.050) | 54.8 (0.0-81.9) | 4.5-45.4 |
| **Overall** | **9** | **623** | **0**.**0-28**.**0** | **18**.**0** | **15**.**0 (7**.**6-24**.**2)** | **47**.**6 (p <0**.**001)** | **83**.**2 (69**.**5-90**.**7)** | **0**.**0-50**.**7** |
| **Intermediate-risk populations** | | | | | | | | |
| Unclear assays | 1 | 2,650 | - | - | 1.6 (1.1-2.1) | **-** | **-** | **-** |
| **Overall** | **1** | **2,650** | **-** | **-** | **1**.**6 (1**.**1-2**.**1)** | - | - | - |
| **Female sex workers** | | | | | | | | |
| Unclear assays | 1 | 642 | - | - | 53.3 (49.3-57.2) | - | - | - |
| **Overall** | **1** | **642** | **-** | **-** | **53**.**3 (49**.**3-57**.**2)** | **-** | **-** | **-** |
| **Symptomatic women** | | | | | | | | |
| ELISA/EIA | 1 | 66 | - | - | 54.5 (41.8-66.9) | - | - | - |
| Blood tests for antibodies^‡‡^ | 2 | 345 | 12.5-32.8 | 22.7 | 16.5 (12.8-20.9) | - | - | - |
| **Overall** | **3** | **411** | **12**.**5-54**.**6** | **32**.**8** | **31**.**5 (10**.**2-58**.**0)** | **52**.**2 (p <0**.**001)** | **96**.**2 (91**.**9-98**.**2)** | **0**.**0-100**.**0** |
| **Symptomatic men** | | | | | | | | |
| Blood tests for antibodies^‡‡^ | 2 | 391 | 10.6-13.7 | 12.2 | 12.3 (9.2-15.9) | - | - | - |
| **Overall** | **2** | **391** | **10**.**6-13**.**7** | **12**.**2** | **12**.**3 (9**.**2-15**.**9)** | **-** | **-** | **-** |
| **Infertility clinic attendees** | | | | | | | | |
| ELISA/EIA | 2 | 227 | 25.0-51.1 | 38.1 | 40.5 (34.1-47.2) | - | - | - |
| Blood tests for antibodies^‡‡^ | 2 | 97 | 26.7-65.0 | 45.9 | 41.2 (31.3-51.7) | - | - | - |
| **Overall** | **4** | **324** | **25**.**0-65**.**0** | **38**.**9** | **41**.**1 (23**.**3-60**.**1)** | **29**.**8 (p <0**.**001)** | **89**.**9 (77**.**1-95**.**6)** | **0**.**0-96**.**1** |
| **Women with adverse pregnancy or birth outcomes^**^** | | | | | | | | |
| ELISA/EIA | 2 | 60 | 14.3-33.3 | 23.8 | 26.7 (16.1-39.7) | - | - | - |
| Blood tests for antibodies^‡‡^ | 2 | 90 | 49.0-49.0 | 49.0 | 48.9 (38.2-59.7) | - | - | - |
| **Overall** | **4** | **150** | **14**.**3-49**.**0** | **41**.**2** | **37**.**0 (22**.**3-53**.**0)** | **10**.**1 (p = 0**.**018)** | **70**.**4 (15**.**00-89**.**70)** | **1**.**2-85**.**1** |
| **Individuals living with HIV and individuals in HIV-discordant couples** | | | | | | | | |
| Blood tests for antibodies^‡‡^ | 1 | 20 | - | - | 30.0 (11.9-54.3) | **-** | **-** | **-** |
| **Overall** | **1** | **20** | **-** | **-** | **30**.**0 (11**.**9-54**.**3)** | **-** | **-** | **-** |
| **Patients with confirmed or suspected STIs and related infections** | | | | | | | | |
| ELISA/EIA | 1 | 100 | - | - | 97.0 (91.5-99.4) | **-** | **-** | **-** |
| **Overall** | **1** | **100** | **-** | **-** | **97**.**0 (91**.**5-99**.**4)** | - | - | - |
| **Other populations**^††^ | | | | | | | | |
| Blood tests for antibodies^‡‡^ | 5 | 2,222 | 31.1-68.8 | 47.2 | 49.4 (34.8-64.1) | 115.2 (p <0.001) | 96.5 (94.1-97.9) | 7.2-92.2 |
| **Overall** | **5** | **2,222** | **31**.**1-68**.**8** | **47**.**2** | **49**.**4 (34**.**8-64**.**1)** | **115**.**2 (p <0**.**001)** | **96**.**5 (94**.**1-97**.**9)** | **7**.**2-92**.**2** |

Abbreviations: CI, Confidence interval; CT, *Chlamydia trachomatis*; EIA, Enzyme immunoassay; ELISA, Enzyme-linked immunosorbent assay; HIV, Human immunodeficiency virus; NG, *Neisseria gonorrhoeae*; STI, Sexually transmitted infection.

A minimum of three studies was required to perform a meta-analysis.

^*^Q: The Cochran’s Q statistic is a measure assessing the existence of heterogeneity in pooled outcome measures, here NG prevalence.

^†^I^2^: A measure that assesses the magnitude of between-study variation that is due to true differences in NG prevalence across studies rather than chance.

^‡^Prediction interval: A measure that estimates the distribution (95% interval) of true NG prevalence around the estimated mean.

^§^Culture refers to microbiological culture results, with or without accompanying Gram stain analysis.

^¶^Gram stain/microscopy refers to diagnostic methods based on Gram stain alone, microscopy alone, Gram stain or microscopy confirmed by culture and/or PCR, or Gram stain or microscopy performed with or without culture.

^||^The term “men who have sex with men” is used inclusively and encompasses men who have sex with men, transgender people, and male or transgender sex workers.

^**^Adverse pregnancy or birth outcomes were defined to include miscarriage, ectopic pregnancy, stillbirth, preterm delivery, small-for-gestational-age infants, and related complications.

^††^Other populations include groups with an undetermined risk of acquiring NG infection, such as cervical cancer patients, individuals evaluated following sexual assault, and mixed or undefined populations.

^‡‡^Blood tests for antibodies include haemagglutination assays, complement fixation tests, and measurements of immunoglobulins (e.g., IgG, IgA).

# Fig G. Forest plots for urogenital infection. Forest plots presenting outcomes of the pooled mean NG prevalence in urogenital specimens among different populations in sub-Saharan Africa.

1. General populations

**
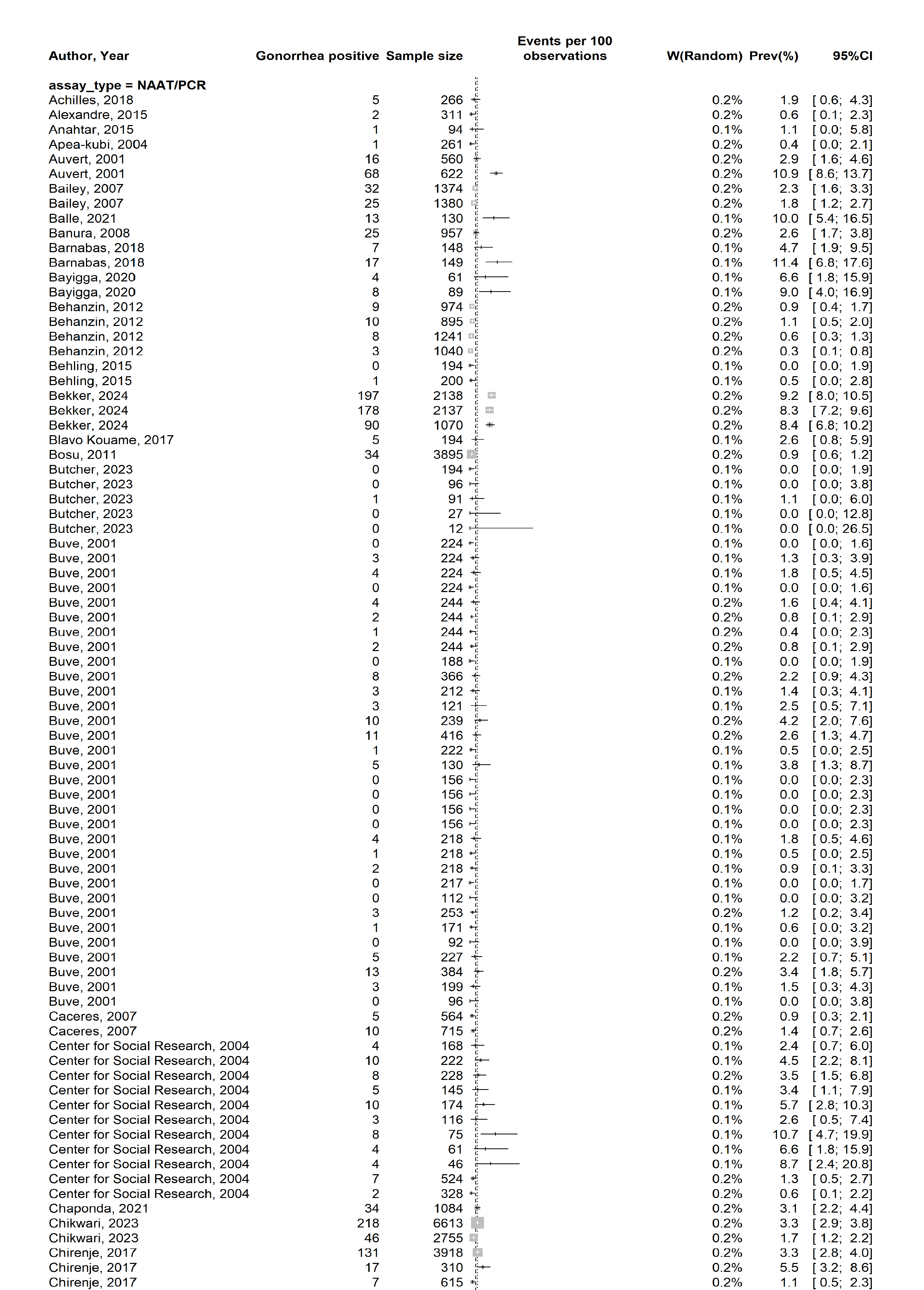
**

**
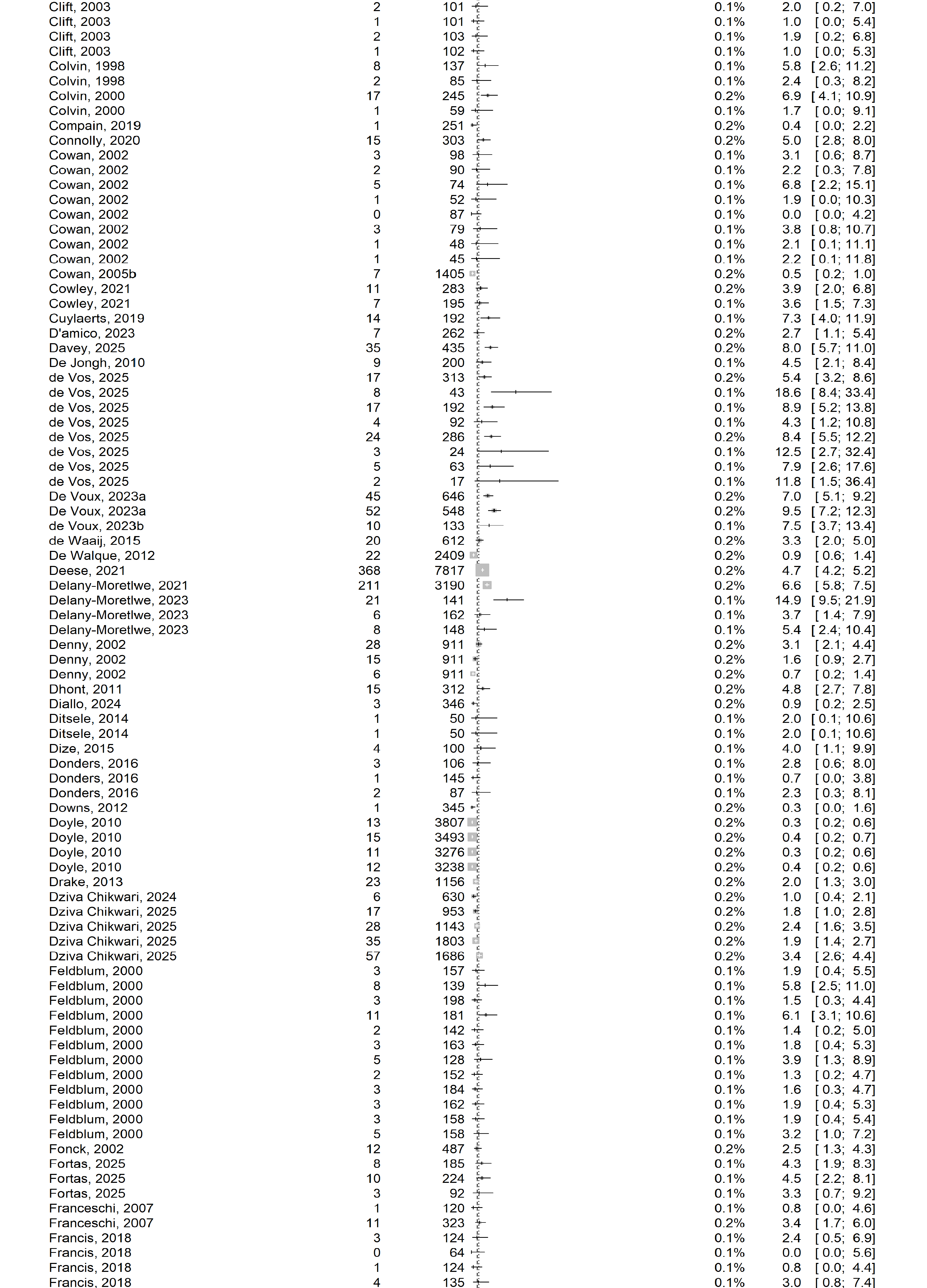
**

**
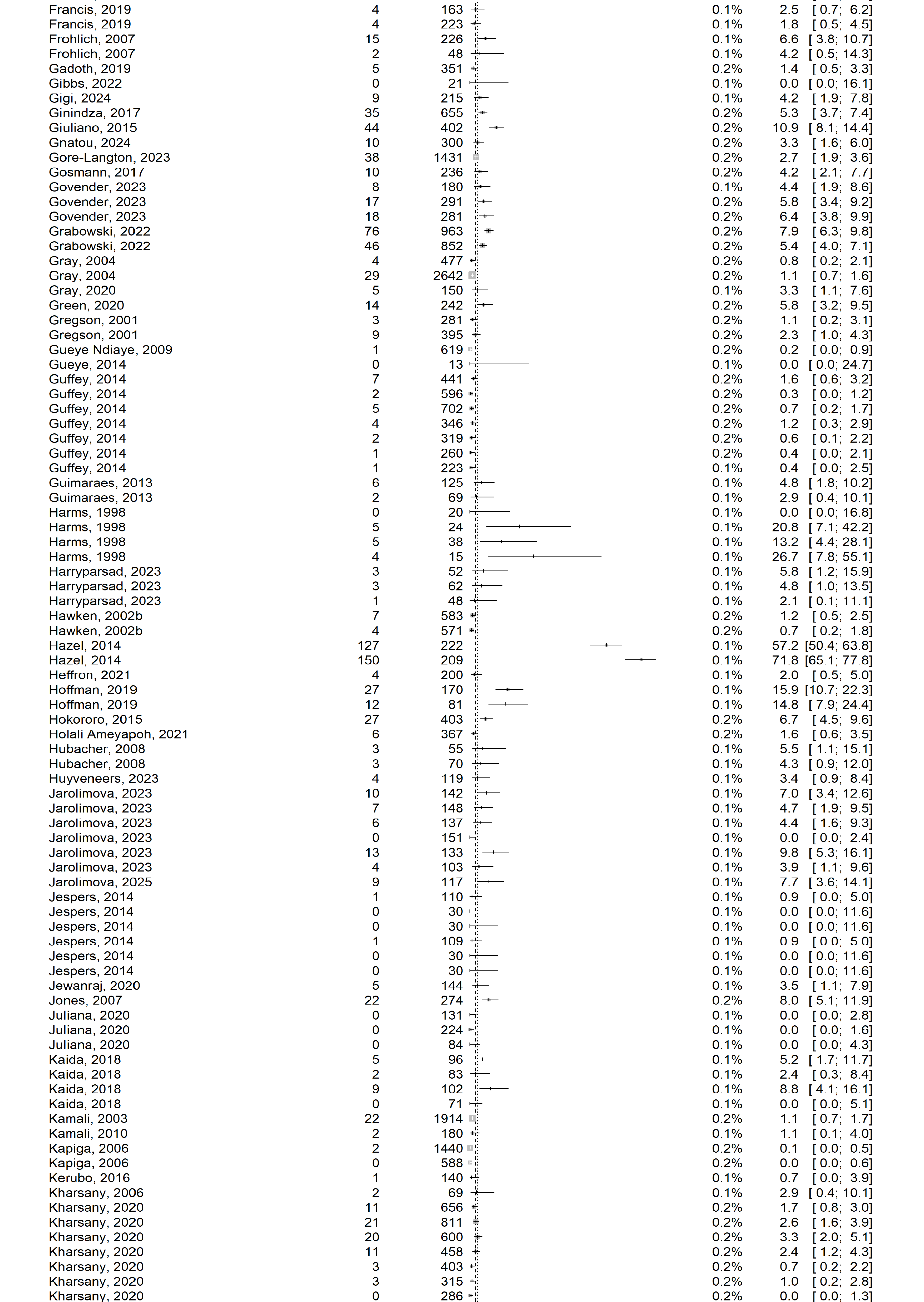
**

**
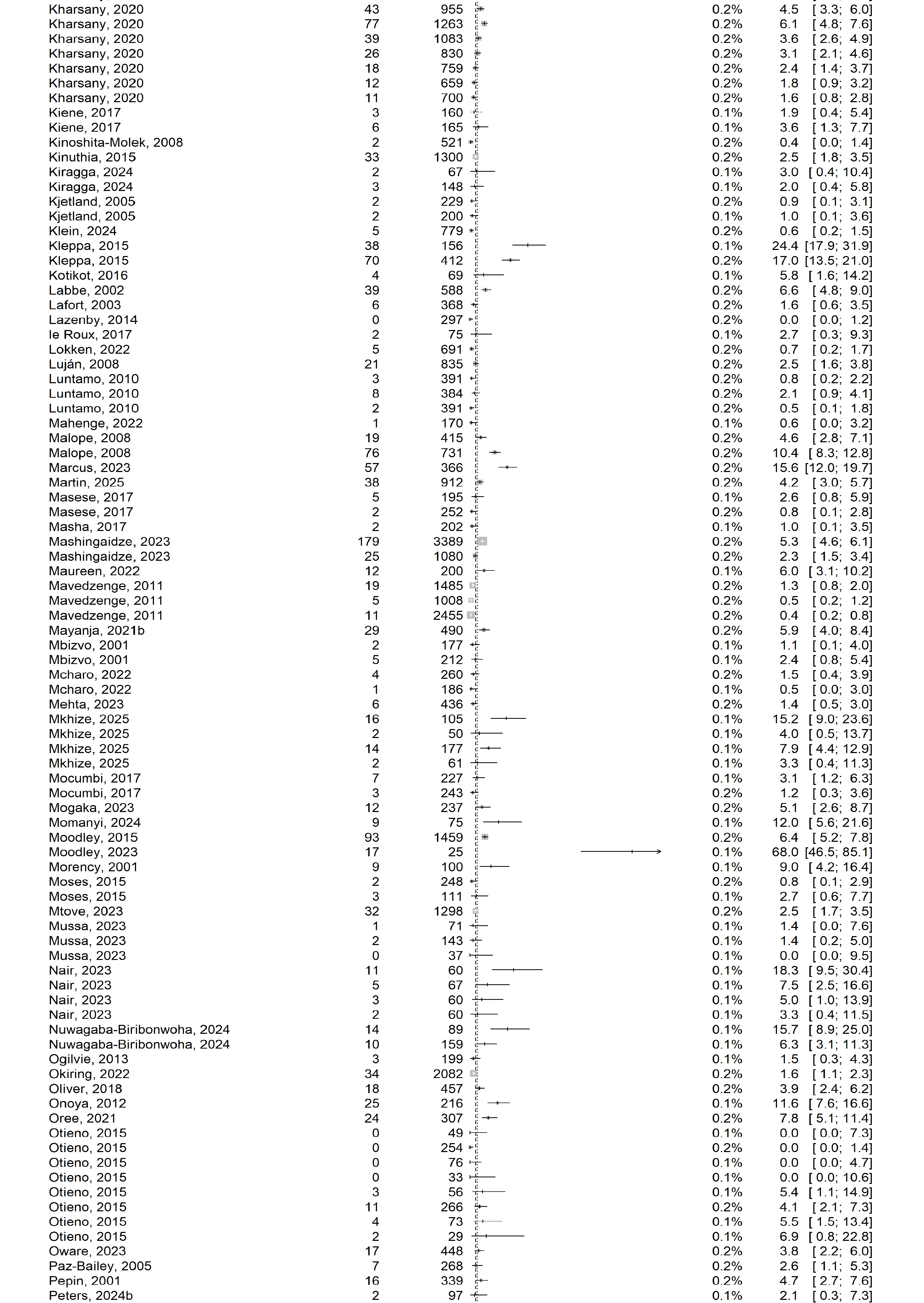
**

**
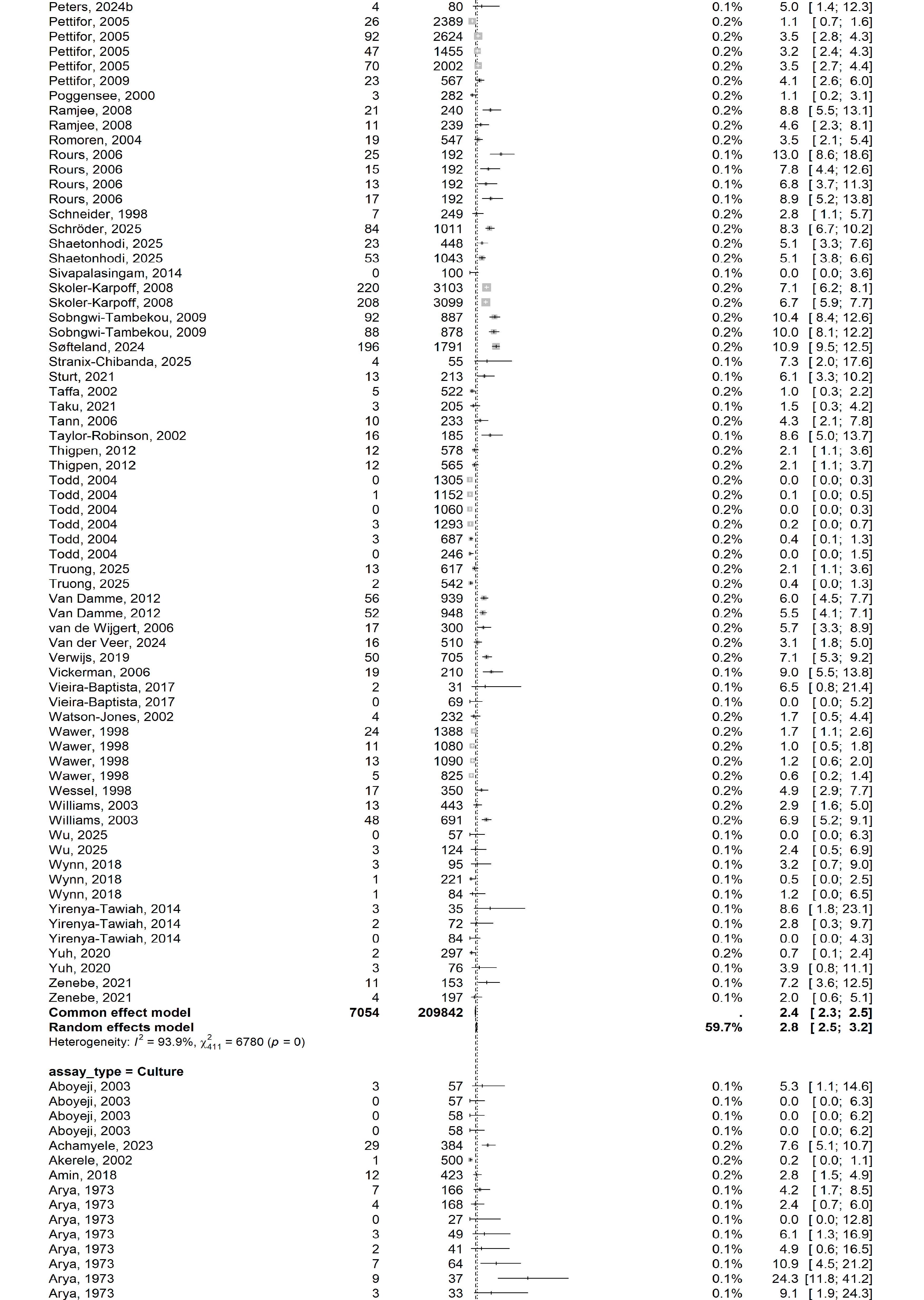
**

**
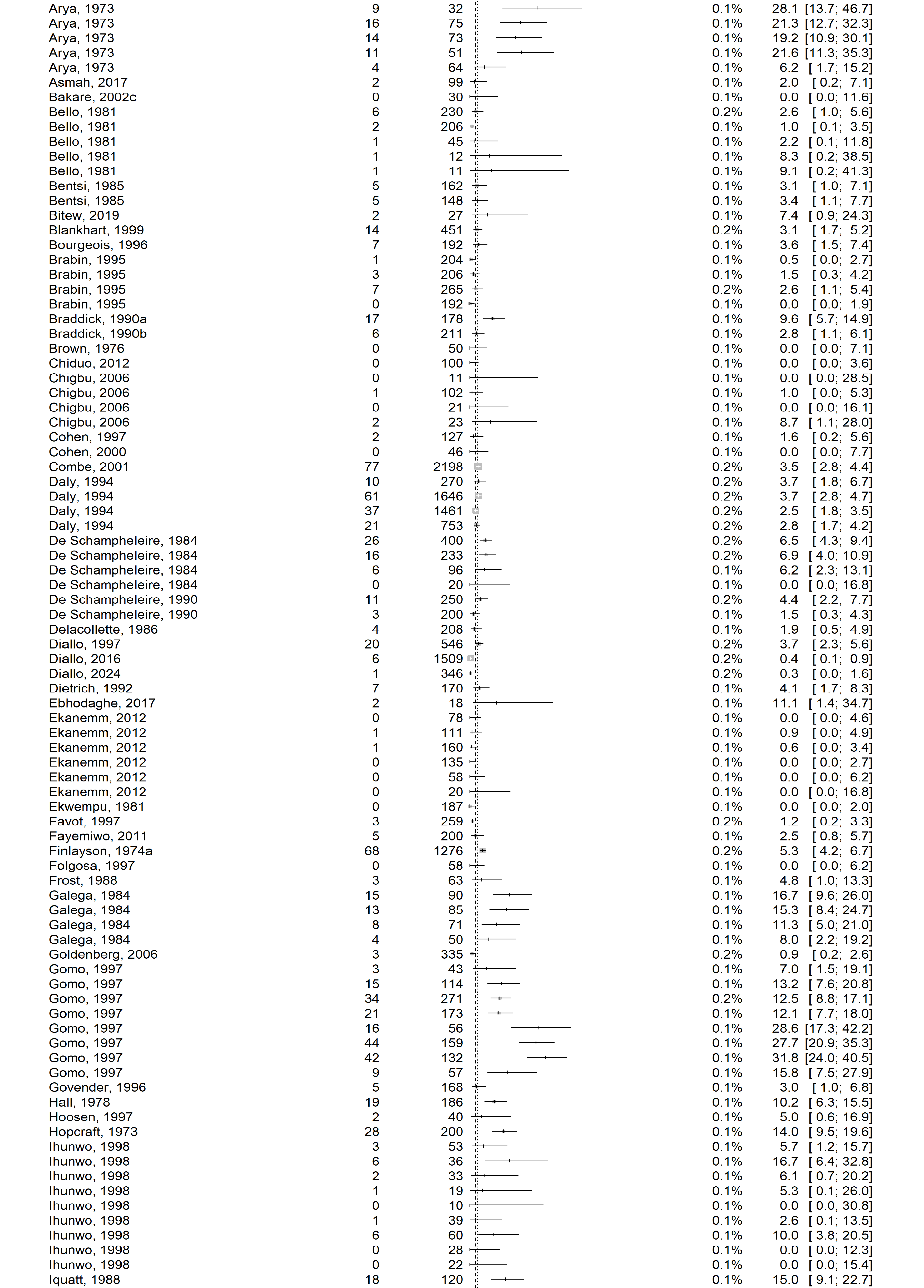
**

**
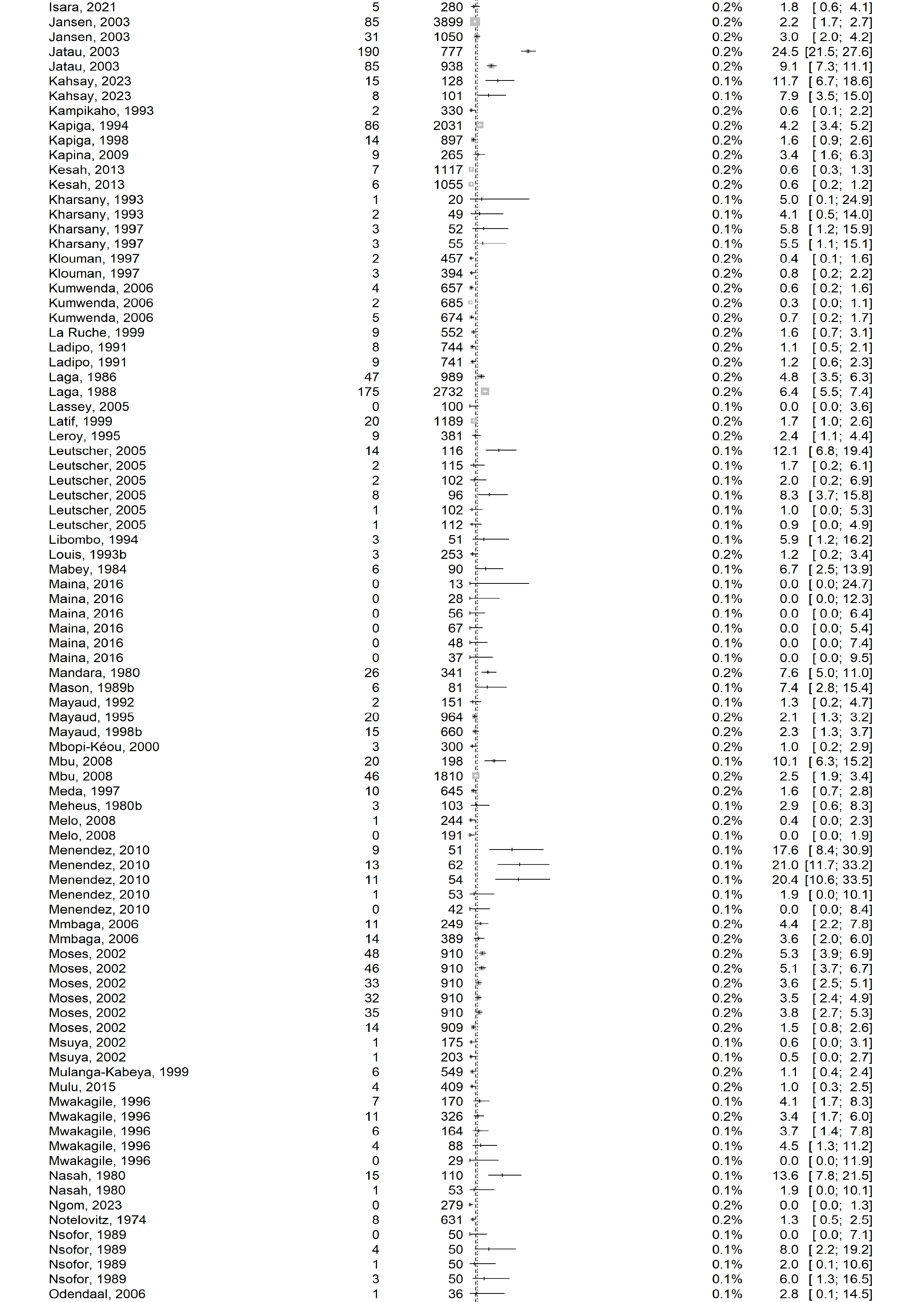
**

**
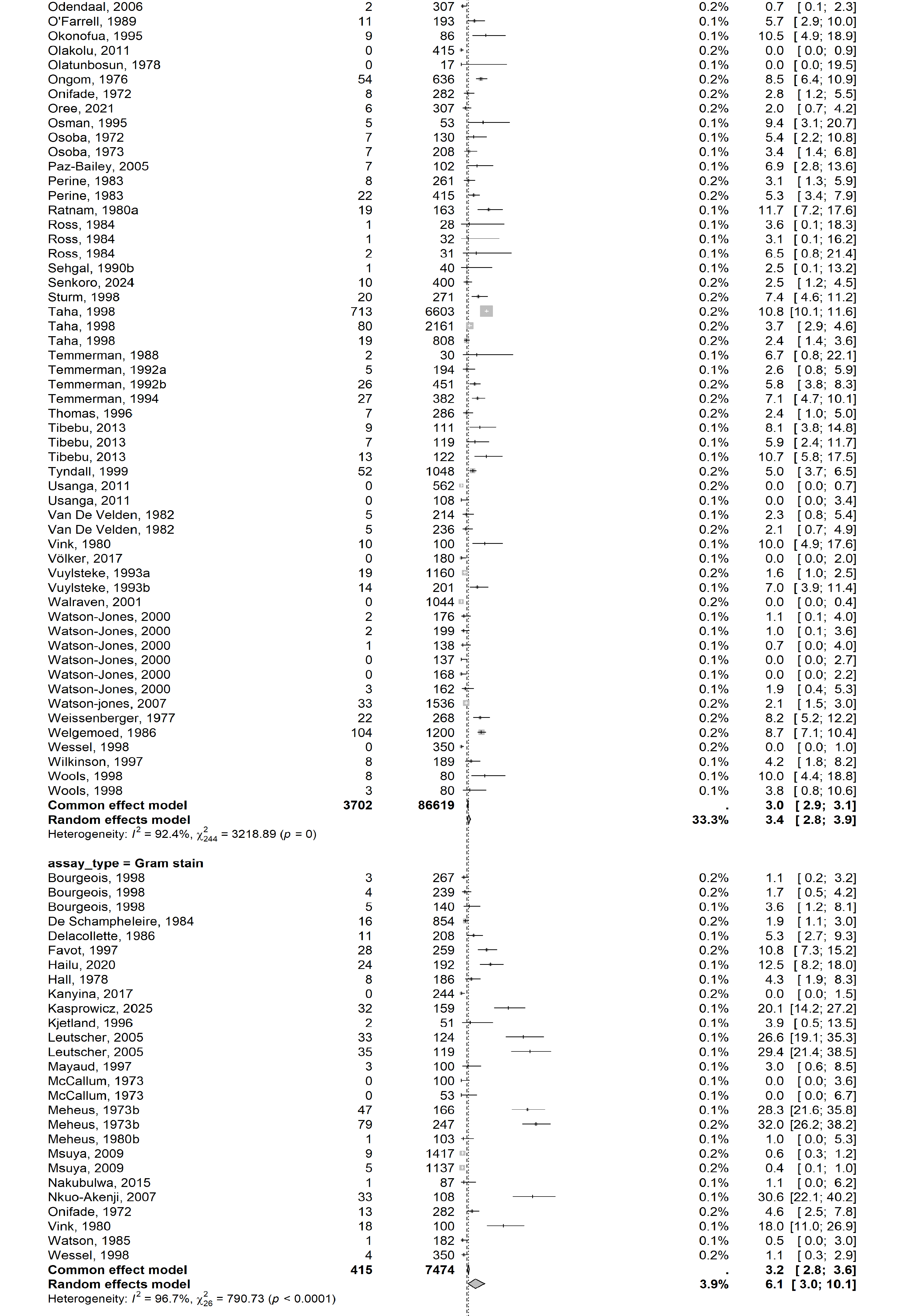
**

**
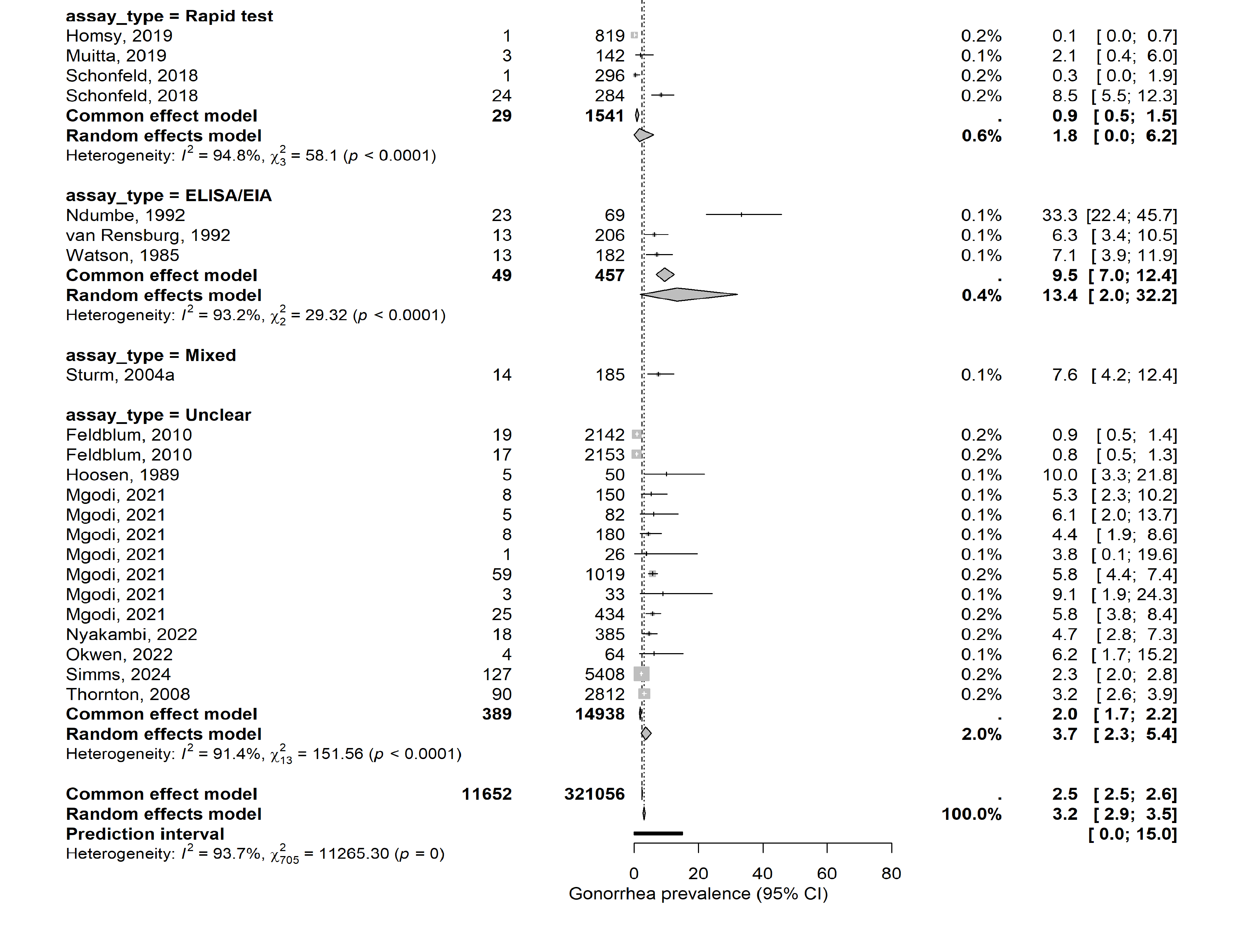
**

Abbreviations: CI, Confidence interval; EIA, Enzyme Immunoassay; ELISA, Enzyme-linked immunosorbent assay; NAAT, Nucleic acid amplification test; PCR, Polymerase chain reaction.

1. Intermediate-risk populations

**
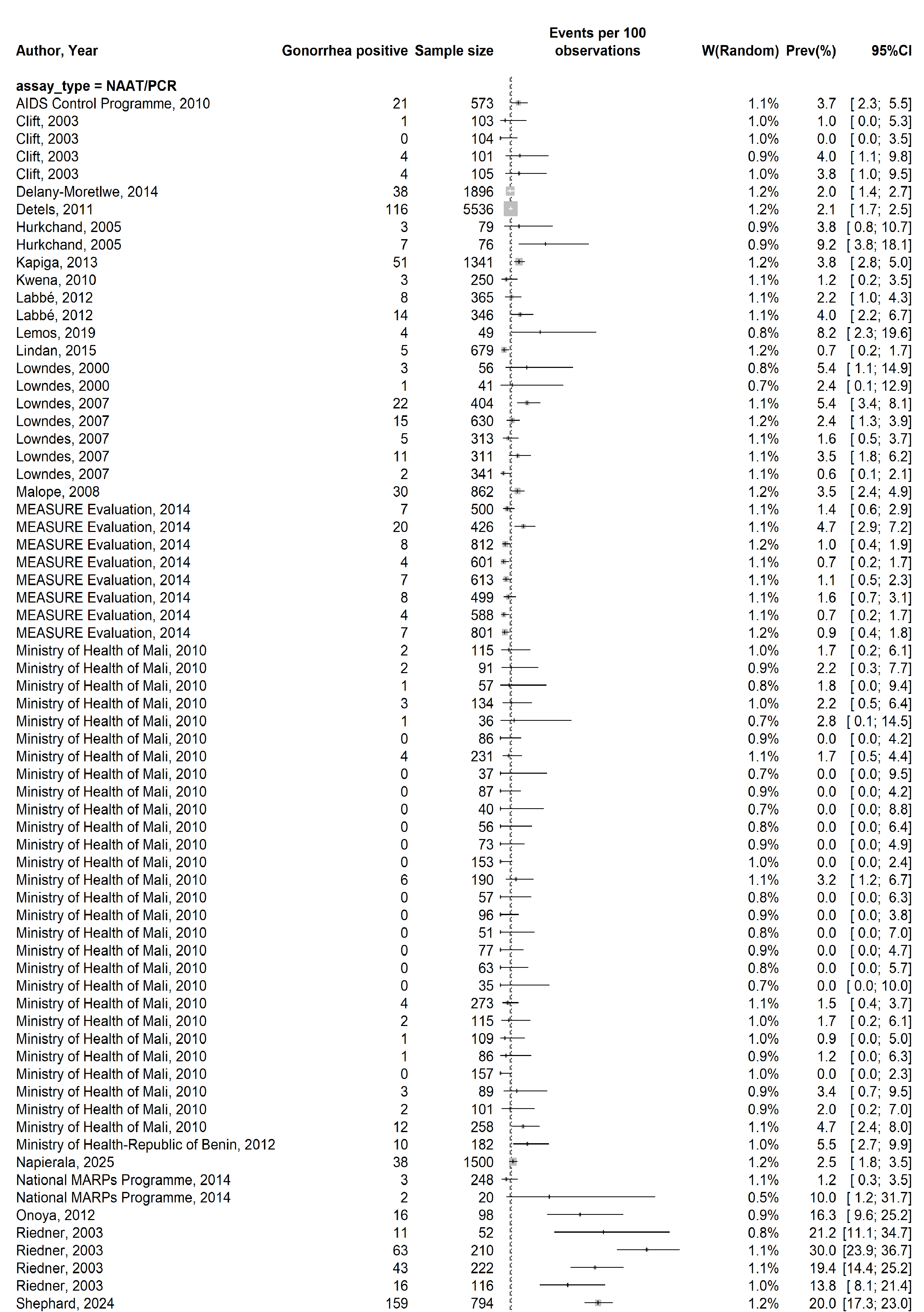
**

**
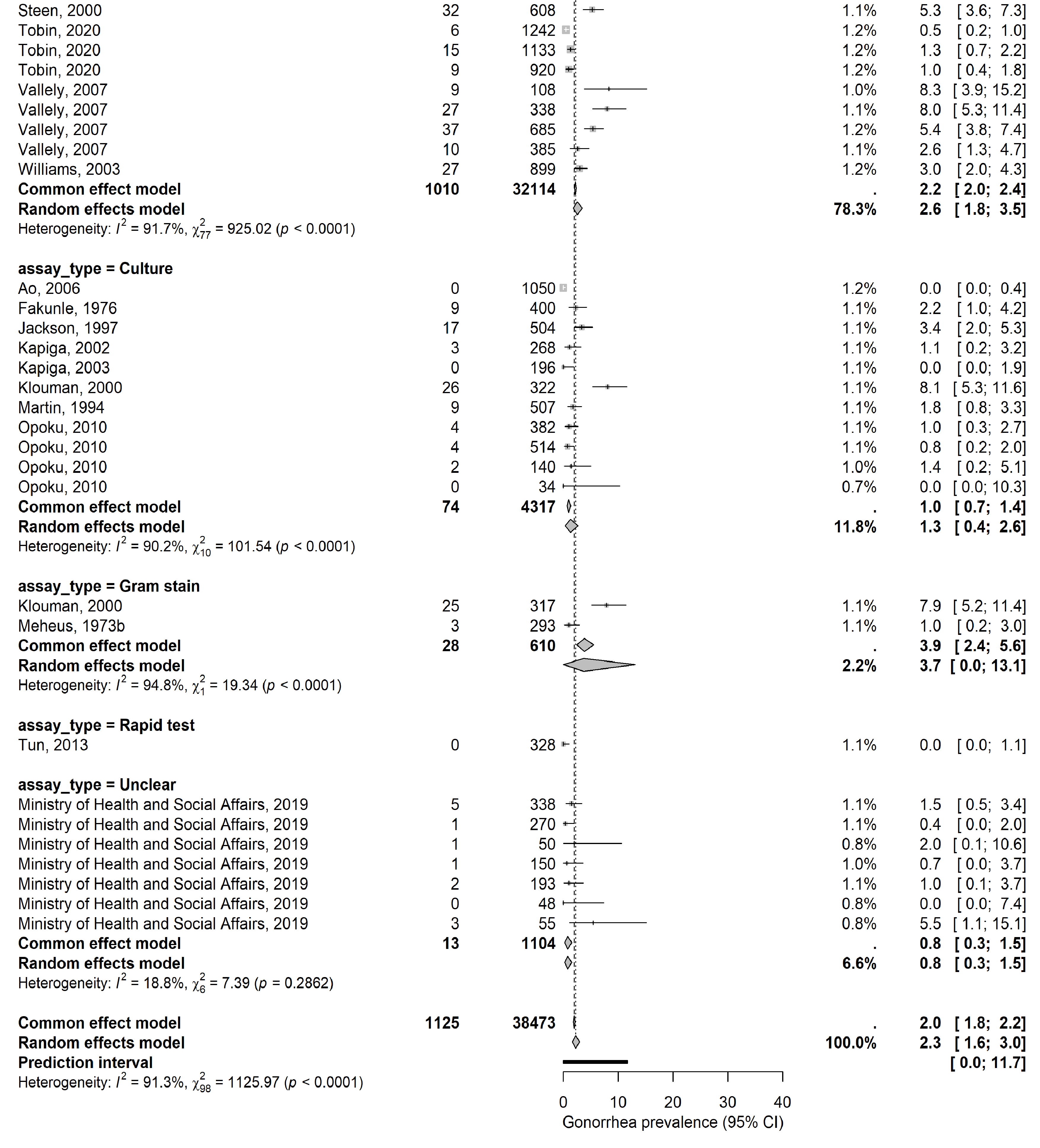
**

Abbreviations: CI, Confidence interval; NAAT, Nucleic acid amplification test; PCR, Polymerase chain reaction.

1. Female sex workers


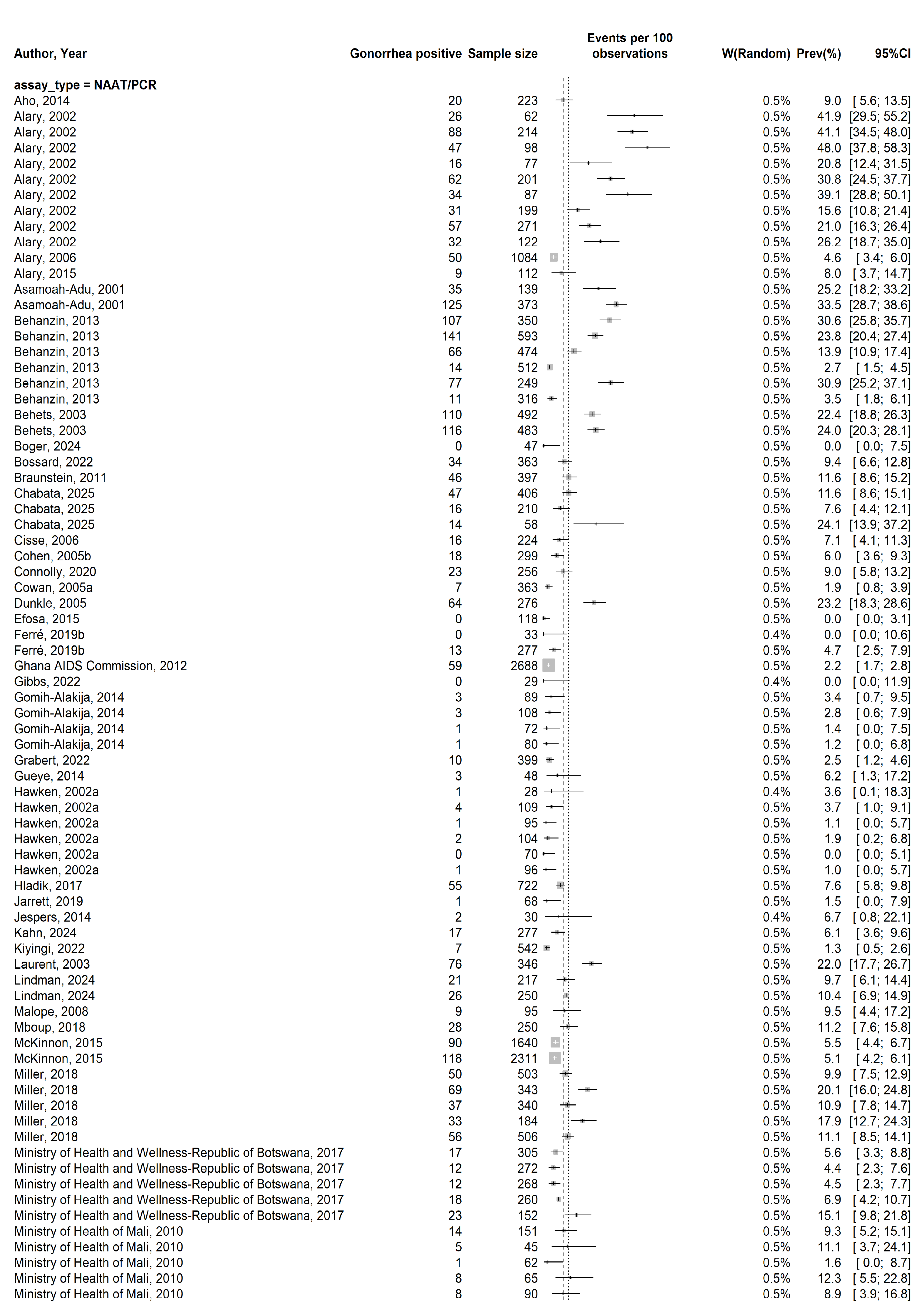


**
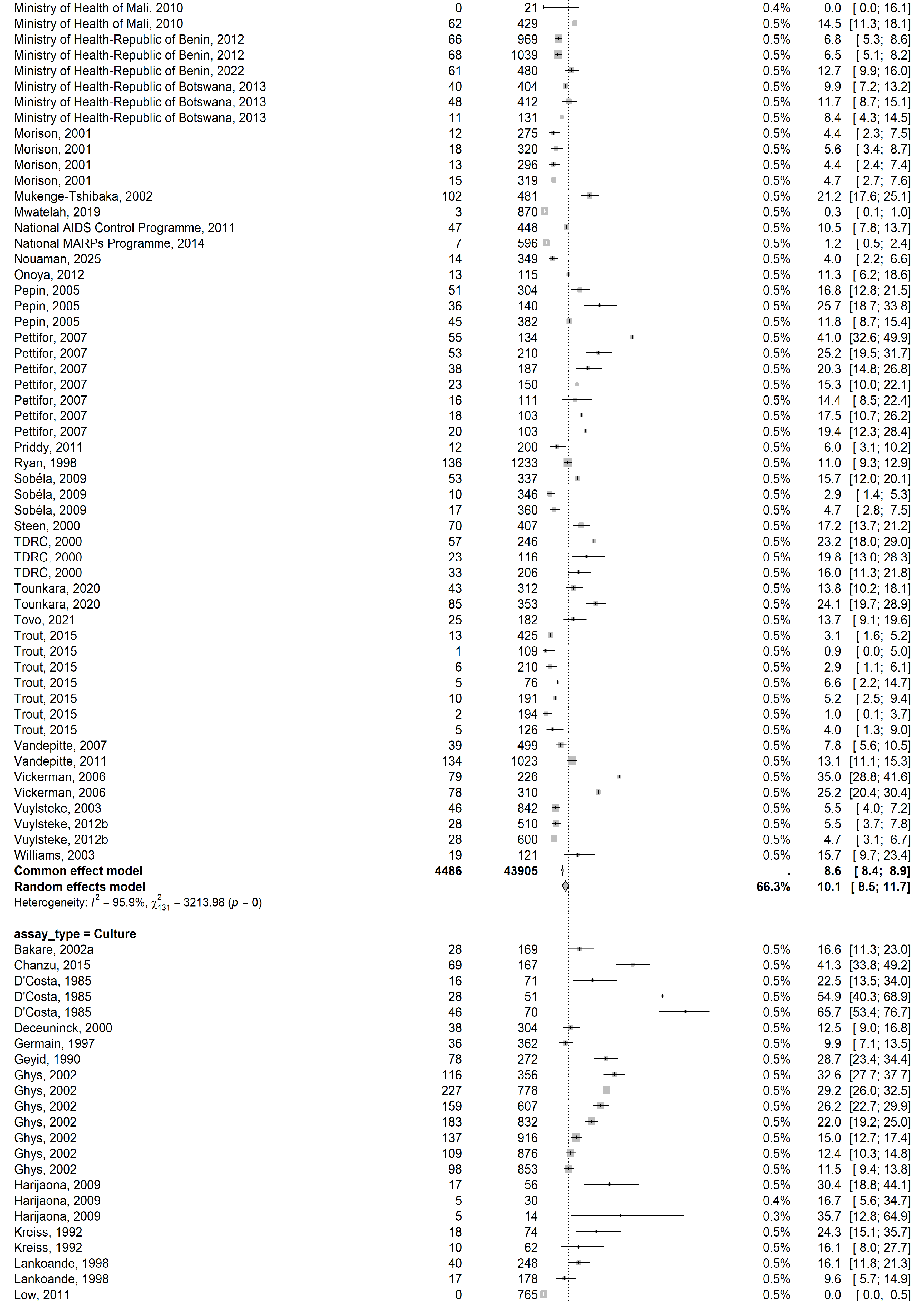
**

**
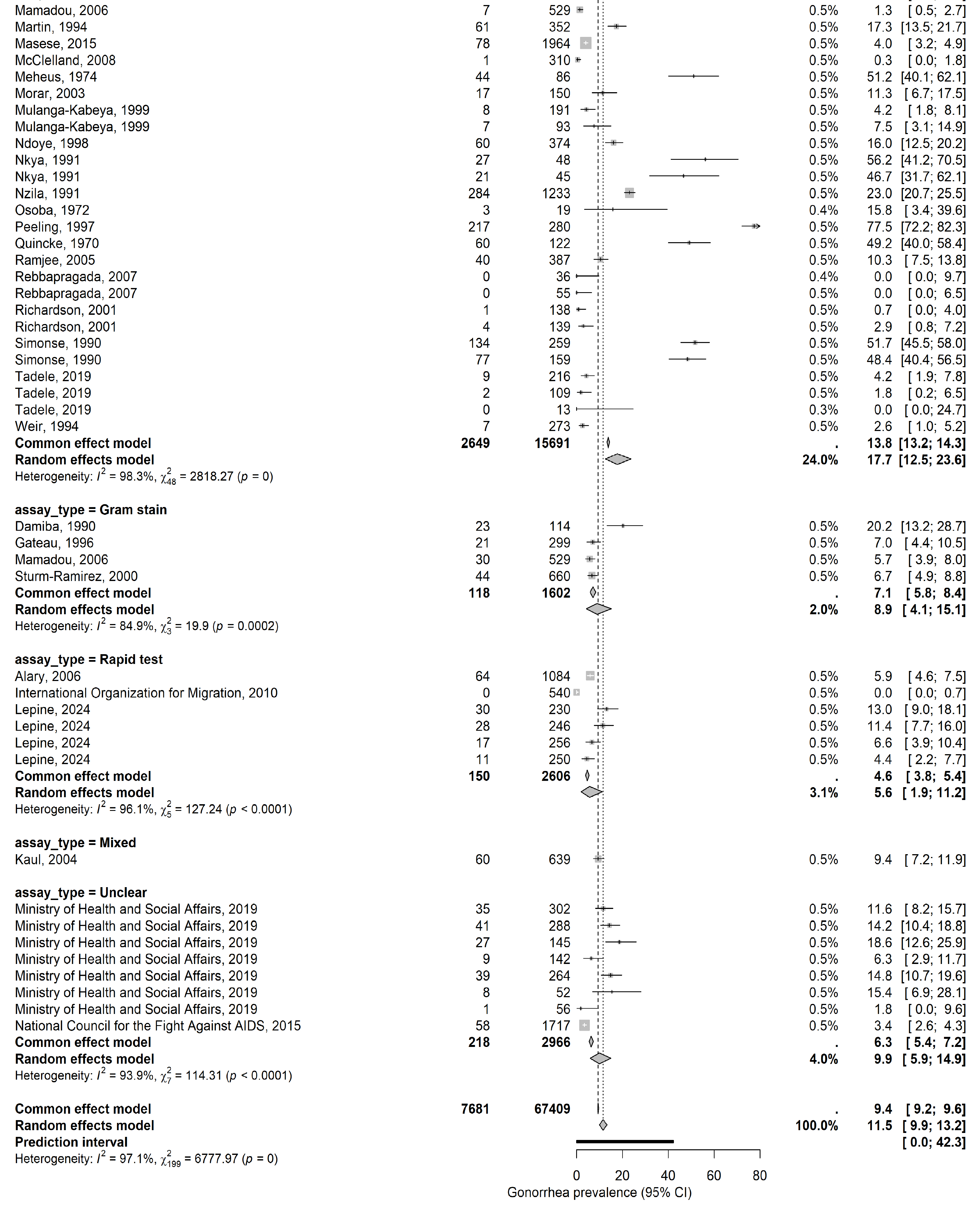
**

Abbreviations: CI, Confidence interval; NAAT, Nucleic acid amplification test; PCR, Polymerase chain reaction.

1. Men who have sex with men^*^

**
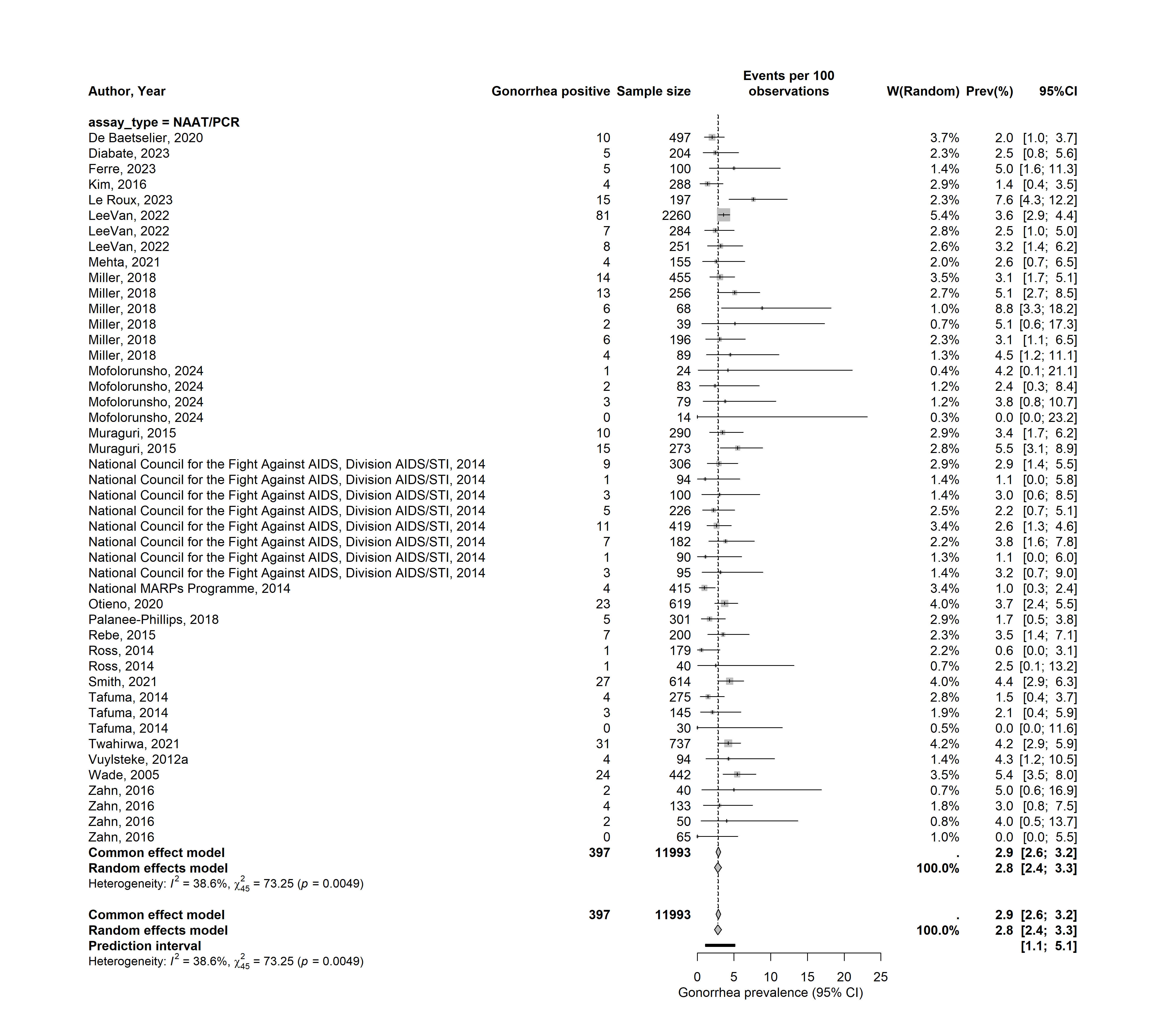
**

Abbreviations: CI, Confidence interval; NAAT, Nucleic acid amplification test; PCR, Polymerase chain reaction.

^*^The term “Men who have sex with men” is used inclusively to also include transgender individuals and male or transgender sex workers.

1. Symptomatic women


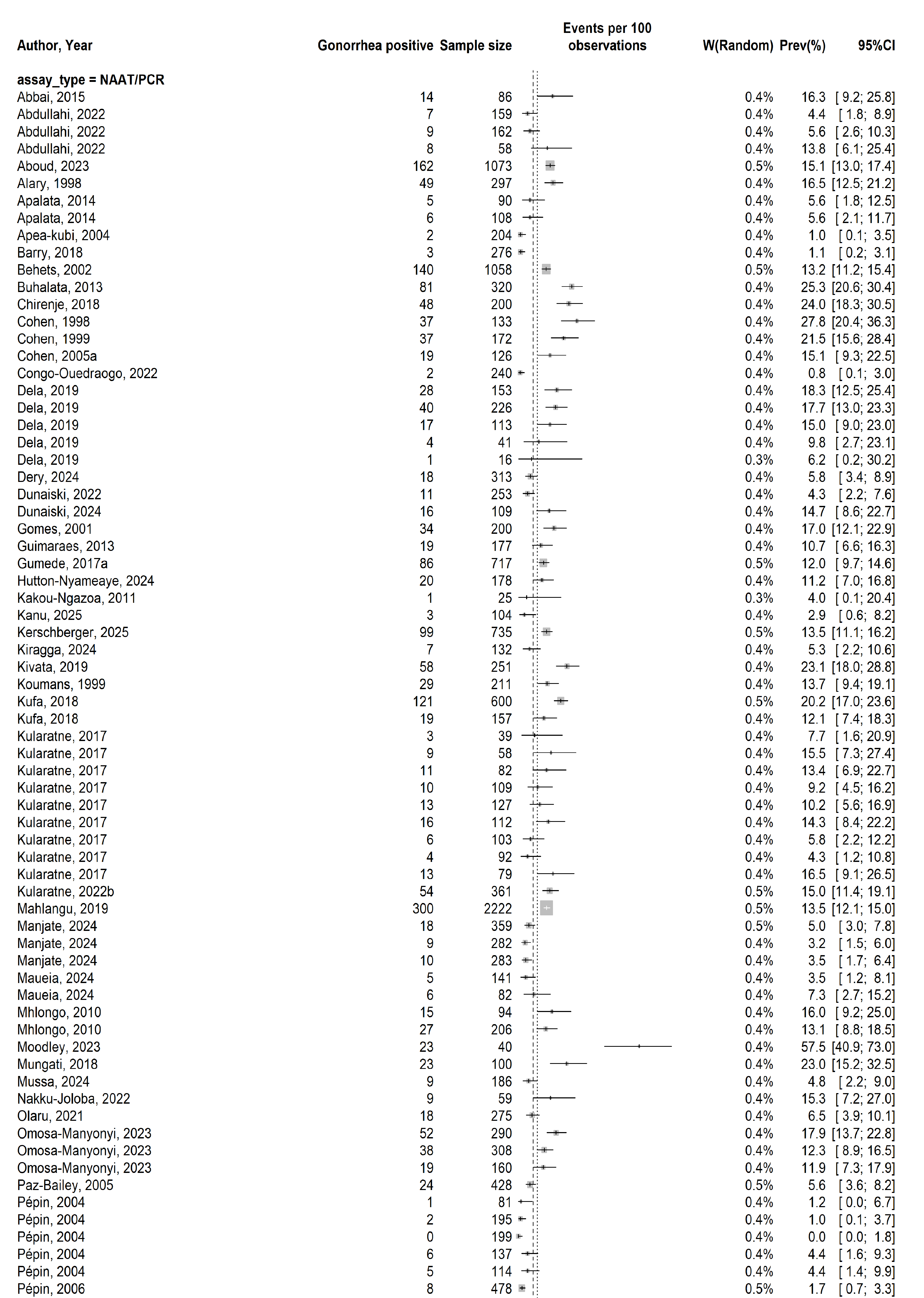


***-
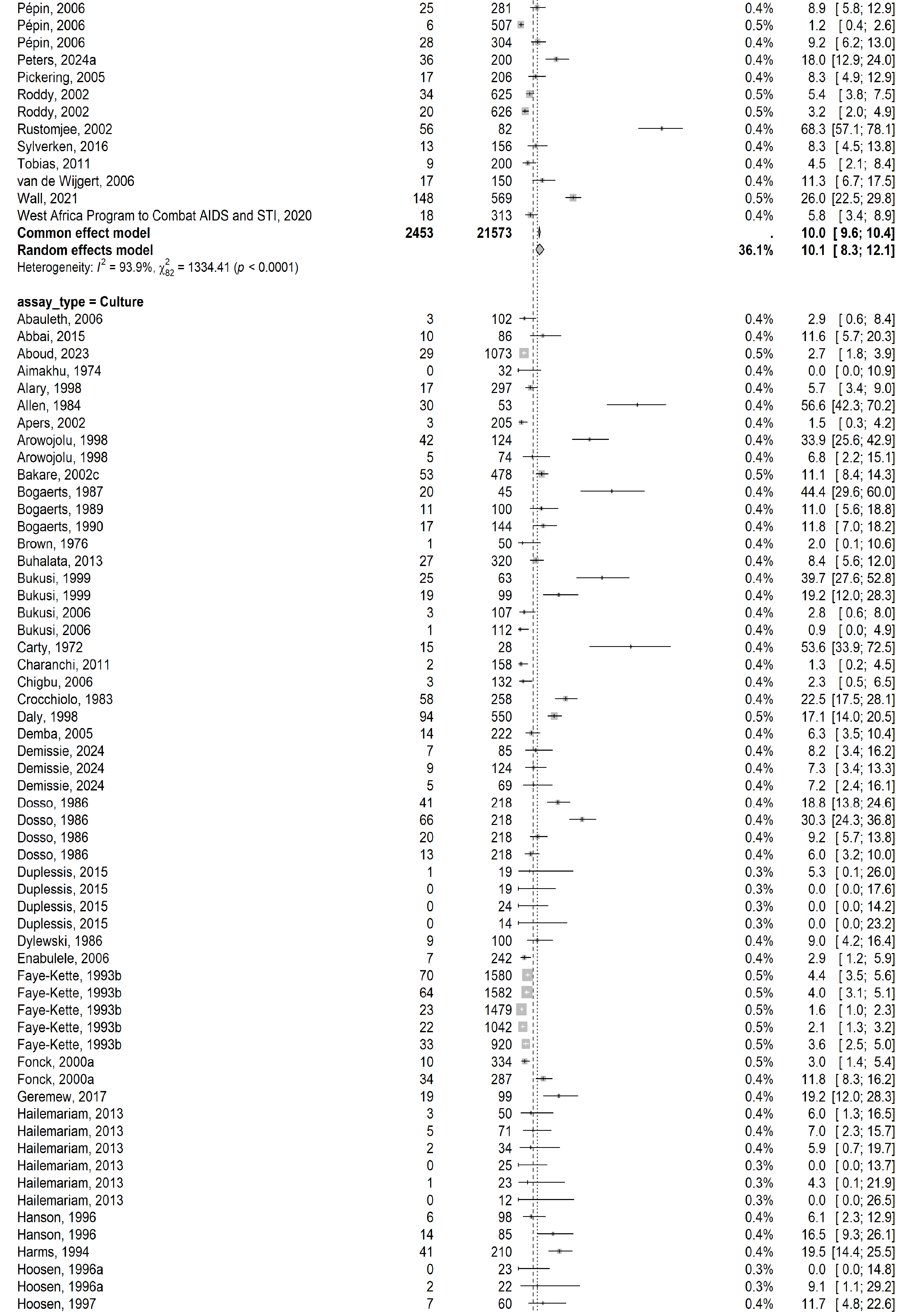
**

**
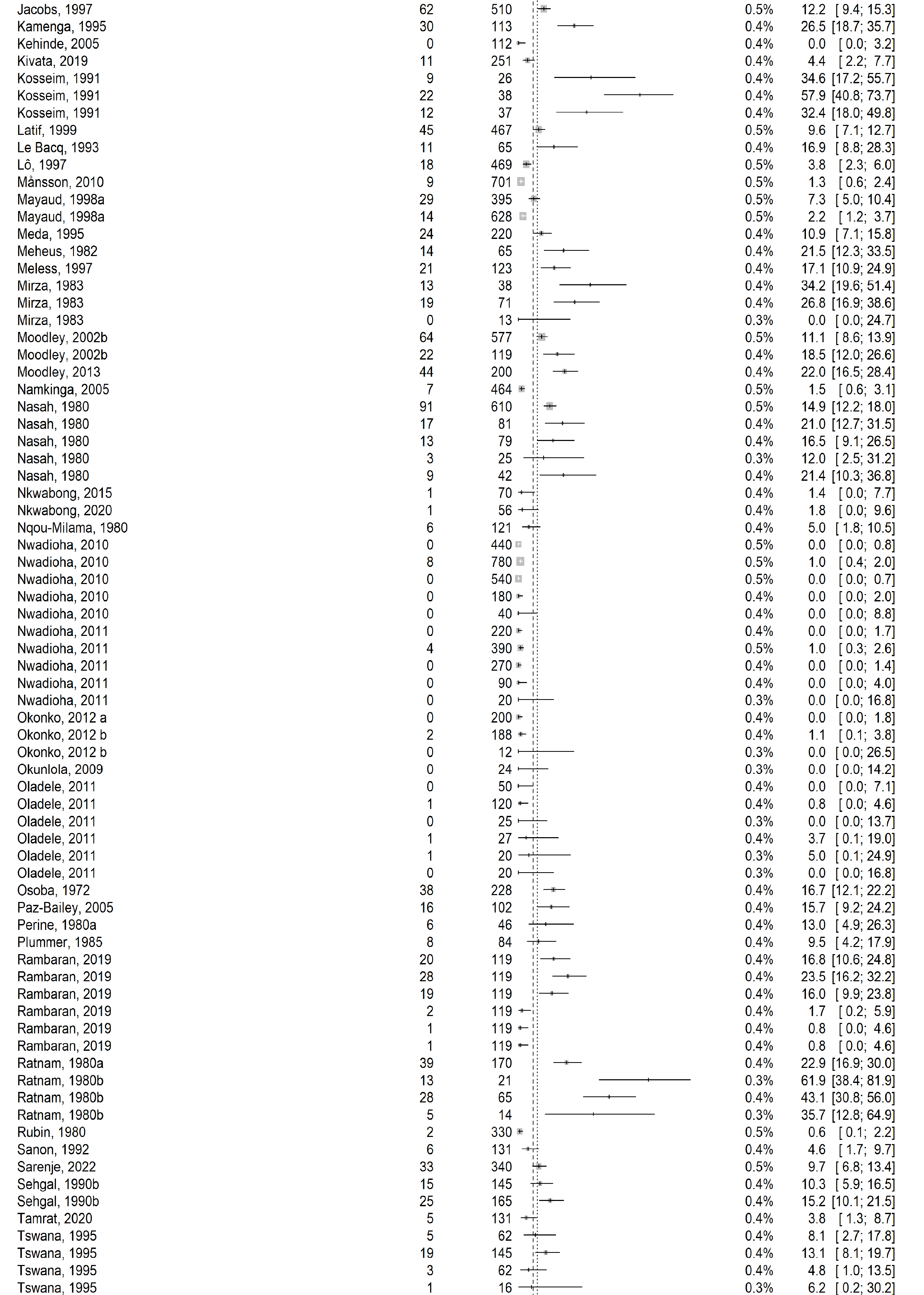
**

**
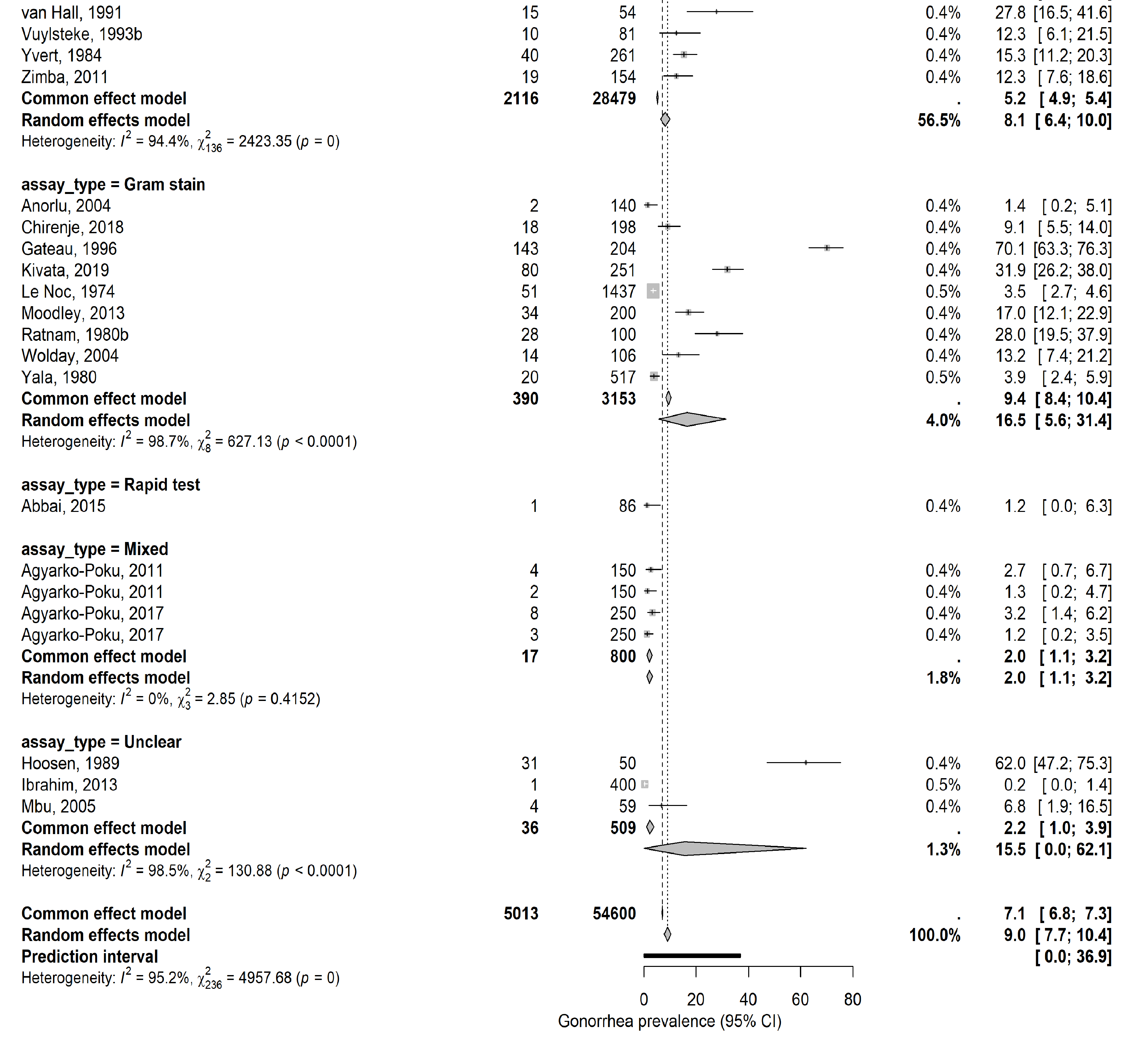
**

Abbreviations: CI, Confidence interval; NAAT, Nucleic acid amplification test; PCR, Polymerase chain reaction.

1. Symptomatic men


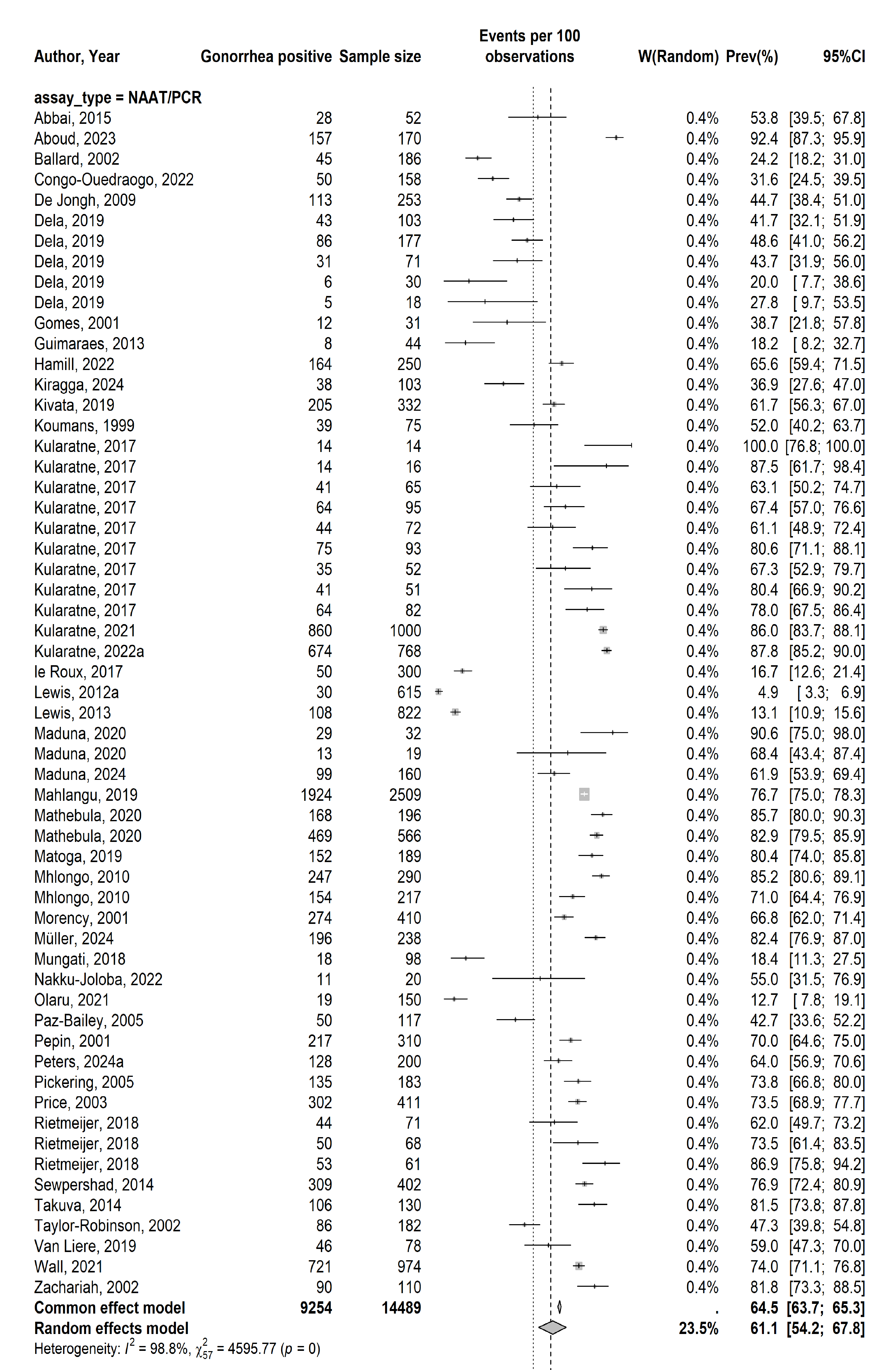


**
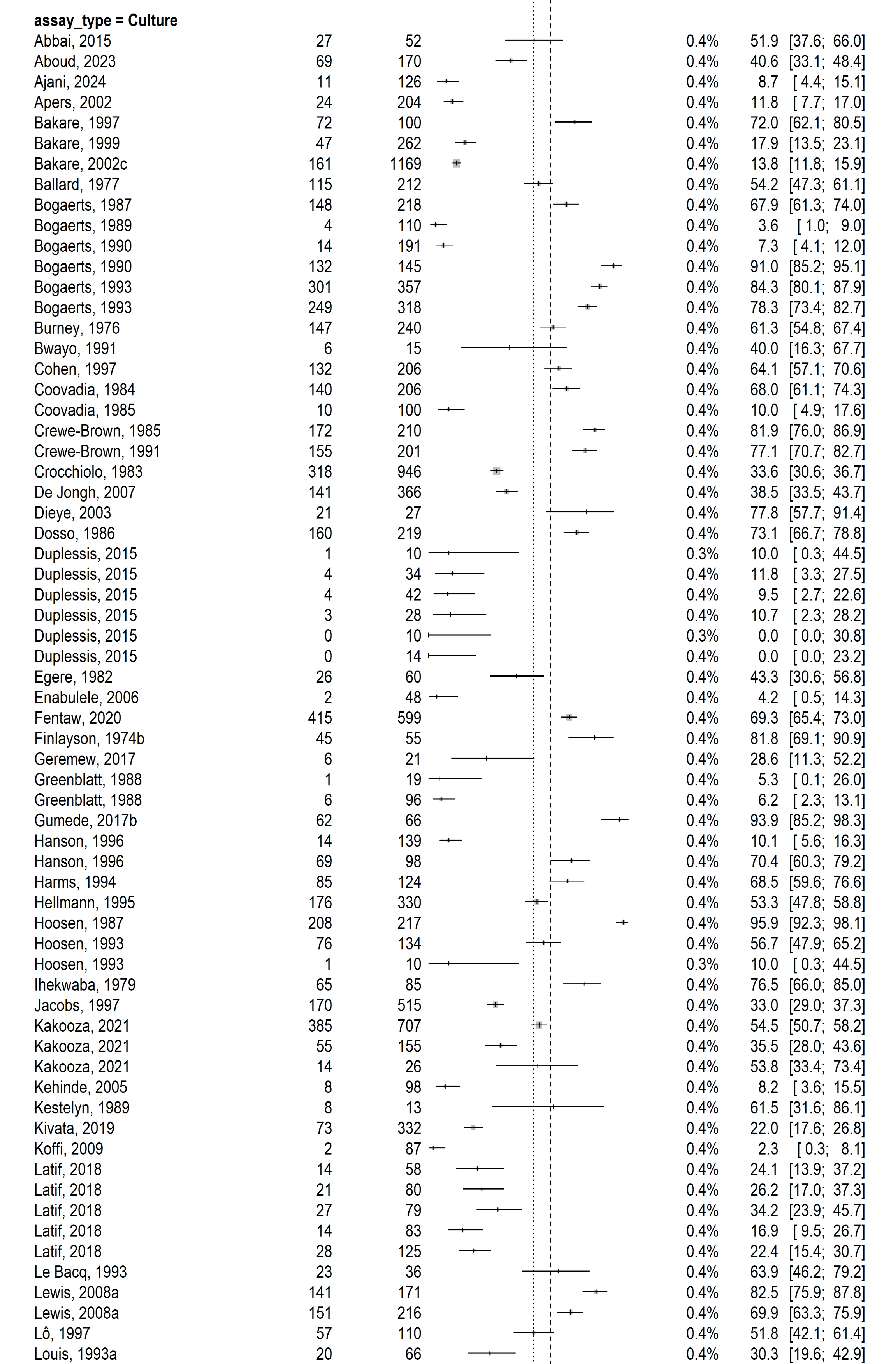
**

**
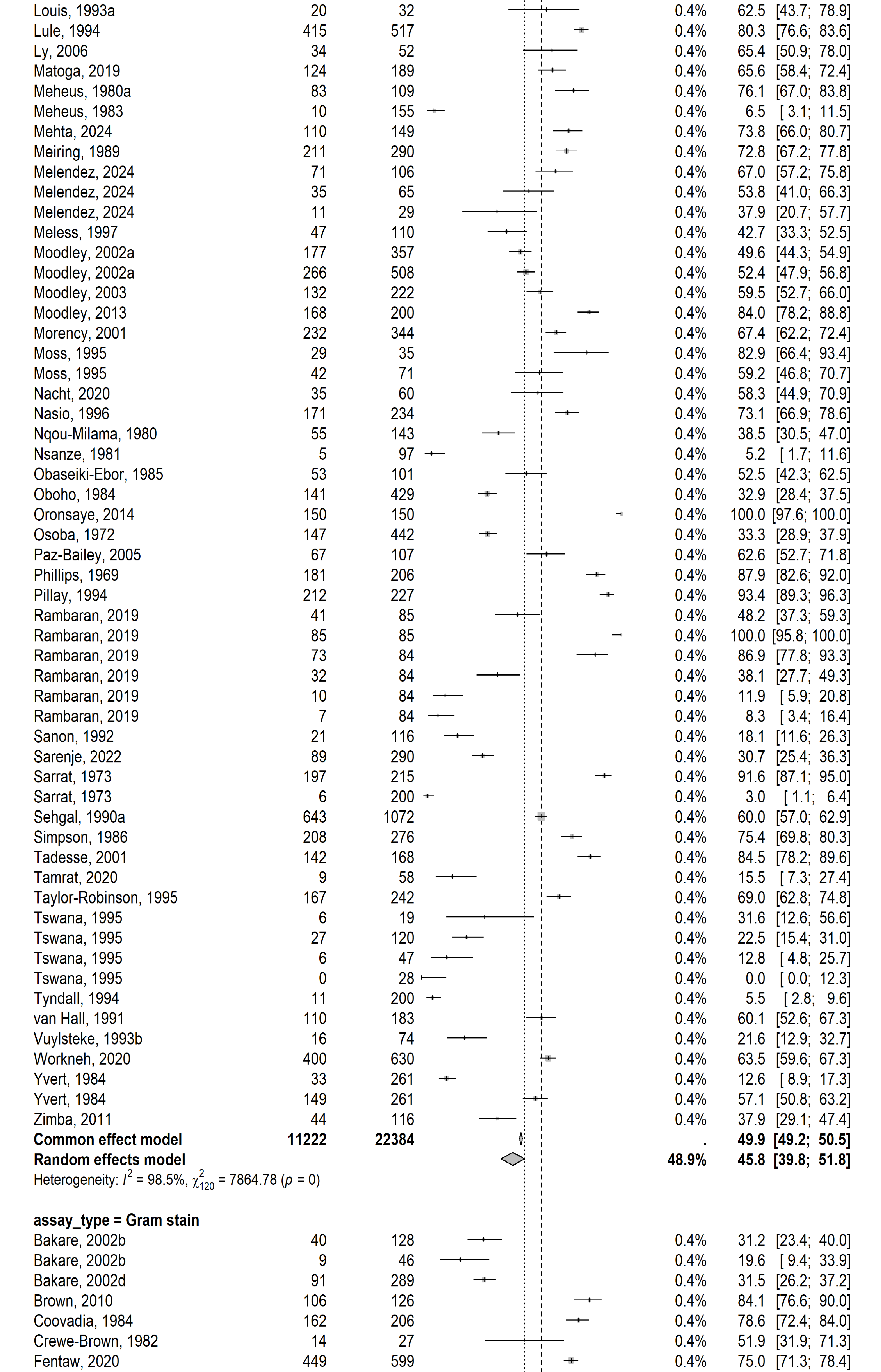
**

**
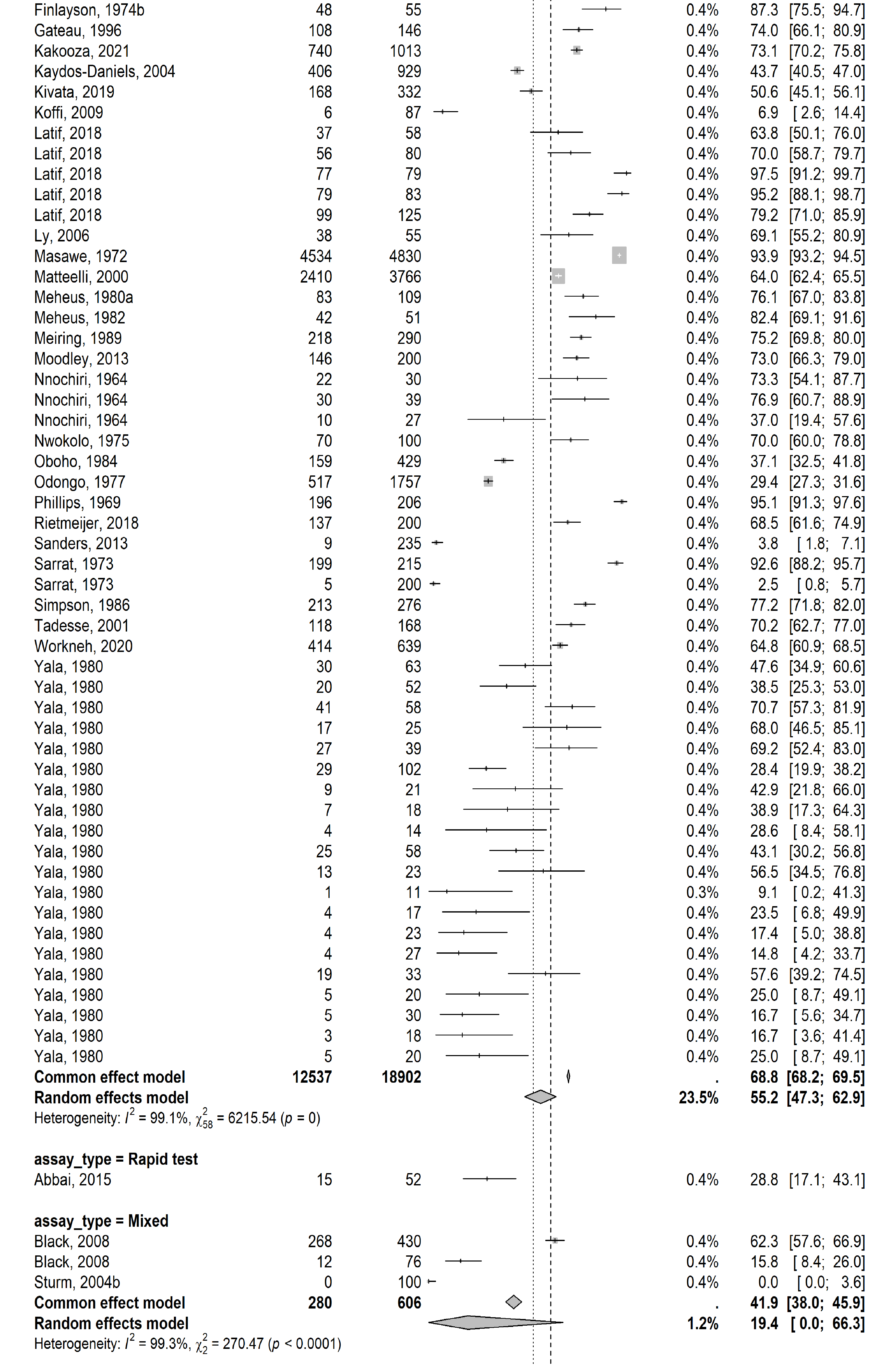
**

**
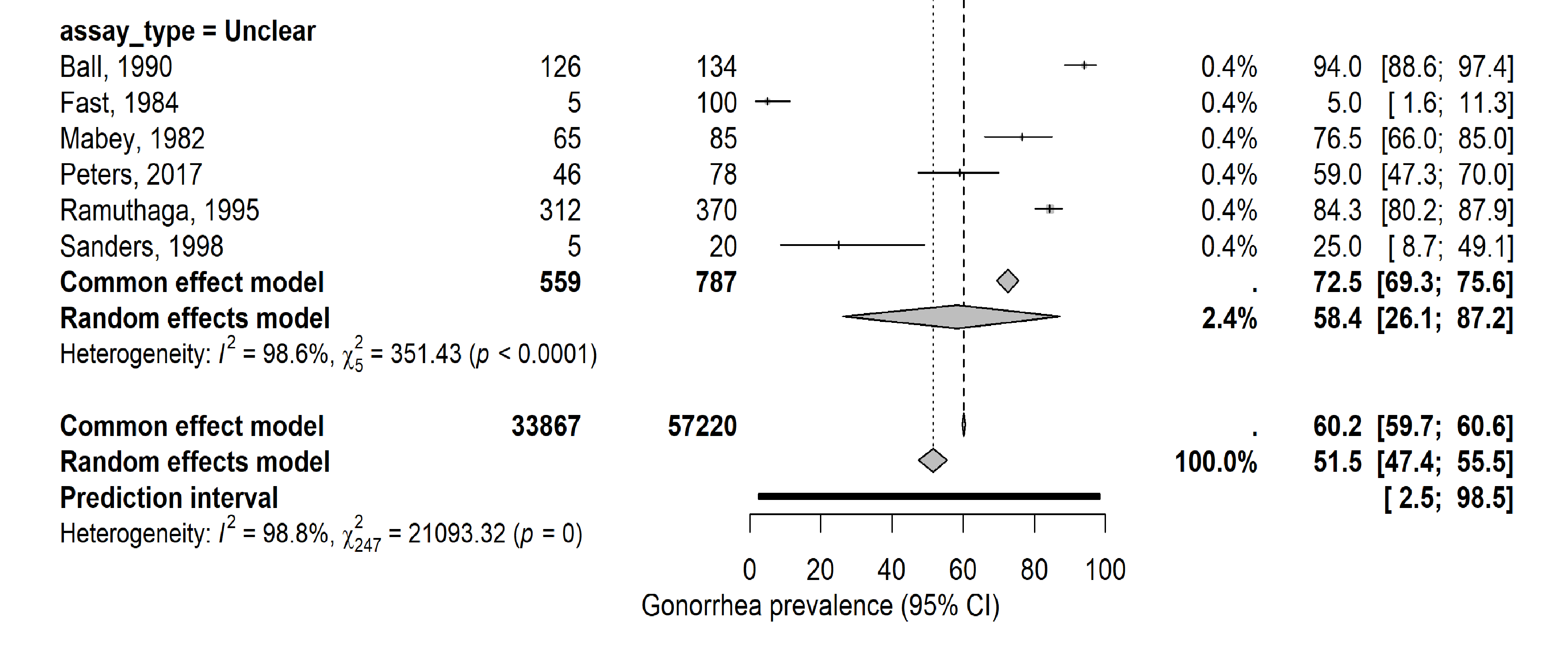
**

Abbreviations: CI, Confidence interval; NAAT, Nucleic acid amplification test; PCR, Polymerase chain reaction.

1. Symptomatic women and men


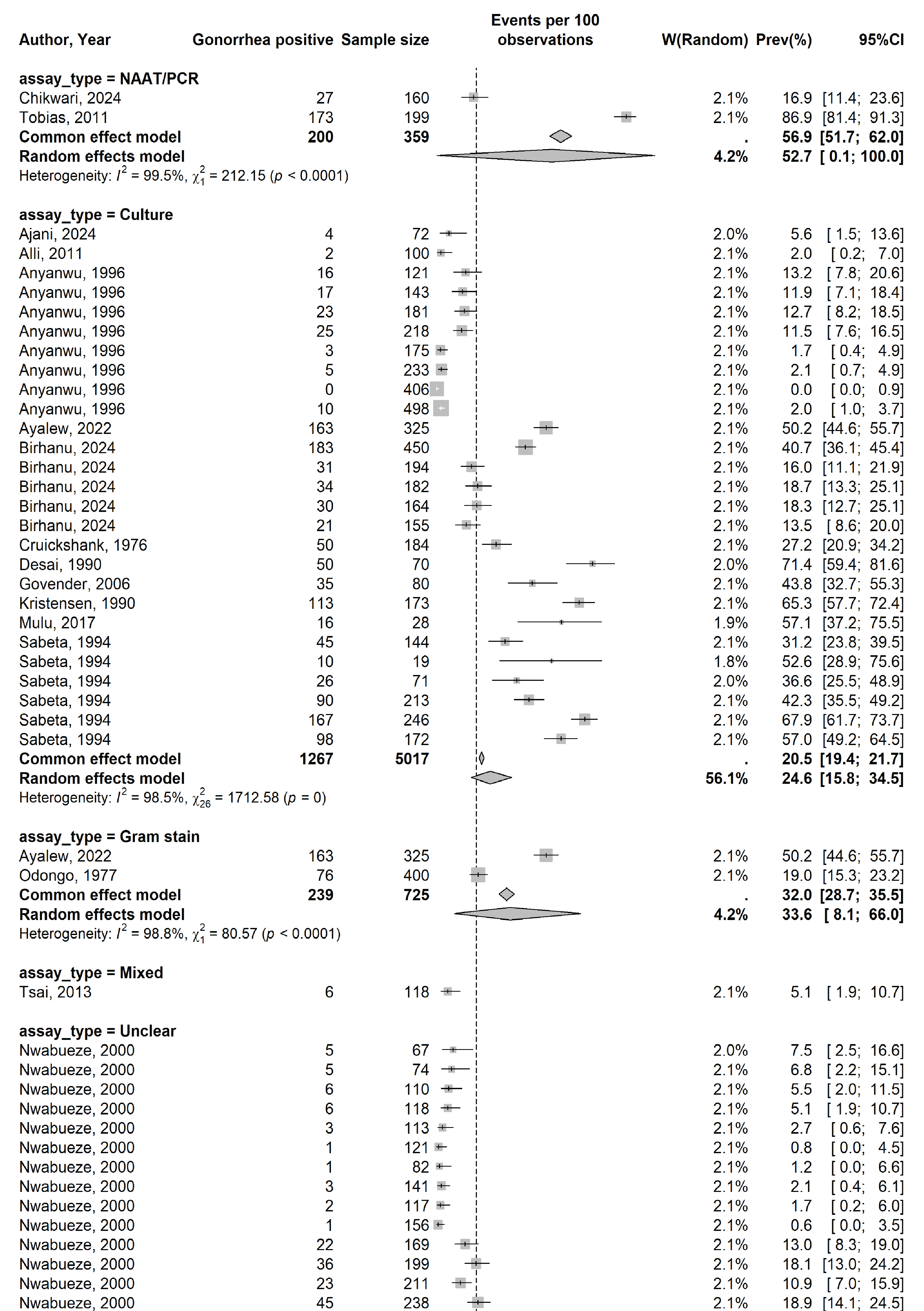


**
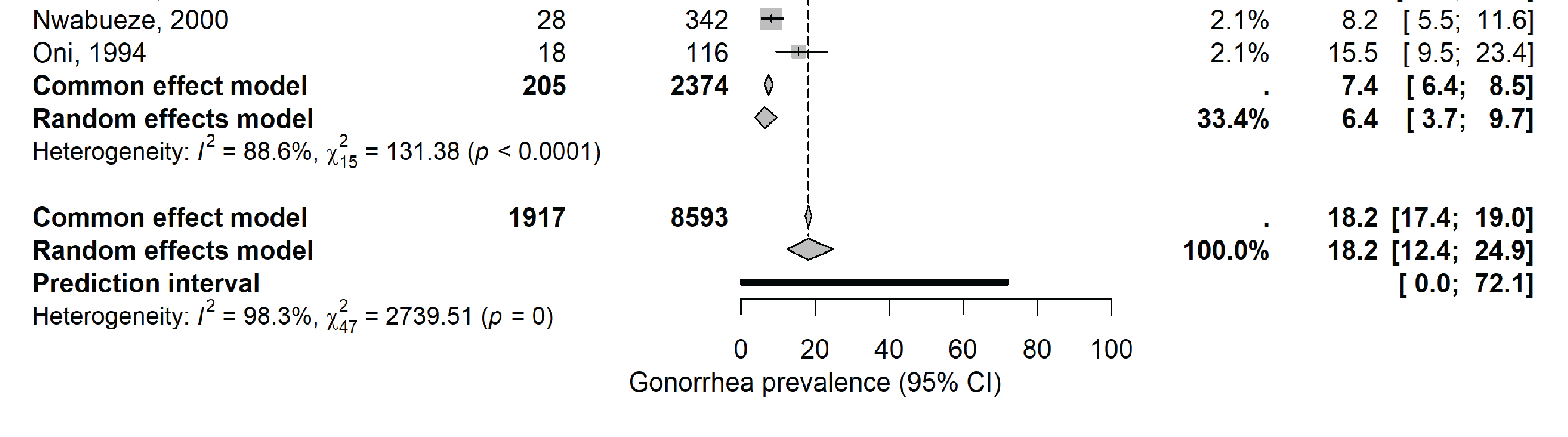
**

Abbreviations: CI, Confidence interval; NAAT, Nucleic acid amplification test; PCR, Polymerase chain reaction.

1. Infertility clinic attendees


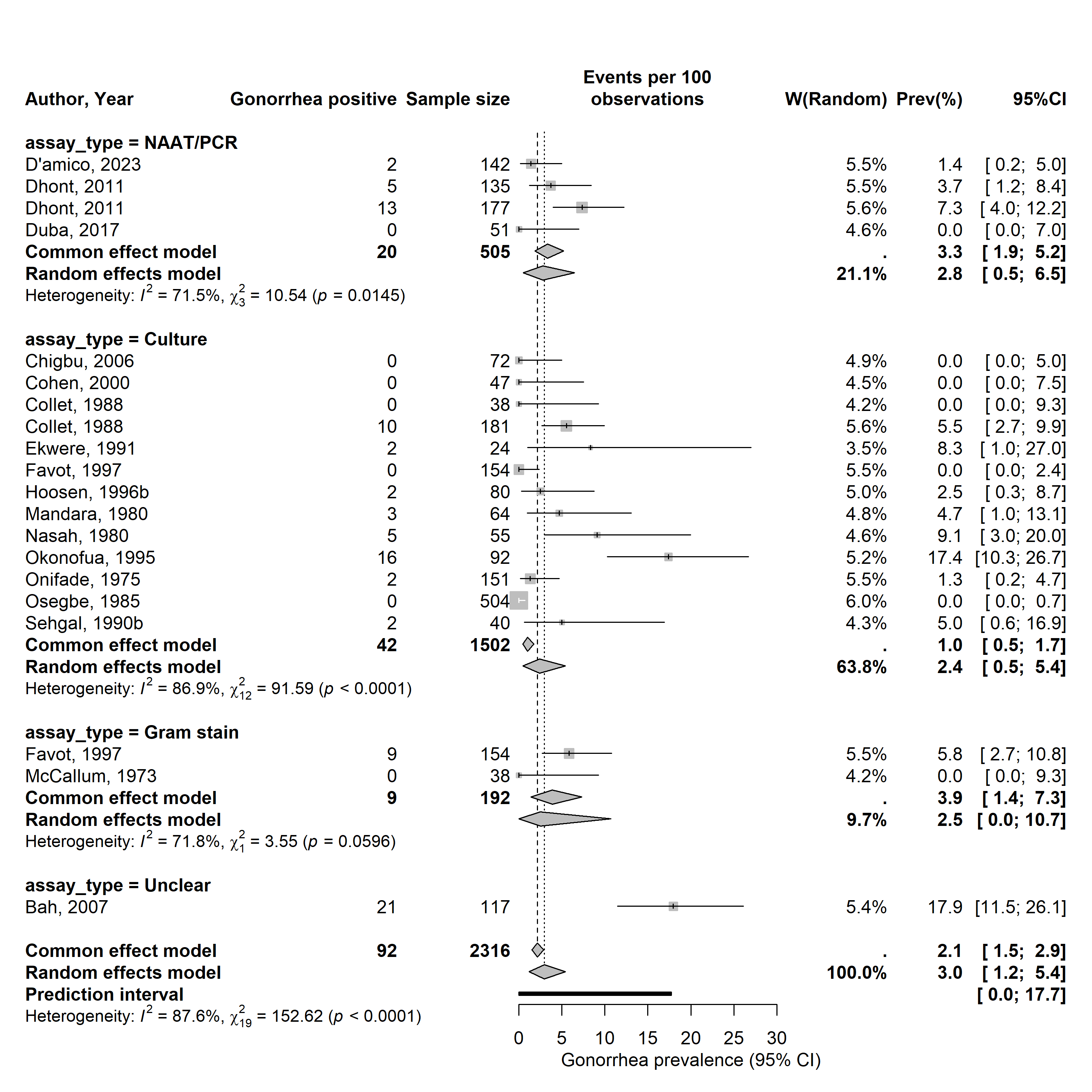


Abbreviations: CI, Confidence interval; NAAT, Nucleic acid amplification test; PCR, Polymerase chain reaction.

1. Women with adverse pregnancy and birth outcomes^†^


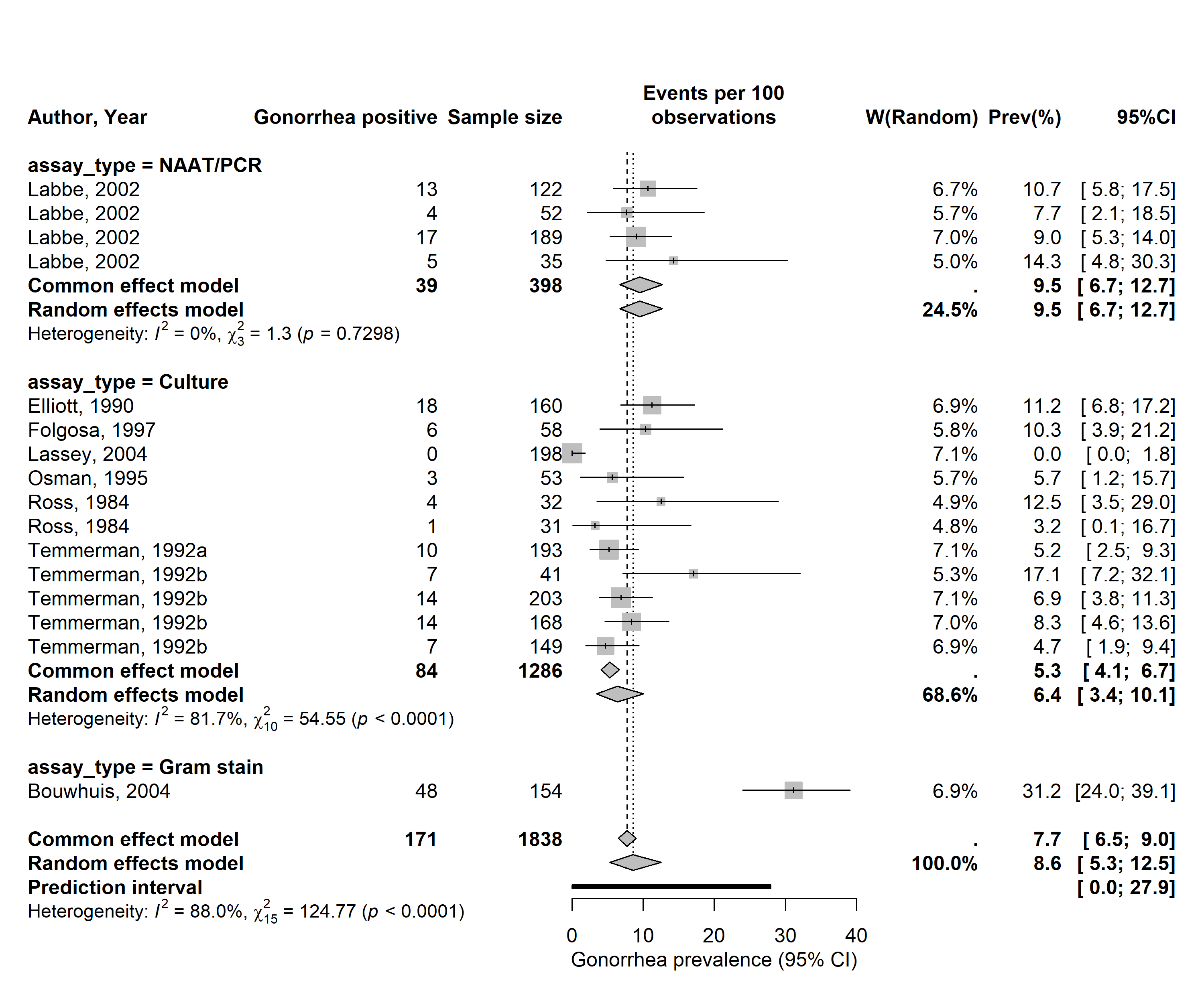


Abbreviations: CI, Confidence interval; NAAT, Nucleic acid amplification test; PCR, Polymerase chain reaction.

^†^Adverse pregnancy or birth outcomes were defined to include miscarriage, ectopic pregnancy, stillbirth, preterm delivery, small-for-gestational-age infants, and related complications.

1. STI clinic attendees

**
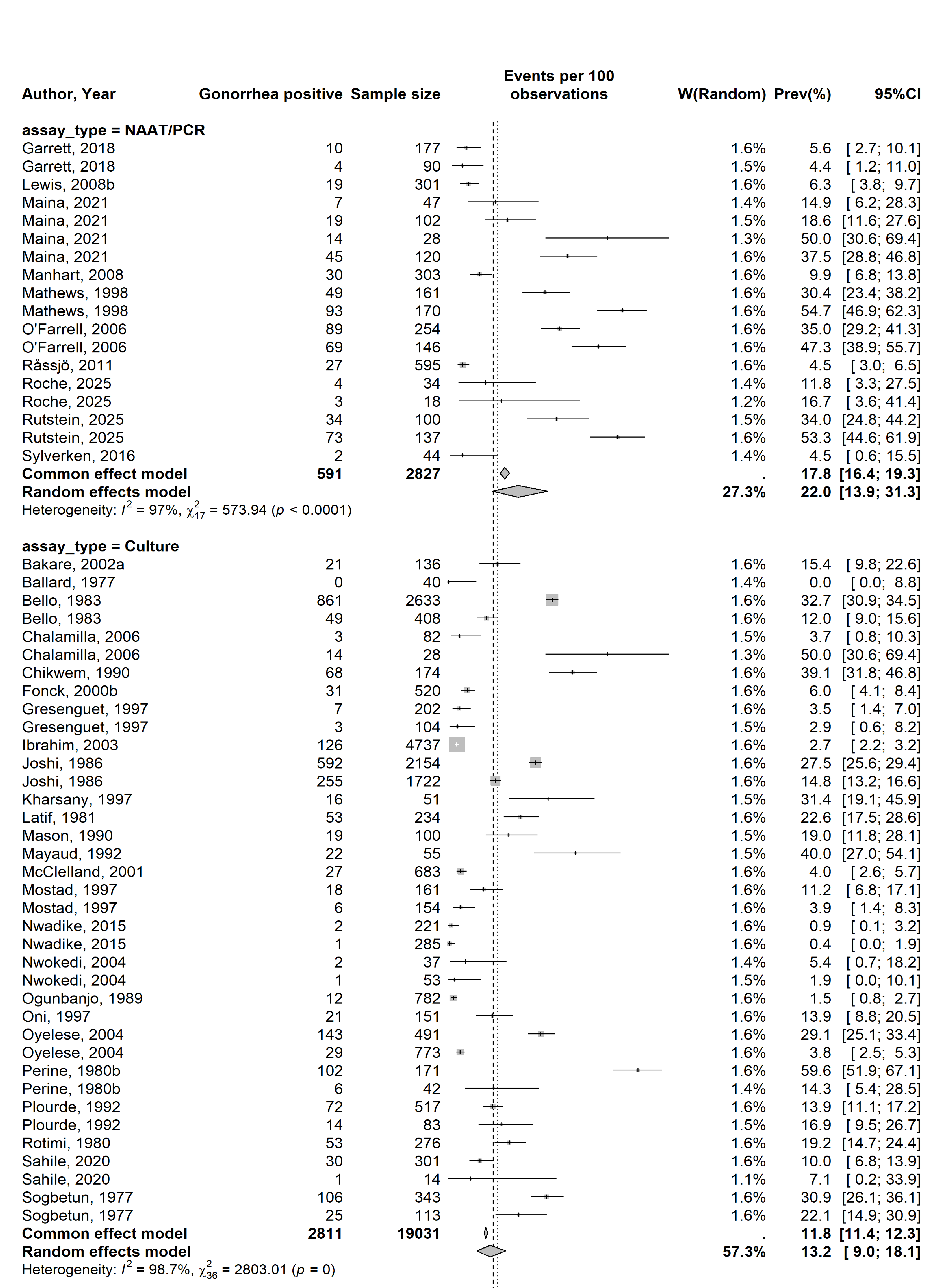
**

**
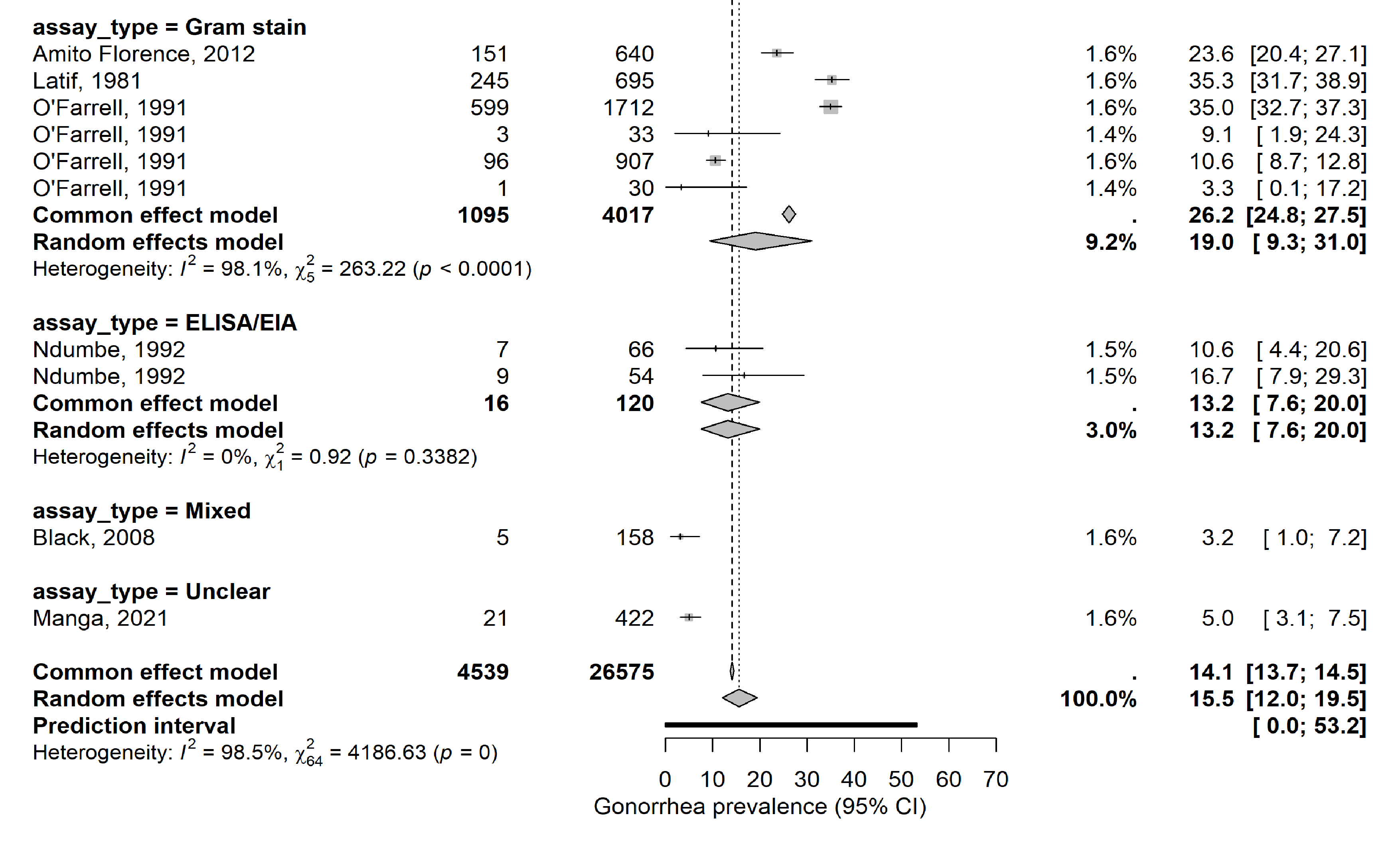
**

Abbreviations: CI, Confidence interval; EIA, Enzyme Immunoassay; ELISA, Enzyme-linked immunosorbent assay; NAAT, Nucleic acid amplification test; PCR, Polymerase chain reaction; STI, Sexually transmitted infection.

1. Sexual contacts of persons infected with NG/CT

**
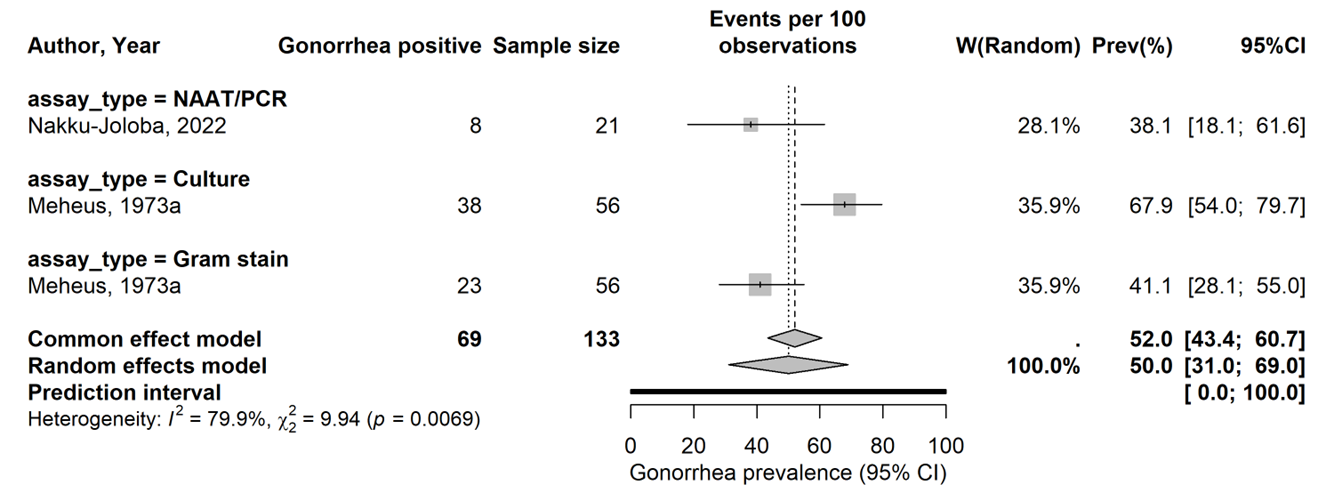
**

Abbreviations: CI, Confidence interval; CT, *Chlamydia trachomatis*; NAAT, Nucleic acid amplification test; NG, *Neisseria gonorrhoeae;* PCR, Polymerase chain reaction.

1. Patients with confirmed or suspected STIs and related infections

**
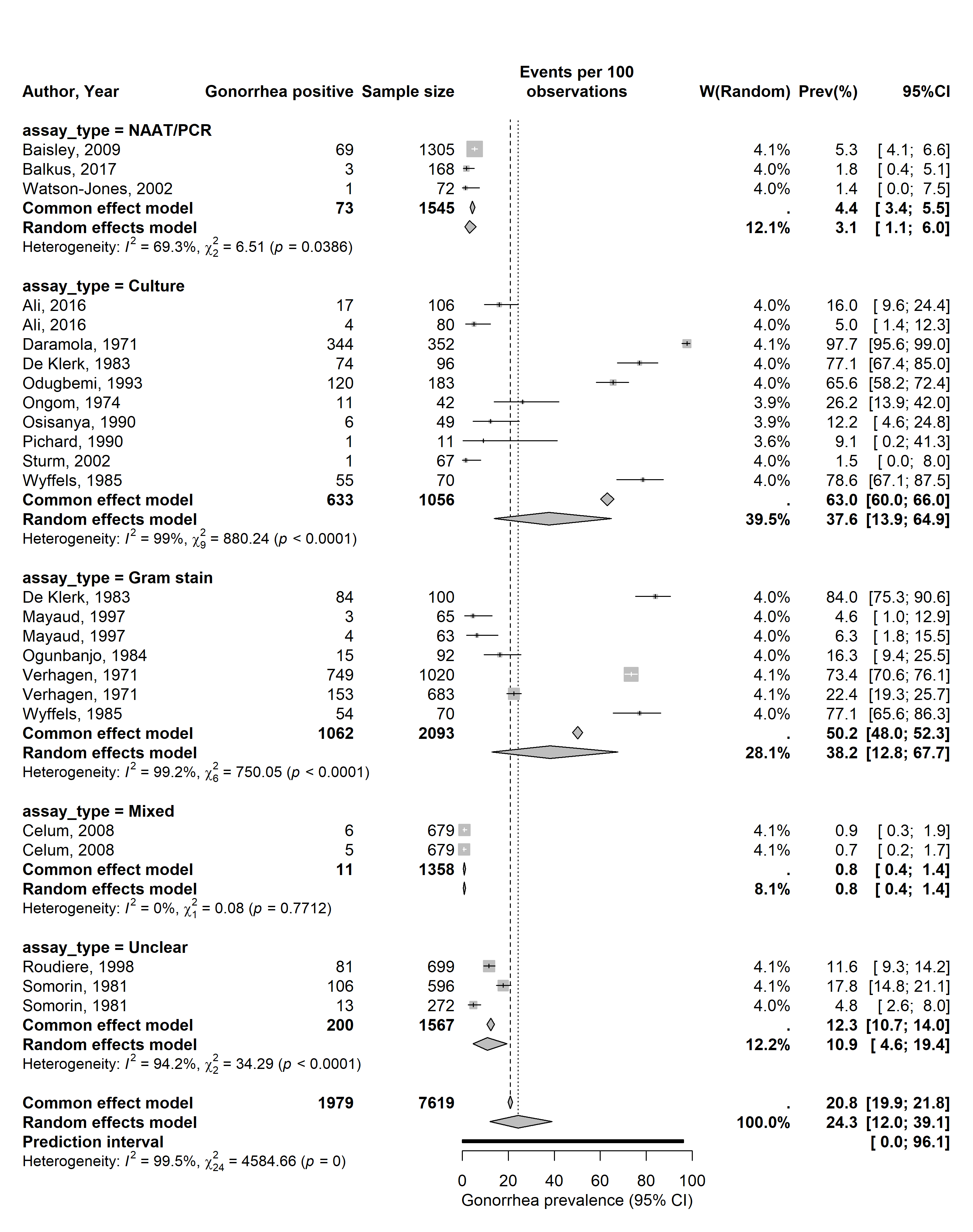
**

Abbreviations: CI, Confidence interval; NAAT, Nucleic acid amplification test; PCR, Polymerase chain reaction; STI, Sexually transmitted infection.

1. Individuals living with HIV and individuals in HIV-discordant couples


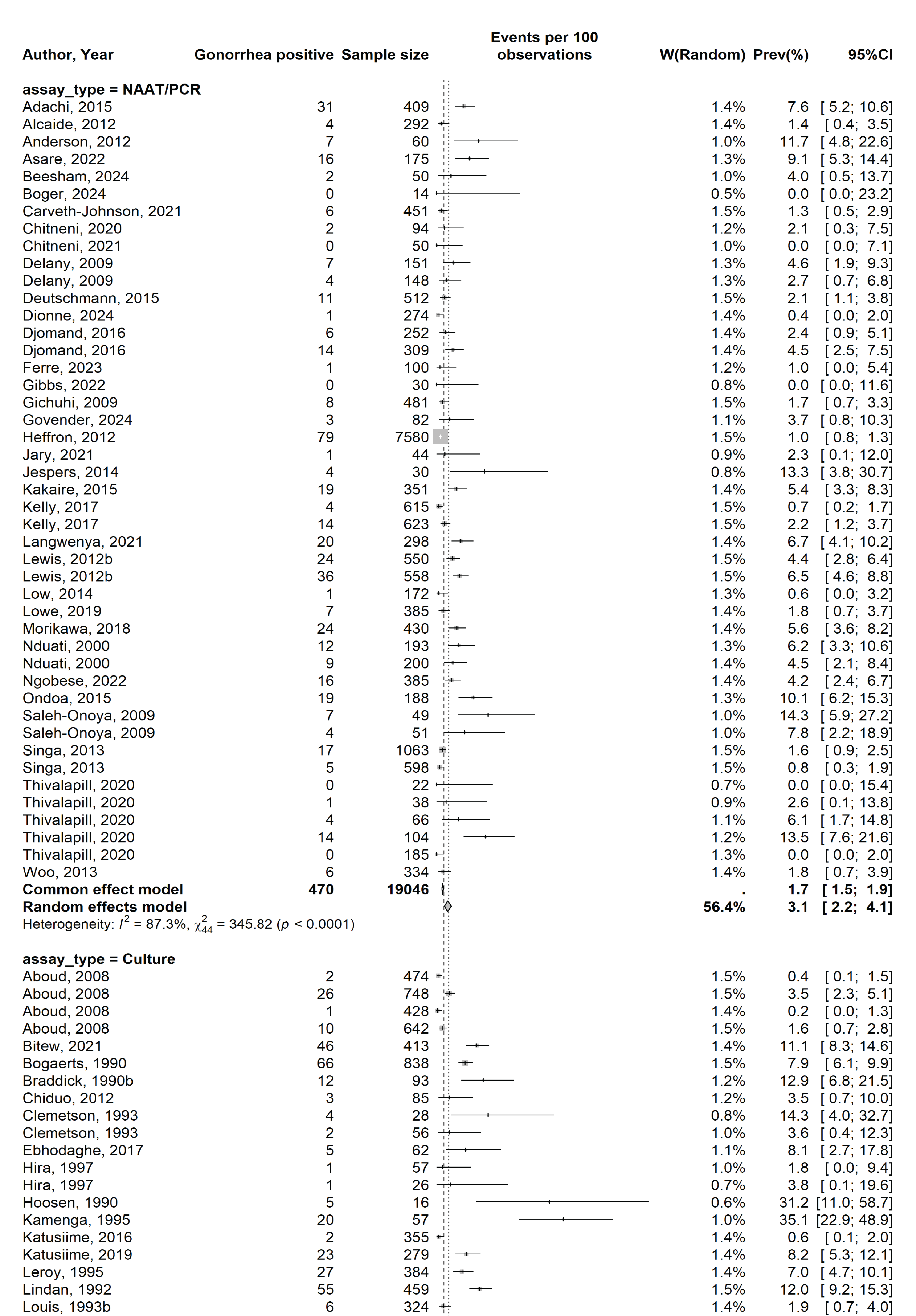


**
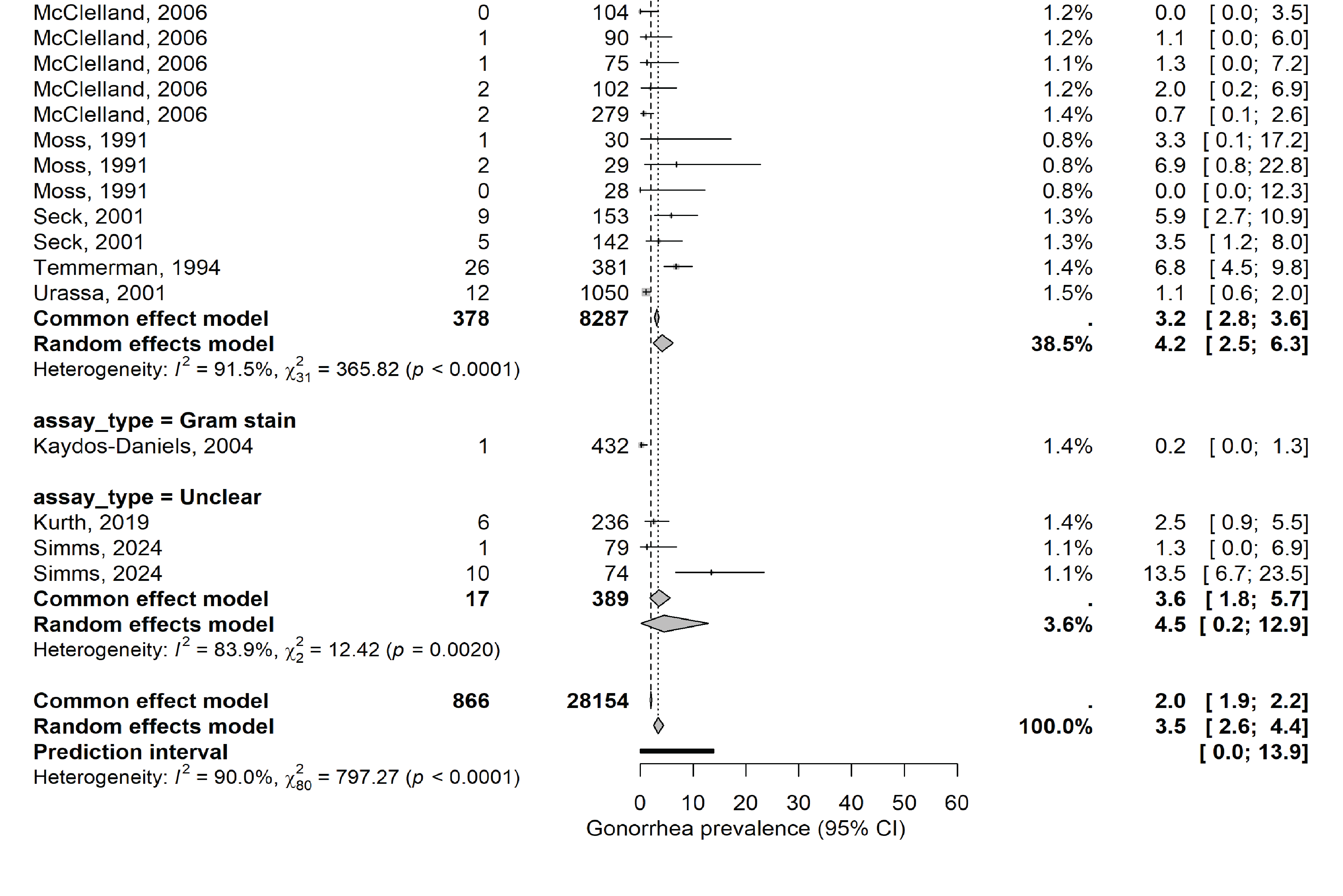
**

Abbreviations: CI, Confidence interval; HIV, human immunodeficiency virus; NAAT, Nucleic acid amplification test; PCR, Polymerase chain reaction.

1. Other populations^‡^


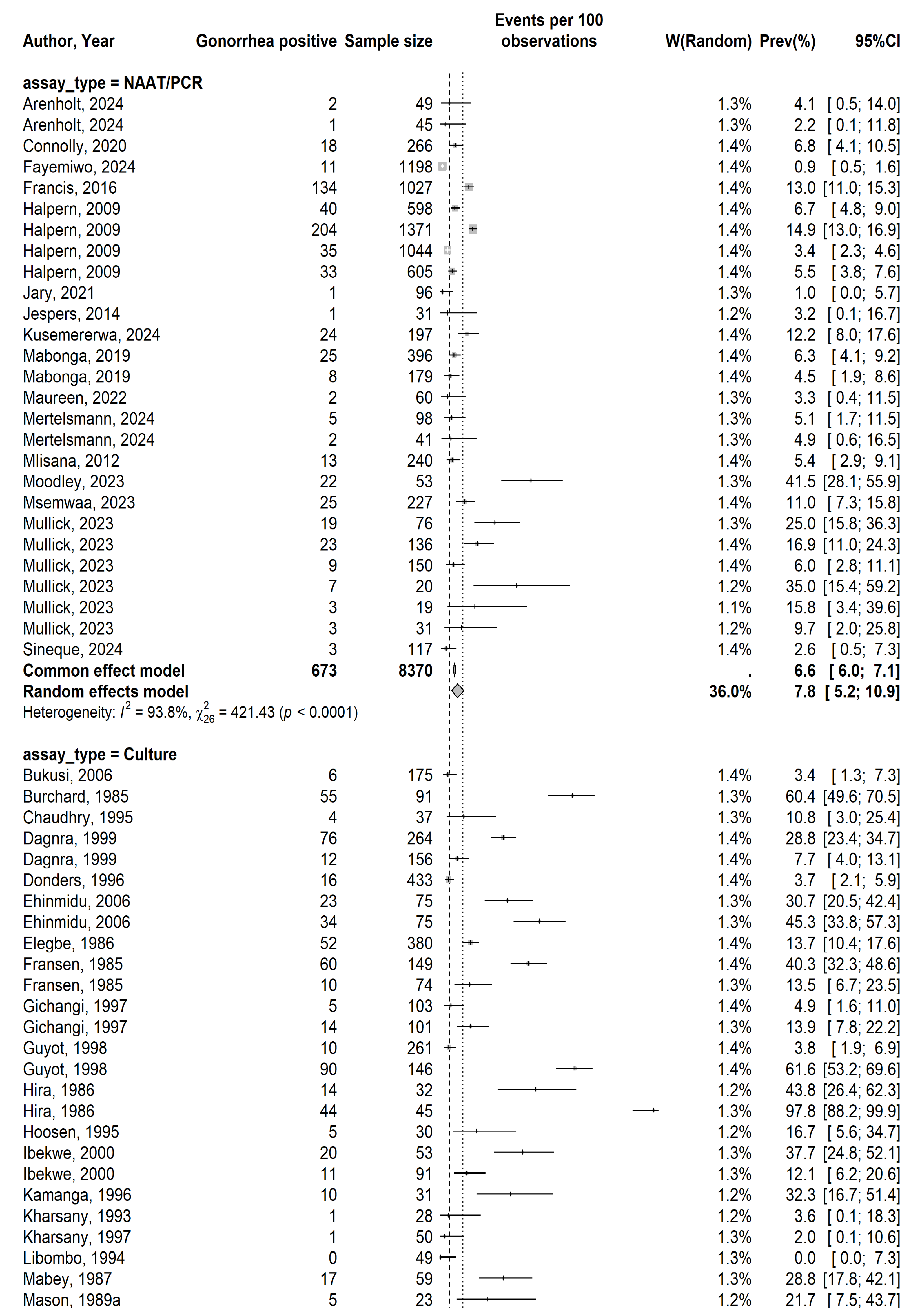


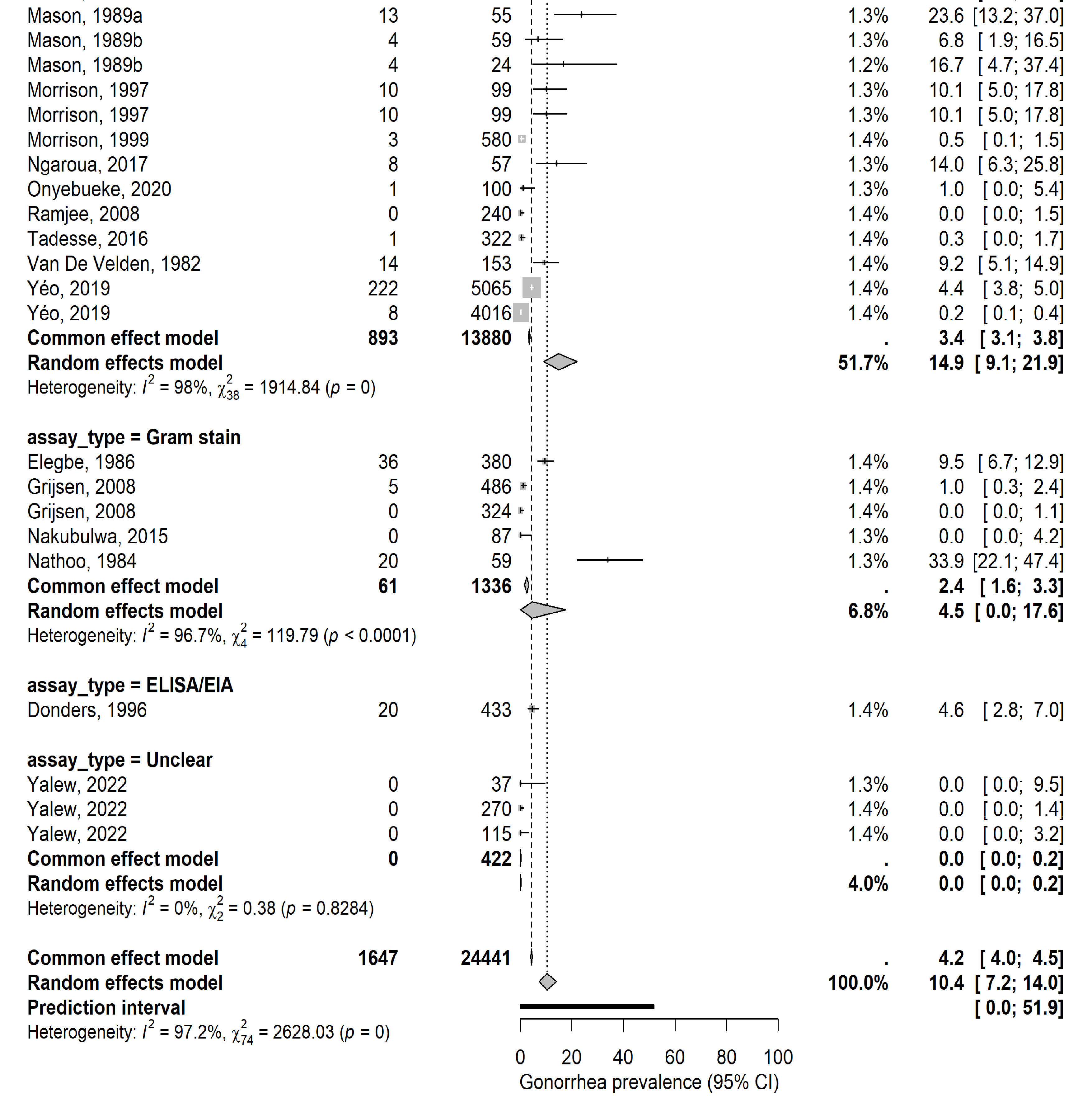


Abbreviations: CI, Confidence interval; EIA, Enzyme immunoassay; ELISA, Enzyme-linked immunosorbent assay; NAAT, Nucleic acid amplification test; NG, *Neisseria gonorrhoeae*; PCR, Polymerase chain reaction.

**^‡^**The "Other populations" category comprises groups with an undetermined risk of NG infection, including cervical cancer patients, victims of sexual assault, and mixed or undefined populations.

# Fig H. Forest plots for anorectal infection. Forest plots presenting outcomes of the pooled mean NG prevalence in anorectal specimens among different populations in sub-Saharan Africa.

1. General populations

**
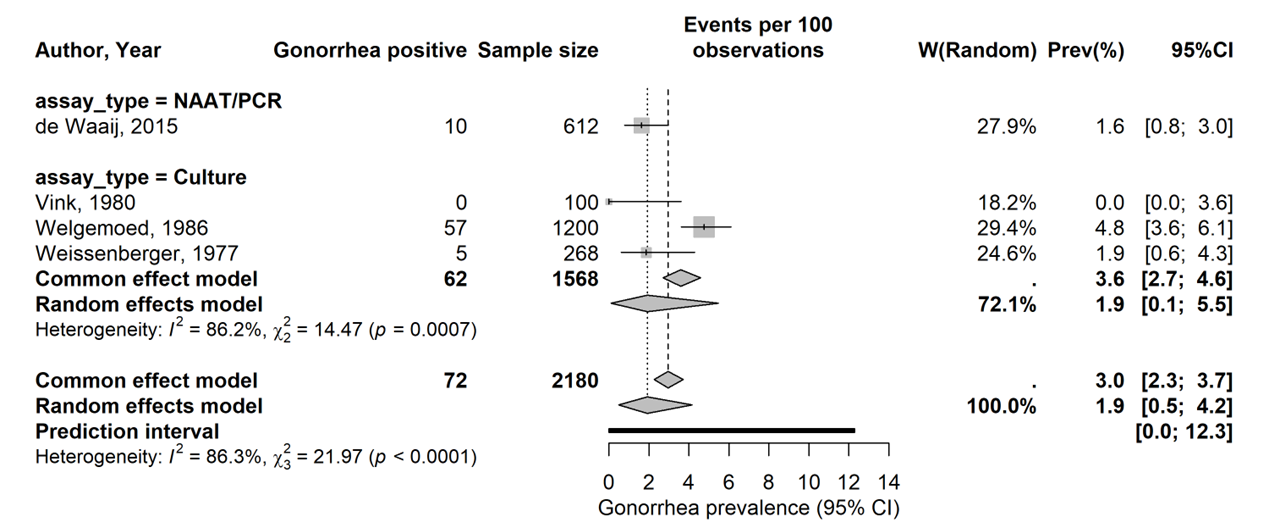
**

Abbreviations: CI, Confidence interval; NAAT, Nucleic acid amplification test; PCR, Polymerase chain reaction.

1. Female sex workers

**
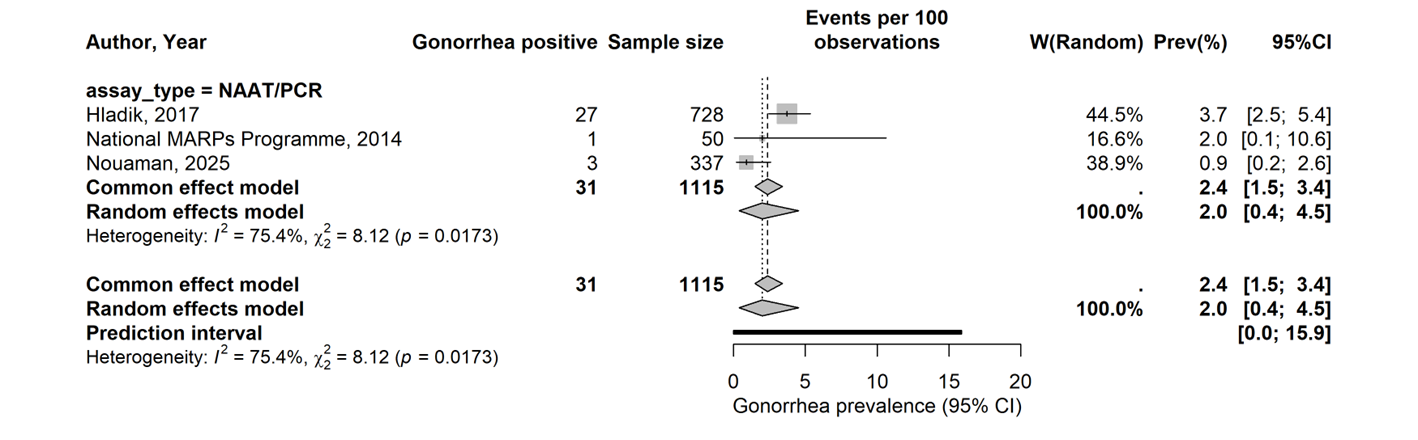
**

Abbreviations: CI, Confidence interval; NAAT, Nucleic acid amplification test; PCR, Polymerase chain reaction.

1. Men who have sex with men^*^

**
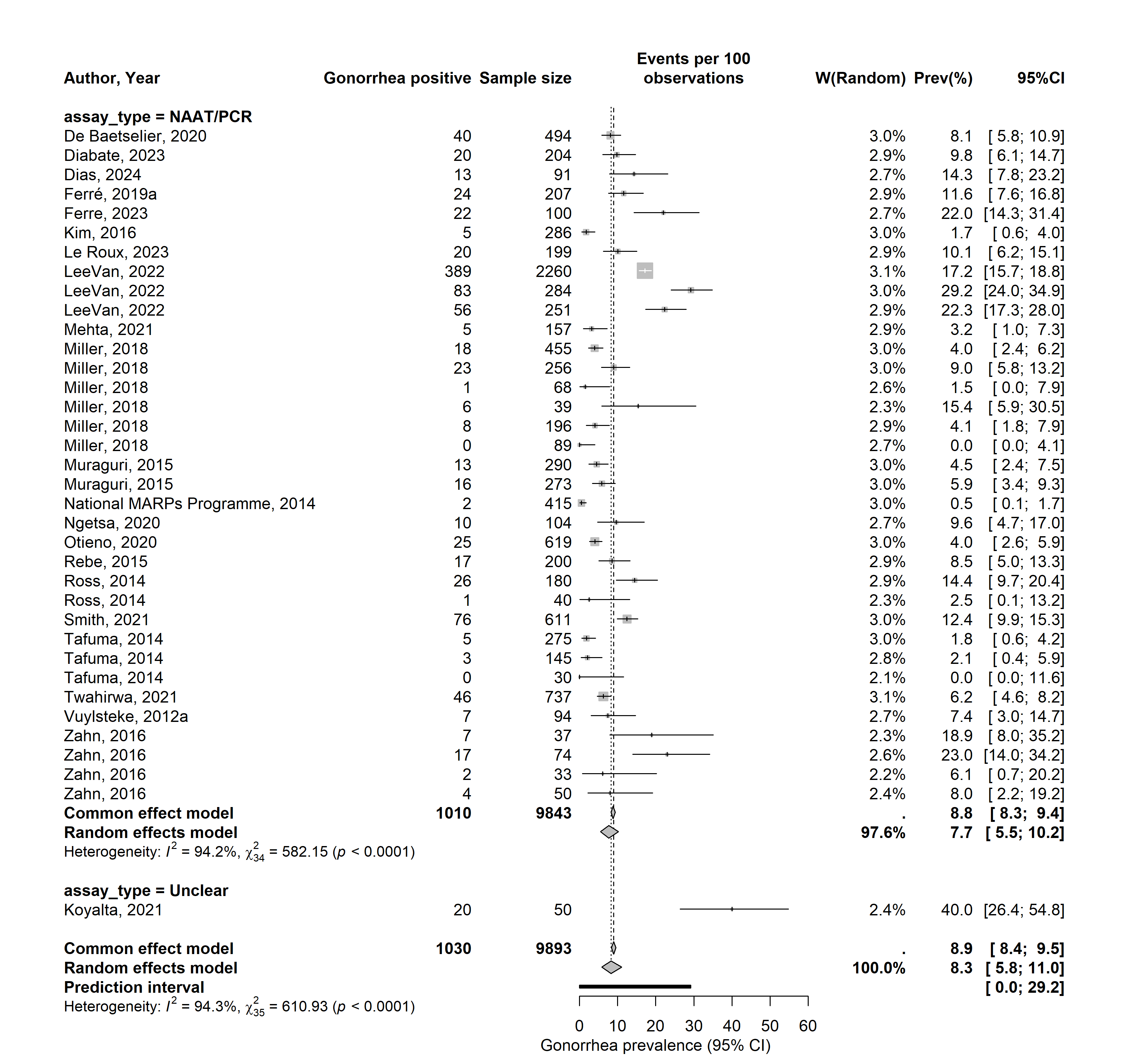
**

Abbreviations: CI, Confidence interval; NAAT, Nucleic acid amplification test; PCR, Polymerase chain reaction.

^*^The term “men who have sex with men” is used inclusively and encompasses men who have sex with men, transgender people, and male or transgender sex workers.

1. Symptomatic men

**
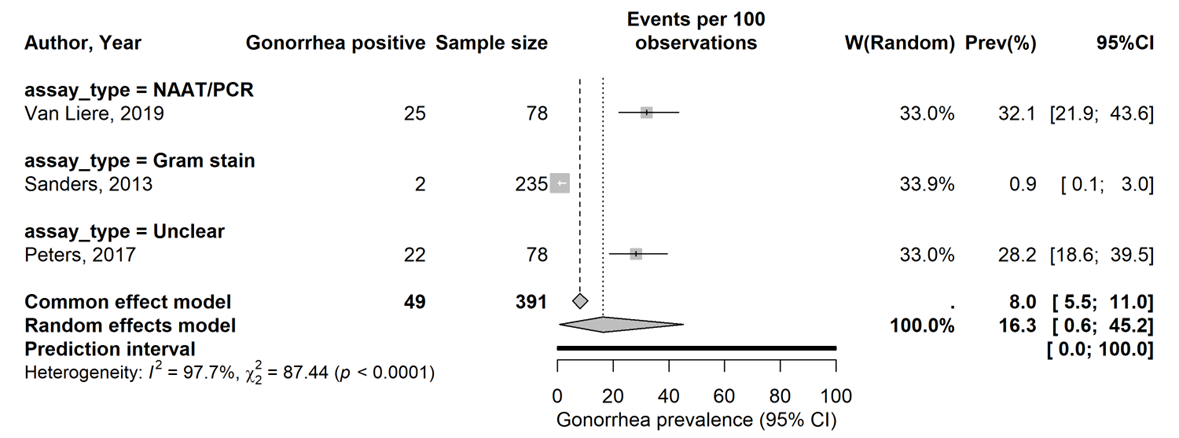
**

Abbreviations: CI, Confidence interval; NAAT, Nucleic acid amplification test; PCR, Polymerase chain reaction.

.

# **Fig I.** Forest plots for oropharyngeal infection. Forest plots presenting outcomes of the pooled mean NG prevalence in oropharyngeal specimens among different populations in sub-Saharan Africa.

1. General populations


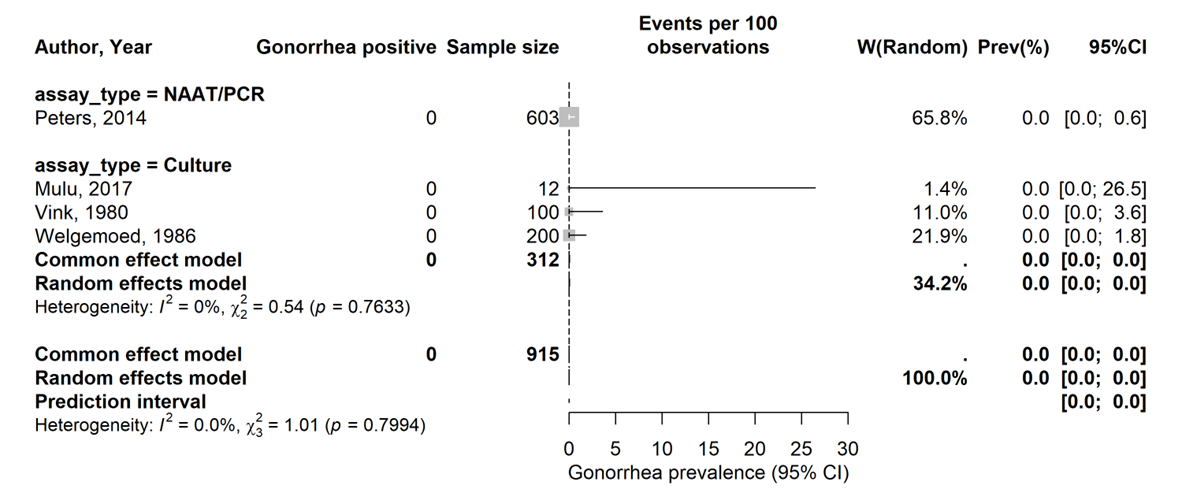


Abbreviations: CI, Confidence interval; NAAT, Nucleic acid amplification test; PCR, Polymerase chain reaction.

1. Men who have sex with men^*^


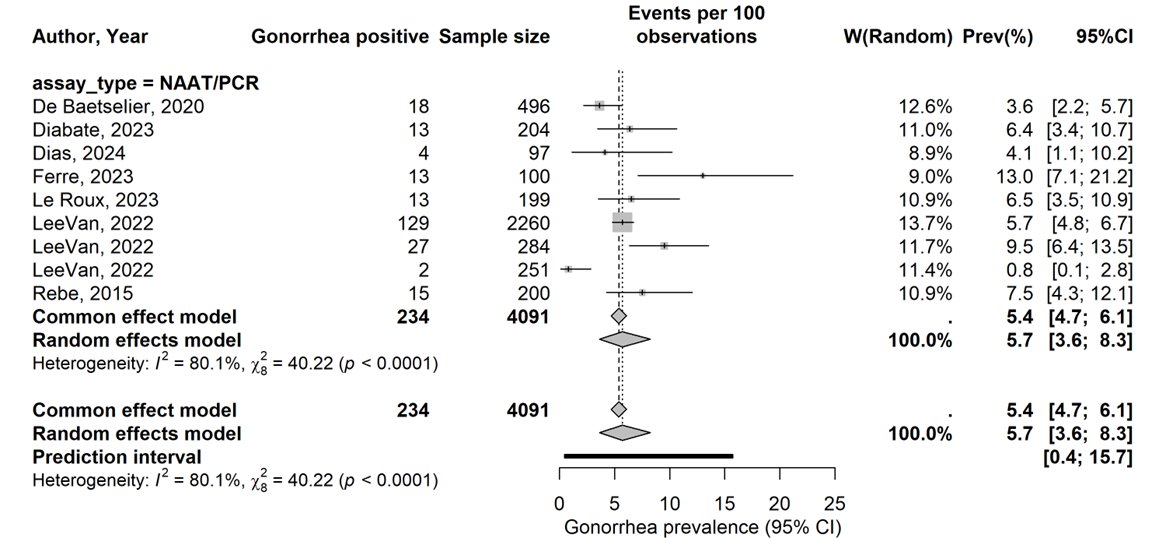


Abbreviations: CI, Confidence interval; NAAT, Nucleic acid amplification test; PCR, Polymerase chain reaction.

^*^The term “men who have sex with men” is used inclusively and encompasses men who have sex with men, transgender people, and male or transgender sex workers.

1. Symptomatic men

**
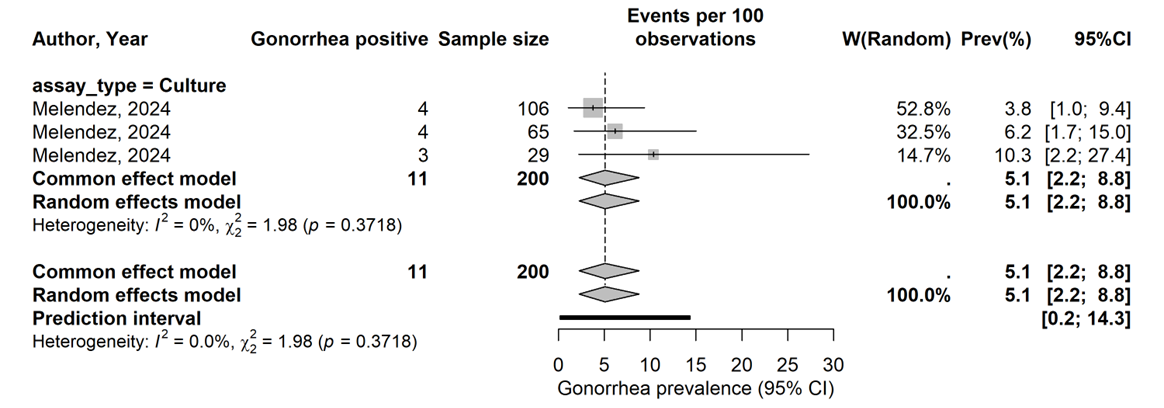
**

Abbreviations: CI, Confidence interval.

#

# **Table H.** NG prevalence estimates for select populations. Pooled mean prevalence of NG infection in sub-Saharan Africa for select populations, stratified by anatomical site.

| **Population type** | **Stratified prevalence measures** | **Sample** | **NG prevalence (%)** | | **Pooled mean NG prevalence (%)** | **Heterogeneity measures** | | | |  |
| --- | --- | --- | --- | --- | --- | --- | --- | --- | --- | --- |
|  | **Total n** | **Total N** | **Range** | **Median** | **Mean**  **(95% CI)** | **Q^*^**  **(p-value)** | **I²**^†^ **(%)**  **(95% CI)** | **Prediction interval^‡^ (%)** | | |
| **General populations** | | | | | | | | | |  |
| **Pregnant women/Antenatal clinic attendees** | | | | | | | | | |  |
| Urogenital | 165 | 66,546 | 0.0-68.0 | 3.0 | 3.1 (2.6-3.7) | 2,022.2 (p <0.001) | 91.9 (91.0-92.7) | | 0.0-12.5 |  |
| Anorectal | 1 | 1,200 | - | - | 4.8 (3.6-6.0) | - | - | | - |  |
| Oropharyngeal | 1 | 200 | - | - | 0.0 (0.0-1.5) | - | - | | - |  |
| Blood tested for antibodies^§^ | 6 | 289 | 4.8-28.0 | 26.5 | 20.2 (11.3-30.8) | 26.1 (p <0.001) | 80.8 (58.7-91.1) | | 0.4-55.0 |  |
| Unspecified/mixed | 9 | 2,737 | 0.9-14.0 | 4.7 | 5.2 (2.8-8.3) | 58.5 (p <0.001) | 86.3 (76.0-92.2) | | 0.0-18.2 |  |
| **General populations other than pregnant women** | | | | | | | | | |  |
| Urogenital | 541 | 254,510 | 0.0-71.8 | 2.6 | 3.2 (2.8-3.6) | 9,111.3 (p <0.001) | 94.1 (93.7- 94.4) | | 0.0- 15.8 |  |
| Anorectal | 3 | 980 | 0.0-1.9 | 1.6 | 1.4 (0.7-2.2) | 2.7 (p = 0.257) | 26.5 (0.0-92.4) | | 0.1-3.5 |  |
| Oropharyngeal | 3 | 715 | 0.0-0.0 | 0.0 | 0.0 (0.0-0.0) | 1.0 (p = 0.619) | 0.0 (0.0-89.6) | | 0.0-0.1 |  |
| Blood tested for antibodies^§^ | 3 | 334 | 0.0-17.5 | 7.5 | 6.8 (0.0-20.9) | 19.3 (p <0.001) | 89.7 (72.1-96.2) | | 0.0-82.1 |  |
| Unspecified/mixed | 65 | 2,049,879 | 0.0-24.5 | 5.0 | 3.5 (2.5-4.8) | 13,529.2 (p <0.001) | 99.5 (99.5-99.6) | | 0.0-17.9 |  |
| **Intermediate-risk populations** | | | | | | | | | |  |
| **Clients of female sex workers** | | | | | | | | | |  |
| Urogenital | 10 | 4,839 | 0.6-5.4 | 3.0 | 2.8 (1.9-3.7) | 25.7 (p = 0.002) | 64.9 (31.1-82.2) | | 0.6-6.3 |  |
| Anorectal | 1 | 573 | - | - | 0.0 (0.0-0.5) | - | - | | - |  |
| **Military men** | | | | | | | | | |  |
| Urogenital | 5 | 3,988 | 0.5-2.3 | 1.0 | 1.1 (0.6-1.7) | 9.9 (p = 0.043) | 59.5 (0.0-84.9) | | 0.1-3.1 |  |
| Blood tested for antibodies^§^ | 1 | 2,650 | - | - | 1.6 (1.1-2.1) | - | - | | - |  |
| Unspecified/mixed | 1 | 20 | - | - | 65.0 (44.0-86.0) | - | - | | - |  |
| **Truck and taxi drivers** | | | | | | | | | |  |
| Urogenital | 20 | 6,553 | 0.0-5.5 | 1.8 | 1.3 (0.9-1.7) | 26.0 (p = 0.129) | 27.0 (0.0-57.7) | | 0.4-2.6 |  |
| **Miners, fishermen, and plantation workers** | | | | | | | | | |  |
| Urogenital | 8 | 2,981 | 0.0-9.9 | 3.3 | 2.8 (1.3-4.6) | 25.6 (p = 0.001) | 72.7 (44.1-86.6) | | 0.0-10.0 |  |

Abbreviations: CI, Confidence interval; NG, *Neisseria gonorrhoeae*.

A minimum of three studies was required to perform a meta-analysis.

^*^Q: The Cochran’s Q statistic is a measure assessing the existence of heterogeneity in pooled outcome measures, here NG prevalence.

^†^I^2^: A measure that assesses the magnitude of between-study variation that is due to true differences in NG prevalence across studies rather than chance.

^‡^Prediction interval: A measure that estimates the distribution (95% interval) of true NG prevalence around the estimated mean.

^§^Blood tests for antibodies include haemagglutination assays, complement fixation tests, and measurements of immunoglobulins (e.g., IgG, IgA).

# **Table I.** NG prevalence estimates by sampling method. Pooled mean prevalence of NG infection in sub-Saharan Africa for populations of public health importance, stratified by studies using A) probability-based sampling and B) non-probability-based sampling.

| **Population type** | **Stratified prevalence measures** | **Sample** | **NG prevalence (%)** | | | **Pooled mean NG prevalence (%)** | **Heterogeneity measures** | | | |
| --- | --- | --- | --- | --- | --- | --- | --- | --- | --- | --- |
|  | **Total n** | **Total N** | **Range** | | **Median** | **Mean**  **(95% CI)** | **Q^*^**  **(p-value)** | **I²**^†^ **(%)**  **(95% CI)** | | **Prediction interval^‡^ (%)** |
| 1. **Probability-based sampling** | | | | | | | | | | |
| **General populations** | | | | | | | | | | |
| Urogenital | 227 | 112,095 | 0.0-33.3 | 1.9 | | 2.2 (1.8-2.6) | 2,963.6 (p <0.001) | 92.4 (91.7-93.0) | 0.0-10.3 | |
| Anorectal | - | - | - | - | | - | - | - | - | |
| Oropharyngeal | - | - | - | - | | - | - | - | - | |
| Blood tested for antibodies^§^ | 2 | 157 | 4.8-7.5 | 6.2 | | 6.2 (3.5-8.8) | - | - | - | |
| Unspecified/mixed | 5 | 1,796 | 0.0-10.0 | 1.0 | | 1.6 (0.1-4.7) | 31.2 (p <0.001) | 87.2 (72.4-94.0) | 0.0-17.2 | |
| **Female sex workers** | | | | | | | | | | |
| Urogenital | 55 | 20,121 | 0.0-51.2 | 7.1 | | 8.4 (6.3-10.8) | 1,385.9 (p <0.001) | 96.1 (95.5-96.6) | 0.0-30.5 | |
| Anorectal | 2 | 778 | 2.0-3.7 | 2.9 | | 2.9 (1.2-4.5) | - | - | - | |
| Blood tested for antibodies^§^ | - | - | - | - | | - | - | - | - | |
| Unspecified/mixed | 2 | 1,347 | 4.7-17.4 | 11.1 | | 11.1 (0.0-23.5) | - | - | - | |
| **Men who have sex with men^¶^** | | | | | | | | | | |
| Urogenital | 22 | 7,689 | 0.0-9.0 | 2.8 | | 2.8 (2.1-3.5) | 45.4 (p = 0.002) | 53.7 (24.9-71.5) | 0.8-5.7 | |
| Anorectal | 22 | 7,591 | 0.0-29.1 | 4.0 | | 6.5 (3.9-9.8) | 512.6 (p <0.001) | 95.9 (94.8-96.8) | 0.0-27.3 | |
| Oropharyngeal | 4 | 2,999 | 0.9-9.4 | 6.1 | | 5.0 (1.8-9.6) | 26.8 (p <0.001) | 88.8 (73.9-95.2) | 0.0-26.2 | |
| Unspecified/mixed | 12 | 2,423 | 0.0-18.0 | 1.8 | | 3.3 (1.1-6.6) | 150.3 (p <0.001) | 92.7 (89.1-95.1) | 0.0-20.8 | |
| 1. **Non-probability-based sampling** | | | | | | | | | | |
| **General populations** | | | | | | | | | | |
| Urogenital | 479 | 208,961 | 0.0-71.8 | 3.3 | | 3.7 (3.3-4.2) | 7,768.6 (p <0.001) | 93.8 (93.5-94.2) | 0.0-17.1 | |
| Anorectal | 4 | 2,180 | 0.0-4.8 | 1.7 | | 1.9 (0.5-4.2) | 22.0 (p <0.001) | 86.3 (66.8-94.4) | 0.0-12.3 | |
| Oropharyngeal | 4 | 915 | 0.0-0.0 | 0.0 | | 0.0 (0.0-0.0) | 1.0 (p = 0.799) | 0.0 (0.0-84.7) | 0.0-0.0 | |
| Blood tested for antibodies^§^ | 7 | 466 | 0.0-28.0 | 26.0 | | 18.5 (9.4-29.6) | 29.8 (p <0.001) | 79.9 (59.0-90.1) | 0.0-58.0 | |
| Unspecified/mixed | 69 | 2,050,820 | 0.0-24.5 | 5.0 | | 3.9 (2.8-5.1) | 13,855.4 (p <0.001) | 99.5 (99.5-99.5) | 0.0-18.2 | |
| **Female sex workers** | | | | | | | | | | |
| Urogenital | 145 | 47,288 | 0.0-77.5 | 11.6 | | 12.8 (10.7-15.0) | 5,155.1 (p <0.001) | 97.2 (97.0-97.4) | 0.0-46.4 | |
| Anorectal | 1 | 337 | - | - | | 0.9 (0.1-2.2) | - | - | - | |
| Blood tested for antibodies^§^ | 1 | 642 | - | - | | 53.3 (49.4-57.2) | - | - | - | |
| Unspecified/mixed | 6 | 1,685 | 1.0-32.3 | 8.5 | | 9.0 (2.7-18.0) | 90.7 (p <0.001) | 94.5 (90.5-96.8) | 0.0-46.8 | |
| **Men who have sex with men^¶^** | | | | | | | | | | |
| Urogenital | 24 | 4,304 | 0.0-7.5 | 3.0 | | 2.9 (2.3-3.6) | 27.4 (p = 0.238) | 16.2 (0.0-48.9) | 1.4-4.9 | |
| Anorectal | 14 | 2,302 | 3.2-40.0 | 8.9 | | 11.4 (7.3-16.3) | 96.1 (p <0.001) | 86.5 (78.9-91.3) | 0.2-33.7 | |
| Oropharyngeal | 5 | 1,092 | 3.6-13.0 | 6.5 | | 6.3 (3.7-9.5) | 13.4 (p = 0.009) | 70.2 (24.1-88.3) | 0.5-17.3 | |
| Unspecified/mixed | 2 | 489 | 8.1-9.8 | 9.0 | | 9.0 (7.3-10.6) | - | - | - | |

Abbreviations: CI, Confidence interval; NG, *Neisseria gonorrhoeae*.

A minimum of three studies was required to perform a meta-analysis.

^*^Q: The Cochran’s Q statistic is a measure assessing the existence of heterogeneity in pooled outcome measures, here NG prevalence.

^†^I^2^: A measure that assesses the magnitude of between-study variation that is due to true differences in NG prevalence across studies rather than chance.

^‡^Prediction interval: A measure that estimates the distribution (95% interval) of true NG prevalence around the estimated mean.

^§^Blood tests for antibodies include haemagglutination assays, complement fixation tests, and measurements of immunoglobulins (e.g., IgG, IgA).

^¶^The term “men who have sex with men” is used inclusively and encompasses men who have sex with men, transgender people, and male or transgender sex workers.

# References

1. Page MJ, McKenzie JE, Bossuyt PM, Boutron I, Hoffmann TC, Mulrow CD, et al. The PRISMA 2020 statement: an updated guideline for reporting systematic reviews. BMJ. 2021;372:n71. Epub 2021/03/31. doi: 10.1136/bmj.n71. PubMed PMID: 33782057; PubMed Central PMCID: PMCPMC8005924.

2. Hoy D, Brooks P, Woolf A, Blyth F, March L, Bain C, et al. Assessing risk of bias in prevalence studies: modification of an existing tool and evidence of interrater agreement. J Clin Epidemiol. 2012;65(9):934-9. Epub 2012/06/30. doi: 10.1016/j.jclinepi.2011.11.014. PubMed PMID: 22742910.

3. Munn Z, Moola S, Lisy K, Riitano D, Tufanaru C. Methodological guidance for systematic reviews of observational epidemiological studies reporting prevalence and cumulative incidence data. Int J Evid Based Healthc. 2015;13(3):147-53. Epub 2015/09/01. doi: 10.1097/XEB.0000000000000054. PubMed PMID: 26317388.

4. Furuya-Kanamori L, Barendregt JJ, Doi SAR. A new improved graphical and quantitative method for detecting bias in meta-analysis. Int J Evid Based Healthc. 2018;16(4):195-203. Epub 2018/04/06. doi: 10.1097/XEB.0000000000000141. PubMed PMID: 29621038.
