# Supplementary material for: Prevalence and epidemiological patterns of Neisseria gonorrhoeae infection in sub-Saharan Africa, 1964–2025: Systematic review, meta-analyses, and meta-regressions
Source: PLoS Med. 2026 Jun 23;23(6):e1004936. doi: 10.1371/journal.pmed.1004936 (PMC13289870; doi:10.1371/journal.pmed.1004936)
Supplement: Attachment — Submitted filename: Response to reviewers.docx [file pmed.1004936.s004.docx]

**Prevalence, patterns, and trends of Neisseria gonorrhoeae infection in sub-Saharan Africa, 1964–2025: systematic review, meta-analyses, and meta-regressions**

REPLY TO EDITORIAL AND REVIEWERS’ COMMENTS

We are grateful to the editor, editorial board, and reviewers for assessing our work and for their insightful and useful feedback and suggestions. Please find below a point-by-point reply addressing each of the comments. We have also incorporated these suggestions in the revised manuscript, as noted below. We would be pleased to address any additional matters, should that be necessary.

Note: All references to the revised manuscript pertain to the marked copies of the manuscript files including changes implemented through “track changes”.

*Dear Dr Abu-Raddad,*

*Many thanks for submitting your manuscript "Prevalence, patterns, and trends of Neisseria gonorrhoeae infection in sub-Saharan Africa, 1964–2025: systematic review, meta-analyses, and meta-regressions" (PMEDICINE-D-26-00281R1) to PLOS Medicine. The paper has been reviewed by subject experts and a statistician; their comments are included below and can also be accessed here:*

*As you will see, the reviewers find the study interesting and recommend revising the manuscript to address their independent concerns. In particular, the reviewers request the inclusion of the study protocol and substantially more detail on the methodology of the search and analyses, as well as presentation of subregional estimates and sub-pooled estimates for different population groups. We ask you to temper claims and discuss study limitations, and to extend a caveated discussion elaborating on potential policy implications of the findings. After discussing the paper with the editorial team and an academic editor with relevant expertise, I'm pleased to invite you to revise the paper in response to the reviewers' and academic editor's comments. We plan to send the revised paper to some or all of the original reviewers, and we cannot provide any guarantees at this stage regarding publication.*

Comment: We thank the editor for the time and effort put into handling this submission, the assessment of our work, and the constructive feedback on our manuscript that enriched it and improved its readability. All revisions indicated in this comment have now been addressed, as detailed in our responses below. Please find below a point-by-point reply addressing each of the editorial and reviewers’ comments.

*Please update your search to within 6 months of the anticipated date of resubmission.*

Answer: We appreciate this request. However, implementing it is difficult given the scope and complexity of the study. This review represents a large-scale effort, including over 1,600 prevalence measures, substantially exceeding the size of a typical systematic review. The volume of data extracted is more than an order of magnitude greater than average. The search has already been updated twice (Methods, Page 7, Paragraph 2), in part reflecting the substantial time required to complete analyses following each update.

Updating the search at this stage would require several weeks for screening and data extraction, followed by a complete re-analysis of the dataset. Even the addition of a single study would necessitate re-running all analyses and updating all tables, figures, and supplementary materials. Given the scale of the study, the complexity of the methodology, the number of outcomes assessed, and the need for rigorous validation, these analyses would require at least several additional weeks to complete, including detailed checks to ensure accuracy across all outputs and the large number of inserts presented in both the manuscript and supplementary files. Therefore, this would result in a substantial delay in the revision and resubmission of the manuscript.

At the time of the original submission, the search was updated within approximately 6 months, consistent with best practices for systematic reviews and compliant with *PLOS Medicine* requirements. Importantly, any further update is unlikely to materially affect the findings. The dataset spans a broad time horizon (1964–2025), and extending the search by a few additional months within a body of evidence covering more than 60 years is very unlikely to meaningfully alter the estimates or conclusions. The evidence base is large, stable, and unlikely to have undergone changes over such a short period that would affect the study outcomes.

Finally, this study is epidemiological in nature and provides population-level evidence that does not change rapidly over short timeframes. It does not also evaluate interventions, compare management strategies, or provide clinical decision-making, where newly emerging evidence could alter treatment recommendations or clinical guidelines.

Given these considerations, updating the search to within 6 months of resubmission would entail substantial additional work without a commensurate benefit to the validity or interpretation of the findings.

This point has now been noted in the Discussion (Discussion, Page 23, Paragraph 4).

*Please clarify in the text if the search was restricted to English language publications.*

Answer: The search was not restricted to English-language publications and was conducted without language restrictions. Where necessary, titles, abstracts, and full texts were translated using Google Translate to facilitate screening and data extraction of non-English articles. This has now been clarified in the Methods (Methods, Page 7, Paragraph 2).

*If new code was generated for any analyses, please deposit it in a public repository and provide a doi in the Data Availability statement.*

Answer: No new code was developed, as existing, publicly available packages were used.

*Please include the funder URL(s) in the manuscript metadata.*

Answer: These instructions have been noted and addressed as indicated.

*The last sentence of the Methods and Findings in the Abstract should state the methodological limitations.*

Answer: The abstract has been revised accordingly, and the final sentence of the Methods and Findings now explicitly highlights the key methodological limitations of the study (Abstract).

*Please add to the introduction a statement that at the time of writing, there is no licensed vaccine against NG.*

Answer: The Introduction has been revised to clarify that, at the time of writing, there is no licensed vaccine against NG (Introduction, Page 5, Paragraph 2).

*Please clarify why the GRADE approach is not used to assess certainty of evidence.*

Answer: The GRADE approach was developed to assess the quality of evidence and formulate recommendations related to alternative management strategies, clinical interventions, or health policy decisions [1]. Its application relies on comparative evidence evaluating interventions in terms of benefits, harms, and patient-important outcomes to support clinical decision-making [1].

In contrast, this systematic review focuses solely on the epidemiology of NG infection and does not assess interventions, compare management strategies, or address clinical decision-making processes. As such, there are no interventions or management alternatives that would warrant the application of GRADE. Moreover, GRADE is not intended for purely epidemiological questions related to disease burden or prevalence, where domains such as indirectness, inconsistency, and imprecision in relation to interventions are not directly applicable [1].

Consistent with established best practices for epidemiological reviews, a robust risk of bias assessment tailored to the nature of the included evidence was applied (Methods, Section of “Precision, risk of bias, and publication bias assessments”, Page 10-11; and S3 Table). This approach ensures a transparent and appropriate evaluation of study quality without using a framework designed for intervention studies.

To address this comment, this point has now been clarified in the revised manuscript (Methods, Page 10, Paragraph 5; and S1 Table).

*Please clarify why risk of publication bias is marked as N/A in the PRISMA checklist.*

Answer: We thank you for highlighting this error. Publication bias was indeed assessed, and the PRISMA checklist has been corrected accordingly (S1 Table).

*Is 'mixed sexes', defined in Table 1, used in the cited articles? Please review your manuscript and edit to ensure compliance with our inclusive language requirements* [*https://journals.plos.org/plosmedicine/s/human-subjects-research#loc-categorization*](https://journals.plos.org/plosmedicine/s/human-subjects-research#loc-categorization)

Answer: We have reviewed the use of the term “mixed sexes” and found that it was not consistently used in the original studies. Accordingly, to align with inclusive language recommendations and reflect the intended meaning of strictly male and female biological sex, this term has been replaced with “women and men” throughout the manuscript and Supplementary Material (Multiple instances).

*In addition to these revisions, you may need to complete some formatting changes, which you will receive in a follow up email. A member of our team will be in touch with a set of requests shortly. If you do not receive a separate email within a few days, please assume that checks have been completed, and no additional changes are required.*

Answer: These instructions have been noted and addressed as indicated.

*When you upload your revision, please include a point-by-point response that addresses all of the reviewer and editorial points, indicating the changes made in the manuscript and either an excerpt of the revised text or the location (eg: page and line number) where each change can be found. Please also be sure to check the general editorial comments at the end of this letter and include these in your point-by-point response. When you resubmit your paper, please include a clean version of the paper as the main article file and a version with changes tracked as a marked-up manuscript. It may also be helpful to check the guidelines for revised papers at* [*http://journals.plos.org/plosmedicine/s/revising-your-manuscript*](http://journals.plos.org/plosmedicine/s/revising-your-manuscript) *for any that apply to your paper.*

Answer: These instructions have been noted and addressed as indicated.

*We ask that you submit your revision by Apr 03 2026 11:59PM. However, if this deadline is not feasible, please contact me by email, and we can discuss a suitable alternative.*

Answer: These instructions have been noted and addressed as indicated.

*Don't hesitate to contact me directly with any questions (*[*afarrell@plos.org*](mailto:afarrell@plos.org)*).*

Answer: We appreciate your time and consideration.

-----------------------------------------------------------

**Comments from the academic editor:**

Comment: We thank the academic editor for the time and effort put into handling this submission, the assessment of our work, and the constructive feedback on our manuscript that enriched it and improved its readability. Please find below a point-by-point reply addressing each of the editorial and reviewers’ comments.

*Between-study heterogeneity is extreme for most pooled estimates (often I² >50% with wide prediction intervals). Yet, the manuscript still emphasizes pooled values (e.g. general population prevalence of 3.2%). Given the heterogeneity, I recommend further de-emphasizing pooled point estimates and placing greater weight on prevalence distributions, population-type gradients, and study-design effects. The meta-regression analyses are informative and explain a large proportion of heterogeneity, identifying coherent epidemiological gradients by population type, age, and subregion.*

Answer: We thank the academic editor for the useful comment. We agree that the substantial between-study heterogeneity warrants cautious interpretation of pooled estimates. Accordingly, the manuscript has been revised throughout to de-emphasize pooled point estimates, explicitly highlight the extent of heterogeneity, clarify its implications for the interpretation of the meta-analyses, and place greater emphasis on the distribution of prevalence estimates and identified gradients (Abstract; Methods, Page 12, Paragraph 4; Results, Page 18, Paragraph 4; Discussion, Page 20, Paragraphs 4-5; and Discussion, Page 24, Paragraph 1).

*The risk-of-bias framework remains conceptually limited. Precision (sample size, inappropriately dichotomized at 200), reporting quality, and bias are partly conflated, and the assessment does not adequately capture the fundamental non-representativeness of many clinic-based and convenience samples. The assumptions underlying the assessment of publication bias are unclear. While inclusion of sampling method and sample size in meta-regression is a strength, the manuscript would benefit from a clearer acknowledgment that many included studies cannot be interpreted as population prevalence estimates, regardless of statistical precision.*

Answer: We thank the academic editor for this thoughtful comment. We agree that the risk-of-bias framework has limitations and does not fully address issues of external validity, including the non-representativeness of clinic-based and convenience samples. These limitations have now been acknowledged and discussed in the manuscript (Discussion, Page 24, Paragraph 4; and Page 25, Paragraph 1).

We also acknowledge that the dichotomization of sample size at 200 is a simplification that does not capture the continuum of statistical precision, and this has now been clarified in the revised text (Discussion, Page 25, Paragraph 2). In addition, we have clarified the assumptions underlying the assessment of publication bias, noting that asymmetry in Doi plots may reflect publication bias or small-study effects, but can also arise from true between-study heterogeneity in epidemiological settings such as this (Methods, Page 11, Paragraph 4; and Discussion, Page 25, Paragraph 3).

*The observed decline in prevalence (~1% per year) may be plausible, but interpretation should be more cautious. Changes in diagnostics, testing practices, healthcare access, and study populations over time may contribute substantially to observed trends. I suggest strengthening the discussion to emphasize that true incidence trends cannot be inferred with certainty from these data.*

Answer: We agree with this comment. The description of the observed trend has been moderated, and this point has been clarified and discussed in the revised manuscript (Discussion, Page 21, Paragraph 1; Discussion, Page 25, Paragraph 4; and Page 26, Paragraph 1).

-----------------------------------------------------------

**Comments from the reviewers:**

Reviewer #1:

*This is an exceptionally well conducted and written study as well as very relevant for understanding the patterns in epidemiology of NG in sub-saharan Africa.*

Comment: We thank the reviewer for the time and effort put into this review, the assessment of our work, and the constructive feedback on our manuscript that enriched it and improved its readability. Please find below a point-by-point reply addressing each of the reviewer’s comments.

*Following are my comments:*

*Under Methods, the section on study selection and eligibility criteria, please specify which specimens were taken into consideration for studies' inclusion.*

Answer: We thank the reviewer for this useful comment. We have now clarified in the Methods the types of specimens considered for study inclusion, as suggested (Methods, Page 8, Paragraph 4). Specifically, we indicate that studies reporting NG detection based on urogenital (e.g., urine, urethral, endocervical, or vaginal), anorectal, and oropharyngeal specimens were eligible (Methods, Page 8, Paragraph 4).

*Under Results, it is more common to use abbreviation IBBS than IBBSS.*

Answer: Indeed, this is the more commonly used abbreviation. The abbreviation has now been revised accordingly (several instances).

*Rows 246-249 of Results: please clarify the criteria for the population groups that prevalence estimates are shown for? Suggest not to group symptomatic men and women with FSW, MSM and STI clinic attendees since they overlap (i.e. there are symptomatic women among FSW). Also, it might be good to show estimates for other groups (clients of FSW, military men, ANC clinic attendees, etc).*

**	Of note is that estimates for other population groups are shown later in Results.*

Answer: We apologize for the confusion. Symptomatic women and men were not grouped with FSW, MSM, or STI clinic attendees. The cited paragraph was intended to describe the volume of evidence across the most frequently represented populations, with each population classified and analyzed separately. Population groups were defined according to primary study classifications and organized into domains, including behavioral or occupational risk groups (e.g., FSW, MSM), clinical presentation groups (e.g., symptomatic individuals), and healthcare-seeking populations (e.g., STI clinic attendees). Definitions of population type classifications are provided in Table 1 (Table 1). This has now been clarified in the revised manuscript (Methods, Page 9, Paragraph 4; Page 10, Paragraph 1; and Results, Page 15, Paragraph 4).

We have also added a table presenting meta-analysis results for select populations within these broader categories, including pregnant women/ANC clinic attendees, clients of FSWs, and military personnel (S8 Table), as suggested, and have cited these estimates in the main text (Results, Page 18, Paragraph 3).

*In the rows 278-280 please add the number of studies for urogenital, anorectal and oropharyngeal prevalence estimates as the latter might include a relatively small number of studies.*

Answer: We apologize for not including these numbers in the text, given the large volume of estimates presented. However, the number of estimates for each population and specimen type is provided in Table 2 (Table 2). This has now been clarified in the revised manuscript, including highlighting the small number of anorectal and oropharyngeal prevalence estimates (Results, Page 17, Paragraph 2).

*Row 298: For serological evidence of ever having had NG infection, please show, if available estimates for MSM.*

Answer: Table 2 presents all estimates for all population groups (Table 2); however, no studies reported serological evidence of ever having had NG infection among MSM.

*Discussion*

*Row 382: Due to the highest prevalence in persons younger than 25 years, it might be beneficial to highlight this population group (particularly from KPs and vulnerable groups) as in need of more intensified prevention and testing.*

Answer: Excellent suggestion, thank you. This point has now been highlighted in the Discussion (Discussion, Page 23, Paragraph 2).

Reviewer #2:

*Congratulations to the authors on this large and ambitious systematic review and meta-analysis synthesizing 950 publications, 1,604 prevalence measures, and data from approximately 2.7 million individuals across 1964-2025. The authors estimate a pooled urogenital prevalence of 3.2% in general populations, identify an approximate 1% annual decline, and demonstrate a clear risk gradient across population groups (e.g., higher prevalence among symptomatic men, female sex workers, and anorectal/oropharyngeal infections among MSM). The manuscript is well written and methodologically thoughtful. However, several issues related to transparency, methodological clarity and interpretability, if addressed, would make the manuscript most suitable for publication.*

Comment: We thank the reviewer for the time and effort put into this review, the assessment of our work, and the constructive feedback on our manuscript that enriched it and improved its readability. Please find below a point-by-point reply addressing each of the reviewer’s comments.

*Major comments*

*1. Study protocol*

**	Please upload the full search and analysis protocol as Supplementary Material and clearly describe all deviations or adaptations relative to any previously published protocol.*

Answer: The implemented study protocol was adapted from a previously published protocol [2] and further refined using methodological frameworks applied in prior systematic reviews of NG and other STI prevalence, including those conducted for sub-Saharan Africa [3-12]. Accordingly, no substantive deviations or methodological modifications were introduced relative to previous regional systematic reviews of NG prevalence [4-7], as a consistent methodological approach was applied. Only minor adaptations were made to the definition of population group categories to reflect region-specific contexts (Table 1). This point has now been clarified in the revised manuscript (Methods, Page 6, Paragraph 4; Page 7, Paragraph 1; and Table 1).

However, to address the reviewer’s comment, the study protocol has now been formally registered with PROSPERO, and the registration details have been included in the revised manuscript (Methods, Page 6, Paragraph 4).

Furthermore, detailed descriptions of the study methods are provided in the Supplementary Material. The Methods are supported by several supplementary inserts that offer operational and analytical specifications. S2 Table presents the full database search strategies across all database sources, ensuring transparency and reproducibility (S2 Table). S1 Box defines the geographical scope of sub-Saharan Africa and its subregional classification (S1 Box). S2 Box summarizes all variables extracted from eligible studies, including outcomes and covariates used in the analyses (S2 Box). S3 Box lists the predefined factors included in the meta-regression analyses (S3 Box). S3 Table details the quality assessment framework applied to prevalence studies, including criteria for precision and risk of bias (S3 Table). Together, these supplementary materials provide a comprehensive specification of the study design, data extraction process, and analytical framework underpinning the review.

*2. Definition of prevalence*

**	The manuscript does not clearly define "prevalence": e.g. whether it refers to point prevalence, test positivity, or another measure.*

Answer: Thank you for this excellent point. This point has now been clarified in the revised Methods (Methods, Page 7, Paragraph 4).

**	Please specify the time frame, numerator/denominator, and rules used when multiple anatomical sites were collected from the same participant.*

Answer: We have now explicitly clarified these aspects in the revised Methods (Methods, Page 7, Paragraph 4; and Methods, Page 10, Paragraph 2).

**	The search strategy (Table S2) contains pathogen, disease, and country terms but no explicit prevalence related terms. Please clarify how your operational definition of prevalence informed study identification.*

Answer: Thank you for this pertinent observation. The search strategy was intentionally designed to maximize sensitivity by focusing on NG-related terms (pathogen and disease) and geographic identifiers, without restricting by outcome-specific terms such as “prevalence.” This approach aimed to avoid missing relevant studies that reported prevalence estimates but were not indexed or described using explicit prevalence-related terminology. Accordingly, the operational definition of prevalence was applied during the screening and data extraction stages rather than within the search strategy. Specifically, studies were eligible if they reported sufficient data to compute prevalence. This point has now been clarified in the revised Methods (Methods, Page 7, Paragraph 3).

*3. General population estimate*

**	The reported 3.2% pooled estimate combines heterogeneous population groups (e.g., ANC attendees, blood donors, community based samples).*

Answer: Indeed, this is correct. We agree that the subpopulation structure and substantial between-study heterogeneity warrant cautious interpretation of pooled estimates. Accordingly, the manuscript has been revised throughout to de-emphasize pooled point estimates, explicitly highlight the extent of heterogeneity, clarify its implications for the interpretation of the meta-analyses, and place greater emphasis on the distribution of prevalence estimates and identified gradients (Abstract; Methods, Page 12, Paragraph 4; Results, Page 18, Paragraph 4; Discussion, Page 20, Paragraphs 4-5; and Discussion, Page 24, Paragraph 1).

We have also added a table presenting meta-analysis results for select relevant subpopulations within the broader population categories, including pregnant women/ANC clinic attendees, clients of FSWs, and military personnel, where sufficient measures were available to justify meta-analyses (S8 Table). These estimates have been cited in the main text (Results, Page 18, Paragraph 3). No study was identified that reported prevalence among blood donors.

**	To support interpretability, please consider providing sub pooled estimates for these groups separately and conduct a sensitivity analysis restricted to probability based samples to better approximate a representative population prevalence.*

Answer: Thank you for this useful suggestion. We have added a table presenting meta-analysis results for select subpopulations within the broader general population category (S8 Table), as suggested, and have cited these estimates in the main text (Results, Page 18, Paragraph 3).

We have also added a table presenting meta-analysis results separately for studies using probability-based and non-probability sampling methods (S9 Table; and Results, Page 18, Paragraph 3).

*4. Heterogeneity and small study effects*

**	Many pooled estimates display high heterogeneity (I²), wide prediction intervals and a pronounced small study effect.*

Answer: We agree that the substantial between-study heterogeneity warrants cautious interpretation of pooled estimates. Accordingly, the manuscript has been revised throughout to de-emphasize pooled point estimates, explicitly highlight the extent of heterogeneity, clarify its implications for the interpretation of the meta-analyses, and place greater emphasis on the distribution of prevalence estimates and identified gradients (Abstract; Methods, Page 12, Paragraph 4; Results, Page 18, Paragraph 4; Discussion, Page 20, Paragraphs 4-5; and Discussion, Page 24, Paragraph 1). The discussion of the small-study effect has also been clarified and expanded in the revised manuscript (Methods, Page 11, Paragraph 4; Discussion, Page 24, Paragraph 2; and Discussion, Page 25, Paragraph 2).

**	Please include prediction intervals prominently in the main Results section and also in the Abstract.*

Answer: Prediction intervals have now been highlighted, as suggested (Abstract; and Results, Page 18, Paragraph 4).

*5. Diagnostic methods over six decades*

**	The evidence base spans multiple decades and diagnostic modalities (culture, Gram stain, NAATs).*

**	Please provide assay specific pooled estimates (e.g., NAAT only analyses) and discuss how assay evolution may have influenced prevalence estimates.*

Answer: We address these two comments together, as they are closely related. The potential impact of the evolution of diagnostic methods over time has now been further discussed (Discussion, Page 24, Paragraphs 2-3; Discussion, Page 25, Paragraph 4; and Page 26, Paragraph 1). Pooled mean prevalence of NG infection by assay type is provided in S7 Table (S7 Table).

**	Relatedly, consider elaborating on potential spectrum bias between facility and community based samples, given differences in symptom status and risk profiles.*

Answer: We have now expanded the discussion of these spectrum effects in the revised manuscript (Discussion, Page 24, Paragraph 3).

*6. Temporal trend presentation*

**	The 1% annual decline is an important finding but appears only in tables.*

**	Please incorporate this result into the main Results text with a clear trend figure (based on publication year and, in sensitivity analyses, year of data collection). If data allow, presenting subregional trends would be very informative.*

Answer: We address these two comments together, as they are closely related. The description and discussion of this finding have now been expanded in the revised manuscript (Results, Page 20, Paragraph 1; Discussion, Page 21, Paragraph 1; Discussion, Page 25, Paragraph 4; and Page 26, Paragraph 1). We have also added two figures illustrating temporal trends for the overall region and by subregion, as suggested. Specifically, S2 Fig shows temporal trends in NG prevalence estimates across all population groups, indicating a declining trend over time (S2 Fig). S3 Fig presents subregion-specific trends within sub-Saharan Africa, similarly indicating a declining trend over time across all subregions (S2 Fig). These figures have also been cited in the main text (Results, Page 15, Paragraph 5).

*7. Geographic scope*

**	The title refers to sub Saharan Africa, yet Mauritania (a North African country) is included in the dataset.*

**	Please justify this inclusion or adjust wording and analytical framing accordingly.*

Answer: We address these two comments together, as they are closely related. We appreciate this point and agree that Mauritania is often considered part of North Africa. However, the definition of sub-Saharan Africa in this study follows conventions applied in previous STI research [8, 9] and aligns with regional classifications used by the WHO Regional Office for Africa and UNAIDS (Methods, Page 8, Paragraph 1). Mauritania is classified by WHO within the African Region rather than the Eastern Mediterranean Region. This point has now been clarified in the revised Methods (Methods, Page 8, Paragraph 1). Notably, only two studies were conducted in this country, and therefore this classification has no material impact on the study results or findings.

*8. Comparability with recent syntheses*

**	Please add a short comparison with other recent SSA prevalence estimates, explaining how differences in inclusion criteria, population groups, time periods, and diagnostic methods may account for the higher pooled values observed here.*

Answer: Considering comments from the editors and reviewers, the manuscript has been revised to de-emphasize pooled point estimates and place greater emphasis on the distribution of prevalence estimates and identified gradients (Abstract; Methods, Page 12, Paragraph 4; Results, Page 18, Paragraph 4; Discussion, Page 20, Paragraphs 4-5; and Discussion, Page 24, Paragraph 1). Accordingly, we have avoided direct comparisons with other estimates, although other estimates are cited where pooled results are discussed (Discussion, Page 20, Paragraph 5).

*9. Policy implications*

*The discussion could be strengthened by further elaborating on the policy and programmatic implications, particularly for screening strategies, partner notification, and antimicrobial resistance (AMR) surveillance. The authors may also consider positioning their findings in relation to the 2025 WHO guidance on the management of asymptomatic NG/CT, which outlines prevalence thresholds for screening among young people and pregnant women.*

Answer: Thank you for this excellent suggestion. The Discussion has been expanded to address these points and to cite relevant WHO guidance (Discussion, Page 23, Paragraphs 2-3).

*Minor comments*

**	Search strategy: Please provide complete reproducible search strings (Boolean operators, platforms/hosts, final search date of 4 June 2025) and database specific start dates. Clarify language coverage, including whether French language publications were captured.*

Answer: Full and exact search strategies for all databases are provided in S2 Table (S2 Table), and all searches were conducted from inception. This has now been clarified in the Methods (Methods, Page 7, Paragraph 2).

The search was not restricted to English-language publications and was conducted without language restrictions. Where necessary, titles, abstracts, and full texts were translated using Google Translate to facilitate screening and data extraction of non-English articles. This has now been clarified in the Methods (Methods, Page 7, Paragraph 2).

**	Risk of bias: Specify the exact risk of bias domains used, provide a per study risk of bias summary table in the Supplement. Alternatively, explain why this was not presented.*

Answer: The risk-of-bias domains are described in the Methods (Methods, Page 10, Paragraph 5; and Page 11, Paragraphs 1-4) and detailed in S3 Table (S3 Table). Summary table of the risk-of-bias assessment is provided in S5 Table (S5 Table).

**	Figures/Tables: Consider adding a bubble plot for the meta regression (bubble size = sample size; colour = population type) and a map of country level data coverage. Please include prediction intervals wherever pooled prevalence estimates are presented.*

Answer: We explored the inclusion of bubble plots; however, these did not add substantive value in the context of the meta-regression analyses. The models were multivariable, incorporating multiple covariates simultaneously, whereas bubble plots are better suited to illustrating univariable associations. Accordingly, such plots would not adequately represent the adjusted relationships estimated in the models. In addition, the large number of studies, covariates, and population strata would render graphical displays complex and difficult to interpret. The tabular presentation (Tables 3 and 4) was therefore retained as the clearest, most comprehensive, and most direct approach for conveying the meta-regression findings.

Including prediction intervals alongside all pooled estimates is challenging given the large number of pooled estimates and the large overall manuscript length. However, prediction intervals have now been highlighted, as suggested (Abstract; and Results, Page 18, Paragraph 4). In addition, all prediction intervals are reported in Table 2 and are accessible to readers (Table 2).

Thank you for the helpful suggestion. A map of country-level data coverage has now been added, as recommended, and cited in the main text (S1 Fig; and Results, Page 15, Paragraph 3).

Reviewer #3:

Comment: We thank the reviewer for the time and effort put into this review, the assessment of our work, and the constructive feedback on our manuscript that enriched it and improved its readability. Please find below a point-by-point reply addressing each of the reviewer’s comments.

*ABSTRACT*

*It would be ideal to introduce 95%CI at first use.*

Answer: This has been added, as suggested (Abstract).

*The conclusion (higher than other regions) doesn't flow without having presented prevalence in other regions in Background.*

Answer: We agree with the reviewer. This has been revised as suggested (Abstract).

*Similarly, WHO target should be introduced in Background before mention in Conclusions.*

Answer: This was challenging due to the abstract word count limit; to address the reviewer’s comment, the WHO target has been removed from the abstract (Abstract).

*Some editorialising of group-by-group prevalences would allow more words if needed.*

Answer: We appreciate this suggestion. However, given the objectives of the study, we aimed to retain explicit group-specific prevalence estimates in the abstract to accurately reflect the distribution of the evidence. While editorializing could reduce word count, it would come at the expense of specificity and may obscure important differences between population groups. We therefore opted to preserve a concise set of key estimates while ensuring the abstract remains within the required word limit.

*INTRODUCTION*

*'A key strength...' line 85: this paragraph is Discussion rather than Introduction & should be moved or deleted.*

Answer: This paragraph has been removed, as suggested (Introduction, Page 6, Paragraph 2).

*METHODS*

*'not formally registered with PROSPERO' -> just 'not registered with PROSPERO': there is no notion of formal vs informal or other type of registration.*

Answer: The study protocol has now been formally registered with PROSPERO, and the registration details have been included in the revised manuscript (Methods, Page 6, Paragraph 4).

*search strategy: the supplement notwithstanding, this section should convey some sort of summary of the search undertaken, e.g. what combination of concepts was sought?*

Answer: Excellent suggestion, thank you. This has now been added as suggested (Methods, Page 7, Paragraph 3).

*Precision categorization, line 165: I don't understand the rationale for categorization of precision given that study CIs provide a continuous direct quantification of precision. Moreover, using study count to categorize precision may be problematic for structured survey samples where N is not the only determinant of precision.*

Answer: We thank the reviewer for this useful comment. We agree that statistical precision is inherently a continuous construct, more appropriately reflected by study-specific CIs, and that sample size alone does not fully capture precision, particularly in structured survey designs where clustering and weighting influence variance. However, the use of a dichotomized sample size threshold of 200 was adopted as a pragmatic proxy to facilitate comparability across a large and heterogeneous set of studies and to assess small-study effects, a recognized source of bias in STI research. We acknowledge that this approach represents a simplification and does not account for complex sampling designs, and this limitation has now been clarified in the revised manuscript (Discussion, Page 25, Paragraph 2).

*Without a protocol, I think it's important to state somewhere which (if any) elements of the statistical analysis were prespecified (e.g. choice of groups, p-value cut-offs), or to state that the analyses were not prespecified (if this is true).*

Answer: All elements of the statistical analysis were prespecified based on previously published systematic reviews [3-12]. This has now been clarified in the Methods (Methods, Page 6, Paragraph 4; and Page 7, Paragraph 1).

*Meta-analyses & Meta-regression: I don't think it is explicitly stated what measure of precision was associated with each individual data-point, and how this was derived from the data (e.g. SD estimate from CIs?). This should be done separately in each section.*

Answer: We thank the reviewer for this comment and agree that the measure of precision associated with each data point should be specified. Study-specific standard errors were derived directly from the number of events (or the effective number of events for studies with complex sampling designs) and sample size under the selected transformation, rather than being back-calculated from reported CIs. These standard errors on the transformed scale were then used for inverse-variance weighting in the meta-analyses and in the meta-regression models within a random-effects framework. This has now been explicitly clarified in the revised Methods (Methods, Page 12, Paragraph 2).

*Meta-regression: please state fully what structure was represented via random effects.*

Answer: The meta-regression models were specified within a normally distributed random-effects framework, in which the random-effects term captures residual between-study heterogeneity not explained by the included covariates, thereby allowing for variation in true underlying prevalence across studies beyond sampling error. This has now been clarified in the revised Methods (Methods, Page 13, Paragraph 4).

*RESULTS*

*lines 224-231: Fewer details are needed in words in my opinion, given they are all on Figure 1.*

Answer: This paragraph has been streamlined as suggested by removing some numerical details while retaining the key information needed to convey the study selection process and sources of data (Results, Page 14, Paragraph 4).

*Now I see how low/high 'precision' categories were used, I think it is mainly a terminological issue. I would just avoid the word precision, e.g. by calling the studies small or not small.*

Answer: Thank you for this thoughtful comment. We agree that this is mainly a matter of terminology. However, revising the terminology presents challenges, as it has been used consistently across numerous prior STI/HIV prevalence systematic reviews. Changing it here could introduce confusion and reduce comparability, particularly given the emphasis on methodological consistency across these studies. Nevertheless, as noted in our response to a related comment above, we have clarified the rationale and discussed this point in the revised manuscript (Discussion, Page 25, Paragraph 2).

*Introduce '95% CI' at first use.*

Answer: The 95% CI acronym has been defined at its first use, which occurred in the Methods (Methods, Page 12, Paragraph 1).

*DISCISSION*

*Opening paragraph: of course Africa is also a notably young region and NG has higher prevalence among the young. I think it would be important to briefly consider age-specific/standardized comparisons to assure readers this is comparatively high prevalence is not just due to different demography.*

Answer: Excellent point, thank you. We agree with the reviewer. A standardized comparison is effectively already provided in the meta-regression analyses, which assessed the effect of age on prevalence after adjustment for other covariates and potential confounders and confirmed higher prevalence among individuals younger than 25 years (Table 3; Table 4; and Results, Page 19, Paragraph 3). This point has now also been highlighted in the Discussion (Discussion, Page 20, Paragraph 5).

*FIGURE 1*

*There seems to be a missing 'Records eligible for inclusion' box on the database half of the diagram.*

Answer: We appreciate the reviewer’s comment and agree that adding this box enhances clarity. The PRISMA flow diagram follows the PRISMA 2020 reporting standard [13], which does not include a separate “records eligible for inclusion” box in the main flowchart. However, we have now added this box to improve clarity and flow (Fig 1).

*What does 'marked as ineligible by automation tools' mean?*

Answer: As indicated above, we have used the terminology and diagram structure in accordance with the PRISMA 2020 reporting standard **[13]**. The term “marked as ineligible by automation tools” refers to records excluded during the initial screening process using automated or semi-automated tools (e.g., screening platforms) based on predefined criteria, such as non-relevant keywords or clearly ineligible study types. In this study, title and abstract screening were conducted manually, and no automation tools were used. This term has now been explained in a footnote to the figure (Fig 1).

*FIGURE SUGGESTION*

*I think a map showing where the data came from (e.g. point size or count as text) potentially with choropleth encoding of the most commonly-reported population prevalence would be really useful. If the authors don't want to add more exhibits, the table defining risk groups could be moved to the supplement.*

*Africa is such a huge and varied place, I do think it is important to convey where the data are from, and any variation across countries better than is currently done.*

Answer: We address these two comments together, as they are closely related. We thank the reviewer for this helpful suggestion. We agree that a map illustrating the geographical distribution of data is valuable, and we have therefore added a figure showing country-level data coverage, including the number of prevalence measures contributed by each country, and cited it in the main text (S1 Fig; Results, Page 15, Paragraph 3).

However, we are inclined not to include choropleth encoding of prevalence. Given the diversity of population types studied and the marked variation in prevalence across these groups, together with substantial between-study heterogeneity and differences in year of study, age structure, and study methods, assigning a single summary prevalence value to each country would be difficult to interpret and potentially misleading. Instead, the study emphasizes the distribution of estimates and gradients across population groups, which provides a more meaningful representation of the epidemiological patterns.

*CODE & DATA AVAILABILITY*

*I didn't see the data and code from this study were made available publicly. This would be useful and enhance transparency and reproducibility.*

Answer: The study used existing publicly available statistical packages, specifically the *meta* package for meta-analyses and the *metareg* package for meta-regression analyses; therefore, no custom code was generated or required for reporting. The data were extracted from published literature, and relevant summary data are presented in both the manuscript and the Supplementary Material. The data sharing statement has now been amended to clarify these points (Data availability statement).

Notably, the database of extracted prevalence measures will also be integrated into the WHO STI Prevalence Atlas, a Gates Foundation–funded WHO project currently under development to provide a publicly accessible database of STI prevalence data across world regions.

*Any attachments provided with reviews can be seen via the following link:*

Answer: No link was provided, and the reviews from all three reviewers were included in the original decision email.

*------------------------------------------------------------*

**General editorial requests:**

*Please check each item carefully and indicate where addressed.*

**	We ask every co-author listed on the manuscript to fill in a contributing author statement, making sure to declare all competing interests. If any of the co-authors have not filled in the statement, we will remind them to do so when the paper is revised. If all statements are not completed in a timely fashion this could hold up the re-review process. If new competing interests are declared later in the revision process, this may also hold up the submission. Should there be a problem getting one of your co-authors to fill in a statement we will be in contact. Please do not add or remove authors without first discussing this with the handling editor. You can see our competing interests policy here:* [*http://journals.plos.org/plosmedicine/s/competing-interests*](http://journals.plos.org/plosmedicine/s/competing-interests)*.*

Answer: These instructions have been noted and addressed as indicated.

**	Please upload any figures associated with your paper as individual TIF or EPS files with 300dpi resolution at resubmission; please read our figure guidelines for more information on our requirements:* [*http://journals.plos.org/plosmedicine/s/figures*](http://journals.plos.org/plosmedicine/s/figures)*. While revising your submission, we strongly recommend that you use PLOS's NAAS tool (*[*https://ngplosjournals.pagemajik.ai/artanalysis*](https://ngplosjournals.pagemajik.ai/artanalysis)*) to test your figure files. NAAS can convert your figure files to the TIFF file type and meet basic requirements (such as print size, resolution), or provide you with a report on issues that do not meet our requirements and that NAAS cannot fix.*

*After uploading your figures to PLOS's NAAS tool -* [*https://ngplosjournals.pagemajik.ai/artanalysis*](https://ngplosjournals.pagemajik.ai/artanalysis)*, NAAS will process the files provided and display the results in the "Uploaded Files" section of the page as the processing is complete.*

*If the uploaded figures meet our requirements (or NAAS is able to fix the files to meet our requirements), the figure will be marked as "fixed" above. If NAAS is unable to fix the files, a red "failed" label will appear above.*

*When NAAS has confirmed that the figure files meet our requirements, please download the file via the download option, and include these NAAS processed figure files when submitting your revised manuscript.*

Answer: These instructions have been noted and addressed as indicated.

**	Please ensure that the paper adheres to the PLOS Data Availability Policy (see* [*http://journals.plos.org/plosmedicine/s/data-availability*](http://journals.plos.org/plosmedicine/s/data-availability)*), which requires that all data underlying the study's findings be provided in a repository or as Supporting Information. For data residing with a third party, authors are required to provide instructions with contact information (web or email address) for obtaining the data. Please note that a study author cannot be the contact person for the data. PLOS journals do not allow statements supported by "data not shown" or "unpublished results." For such statements, authors must provide supporting data or cite public sources that include it.*

Answer: The data for this study were extracted from published literature. The analyses of data are presented in the main manuscript and the Supplementary Material (Data availability statement).

**	We expect all researchers with submissions to PLOS in which author-generated code underpins the findings in the manuscript to make all author-generated code available without restrictions upon publication of the work. In cases where code is central to the manuscript, we may require the code to be made available as a condition of publication. Authors are responsible for ensuring that the code is reusable and well documented. Please make any custom code available, either as part of your data deposition or as a supplementary file. Please add a sentence to your data availability statement regarding any code used in the study, e.g. "The code used in the analysis is available from Github [URL] and archived in Zenodo [DOI link]" Please review our guidelines at* [*https://journals.plos.org/plosmedicine/s/materials-software-and-code-sharing*](https://journals.plos.org/plosmedicine/s/materials-software-and-code-sharing) *and ensure that your code is shared in a way that follows best practice and facilitates reproducibility and reuse. Because Github depositions can be readily changed or deleted, we encourage you to make a permanent DOI'd copy (e.g. in Zenodo) and provide the URL.*

Answer: The study used existing publicly available statistical packages, specifically the *meta* package for meta-analyses and the *metareg* package for meta-regression analyses; therefore, no custom code was generated or required for reporting. The data sharing statement has now been amended to clarify these points (Data availability statement).

**	Please ensure that the study is reported according to the PRISMA guideline and include the completed PRISMA checklist as Supporting Information. When completing the checklist, please use section and paragraph numbers, rather than page numbers. Please add the following statement, or similar, to the Methods: "This study is reported as per PRISMA guideline (S1 Checklist)."*

Answer: These instructions have been noted and addressed as indicated (S1 Table. PRISMA checklist in Supplementary Material; and Methods, Page 6, Paragraph 3).

*FORMATTING - GENERAL*

**	Abstract: Please structure your abstract using the PLOS Medicine headings (Background, Methods and Findings, Conclusions). Please combine the Methods and Findings sections into one section.*

Answer: These instructions have been noted and addressed as indicated (Abstract).

**	At this stage, we ask that you include a short, non-technical Author Summary of your research to make findings accessible to a wide audience that includes both scientists and non-scientists. The Author Summary should immediately follow the Abstract in your revised manuscript. This text is subject to editorial change and should be distinct from the scientific abstract. Ideally each sub-heading should contain 2-3 single sentence, concise bullet points containing the most salient points from your study. In the final bullet point of 'What Do These Findings Mean?', please include the main limitations of the study in non-technical language. Please see our author guidelines for more information:* [*https://journals.plos.org/plosmedicine/s/revising-your-manuscript#loc-author-summary*](https://journals.plos.org/plosmedicine/s/revising-your-manuscript#loc-author-summary)*.*

Answer: These instructions have been noted and addressed as indicated (Author summary).

**	Please express the main results with 95% CIs as well as p values. When reporting p values please report as p<0.001 and where higher as the exact p value p=0.002, for example. Throughout, suggest reporting statistical information as follows to improve clarity for the reader "22% (95% CI [13%,28%]; p</=)". Please be sure to define all numerical values at first use.*

Answer: These instructions have been noted and addressed as indicated.

**	Please include page numbers and line numbers in the manuscript file. Use continuous line numbers (do not restart the numbering on each page).*

Answer: These instructions have been noted and addressed as indicated.

**	Please cite the reference numbers in square brackets. Citations should precede punctuation.*

Answer: These instructions have been noted and addressed as indicated. Please note that reference formatting was done using the EndNote style for PLOS journals.

*FIGURES AND TABLES*

**	Please provide titles and legends for all figures and tables (including those in Supporting Information files).*

Answer: These instructions have been noted and addressed as indicated.

**	Please define all abbreviations used in each figure/table (including those in Supporting Information files).*

Answer: These instructions have been noted and addressed as indicated.

**	Please consider avoiding the use of red and green in order to make your figure more accessible to those with color blindness.*

Answer: These instructions have been noted and addressed as indicated for all figures.

*SUPPLEMENTARY MATERIAL*

**	Please note that supplementary material will be posted as supplied by the authors. Therefore, please amend it according to the relevant comments outlined here.*

Answer: These instructions have been noted and addressed as indicated.

**	Please cite your Supporting Information as outlined here:* [*https://journals.plos.org/plosmedicine/s/supporting-information*](https://journals.plos.org/plosmedicine/s/supporting-information)

Answer: These instructions have been noted and addressed as indicated.

*REFERENCES*

**	PLOS uses the numbered citation (citation-sequence) method and first six authors, et al.*

Answer: These instructions have been noted and addressed as indicated. Please note that reference formatting was done using the EndNote style for PLOS journals.

**	Please ensure that journal name abbreviations match those found in the National Center for Biotechnology Information (NCBI) databases (*[*http://www.ncbi.nlm.nih.gov/nlmcatalog/journals*](http://www.ncbi.nlm.nih.gov/nlmcatalog/journals)*), and are appropriately formatted and capitalised.*

Answer: These instructions have been noted and addressed as indicated.

**	Where website addresses are cited, please include the complete URL and specify the date of access (e.g. [accessed: 12/06/2023]).*

Answer: These instructions have been noted and addressed as indicated.

**	Please also see* [*https://journals.plos.org/plosmedicine/s/submission-guidelines#loc-references*](https://journals.plos.org/plosmedicine/s/submission-guidelines#loc-references) *for further details on reference formatting.*

Answer: These instructions have been noted and addressed as indicated. Please note that reference formatting was done using the EndNote style for PLOS journals.

**	[FOR POPULATION HEALTH ESTIMATES] If pertinent, please ensure that the study is reported according to the GATHER statement (available from* [*https://www.equator-network.org/reporting-guidelines/gather-statement*](https://www.equator-network.org/reporting-guidelines/gather-statement)*) and include the completed checklist as Supporting Information. Please add the following statement, or similar, to the Methods: "This study is reported as per the Guidelines for Accurate and Transparent Health Estimates Reporting (GATHER) statement (S1 Checklist)." When completing the checklist, please use section and paragraph numbers, rather than page numbers.*

Answer: This does not apply to this study.

**	Please state in the Methods section whether the study had a prospective protocol or analysis plan. If a prospective analysis plan (from your funding proposal, IRB or other ethics committee submission, study protocol, or other planning document written before analyzing the data) was used in designing the study, please include the relevant document(s) with your revised manuscript as a Supporting Information file to be published alongside your study and cite it in the Methods section. A legend for this file should be included at the end of your manuscript. If no such document exists, please make sure that the Methods section transparently describes when analyses were planned, and when/why any data-driven changes to analyses took place. Changes in the analysis, including those made in response to peer review comments, should be identified as such in the Methods section of the paper, with rationale.*

Answer: This point is addressed and clarified in the Methods (Methods, Page 6, Paragraph 4; and Page 7, Paragraph 1).

*SYSTEMATIC REVIEWS & META-ANALYSES*

**	Please report your SR/MA according to the PRISMA guidelines provided at the EQUATOR site.* [*http://www.equator-network.org/reporting-guidelines/prisma/*](http://www.equator-network.org/reporting-guidelines/prisma/)*. Please provide the completed PRISMA checklist as Supporting Information. When completing the checklist, please use section and paragraph numbers, rather than page numbers. Please add the following statement, or similar, to the Methods: "This study is reported as per the Preferred Reporting Items for Systematic Reviews and Meta-Analyses (PRISMA) guideline (S1 Checklist)."*

Answer: These instructions have been noted and addressed as indicated (S1 Table. PRISMA checklist in Supplementary Material; and Methods, Page 6, Paragraph 3).

**	Abstract: Please report your abstract according to PRISMA for abstracts (*[*https://doi.org/10.1371/journal.pmed.1001419*](https://doi.org/10.1371/journal.pmed.1001419)*) following the PLOS Medicine abstract structure (Background, Methods and Findings, Conclusions). Please ensure you provide dates of search, data sources, number of studies included, types of study designs included, eligibility criteria, and synthesis/appraisal methods.*

Answer: These instructions have been noted and addressed as indicated and relevant, while also ensuring that the abstract remains within appropriate word count limits (Abstract).

**	Please note that we expect searches to be updated to within 6 months of the time of submission.*

Answer: We appreciate this request. However, implementing it is difficult given the scope and complexity of the study. This review represents a very large-scale effort, including over 1,600 prevalence measures, substantially exceeding the size of a typical systematic review. The volume of data extracted is more than an order of magnitude greater than average. The search has already been updated twice (Methods, Page 7, Paragraph 2), in part reflecting the substantial time required to complete analyses following each update.

Updating the search at this stage would require several weeks for screening and data extraction, followed by a complete re-analysis of the dataset. Even the addition of a single study would necessitate re-running all analyses and updating all tables, figures, and supplementary materials. Given the scale of the study, the complexity of the methodology, the number of outcomes assessed, and the need for rigorous validation, these analyses would require at least several additional weeks to complete, including detailed checks to ensure accuracy across all outputs and the large number of inserts presented in both the manuscript and supplementary files. Therefore, this would result in a substantial delay in the revision and resubmission of the manuscript.

At the time of the original submission, the search was updated within approximately 6 months, consistent with best practices for systematic reviews and compliant with *PLOS Medicine* requirements. Importantly, any further update is unlikely to materially affect the findings. The dataset spans a broad time horizon (1964–2025), and extending the search by a few additional months within a body of evidence covering more than 60 years is very unlikely to meaningfully alter the estimates or conclusions. The evidence base is large, stable, and unlikely to have undergone changes over such a short period that would affect the study outcomes.

Finally, this study is epidemiological in nature and provides population-level evidence that does not change rapidly over short timeframes. It does not also evaluate interventions, compare management strategies, or provide clinical decision-making, where newly emerging evidence could alter treatment recommendations or clinical guidelines.

Given these considerations, updating the search to within 6 months of resubmission would entail substantial additional work without a commensurate benefit to the validity or interpretation of the findings.

To address this comment, this point has now been noted in the Discussion (Discussion, Page 23, Paragraph 4).

***Journal Requirements:***

*1) We have noticed that you have uploaded Supporting Information files, but you have not included a list of legends. Please add a full list of legends for your Supporting Information files after the references list.*

Answer: These instructions have been noted and addressed as indicated.

**References**

1.	Guyatt G, Oxman AD, Akl EA, Kunz R, Vist G, Brozek J, et al. GRADE guidelines: 1. Introduction-GRADE evidence profiles and summary of findings tables. J Clin Epidemiol. 2011;64(4):383-94. Epub 2011/01/05. doi: 10.1016/j.jclinepi.2010.04.026. PubMed PMID: 21195583.

2.	Chemaitelly H, Harfouche M, Blondeel K, Matsaseng TC, Kiarie J, Toskin I, et al. Global epidemiology of Neisseria gonorrhoeae in infertile populations: protocol for a systematic review. BMJ Open. 2019;9(5):e025808. Epub 2019/05/28. doi: 10.1136/bmjopen-2018-025808. PubMed PMID: 31122971; PubMed Central PMCID: PMCPMC6538097.

3.	Chemaitelly H, Majed A, Abu-Hijleh F, Blondeel K, Matsaseng TC, Kiarie J, et al. Global epidemiology of Neisseria gonorrhoeae in infertile populations: systematic review, meta-analysis and metaregression. Sex Transm Infect. 2021;97(2):157-69. Epub 2020/05/20. doi: 10.1136/sextrans-2020-054515. PubMed PMID: 32423944; PubMed Central PMCID: PMCPMC7892374.

4.	Chidiac O, AlMukdad S, Harfouche M, Harding-Esch E, Abu-Raddad LJ. Epidemiology of gonorrhoea: systematic review, meta-analyses, and meta-regressions, World Health Organization European Region, 1949 to 2021. Euro Surveill. 2024;29(9). Epub 2024/03/01. doi: 10.2807/1560-7917.ES.2024.29.9.2300226. PubMed PMID: 38426239; PubMed Central PMCID: PMCPMC10986664.

5.	Chemaitelly H, Harfouche M, Smolak A, Ageeb R, Mohamoud YA, Alaama AS, et al. Epidemiology of gonorrhea in countries of the Middle East and North Africa: systematic review, meta analyses, and meta regressions. BMC Global and Public Health. 2024;2(1):56. doi: 10.1186/s44263-024-00088-9.

6.	Ageeb RA, Almukdad S, Alalami H, Harfouche M, Abu-Raddad LJ. Epidemiology of Neisseria gonorrhoeae in South-East Asia: Systematic review, meta-analyses, and meta-regressions. J Infect. 2025;91(2):106545. Epub 2025/07/01. doi: 10.1016/j.jinf.2025.106545. PubMed PMID: 40581330.

7.	Harfouche M, Khan A, Taha A, El-Khoury R, Ageeb RA, Abu-Raddad LJ. Prevalence and epidemiological patterns of Neisseria gonorrhoeae infection in Canada, 1969-2025: a systematic review and meta-analysis. BMC Public Health. 2026. Epub 2026/03/04. doi: 10.1186/s12889-026-26579-y. PubMed PMID: 41776523.

8.	Harfouche M, Abu-Hijleh FM, James C, Looker KJ, Abu-Raddad LJ. Epidemiology of herpes simplex virus type 2 in sub-Saharan Africa: Systematic review, meta-analyses, and meta-regressions. EClinicalMedicine. 2021;35:100876. Epub 2021/05/25. doi: 10.1016/j.eclinm.2021.100876. PubMed PMID: 34027335; PubMed Central PMCID: PMCPMC8129943.

9.	Harfouche M, Chemaitelly H, Abu-Raddad LJ. Herpes simplex virus type 1 epidemiology in Africa: Systematic review, meta-analyses, and meta-regressions. J Infect. 2019;79(4):289-99. doi: 10.1016/j.jinf.2019.07.012. PubMed PMID: 31376458.

10.	Harfouche M, Gherbi WS, Alareeki A, Alaama AS, Hermez JG, Smolak A, et al. Epidemiology of Trichomonas vaginalis infection in the Middle East and North Africa: systematic review, meta-analyses, and meta-regressions. EBioMedicine. 2024;106:105250. Epub 2024/07/19. doi: 10.1016/j.ebiom.2024.105250. PubMed PMID: 39024899; PubMed Central PMCID: PMCPMC11286989.

11.	El-Jamal M, Annan B, Al Tawil A, Hamati M, Almukdad S, Fakih I, et al. Syphilis infection prevalence in the Middle East and North Africa: a systematic review and meta-analysis. EClinicalMedicine. 2024;75:102746. Epub 2025/01/07. doi: 10.1016/j.eclinm.2024.102746. PubMed PMID: 39763595; PubMed Central PMCID: PMCPMC11701444.

12.	Smolak A, Chemaitelly H, Hermez JG, Low N, Abu-Raddad LJ. Epidemiology of Chlamydia trachomatis in the Middle East and north Africa: a systematic review, meta-analysis, and meta-regression. Lancet Glob Health. 2019;7(9):e1197-e225. doi: 10.1016/S2214-109X(19)30279-7. PubMed PMID: 31402004.

13.	Page MJ, McKenzie JE, Bossuyt PM, Boutron I, Hoffmann TC, Mulrow CD, et al. The PRISMA 2020 statement: an updated guideline for reporting systematic reviews. BMJ. 2021;372:n71. Epub 2021/03/31. doi: 10.1136/bmj.n71. PubMed PMID: 33782057; PubMed Central PMCID: PMCPMC8005924.
