# Supplementary material for: Prevalence and epidemiological patterns of Neisseria gonorrhoeae infection in sub-Saharan Africa, 1964–2025: Systematic review, meta-analyses, and meta-regressions
Source: PLoS Med. 2026 Jun 23;23(6):e1004936. doi: 10.1371/journal.pmed.1004936 (PMC13289870; doi:10.1371/journal.pmed.1004936)
Supplement: Attachment — Submitted filename: Response_to_reviewers_auresp_3.docx [file pmed.1004936.s005.docx]

**Prevalence and epidemiological patterns of *Neisseria gonorrhoeae* infection in sub-Saharan Africa, 1964–2025: systematic review, meta-analyses, and meta-regressions**

REPLY TO EDITORIAL AND REVIEWERS’ COMMENTS

We are grateful to the editor, editorial board, and reviewers for assessing our work and for their insightful and useful feedback and suggestions. Please find below a point-by-point reply addressing each of the comments. We have also incorporated these suggestions in the revised manuscript, as noted below. We would be pleased to address any additional matters, should that be necessary.

Note: All references to the revised manuscript pertain to the marked copies of the manuscript files including changes implemented through “track changes”.

*Dear Dr. Abu-Raddad,*

*Thank you very much for re-submitting your manuscript "Prevalence, patterns, and trends of Neisseria gonorrhoeae infection in sub-Saharan Africa, 1964–2025: systematic review, meta-analyses, and meta-regressions" (PMEDICINE-D-26-00281R2) for review by PLOS Medicine.*

*I have discussed the paper with my colleagues and the academic editor and it was also seen again by 3 reviewers. I am pleased to say that provided the remaining editorial and production issues are dealt with we are planning to accept the paper for publication in the journal.*

Comment: We thank the editor for the time and effort put into handling this submission, the assessment of our work, and the constructive feedback on our manuscript that enriched it and improved its readability. Please find below a point-by-point reply addressing each of the editorial and reviewers’ comments.

**Please note that the academic editor and statistical reviewer require deposition of the code and data in a public repository.*

Answer: Thank you for this useful suggestion. In response to the recommendation from the Academic Editor and Statistical Reviewer, the extracted dataset and the analytical code used for the meta-analyses and meta-regression analyses have now been deposited in publicly accessible GitHub repositories. The corresponding links have been added to the Data Availability Statement in the manuscript as follows: data repository: <https://github.com/Bayan199/NG-in-Africa_data-repository.git>; code repository: <https://github.com/Bayan199/NG-in-Africa_code-repository.git> (Data availability statement).

*The remaining issues that need to be addressed are listed at the end of this email. Any accompanying reviewer attachments can be seen via the link below. Please take these into account before resubmitting your manuscript:*

****Please note while forming your response, if your article is accepted, you may have the opportunity to make the peer review history publicly available. The record will include editor decision letters (with reviews) and your responses to reviewer comments. If eligible, we will contact you to opt in or out.****

Answer: These instructions have been noted and addressed as indicated.

*In revising the manuscript for further consideration here, please ensure you address the specific points made by each reviewer and the editors. In your rebuttal letter you should indicate your response to the reviewers' and editors' comments and the changes you have made in the manuscript. Please submit a clean version of the paper as the main article file. A version with changes marked must also be uploaded as a marked up manuscript file.*

Answer: These instructions have been noted and addressed as indicated.

*Please also check the guidelines for revised papers at* [*http://journals.plos.org/plosmedicine/s/revising-your-manuscript*](http://journals.plos.org/plosmedicine/s/revising-your-manuscript) *for any that apply to your paper. If you haven't already, we ask that you provide a short, non-technical Author Summary of your research to make findings accessible to a wide audience that includes both scientists and non-scientists. The Author Summary should immediately follow the Abstract in your revised manuscript. This text is subject to editorial change and should be distinct from the scientific abstract.*

Answer: These instructions have been noted and addressed as indicated.

*In addition to these revisions, you may need to complete some formatting changes, which you will receive in a follow up email. A member of our team will be in touch with a set of requests shortly. If you do not receive a separate email within a few days, please assume that checks have been completed, and no additional changes are required.*

Answer: These instructions have been noted and addressed as indicated.

*We expect to receive your revised manuscript within 1 week. Please email us (*[*plosmedicine@plos.org*](mailto:plosmedicine@plos.org)*) if you have any questions or concerns.*

Answer: These instructions have been noted and addressed as indicated.

*We ask every co-author listed on the manuscript to fill in a contributing author statement. If any of the co-authors have not filled in the statement, we will remind them to do so when the paper is revised. If all statements are not completed in a timely fashion this could hold up the re-review process. Should there be a problem getting one of your co-authors to fill in a statement we will be in contact. YOU MUST NOT ADD OR REMOVE AUTHORS UNLESS YOU HAVE ALERTED THE EDITOR HANDLING THE MANUSCRIPT TO THE CHANGE AND THEY SPECIFICALLY HAVE AGREED TO IT.*

Answer: These instructions have been noted and addressed as indicated.

*Please ensure that the paper adheres to the PLOS Data Availability Policy (see* [*http://journals.plos.org/plosmedicine/s/data-availability*](http://journals.plos.org/plosmedicine/s/data-availability)*), which requires that all data underlying the study's findings be provided in a repository or as Supporting Information. For data residing with a third party, authors are required to provide instructions with contact information for obtaining the data. PLOS journals do not allow statements supported by "data not shown" or "unpublished results." For such statements, authors must provide supporting data or cite public sources that include it.*

Answer: The extracted dataset and the analytical code used for the meta-analyses and meta-regression analyses have now been deposited in publicly accessible GitHub repositories. The corresponding links have been added to the Data Availability Statement in the manuscript as follows: data repository: <https://github.com/Bayan199/NG-in-Africa_data-repository.git>; code repository: <https://github.com/Bayan199/NG-in-Africa_code-repository.git> (Data availability statement).

*To enhance the reproducibility of your results, we recommend that you deposit your laboratory protocols in protocols.io, where a protocol can be assigned its own identifier (DOI) such that it can be cited independently in the future. Additionally, PLOS ONE offers an option to publish peer-reviewed clinical study protocols. Read more information on sharing protocols at* [*https://plos.org/protocols?utm_medium=editorial-email&utm_source=authorletters&utm_campaign=protocols*](https://plos.org/protocols?utm_medium=editorial-email&utm_source=authorletters&utm_campaign=protocols)

Answer: The study protocol has been formally registered with PROSPERO, and the registration details have been included in the manuscript (Methods, Page 6, Paragraph 4).

*Please review your reference list to ensure that it is complete and correct. If you have cited papers that have been retracted, please include the rationale for doing so in the manuscript text, or remove these references and replace them with relevant current references. Any changes to the reference list should be mentioned in the rebuttal letter that accompanies your revised manuscript.*

Answer: The reference list has been reviewed to ensure completeness and accuracy. No retracted references were identified, and therefore no changes were required to the reference list. Accordingly, no modifications to the references were made in the revised manuscript.

*Please note, when your manuscript is accepted, an uncorrected proof of your manuscript will be published online ahead of the final version, unless you've already opted out via the online submission form. If, for any reason, you do not want an earlier version of your manuscript published online or are unsure if you have already indicated as such, please let the journal staff know immediately at* [*plosmedicine@plos.org*](mailto:plosmedicine@plos.org)*.*

Answer: Thank you for this information. This is acceptable to the authors, and no opt-out request is needed at this time.

-----------------------------------------------------------

**Requests from Editors:**

Comment: We thank the editors for the time and effort put into handling this submission, the assessment of our work, and the constructive feedback on our manuscript that enriched it and improved its readability. Please find below a point-by-point reply addressing each of the editorial and reviewers’ comments.

** Patterns and trends appear to be redundant in the title I suggest to remove ', and trends'*

Answer: Thank you for this useful suggestion. The title has been revised accordingly to remove “and trends” to avoid redundancy. The revised title now reads: “Prevalence and epidemiological patterns of *Neisseria gonorrhoeae* infection in sub-Saharan Africa, 1964–2025: systematic review, meta-analyses, and meta-regressions” (Title).

** In the Abstract Background section, please clarify what was not known, to justify performing these analyses.*

Answer: Thank you for this useful suggestion. The Abstract Background section has been revised to clarify the existing evidence gap and the rationale for conducting these analyses (Abstract).

** Please provide an additional sentence to the Conclusions statement in the Abstract suggesting next steps, or remaining gaps in knowledge, etc.*

Answer: Thank you for this useful suggestion. An additional sentence has been added to the Conclusions section of the Abstract to highlight key next steps for strengthening NG control efforts in sub-Saharan Africa (Abstract).

** Please confirm that your abstract complies with our requirements, including format (three sections: Background, Methods and Findings, and Conclusions) and providing all the information relevant to this study type* [*https://journals.plos.org/plosmedicine/s/submission-guidelines#loc-abstract*](https://journals.plos.org/plosmedicine/s/submission-guidelines#loc-abstract)

Answer: The abstract has been reviewed and drafted to ensure compliance with the journal requirements, including the required structure and inclusion of all information relevant to this study type.

** Please ensure that the Introduction ends with a clear description of the study question or hypothesis.*

Answer: The Introduction concludes with a description of the study objectives and research questions addressed in this work.

** Please ensure that all abbreviations are defined at first use throughout the text.*

Answer: The manuscript has been reviewed and checked to ensure that all abbreviations are defined at first use throughout the text.

** Please confirm that all numbers presented in the abstract are present and identical to numbers presented in the main manuscript text.*

Answer: This has been checked to ensure that all numbers presented in the abstract are present and identical to those reported in the main manuscript text.

** All authors must declare their relevant competing interests per the PLOS policy, which can be seen here:* [*https://journals.plos.org/plosmedicine/s/competing-interests*](https://journals.plos.org/plosmedicine/s/competing-interests) *For authors with ties to industry, please indicate whether any of the interests has a financial stake in the results of the current study.*

Answer: These instructions have been noted and addressed as indicated.

** Please remove 'modest' from your competing interests statement due to its subjectivity.*

Answer: This has been addressed by removing the term “modest” from the competing interests statement (Competing interests).

** PLOS defines the “minimal data set” to consist of the data set used to reach the conclusions drawn in the manuscript with related metadata and methods, and any additional data required to replicate the reported study findings in their entirety. Authors do not need to submit their entire data set, or the raw data collected during an investigation. Please submit the following data:*

*The values behind the means, standard deviations and other measures reported; The values used to build graphs; The points extracted from images for analysis.*

Answer: Thank you for this clarification. The minimal dataset required to reproduce the analyses, including the extracted analytical dataset underlying the reported estimates, tables, and figures, together with the corresponding analytical code, has been deposited in the publicly accessible GitHub repositories referenced in the Data Availability Statement (Data availability statement).

** The Data Availability Statement (DAS) and code statements require revision to address the request from the academic editor and the statistical reviewer.*

*If the data are freely or publicly available, note this and state the location of the data: within the paper, in Supporting Information files, or in a public repository (include the DOI or accession number).*

Answer: The extracted dataset and the analytical code used for the meta-analyses and meta-regression analyses have now been deposited in publicly accessible GitHub repositories. The corresponding links have been added to the Data Availability Statement in the manuscript as follows: data repository: <https://github.com/Bayan199/NG-in-Africa_data-repository.git>; code repository: <https://github.com/Bayan199/NG-in-Africa_code-repository.git> (Data availability statement).

** Please provide the source code needed to replicate the study's findings in a repository (such as GitHub, SourceForge or Bitbucket) or a cloud computing service (such as Code Ocean). Protection of authors’ intellectual property will not be cause for exception. Please explain in the manuscript’s Data Availability Statement how readers can access the shared code.*

Answer: The extracted dataset and the analytical code used for the meta-analyses and meta-regression analyses have now been deposited in publicly accessible GitHub repositories. The corresponding links have been added to the Data Availability Statement in the manuscript as follows: data repository: <https://github.com/Bayan199/NG-in-Africa_data-repository.git>; code repository: <https://github.com/Bayan199/NG-in-Africa_code-repository.git> (Data availability statement).

** Please provide the unadjusted comparisons as well as the adjusted comparisons in all relevant Tables.*

Answer: Thank you for this useful suggestion. All meta-regression analysis tables already include both unadjusted and adjusted effect size estimates.

** Please specify the variables controlled for in all relevant Tables, if relevant.*

Answer: This has already been addressed in all meta-regression analysis results tables. The criteria for inclusion of variables in the multivariable analyses are described in the Methods section and all variables controlled for in the adjusted analyses are presented in the corresponding tables.

** Please show graph axes beginning at zero. If this is not possible, please show a break in the axis.*

Answer: This is already satisfied in all figures where applicable, with graph axes beginning at zero in all relevant figures.

** Please confirm that the appropriate usage rights apply to the use of the map in the supporting information. Please see our guidelines for map images:* [*https://journals.plos.org/plosmedicine/s/figures#loc-maps*](https://journals.plos.org/plosmedicine/s/figures#loc-maps)

Answer: We confirm that the appropriate usage rights apply to the use of the map in the Supporting Information. The map included in the earlier version of the Supporting Information has now been replaced by a new map generated in R using Natural Earth public-domain country boundary shapefiles (<https://www.naturalearthdata.com/>), which are compatible with the Creative Commons Attribution (CC BY) license requirements of PLOS journals (S1 Fig).

** Please clarify for figures whether they include images of branded products or are generated by commercial products. Please confirm you have appropriate permission or you may wish to remove these images. For more information, please see our guidelines:* [*https://journals.plos.org/plosmedicine/s/figures#loc-licenses-and-copyright*](https://journals.plos.org/plosmedicine/s/figures#loc-licenses-and-copyright) *and* [*https://journals.plos.org/plosmedicine/s/licenses-and-copyright*](https://journals.plos.org/plosmedicine/s/licenses-and-copyright)

*Figures, tables, and images in PLOS journals are published under the Creative Commons Attribution (CC BY) license.*

Answer: We confirm that the figures do not include images of branded products. The map was generated in R using Natural Earth public-domain country boundary shapefiles (<https://www.naturalearthdata.com/>), which are compatible with the Creative Commons Attribution (CC BY) license requirements of PLOS journals (S1 Fig).

** Please clarify the reason for the inclusion of the 'terms of service' pdf in the file inventory.*

Answer: The “terms of service” PDF was initially included to document map usage permissions. However, as the map has now been regenerated in R using Natural Earth public-domain shapefiles, this file is no longer required and can be removed from the file inventory.

-----------------------------------------------------------

**Comments from the reviewers:**

Reviewer #1:

*I would like to thank the authors for providing detailed responses to the comments that I had.*

Comment: We thank the reviewer for the time and effort put into this review, the assessment of our work, and the constructive feedback on our manuscript that enriched it and improved its readability.

Reviewer #2:

*The manuscript has been excellently revised. Thank you for responding thoughtfully to my comments, and congratulations to the entire team on this valuable contribution to the epidemiological literature.*

Comment: We thank the reviewer for the time and effort put into this review, the assessment of our work, and the constructive feedback on our manuscript that enriched it and improved its readability.

Reviewer #3:

*I'm fine with the responses, except on code & data.*

Comment: We thank the reviewer for the time and effort put into this review, the assessment of our work, and the constructive feedback on our manuscript that enriched it and improved its readability. Please find below a point-by-point reply addressing each of the reviewer’s comments.

*Saying the code is available in the published manuscripts is weak: the point of the SR is to have gathered it. Saying it will be part of some database in the future is not a reason not to release it with this paper.*

*Similarly, saying you've used standard commands is not a reason not to release analysis code. It could still contain errors or steps that would facilitate reproduction of results by others.*

Answer: Thank you for this useful suggestion. In response to the recommendation from the Academic Editor and Statistical Reviewer, the extracted dataset and the analytical code used for the meta-analyses and meta-regression analyses have now been deposited in publicly accessible GitHub repositories. The corresponding links have been added to the Data Availability Statement in the manuscript as follows: data repository: <https://github.com/Bayan199/NG-in-Africa_data-repository.git>; code repository: <https://github.com/Bayan199/NG-in-Africa_code-repository.git> (Data availability statement).

-----------------------------------------------------------

**Journal Requirements:**

*1) Please ensure your short title is correct in the submission form, including symbols, italicisation etc. Your short title will be typeset from the submission form directly.*

*NOTE: For clinical trials, systematic reviews, or meta-analyses, the subtitle should include the study design.*

Answer: The short title has now been revised to comply with these requirements, and we will ensure that this is reflected in the online submission. The new title reads: “Gonorrhea epidemiology in sub-Saharan Africa: systematic review and meta-analysis” (Short title).

*2) In your Data Availability Statement, please ensure you have provided all necessary access information in complete sentences. Access information should include the name of any data repositories used as well as digital object identifiers (DOIs), accession numbers or codes, or other persistent identifiers for all relevant data. If all data has been provided within your submission files, please use the statement "All relevant data is available within the manuscript and Supporting Information files." The Data Availability Statement will appear on the published version of your manuscript.*

Answer: In response to the recommendation from the Academic Editor and Statistical Reviewer, the extracted dataset and the analytical code used for the meta-analyses and meta-regression analyses have now been deposited in publicly accessible GitHub repositories. The corresponding links have been added to the Data Availability Statement in the manuscript as follows: data repository: <https://github.com/Bayan199/NG-in-Africa_data-repository.git>; code repository: <https://github.com/Bayan199/NG-in-Africa_code-repository.git> (Data availability statement).

*3) Some material included in your submission may be copyrighted. According to PLOS’s copyright policy, authors who use figures or other material (e.g., graphics, clipart, maps) from another author or copyright holder must demonstrate or obtain permission to publish this material under the Creative Commons Attribution 4.0 International (CC BY 4.0) License used by PLOS journals. Please closely review the details of PLOS’s copyright requirements here: PLOS Licenses and Copyright. If you need to request permissions from a copyright holder, you may use PLOS's Copyright Content Permission form.*

*Please respond directly to this email and provide any known details concerning your material's license terms and permissions required for reuse, even if you have not yet obtained copyright permissions or are unsure of your material's copyright compatibility. Once you have responded and addressed all other outstanding technical requirements, you may resubmit your manuscript within Editorial Manager.*

*Potential Copyright Issues:*

*[ Figure S1]: Thank you for stating "Source: SmartDraw Software, LLC (2026). Map adapted using the SmartDraw online platform and reproduced with permission. This material is not covered by the article’s CC BY 4.0 license.*

*The base map layer is available at:* [*https://app.smartdraw.com/editor.aspx?templateId=cdf0e65a-cd4d-47eb-97d6-5846db784aa0&flags=128*](https://app.smartdraw.com/editor.aspx?templateId=cdf0e65a-cd4d-47eb-97d6-5846db784aa0&flags=128)*"*

*This copyright to SmartDraw Software is too restrictive. Please either remove or recreate using a open source alternative*

*See these open source resources you may use to replace images / clip-art:*

*-* [*https://commons.wikimedia.org*](https://commons.wikimedia.org)

*-* [*https://openclipart.org/*](https://openclipart.org/)*"*

Answer: We confirm that the appropriate usage rights apply to the use of the map in the Supporting Information. The map included in the earlier version of the Supporting Information has now been replaced by a new map generated in R using Natural Earth public-domain country boundary shapefiles (<https://www.naturalearthdata.com/>), which are compatible with the Creative Commons Attribution (CC BY) license requirements of PLOS journals (S1 Fig).
