# Supplementary material for: Prevalence and epidemiological patterns of Neisseria gonorrhoeae infection in sub-Saharan Africa, 1964–2025: Systematic review, meta-analyses, and meta-regressions
Source: PLoS Med. 2026 Jun 23;23(6):e1004936. doi: 10.1371/journal.pmed.1004936 (PMC13289870; doi:10.1371/journal.pmed.1004936)
Supplement: Peer review history

# Editor report: Decision Letter 0

**DOI:** 10.1371/journal.pmed.1004936.r001

**Authors:** Alison Farrell

27 Jan 2026

Dear Dr Abu-Raddad,

Thank you for submitting your manuscript entitled "Prevalence, patterns, and trends of Neisseria gonorrhoeae infection in sub-Saharan Africa, 1964–2025: systematic review, meta-analyses, and meta-regressions" for consideration by PLOS Medicine.

Your manuscript has now been evaluated by the PLOS Medicine editorial staff, and I am writing to let you know that we would like to send your submission out for external peer review.

However, before we can send your manuscript to reviewers, we need you to complete your submission by providing the metadata that is required for full assessment. To this end, please login to Editorial Manager where you will find the paper in the 'Submissions Needing Revisions' folder on your homepage. Please click 'Revise Submission' from the Action Links and complete all additional questions in the submission questionnaire.

For clinical studies, please upload a copy of your trial study protocol as a supporting information file. The study protocol should be the version submitted for approval to the institutional review board or ethics committee, should include any amendments to the study protocol, as well as the date of their approval by the institutional review or ethics committee. Please also detail any deviations from the study protocol in the Methods section of your manuscript. The editors will consider the protocol and study conduct prior to a final decision for external review.

Please re-submit your manuscript within two working days, i.e. by Jan 29 2026 11:59PM.

Login to Editorial Manager here: https://www.editorialmanager.com/pmedicine

Once your full submission is complete, your paper will undergo a series of checks in preparation for peer review. Once your manuscript has passed all checks it will be sent out for review.

Feel free to email us at plosmedicine@plos.org if you have any queries relating to your submission.

Kind regards,

Alison Farrell, Ph.D.

Senior Editor

PLOS Medicine

# Peer review: Decision Letter 1

**DOI:** 10.1371/journal.pmed.1004936.r002

**Authors:** Alison Farrell

13 Mar 2026

Dear Dr Abu-Raddad,

Many thanks for submitting your manuscript "Prevalence, patterns, and trends of Neisseria gonorrhoeae infection in sub-Saharan Africa, 1964–2025: systematic review, meta-analyses, and meta-regressions" (PMEDICINE-D-26-00281R1) to PLOS Medicine. The paper has been reviewed by subject experts and a statistician; their comments are included below and can also be accessed here: [LINK]

As you will see, the reviewers find the study interesting and recommend revising the manuscript to address their independent concerns. In particular, the reviewers request the inclusion of the study protocol and substantially more detail on the methodology of the search and analyses, as well as presentation of subregional estimates and sub-pooled estimates for different population groups. We ask you to temper claims and discuss study limitations, and to extend a caveated discussion elaborating on potential policy implications of the findings. After discussing the paper with the editorial team and an academic editor with relevant expertise, I'm pleased to invite you to revise the paper in response to the reviewers' and academic editor's comments. We plan to send the revised paper to some or all of the original reviewers, and we cannot provide any guarantees at this stage regarding publication.

Please update your search to within 6 months of the anticipated date of resubmission.

Please clarify in the text if the search was restricted to English language publications.

If new code was generated for any analyses, please deposit it in a public repository and provide a doi in the Data Availability statement.

Please include the funder URL(s) in the manuscript metadata.

The last sentence of the Methods and Findings in the Abstract should state the methodological limitations.

Please add to the introduction a statement that at the time of writing, there is no licensed vaccine against NG.

Please clarify why the GRADE approach is not used to assess certainty of evidence.

Please clarify why risk of publication bias is marked as N/A in the PRISMA checklist.

Is 'mixed sexes', defined in Table 1, used in the cited articles? Please review your manuscript and edit to ensure compliance with our inclusive language requirements https://journals.plos.org/plosmedicine/s/human-subjects-research#loc-categorization

In addition to these revisions, you may need to complete some formatting changes, which you will receive in a follow up email. A member of our team will be in touch with a set of requests shortly. If you do not receive a separate email within a few days, please assume that checks have been completed, and no additional changes are required.

When you upload your revision, please include a point-by-point response that addresses all of the reviewer and editorial points, indicating the changes made in the manuscript and either an excerpt of the revised text or the location (eg: page and line number) where each change can be found. Please also be sure to check the general editorial comments at the end of this letter and include these in your point-by-point response. When you resubmit your paper, please include a clean version of the paper as the main article file and a version with changes tracked as a marked-up manuscript. It may also be helpful to check the guidelines for revised papers at http://journals.plos.org/plosmedicine/s/revising-your-manuscript for any that apply to your paper.

We ask that you submit your revision by Apr 03 2026 11:59PM. However, if this deadline is not feasible, please contact me by email, and we can discuss a suitable alternative.

Don't hesitate to contact me directly with any questions (afarrell@plos.org).

Best regards,

Alison

Alison Farrell, Ph.D.

Senior Editor

PLOS Medicine

afarrell@plos.org

-----------------------------------------------------------

Comments from the academic editor:

Between-study heterogeneity is extreme for most pooled estimates (often I² >50% with wide prediction intervals). Yet, the manuscript still emphasizes pooled values (e.g. general population prevalence of 3.2%). Given the heterogeneity, I recommend further de-emphasizing pooled point estimates and placing greater weight on prevalence distributions, population-type gradients, and study-design effects. The meta-regression analyses are informative and explain a large proportion of heterogeneity, identifying coherent epidemiological gradients by population type, age, and subregion.

The risk-of-bias framework remains conceptually limited. Precision (sample size, inappropriately dichotomized at 200), reporting quality, and bias are partly conflated, and the assessment does not adequately capture the fundamental non-representativeness of many clinic-based and convenience samples. The assumptions underlying the assessment of publication bias are unclear. While inclusion of sampling method and sample size in meta-regression is a strength, the manuscript would benefit from a clearer acknowledgment that many included studies cannot be interpreted as population prevalence estimates, regardless of statistical precision.

The observed decline in prevalence (~1% per year) may be plausible, but interpretation should be more cautious. Changes in diagnostics, testing practices, healthcare access, and study populations over time may contribute substantially to observed trends. I suggest strengthening the discussion to emphasize that true incidence trends cannot be inferred with certainty from these data.

-----------------------------------------------------------

Comments from the reviewers:

Reviewer #1: This is an exceptionally well conducted and written study as well as very relevant for understanding the patterns in epidemiology of NG in sub-saharan Africa.

Following are my comments:

Under Methods, the section on study selection and eligibility criteria, please specify which specimens were taken into consideration for studies' inclusion.

Under Results, it is more common to use abbreviation IBBS than IBBSS.

Rows 246-249 of Results: please clarify the criteria for the population groups that prevalence estimates are shown for? Suggest not to group symptomatic men and women with FSW, MSM and STI clinic attendees since they overlap (i.e. there are symptomatic women among FSW). Also, it might be good to show estimates for other groups (clients of FSW, military men, ANC clinic attendees, etc).

* Of note is that estimates for other population groups are shown later in Results.

In the rows 278-280 please add the number of studies for urogenital, anorectal and oropharyngeal prevalence estimates as the latter might include a relatively small number of studies.

Row 298: For serological evidence of ever having had NG infection, please show, if available estimates for MSM.

Discussion

Row 382: Due to the highest prevalence in persons younger than 25 years, it might be beneficial to highlight this population group (particularly from KPs and vulnerable groups) as in need of more intensified prevention and testing.

Reviewer #2: Congratulations to the authors on this large and ambitious systematic review and meta-analysis synthesizing 950 publications, 1,604 prevalence measures, and data from approximately 2.7 million individuals across 1964-2025. The authors estimate a pooled urogenital prevalence of 3.2% in general populations, identify an approximate 1% annual decline, and demonstrate a clear risk gradient across population groups (e.g., higher prevalence among symptomatic men, female sex workers, and anorectal/oropharyngeal infections among MSM). The manuscript is well written and methodologically thoughtful. However, several issues related to transparency, methodological clarity and interpretability, if addressed, would make the manuscript most suitable for publication.

Major comments

1. Study protocol

* Please upload the full search and analysis protocol as Supplementary Material and clearly describe all deviations or adaptations relative to any previously published protocol.

2. Definition of prevalence

* The manuscript does not clearly define "prevalence": e.g. whether it refers to point prevalence, test positivity, or another measure.

* Please specify the time frame, numerator/denominator, and rules used when multiple anatomical sites were collected from the same participant.

* The search strategy (Table S2) contains pathogen, disease, and country terms but no explicit prevalence related terms. Please clarify how your operational definition of prevalence informed study identification.

3. General population estimate

* The reported 3.2% pooled estimate combines heterogeneous population groups (e.g., ANC attendees, blood donors, community based samples).

* To support interpretability, please consider providing sub pooled estimates for these groups separately and conduct a sensitivity analysis restricted to probability based samples to better approximate a representative population prevalence.

4. Heterogeneity and small study effects

* Many pooled estimates display high heterogeneity (I²), wide prediction intervals and a pronounced small study effect.

* Please include prediction intervals prominently in the main Results section and also in the Abstract.

5. Diagnostic methods over six decades

* The evidence base spans multiple decades and diagnostic modalities (culture, Gram stain, NAATs).

* Please provide assay specific pooled estimates (e.g., NAAT only analyses) and discuss how assay evolution may have influenced prevalence estimates.

* Relatedly, consider elaborating on potential spectrum bias between facility and community based samples, given differences in symptom status and risk profiles.

6. Temporal trend presentation

* The 1% annual decline is an important finding but appears only in tables.

* Please incorporate this result into the main Results text with a clear trend figure (based on publication year and, in sensitivity analyses, year of data collection). If data allow, presenting subregional trends would be very informative.

7. Geographic scope

* The title refers to sub Saharan Africa, yet Mauritania (a North African country) is included in the dataset.

* Please justify this inclusion or adjust wording and analytical framing accordingly.

8. Comparability with recent syntheses

* Please add a short comparison with other recent SSA prevalence estimates, explaining how differences in inclusion criteria, population groups, time periods, and diagnostic methods may account for the higher pooled values observed here.

9. Policy implications

The discussion could be strengthened by further elaborating on the policy and programmatic implications, particularly for screening strategies, partner notification, and antimicrobial resistance (AMR) surveillance. The authors may also consider positioning their findings in relation to the 2025 WHO guidance on the management of asymptomatic NG/CT, which outlines prevalence thresholds for screening among young people and pregnant women.

Minor comments

* Search strategy: Please provide complete reproducible search strings (Boolean operators, platforms/hosts, final search date of 4 June 2025) and database specific start dates. Clarify language coverage, including whether French language publications were captured.

* Risk of bias: Specify the exact risk of bias domains used, provide a per study risk of bias summary table in the Supplement. Alternatively, explain why this was not presented.

* Figures/Tables: Consider adding a bubble plot for the meta regression (bubble size = sample size; colour = population type) and a map of country level data coverage. Please include prediction intervals wherever pooled prevalence estimates are presented.

Reviewer #3: ABSTRACT

It would be ideal to introduce 95%CI at first use.

The conclusion (higher than other regions) doesn't flow without having presented prevalence in other regions in Background.

Similarly, WHO target should be introduced in Background before mention in Conclusions.

Some editorialising of group-by-group prevalences would allow more words if needed.

INTRODUCTION

'A key strength...' line 85: this paragraph is Discussion rather than Introduction & should be moved or deleted.

METHODS

'not formally registered with PROSPERO' -> just 'not registered with PROSPERO': there is no notion of formal vs informal or other type of registration.

search strategy: the supplement notwithstanding, this section should convey some sort of summary of the search undertaken, e.g. what combination of concepts was sought?

Precision categorization, line 165: I don't understand the rationale for categorization of precision given that study CIs provide a continuous direct quantification of precision. Moreover, using study count to categorize precision may be problematic for structured survey samples where N is not the only determinant of precision.

Without a protocol, I think it's important to state somewhere which (if any) elements of the statistical analysis were prespecified (e.g. choice of groups, p-value cut-offs), or to state that the analyses were not prespecified (if this is true).

Meta-analyses & Meta-regression: I don't think it is explicitly stated what measure of precision was associated with each individual data-point, and how this was derived from the data (e.g. SD estimate from CIs?). This should be done separately in each section.

Meta-regression: please state fully what structure was represented via random effects.

RESULTS

lines 224-231: Fewer details are needed in words in my opinion, given they are all on Figure 1.

Now I see how low/high 'precision' categories were used, I think it is mainly a terminological issue. I would just avoid the word precision, e.g. by calling the studies small or not small.

Introduce '95% CI' at first use.

DISCISSION

Opening paragraph: of course Africa is also a notably young region and NG has higher prevalence among the young. I think it would be important to briefly consider age-specific/standardized comparisons to assure readers this is comparatively high prevalence is not just due to different demography.

FIGURE 1

There seems to be a missing 'Records eligible for inclusion' box on the database half of the diagram.

What does 'marked as ineligible by automation tools' mean?

FIGURE SUGGESTION

I think a map showing where the data came from (e.g. point size or count as text) potentially with choropleth encoding of the most commonly-reported population prevalence would be really useful. If the authors don't want to add more exhibits, the table defining risk groups could be moved to the supplement.

Africa is such a huge and varied place, I do think it is important to convey where the data are from, and any variation across countries better than is currently done.

CODE & DATA AVAILABILITY

I didn't see the data and code from this study were made available publicly. This would be useful and enhance transparency and reproducibility.

Any attachments provided with reviews can be seen via the following link: [LINK]

--------------------------------------------------------- ---

General editorial requests:

Please check each item carefully and indicate where addressed.

* We ask every co-author listed on the manuscript to fill in a contributing author statement, making sure to declare all competing interests. If any of the co-authors have not filled in the statement, we will remind them to do so when the paper is revised. If all statements are not completed in a timely fashion this could hold up the re-review process. If new competing interests are declared later in the revision process, this may also hold up the submission. Should there be a problem getting one of your co-authors to fill in a statement we will be in contact. Please do not add or remove authors without first discussing this with the handling editor. You can see our competing interests policy here: http://journals.plos.org/plosmedicine/s/competing-interests.

* Please upload any figures associated with your paper as individual TIF or EPS files with 300dpi resolution at resubmission; please read our figure guidelines for more information on our requirements: http://journals.plos.org/plosmedicine/s/figures. While revising your submission, we strongly recommend that you use PLOS's NAAS tool (https://ngplosjournals.pagemajik.ai/artanalysis) to test your figure files. NAAS can convert your figure files to the TIFF file type and meet basic requirements (such as print size, resolution), or provide you with a report on issues that do not meet our requirements and that NAAS cannot fix.

After uploading your figures to PLOS's NAAS tool - https://ngplosjournals.pagemajik.ai/artanalysis, NAAS will process the files provided and display the results in the "Uploaded Files" section of the page as the processing is complete.

If the uploaded figures meet our requirements (or NAAS is able to fix the files to meet our requirements), the figure will be marked as "fixed" above. If NAAS is unable to fix the files, a red "failed" label will appear above.

When NAAS has confirmed that the figure files meet our requirements, please download the file via the download option, and include these NAAS processed figure files when submitting your revised manuscript.

* Please ensure that the paper adheres to the PLOS Data Availability Policy (see http://journals.plos.org/plosmedicine/s/data-availability), which requires that all data underlying the study's findings be provided in a repository or as Supporting Information. For data residing with a third party, authors are required to provide instructions with contact information (web or email address) for obtaining the data. Please note that a study author cannot be the contact person for the data. PLOS journals do not allow statements supported by "data not shown" or "unpublished results." For such statements, authors must provide supporting data or cite public sources that include it.

* We expect all researchers with submissions to PLOS in which author-generated code underpins the findings in the manuscript to make all author-generated code available without restrictions upon publication of the work. In cases where code is central to the manuscript, we may require the code to be made available as a condition of publication. Authors are responsible for ensuring that the code is reusable and well documented. Please make any custom code available, either as part of your data deposition or as a supplementary file. Please add a sentence to your data availability statement regarding any code used in the study, e.g. "The code used in the analysis is available from Github [URL] and archived in Zenodo [DOI link]" Please review our guidelines at https://journals.plos.org/plosmedicine/s/materials-software-and-code-sharing and ensure that your code is shared in a way that follows best practice and facilitates reproducibility and reuse. Because Github depositions can be readily changed or deleted, we encourage you to make a permanent DOI'd copy (e.g. in Zenodo) and provide the URL.

* Please ensure that the study is reported according to the PRISMA guideline and include the completed PRISMA checklist as Supporting Information. When completing the checklist, please use section and paragraph numbers, rather than page numbers. Please add the following statement, or similar, to the Methods: "This study is reported as per PRISMA guideline (S1 Checklist)."

FORMATTING - GENERAL

* Abstract: Please structure your abstract using the PLOS Medicine headings (Background, Methods and Findings, Conclusions). Please combine the Methods and Findings sections into one section.

* At this stage, we ask that you include a short, non-technical Author Summary of your research to make findings accessible to a wide audience that includes both scientists and non-scientists. The Author Summary should immediately follow the Abstract in your revised manuscript. This text is subject to editorial change and should be distinct from the scientific abstract. Ideally each sub-heading should contain 2-3 single sentence, concise bullet points containing the most salient points from your study. In the final bullet point of 'What Do These Findings Mean?', please include the main limitations of the study in non-technical language. Please see our author guidelines for more information: https://journals.plos.org/plosmedicine/s/revising-your-manuscript#loc-author-summary.

* Please express the main results with 95% CIs as well as p values. When reporting p values please report as p<0.001 and where higher as the exact p value p=0.002, for example. Throughout, suggest reporting statistical information as follows to improve clarity for the reader "22% (95% CI [13%,28%]; p</=)". Please be sure to define all numerical values at first use.

* Please include page numbers and line numbers in the manuscript file. Use continuous line numbers (do not restart the numbering on each page).

* Please cite the reference numbers in square brackets. Citations should precede punctuation.

FIGURES AND TABLES

* Please provide titles and legends for all figures and tables (including those in Supporting Information files).

* Please define all abbreviations used in each figure/table (including those in Supporting Information files).

* Please consider avoiding the use of red and green in order to make your figure more accessible to those with color blindness.

SUPPLEMENTARY MATERIAL

* Please note that supplementary material will be posted as supplied by the authors. Therefore, please amend it according to the relevant comments outlined here.

* Please cite your Supporting Information as outlined here: https://journals.plos.org/plosmedicine/s/supporting-information

REFERENCES

* PLOS uses the numbered citation (citation-sequence) method and first six authors, et al.

* Please ensure that journal name abbreviations match those found in the National Center for Biotechnology Information (NCBI) databases (http://www.ncbi.nlm.nih.gov/nlmcatalog/journals), and are appropriately formatted and capitalised.

* Where website addresses are cited, please include the complete URL and specify the date of access (e.g. [accessed: 12/06/2023]).

* Please also see https://journals.plos.org/plosmedicine/s/submission-guidelines#loc-references for further details on reference formatting.

* [FOR POPULATION HEALTH ESTIMATES] If pertinent, please ensure that the study is reported according to the GATHER statement (available from https://www.equator-network.org/reporting-guidelines/gather-statement) and include the completed checklist as Supporting Information. Please add the following statement, or similar, to the Methods: "This study is reported as per the Guidelines for Accurate and Transparent Health Estimates Reporting (GATHER) statement (S1 Checklist)." When completing the checklist, please use section and paragraph numbers, rather than page numbers.

* Please state in the Methods section whether the study had a prospective protocol or analysis plan. If a prospective analysis plan (from your funding proposal, IRB or other ethics committee submission, study protocol, or other planning document written before analyzing the data) was used in designing the study, please include the relevant document(s) with your revised manuscript as a Supporting Information file to be published alongside your study and cite it in the Methods section. A legend for this file should be included at the end of your manuscript. If no such document exists, please make sure that the Methods section transparently describes when analyses were planned, and when/why any data-driven changes to analyses took place. Changes in the analysis, including those made in response to peer review comments, should be identified as such in the Methods section of the paper, with rationale.

SYSTEMATIC REVIEWS & META-ANALYSES

* Please report your SR/MA according to the PRISMA guidelines provided at the EQUATOR site. http://www.equator-network.org/reporting-guidelines/prisma/. Please provide the completed PRISMA checklist as Supporting Information. When completing the checklist, please use section and paragraph numbers, rather than page numbers. Please add the following statement, or similar, to the Methods: "This study is reported as per the Preferred Reporting Items for Systematic Reviews and Meta-Analyses (PRISMA) guideline (S1 Checklist)."

* Abstract: Please report your abstract according to PRISMA for abstracts (https://doi.org/10.1371/journal.pmed.1001419) following the PLOS Medicine abstract structure (Background, Methods and Findings, Conclusions). Please ensure you provide dates of search, data sources, number of studies included, types of study designs included, eligibility criteria, and synthesis/appraisal methods.

* Please note that we expect searches to be updated to within 6 months of the time of submission.

# Author comment: Author response to Decision Letter 2

**DOI:** 10.1371/journal.pmed.1004936.r003

6 Apr 2026

Attachment Submitted filename: Response to reviewers.docx

# Peer review: Decision Letter 2

**DOI:** 10.1371/journal.pmed.1004936.r004

**Authors:** Alison Farrell

12 May 2026

Dear Dr. Abu-Raddad,

Thank you very much for re-submitting your manuscript "Prevalence, patterns, and trends of Neisseria gonorrhoeae infection in sub-Saharan Africa, 1964–2025: systematic review, meta-analyses, and meta-regressions" (PMEDICINE-D-26-00281R2) for review by PLOS Medicine.

I have discussed the paper with my colleagues and the academic editor and it was also seen again by 3 reviewers. I am pleased to say that provided the remaining editorial and production issues are dealt with we are planning to accept the paper for publication in the journal.

*Please note that the academic editor and statistical reviewer require deposition of the code and data in a public repository.

The remaining issues that need to be addressed are listed at the end of this email. Any accompanying reviewer attachments can be seen via the link below. Please take these into account before resubmitting your manuscript:

[LINK]

***Please note while forming your response, if your article is accepted, you may have the opportunity to make the peer review history publicly available. The record will include editor decision letters (with reviews) and your responses to reviewer comments. If eligible, we will contact you to opt in or out.***

In revising the manuscript for further consideration here, please ensure you address the specific points made by each reviewer and the editors. In your rebuttal letter you should indicate your response to the reviewers' and editors' comments and the changes you have made in the manuscript. Please submit a clean version of the paper as the main article file. A version with changes marked must also be uploaded as a marked up manuscript file.

Please also check the guidelines for revised papers at http://journals.plos.org/plosmedicine/s/revising-your-manuscript for any that apply to your paper. If you haven't already, we ask that you provide a short, non-technical Author Summary of your research to make findings accessible to a wide audience that includes both scientists and non-scientists. The Author Summary should immediately follow the Abstract in your revised manuscript. This text is subject to editorial change and should be distinct from the scientific abstract.

In addition to these revisions, you may need to complete some formatting changes, which you will receive in a follow up email. A member of our team will be in touch with a set of requests shortly. If you do not receive a separate email within a few days, please assume that checks have been completed, and no additional changes are required.

We expect to receive your revised manuscript within 1 week. Please email us (plosmedicine@plos.org) if you have any questions or concerns.

We ask every co-author listed on the manuscript to fill in a contributing author statement. If any of the co-authors have not filled in the statement, we will remind them to do so when the paper is revised. If all statements are not completed in a timely fashion this could hold up the re-review process. Should there be a problem getting one of your co-authors to fill in a statement we will be in contact. YOU MUST NOT ADD OR REMOVE AUTHORS UNLESS YOU HAVE ALERTED THE EDITOR HANDLING THE MANUSCRIPT TO THE CHANGE AND THEY SPECIFICALLY HAVE AGREED TO IT.

Please ensure that the paper adheres to the PLOS Data Availability Policy (see http://journals.plos.org/plosmedicine/s/data-availability), which requires that all data underlying the study's findings be provided in a repository or as Supporting Information. For data residing with a third party, authors are required to provide instructions with contact information for obtaining the data. PLOS journals do not allow statements supported by "data not shown" or "unpublished results." For such statements, authors must provide supporting data or cite public sources that include it.

To enhance the reproducibility of your results, we recommend that you deposit your laboratory protocols in protocols.io, where a protocol can be assigned its own identifier (DOI) such that it can be cited independently in the future. Additionally, PLOS ONE offers an option to publish peer-reviewed clinical study protocols. Read more information on sharing protocols at https://plos.org/protocols?utm_medium=editorial-email&utm_source=authorletters&utm_campaign=protocols

Please review your reference list to ensure that it is complete and correct. If you have cited papers that have been retracted, please include the rationale for doing so in the manuscript text, or remove these references and replace them with relevant current references. Any changes to the reference list should be mentioned in the rebuttal letter that accompanies your revised manuscript.

Please note, when your manuscript is accepted, an uncorrected proof of your manuscript will be published online ahead of the final version, unless you've already opted out via the online submission form. If, for any reason, you do not want an earlier version of your manuscript published online or are unsure if you have already indicated as such, please let the journal staff know immediately at plosmedicine@plos.org.

If you have any questions in the meantime, please contact me or the journal staff on plosmedicine@plos.org.

We look forward to receiving the revised manuscript by May 19 2026 11:59PM.

Sincerely,

Alison Farrell, Ph.D.

Senior Editor

PLOS Medicine

plosmedicine.org

------------------------------------------------------------

Requests from Editors:

* Patterns and trends appear to be redundant in the title I suggest to remove ', and trends'

* In the Abstract Background section, please clarify what was not known, to justify performing these analyses.

* Please provide an additional sentence to the Conclusions statement in the Abstract suggesting next steps, or remaining gaps in knowledge, etc.

* Please confirm that your abstract complies with our requirements, including format (three sections: Background, Methods and Findings, and Conclusions) and providing all the information relevant to this study type https://journals.plos.org/plosmedicine/s/submission-guidelines#loc-abstract

* Please ensure that the Introduction ends with a clear description of the study question or hypothesis.

* Please ensure that all abbreviations are defined at first use throughout the text.

* Please confirm that all numbers presented in the abstract are present and identical to numbers presented in the main manuscript text.

* All authors must declare their relevant competing interests per the PLOS policy, which can be seen here: https://journals.plos.org/plosmedicine/s/competing-interests For authors with ties to industry, please indicate whether any of the interests has a financial stake in the results of the current study.

* Please remove 'modest' from your competing interests statement due to its subjectivity.

* PLOS defines the “minimal data set” to consist of the data set used to reach the conclusions drawn in the manuscript with related metadata and methods, and any additional data required to replicate the reported study findings in their entirety. Authors do not need to submit their entire data set, or the raw data collected during an investigation. Please submit the following data:

The values behind the means, standard deviations and other measures reported;

The values used to build graphs;

The points extracted from images for analysis.

* The Data Availability Statement (DAS) and code statements require revision to address the request from the academic editor and the statistical reviewer.

If the data are freely or publicly available, note this and state the location of the data: within the paper, in Supporting Information files, or in a public repository (include the DOI or accession number).

* Please provide the source code needed to replicate the study's findings in a repository (such as GitHub, SourceForge or Bitbucket) or a cloud computing service (such as Code Ocean). Protection of authors’ intellectual property will not be cause for exception. Please explain in the manuscript’s Data Availability Statement how readers can access the shared code.

* Please provide the unadjusted comparisons as well as the adjusted comparisons in all relevant Tables.

* Please specify the variables controlled for in all relevant Tables, if relevant.

* Please show graph axes beginning at zero. If this is not possible, please show a break in the axis.

* Please confirm that the appropriate usage rights apply to the use of the map in the supporting information. Please see our guidelines for map images: https://journals.plos.org/plosmedicine/s/figures#loc-maps

* Please clarify for figures whether they include images of branded products or are generated by commercial products. Please confirm you have appropriate permission or you may wish to remove these images. For more information, please see our guidelines: https://journals.plos.org/plosmedicine/s/figures#loc-licenses-and-copyright and https://journals.plos.org/plosmedicine/s/licenses-and-copyright

Figures, tables, and images in PLOS journals are published under the Creative Commons Attribution (CC BY) license.

* Please clarify the reason for the inclusion of the 'terms of service' pdf in the file inventory.

Comments from Reviewers:

Reviewer #1: I would like to thank the authors for providing detailed responses to the comments that I had.

Reviewer #2: The manuscript has been excellently revised. Thank you for responding thoughtfully to my comments, and congratulations to the entire team on this valuable contribution to the epidemiological literature.

Reviewer #3: I'm fine with the responses, except on code & data.

Saying the code is available in the published manuscripts is weak: the point of the SR is to have gathered it. Saying it will be part of some database in the future is not a reason not to release it with this paper.

Similarly, saying you've used standard commands is not a reason not to release analysis code. It could still contain errors or steps that would facilitate reproduction of results by others.

Any attachments provided with reviews can be seen via the following link:

[LINK]

# Author comment: Author response to Decision Letter 3

**DOI:** 10.1371/journal.pmed.1004936.r005

16 May 2026

Attachment Submitted filename: Response_to_reviewers_auresp_3.docx

# Editor report: Decision Letter 3

**DOI:** 10.1371/journal.pmed.1004936.r006

**Authors:** Alison Farrell

19 May 2026

Dear Dr Abu-Raddad,

On behalf of my colleagues and the Academic Editor, Matthias Egger, I am pleased to inform you that we have agreed to publish your manuscript "Prevalence and epidemiological patterns of Neisseria gonorrhoeae infection in sub-Saharan Africa, 1964–2025: systematic review, meta-analyses, and meta-regressions" (PMEDICINE-D-26-00281R3) in PLOS Medicine.

Before your manuscript can be formally accepted you will need to complete some formatting changes, which you will receive in a follow up email. Please be aware that it may take several days for you to receive this email; during this time no action is required by you. Once you have received these formatting requests, please note that your manuscript will not be scheduled for publication until you have made the required changes.

In the meantime, please log into Editorial Manager at http://www.editorialmanager.com/pmedicine/, click the "Update My Information" link at the top of the page, and update your user information to ensure an efficient production process.

PRESS

We frequently collaborate with press offices. If your institution or institutions have a press office, please notify them about your upcoming paper at this point, to enable them to help maximise its impact. If the press office is planning to promote your findings, we would be grateful if they could coordinate with medicinepress@plos.org. If you have not yet opted out of the early version process, we ask that you notify us immediately of any press plans so that we may do so on your behalf.

We also ask that you take this opportunity to read our Embargo Policy regarding the discussion, promotion and media coverage of work that is yet to be published by PLOS. As your manuscript is not yet published, it is bound by the conditions of our Embargo Policy. Please be aware that this policy is in place both to ensure that any press coverage of your article is fully substantiated and to provide a direct link between such coverage and the published work. For full details of our Embargo Policy, please visit http://www.plos.org/about/media-inquiries/embargo-policy/.

To enhance the reproducibility of your results, we recommend that you deposit your laboratory protocols in protocols.io, where a protocol can be assigned its own identifier (DOI) such that it can be cited independently in the future. Additionally, PLOS ONE offers an option to publish peer-reviewed clinical study protocols. Read more information on sharing protocols at https://plos.org/protocols?utm_medium=editorial-email&utm_source=authorletters&utm_campaign=protocols

Thank you again for submitting to PLOS Medicine. We look forward to publishing your paper.

Sincerely,

Alison Farrell, Ph.D.

Senior Editor

PLOS Medicine
